# Integrated Regulation of the Type III Secretion System and Other Virulence Determinants in Ralstonia solanacearum

**DOI:** 10.1371/journal.ppat.0020082

**Published:** 2006-08-25

**Authors:** Marc Valls, Stéphane Genin, Christian Boucher

**Affiliations:** Laboratoire des Interactions Plantes-Microorganismes (CNRS-INRA), Chemin de Borde Rouge, Castanet Tolosan Cedex, France; University of North Carolina, United States of America

## Abstract

In many plant and animal bacterial pathogens, the Type III secretion system (TTSS) that directly translocates effector proteins into the eukaryotic host cells is essential for the development of disease. In all species studied, the transcription of the TTSS and most of its effector substrates is tightly regulated by a succession of consecutively activated regulators. However, the whole genetic programme driven by these regulatory cascades is still unknown, especially in bacterial plant pathogens. Here, we have characterised the programme triggered by HrpG, a host-responsive regulator of the TTSS activation cascade in the plant pathogen *Ralstonia solanacearum.* We show through genome-wide expression analysis that, in addition to the TTSS, HrpG controls the expression of a previously undescribed TTSS-independent pathway that includes a number of other virulence determinants and genes likely involved in adaptation to life in the host. Functional studies revealed that this second pathway co-ordinates the bacterial production of plant cell wall-degrading enzymes, exopolysaccharide, and the phytohormones ethylene and auxin. We provide experimental evidence that these activities contribute to pathogenicity. We also show that the ethylene produced by R. solanacearum is able to modulate the expression of host genes and can therefore interfere with the signalling of plant defence responses. These results provide a new, integrated view of plant bacterial pathogenicity, where a common regulator activates synchronously upon infection the TTSS, other virulence determinants and a number of adaptive functions, which act co-operatively to cause disease.

## Synopsis

Most pathogenic bacteria have the ability to switch between free-living growth and life within the host tissues. However, the mechanisms that co-ordinate changes in gene expression during the passage between these alternative ecological niches are still largely unknown. A well-studied regulation pathway triggered in response to the host environment is that controlling the transcription of the Type III secretion system (TTSS) genes. The TTSS is a major pathogenicity determinant that delivers bacterial effector proteins directly into the host cell cytosol to promote disease. Here, Valls and colleagues show that the TTSS regulatory pathway is directly connected with other circuits driving the expression of diverse pathogenicity and host-adaptation activities. The authors have identified and characterised the genes co-regulated along with the TTSS via the HrpG regulator. They have found that, in addition to the TTSS, HrpG controls the transcription of a previously unknown TTSS-independent pathway that is essential to pathogenicity and alters the bacterial production of plant cell wall-degrading enzymes, exopolysaccharide, and the phytohormones ethylene and auxin. These findings reveal an important degree of co-ordination between adaptation and virulence functions at the transcriptional level and contribute to a better understanding of the infection process.

## Introduction

Most pathogenic bacteria are nonobligate parasites that have the ability to switch between alternative ecological niches with extremely different characteristics. In the environment, the pathogen needs to mobilise scarce metabolic resources and vie with numerous competitors in order to survive. On the other hand, in the host, metabolic resources are abundant and competition with other micro-organisms is limited, allowing extensive multiplication of the pathogen provided it can cope with and escape host defences. Pathogens have therefore developed integrated regulation circuits to control the co-ordinated physiological switch associated with the passage from one environment to the other. So far, only limited knowledge concerning these global regulatory circuits in pathogenic bacteria is available, although master pathogenicity regulators have been identified in representatives of the main gram-negative taxa [1–6]. Amongst the best studied regulators are those driving the expression of the Type III secretion system (TTSS), a major pathogenicity determinant that delivers bacterial effector proteins directly into the host cell cytosol [7–9]. Transcription of the genes encoding the TTSS machinery and the related effectors is driven by regulatory cascades triggered in response to the host environment [8,10]. In plant bacterial pathogens, although different studies have been addressed to discover the targets of the downstream regulators [11–13], no exhaustive catalogue of the genes affected by regulators located upstream in the TTSS-activation cascades has been performed to date.

Ralstonia solanacearum is a β-proteobacterium responsible for bacterial wilt of over 200 plant species [14]. Plant infection takes place when the bacterium living saprophytically in the soil colonises the root. R. solanacearum subsequently multiplies in the apoplastic space, traverses the endoderm barrier, and invades the vascular system. The ultimate development of wilting symptoms is probably a result of intensive bacterial multiplication within xylem vessels and concomitant production of copious amounts of exopolysaccharides (EPSs) that block water traffic. As in other plant pathogens, the TTSS is essential for pathogenicity, since mutants affected in the *hrp* (hypersensitive reaction and pathogenicity) genes, encoding this machinery, are unable to cause disease [15]. In *R. solanacearum,* the regulatory cascade driving the expression of the TTSS is especially well characterised. A key component of this cascade is HrpG, a two-component response regulator of the OmpR subfamily. HrpG transcription is enhanced by a well-established pathway that responds to direct contact of the bacterium with plant cells [16]. Moreover, HrpG activity is increased by metabolic signals perceived in minimal culture medium through an as-yet-unknown mechanism [17]. In addition, a quorum-sensing signal has been shown to modulate the activity of HrpG through a PhcA-dependent regulatory pathway [18]. In turn, HrpG strongly enhances the transcription of HrpB, an AraC-family transcriptional activator situated at the bottom of the cascade. Following the sequencing and annotation of the R. solanacearum GMI1000 complete genome [19], microarray experiments and search for a consensus motif in gene promoters allowed the identification of the HrpB regulon. It was found that this downstream regulator promotes the co-ordinated expression of the TTSS structural genes and a large ensemble of ORFs containing 80 effector gene candidates [13,20–22]. Homologues for the HrpB and HrpG regulators have also been identified and studied in Xanthomonas sp., where a similar organisation and regulation of the *hrp* cluster is found [23,24]. In addition, analogous multistep activation cascades for the *hrp* genes and related effectors have been described in bacteria from the genera *Pseudomonas* and *Erwinia* [25–27].

To get a global view of the multifaceted genetic programme that takes place during infection, we have found and characterised in this work the targets of the HrpG transcriptional regulator. We propose a pivotal role of HrpG in the transition from saprophytic to parasitic life by demonstrating that it co-regulates the expression of TTSS and cognate effector genes to that of additional pathogenicity determinants and genes likely involved in adaptation to life in the host. The complexity of genetic cross-talk between pathogenicity functions described here denotes that an intricate genetic network is at the basis of the bacterial disease program.

## Results

### Identification of a Large HrpG Regulon Organised in Two Main Pathways

In order to establish the magnitude of the complete HrpG regulon we used a pangenomic microarray [13] to compare the R. solanacearum GMI1000 transcriptome to that of strains mutated for *(ΔhrpG)* or overexpressing this regulator (GMI1000-pBBL12), all grown to late exponential phase in an *hrp*-inducing minimal medium. Using as general threshold a greater than 4-fold difference in the mRNA levels in any of the two comparisons, we detected some 300 genes whose expression was affected by HrpG (Table S2). This threshold value had been established as highly discriminative in our previous work [13].

Since HrpG acts upstream of HrpB in the regulatory cascade, all ORFs detected already known to be regulated by the latter [13] were found differentially expressed in strains altered for HrpG, which constituted an internal validation of the assays. We also added 29 ORFs to the HrpB regulon, as their log differences were greater than 1.5 in our previous studies [13] and appeared to be greater than 2 in the HrpG transcriptomic experiments performed here. But a whole set of ORFs was identified as affected by HrpG in an HrpB-independent manner. To better define this new class of HrpG-regulated genes, additional transcriptome experiments were performed to compare the wild-type strain to an *hrpB*-mutant derivative that overexpressed HrpG from plasmid pBBL12 (*hrpB::Ω*/pBBL12). In the latter strain, mRNA transcripts for genes whose expression is governed by HrpG independently from the HrpB regulator should be differentially represented with respect to the wild-type. Ninety-eight ORFs were detected as up-regulated and 50 as down-regulated by HrpG in this strain. These genes, together with 24 ORFs of the HrpG regulon whose mRNA levels were below detection limit in this experiment but that were clearly unaffected by HrpB [13], were all considered as regulated by HrpG independently from HrpB. In summary, the identified HrpG regulon comprised 399 genes, of which 222 were regulated through HrpB and 184 were modulated directly or through as-yet-unidentified circuits (Table S1). In this sense, no clear and robust consensus motif could be identified in the promoters of the latter subset of genes, which is probably due to the fact that some of them are not direct targets of HrpG. As shown by the previous figures, seven ORFs were found as regulated through HrpG via both pathways. Therefore, HrpG appears to be an important branching node in the *hrp* regulation cascade, affecting positively 112 and negatively 72 ORFs in addition to the well-known HrpB-dependent pathway. This new set of genes is referred hereafter as the HrpB-independent HrpG regulon.

### HrpG Controls the Biological Activity of Virulence Factors beyond the TTSS

A closer scrutiny of the HrpB-independent HrpG-activated ORFs revealed that they contain a significant proportion of membrane proteins (35.5%) and putative enzymes but no predicted Type III effector candidates (Table 1). However, we identified several established and putative virulence genes involved in (1) plant cell wall degradation, (2) phytohormone production, (3) attachment, and (4) bacterial protection responses, together with a set of genes encoding proteins of other predicted functions, including four transcriptional regulators. The class of genes repressed by HrpG independently of HrpB also contained a number of metabolic enzymes and several transcriptional regulators. Finally, an important group of genes corresponded to hypothetical/unknown functions with no similarities in the databanks (49 up-regulated and 31 down-regulated by HrpG).

**Table 1:**
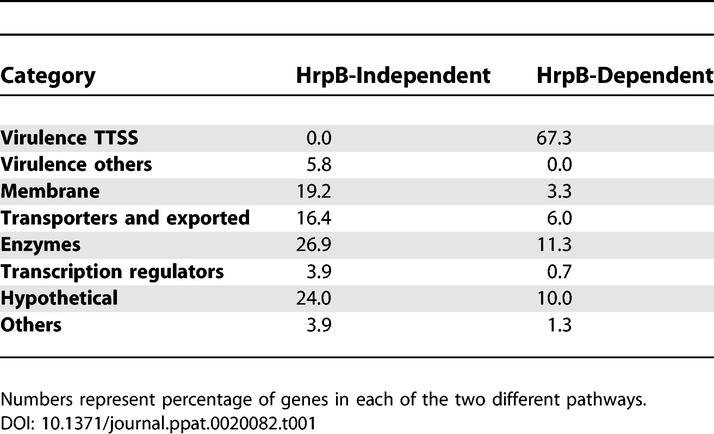
Functional Categories of Genes Positively Regulated by HrpG Belonging Either to the HrpB-Dependent or to the HrpB-Independent Pathway

**Figure 1:**
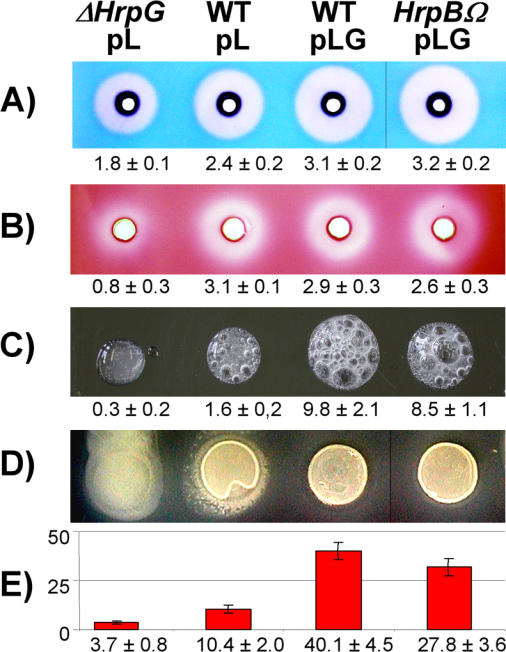
The HrpG Regulator Affects Virulence and Host Colonisation Factors in an *hrpB*-Independent Manner Enzymatic activities for (A) endoglucanase, (B) polygalacturonase, and (C) catalase, as well as (D) production of EPS and (E) *efe* gene expression, are presented. Biological tests were performed in an HrpG-deficient strain *(ΔhrpG)* and the wild-type GMI1000 strain (wild-type), both bearing the empty vector pL, as well as in the wild-type strain bearing plasmid pLG, which overexpresses HrpG. An *hrpB* mutant overexpressing HrpG (*hrpB::Ω*/pLG) was also included in this set of experiments to check the influence of HrpB in the regulation. Measured values with standard deviations are presented below quantitative tests [units: (A, B): halo surface in cm^2^, (C) foam height in cm, (E) Miller units].

We performed biological tests to functionally validate several of the TTSS-independent putative virulence functions found in the HrpG genome-wide expression analyses (Figure 1). Endoglucanase, polygalacturonase, and catalase activities, as well as EPS formation, were measured in the wild-type GMI1000 strain and its *hrpG*-derivative *(ΔhrpG),* both bearing the empty vector pL, and in the wild-type strain bearing plasmid pLG, which overexpresses HrpG from an inducible *Ptac* promoter. Results shown in Figure 1 prove that the changes in gene expression observed through transcriptomics were translated into significant differences in the predicted activities. Both the endoglucanase and catalase activities were decreased in the *hrpG* mutant background, and the inverse effect was observed upon overexpression of the regulator (Figure 1A and 1C). For polygalacturonase, a significant decrease of activity was detected only in the *hrpG* mutant background (Figure 1B). In the case of EPS, its production was increased in the mutant and diminished in the strain containing pLG, a result expected since *epsR,* a predicted target of HrpG identified from microarray expression data, encodes a repressor of EPS biosynthesis [28].

The regulation of RSp1529 which encodes a putative ethylene-forming enzyme was assessed by using a genomic fusion with the *lacZ* reporter gene that had been introduced into the above-mentioned strains (Figure 1E). Quantitative RT-mediated real-time PCR was also employed to confirm the HrpG dependency of the expression of both lectins, two serine proteases, two ORFs involved in polyamine synthesis, three ORFs belonging to the indole/auxin metabolism operon, and an *avrD*-related gene (Table 2). Despite its name, *avrD* does not encode a Type III effector but directs the synthesis of a small diffusible molecule in P. syringae that elicits a hypersensitive response on resistant host plants [29].

**Table 2:**
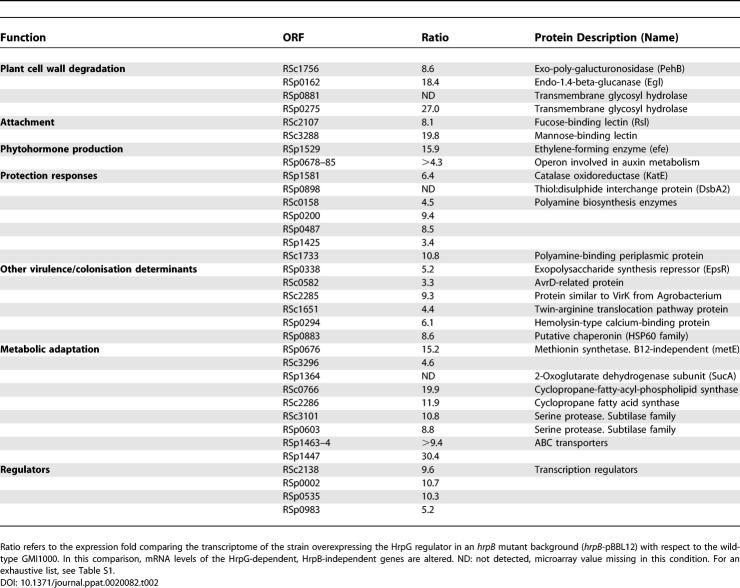
A Subset of Genes with Known Function Whose Expression Is Dependent on HrpG but Not on HrpB, including Several Known or Candidate Pathogenicity Functions

### HrpG Specifically Activates Bacterial Production of Plant Hormones

**Figure 2:**
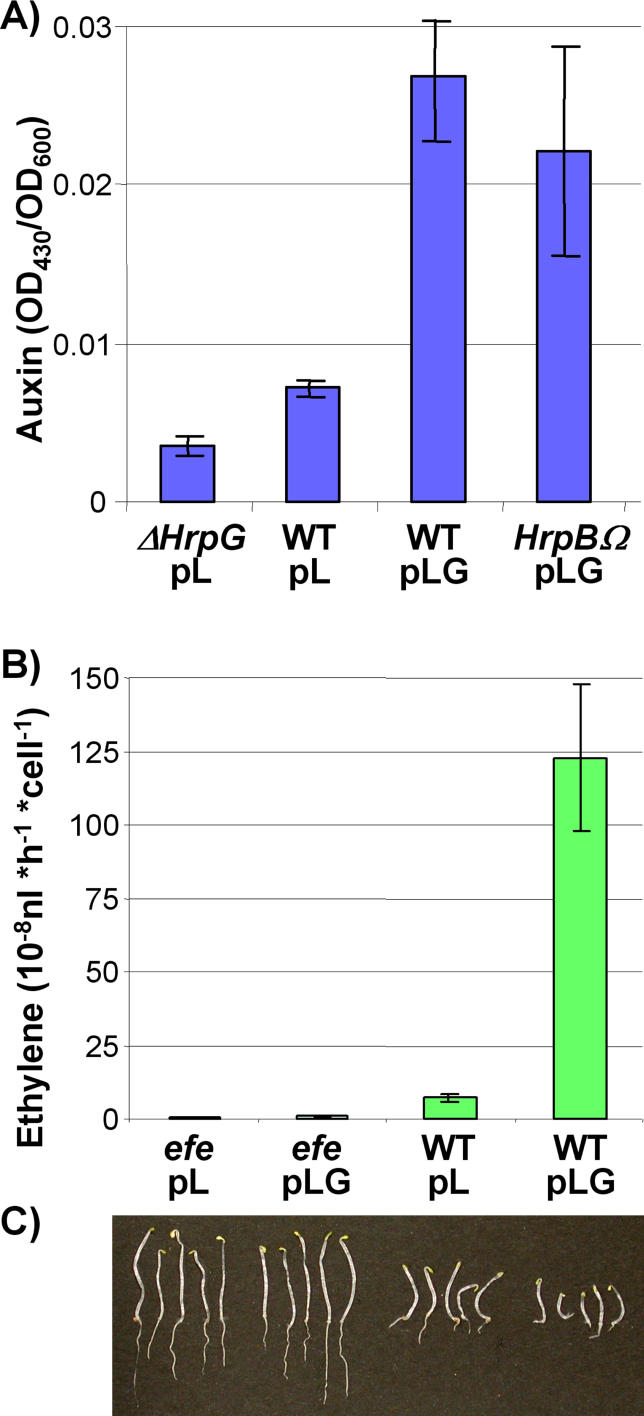
HrpG Promotes Phytohormone Production in R. solanacearum (A) Auxin production by a strain deleted for *hrpG* (Δ*hrpG),* the GMI1000 reference strain (wild-type), an HrpG overproducer (wild-type/pLG), and a strain overproducing the regulator in an *hrpB* mutant background (*hrpB::*Ω/pLG). Auxin concentrations in culture medium of overnight-grown cultures are presented as OD_530_/OD_600_ ratios. (B) Ethylene production by wild-type R. solanacearum and an ethylene-forming enzyme mutant *(efe)* bearing the pL empty vector or its *hrpG*-overexpressing derivative (pLG). The hormone was measured by chromatography from the gas phase of overnight-sealed cultures. (C) Triple-response assay with bacterial ethylene. Gas phases of cultures equivalent to those in (B) were injected onto sealed flasks containing A. thaliana seedlings photographed after 96 h of growth in the dark. The extent of the triple-response symptoms (cotyledon curvature, hypocotyl shortening, and root growth inhibition) is correlated to ethylene production.

The existence of a cluster of HrpG-dependent genes putatively involved in indole metabolism (RSp0678–85) led us to hypothesise that it could drive auxin synthesis. In order to determine whether auxin production in R. solanacearum was influenced by HrpG, we quantified the indoleacetic acid (IAA) present in culture supernatants of the wild-type, the HrpG-deficient, and the HrpG-overexpressing strains. We found that IAA concentrations were 3-fold higher upon *hrpG* overproduction with respect to the wild-type and decreased in the *hrpG* mutant (Figure 2A). Auxin production was also monitored in the *hrpB::Ω*/pLG strain to establish that it was not dependent on HrpB.

We also investigated the function of the HrpG-regulated gene RSp1529 which encodes a predicted ethylene-forming enzyme (efe). We constructed a mutant strain (*efe::Ω*) and analysed the capability of this mutant and of the wild-type strain to synthesise ethylene. These two strains carrying the empty pL plasmid were grown in sealed flasks in minimal medium and the ethylene content of the gas phase was measured. As shown in Figure 2B, the strain GMI1000 generates ethylene at levels comparable to recognised ethylene producers such as Pseudomonas syringae pv. *sesami* and 2 logs higher than the R. solanacearum K60 strain [30]. On the opposite, this capacity was almost undetectable in the *efe::Ω* mutant. Ethylene quantification from sealed cultures of GMI1000/pLG and its *efe::Ω*/pLG derivative, both overexpressing HrpG from the plasmid, indicated that the level of this phytohormone increased over 10-fold by HrpG overproduction and that this increase was only observed when a functional RSp1529 gene was present (Figure 2B). Complementation of the *efe::Ω* mutant with a plasmid bearing a wild-type version of the *efe* gene (RSp1529) confirmed that production of the hormone was strictly dependent on this gene (unpublished data). To further confirm ethylene production, the same samples used for gas chromatography were injected into sealed flasks containing Arabidopsis thaliana germinating seeds. After 96 h, the characteristic ethylene triple response was observed on the plantlets treated with gas from wild-type bacteria. These effects were still more apparent with the HrpG-overproducing strain bearing an intact *efe* gene but unnoticeable in experiments with the *efe* mutant (Figure 2C). In conclusion, these results provide the first experimental evidence that both ethylene and auxin production is enhanced simultaneously with TTSS expression in a plant pathogen.

### Bacterial Ethylene Production Modulates the Expression of Plant Genes

**Figure 3:**
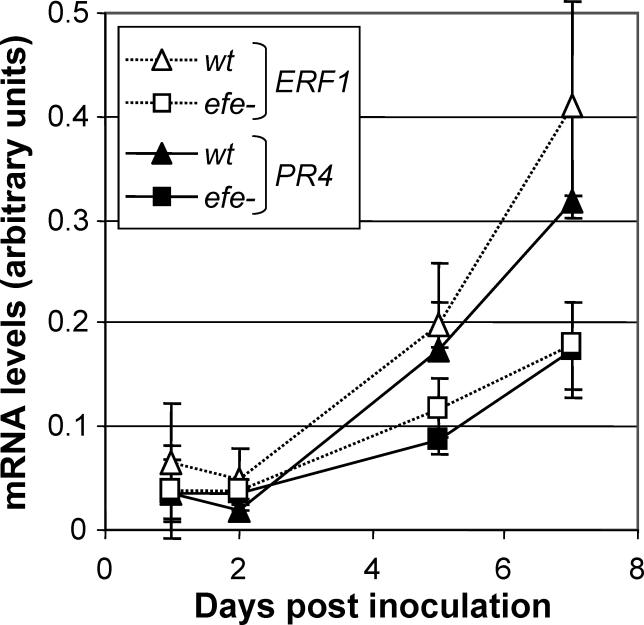
Effects of Ethylene Produced by R. solanacearum on the Plant Host Expression of A. thaliana ethylene-responsive genes upon inoculation with GMI1000*.* mRNA levels of the ethylene response factor 1 gene (*ERF1,* hollow symbols and dotted lines) and the pathogen response gene 4 (*PR4,* shaded symbols) were measured by quantitative real-time PCR during infection by a wild-type (triangles) or an ethylene-deficient strain (squares).

To check whether the amount of ethylene produced by bacteria in the plant was sufficient to be perceived by the host and to affect its physiology, we inoculated *Arabidopsis* plants with the GM1000 strain and its *efe* mutant derivative and measured the mRNA levels of various plant ethylene-responsive genes during infection. As illustrated in Figure 3, the mRNA levels of both *ERF1* (ethylene response factor 1) and *PR4* (pathogen response gene 4) increase gradually during plant colonisation by GMI1000. Both genes attain an approximately 20-fold higher expression at day 7 post inoculation (a time when wilting symptoms are clearly visible) with respect to day 2. On the opposite, when plants were inoculated with the *efe* mutant strain, the disease evolved at the same pace as for a wild-type strain, but the plant ethylene- and pathogen-responsive genes were markedly less induced at advanced stages of the disease (Figure 3). Similar results were obtained using the *PR3* gene (unpublished data), which occupies a position comparable to *PR4* in the plant transcription cascade responding to pathogen infection.

### Uncoupling of the HrpB/HrpG Regulation Cascade Reveals that HrpG-Regulated Functions beyond the TTSS Are Essential to Pathogenicity

**Figure 4:**
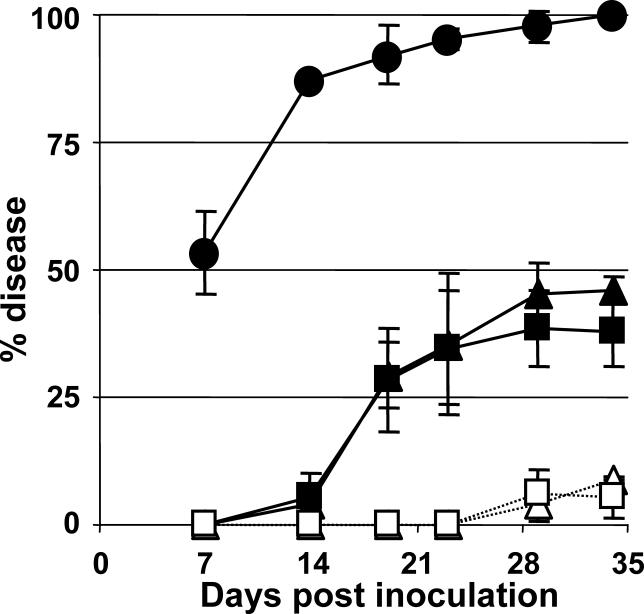
Influence of the *hrpG*-Regulated Functions Belonging to the TTSS-Independent Pathway on R. solanacearum Pathogenicity Pathogenicity tests on tomato plants. In the *Pkan::hrpB* (triangles) and *Ptac::hrpB* (squares) strains, transcription of the TTSS is constitutive and uncoupled to HrpG. These strains (solid symbols) and their *ΔhrpG* counterparts (hollow symbols and dotted lines) were used to inoculate tomato plants, as well as the wild-type strain GMI1000 (circles). The percentage of wilting recorded upon time is represented. In all cases, points indicate mean values and error bars indicate standard deviations of at least three experiments.

Considering (1) the large repertoire of HrpG-regulated genes and (2) that these target genes could have a collective and synergistic effect on bacterial virulence, which might be limiting for a single-gene disruption approach, we decided to test the global contribution of these HrpG-responsive genes to pathogenicity. To do this without impeding TTSS expression—because a TTSS mutant is totally nonpathogenic—we designed a strain in which the transcription of the HrpB regulator was constitutive and no longer responded to HrpG. Hence, the entire *hrpB* promoter was replaced by the *Pkan* promoter in its genomic context, to obtain strain *Pkan::hrpB.* Similarly, a *Ptac::hrpB* strain, bearing the inducible *Ptac* promoter instead of *Pkan,* was also constructed. We then introduced the *Pkan::hrpB* and the *Ptac::hrpB* fusions in the *ΔhrpG* background. Tomato plants were inoculated with the *Pkan::hrpB* and *Ptac::hrpB* strains and their *ΔhrpG* derivatives, and the wilting symptoms were quantified over time. IPTG was added to the bacterial cultures and to the inoculum of strains containing *Ptac* to ensure *hrpB* transcription. Results presented in Figure 4 show that the strains bearing *hrpG* and constitutively expressing the TTSS, through either *Ptac* or *Pkan,* were pathogenic on tomato, producing 39% to 45% of wilting 30 d after inoculation, whereas the same strains devoid of HrpG were almost nonpathogenic (approximately 6% of wilting). Strains constitutively expressing the TTSS did not show the aggressiveness of the wild-type strain (97% wilting at day 30), probably due to inadequate levels or timing of transcription of this major virulence determinant. Despite this, the experiments uncoupling the HrpB-dependent pathway from the rest of genes governed by HrpG definitely demonstrated that, collectively, the latter play an important role in pathogenicity.

## Discussion

**Figure 5:**
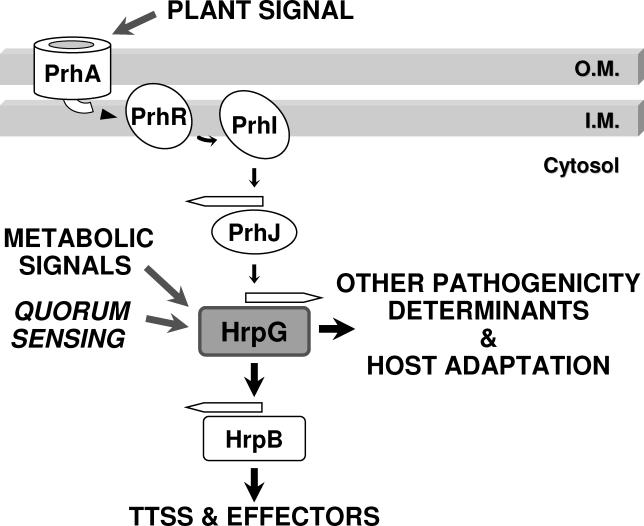
Schematic Model of the Hrp Regulation Cascade in R. solanacearum A plant signal is sensed by the outer-membrane receptor PrhA and is transduced to PrhJ through PrhI/R [63]. HrpG is the key/central component that integrates in the cascade at least three signals, as its activity depends on the plant host contact [16], bacterial metabolic signals related to growth conditions [17], and a phc-dependent quorum sensing signal [18]. HrpG regulates the expression of the TTSS apparatus and effector genes (through HrpB activation), and it controls as well the newly described HrpB-independent functions that also contribute to pathogenicity. Rounded forms symbolise proteins, and open arrows, gene sequences.

The data presented here describe a regulatory network responsible for the control of central aspects of R. solanacearum virulence including the TTSS. Our results show that HrpG directs the expression of two independent pathways (Figure 5). The first one is the well-established HrpB-dependent pathway mainly devoted to the expression of the TTSS structural genes and the associated effectors [13,21]. The second one has emerged from this study, is independent of HrpB, and does not include any of the previous functions but other virulence/host colonisation determinants and a panoply of activities putatively involved in adaptation to life in planta (Table 1). HrpG had been previously shown to assimilate three major signals: the physical contact with the plant host [16], the bacterial metabolic status [17], and a quorum-sensing signal [18]. It thus appears that this regulator plays a pivotal role in the molecular switch between saprophytic and pathogenic lifestyles responding to signals perceived during the soil/plant environment transition by shifting the expression of a large set of genes in addition to those driving the biogenesis of the TTSS (Figure 5). Twelve of the genes newly identified as activated by *hrpG* had been previously isolated in a nonexhaustive IVET screening in tomato [31], confirming their induction during the infection programme. Also supporting a role of HrpG as a master regulatory gene is the finding that it appears to control the expression of several downstream regulators (ten candidates). Hrp regulatory genes therefore orchestrate a complex genetic program since HrpB also mediates gene regulation through other downstream regulatory components [13]. The characterisation of these transcriptional regulators under the control of HrpG or HrpB and their respective targets deserves further study in an attempt to define the relative contribution of the different subsets to pathogenicity.

The HrpG-dependent pathway described here modulates the expression of several previously established virulence genes (encoding EPS biosynthesis and plant cell wall–degrading enzymes) and other candidates (such as phytohormone genes). Our data prove that this pathway is essential for full virulence independently of the *hrpB*-dependent activities, since a Δ*hrpG* strain constitutively expressing *hrpB* is strongly altered in its ability to wilt tomato plants (Figure 4). Amongst the HrpG target genes we identified, at least three were reported to individually affect pathogenicity: the endoglucanase *egl* [32], the exo-poly-α-d-galacturonosidase *pehB* [33], and the EPS regulator *epsR* [28]. Although the individual effect of these genes to virulence appears to be relatively minor, their additive contribution could account for the strongly reduced virulence observed with the Δ*hrpG Pkan*::*hrpB* mutant strain. This is reminiscent of the situation found with the TTSS effectors, where disruption of a single gene generally did not lead to virulence reduction [21]. However, several other candidate genes identified here might also be involved in virulence, and further work will be needed in order to clarify their role in the process.

The finding that endoglucanase and polygalacturonase activities are specifically enhanced by HrpG may also provide a clue to understand the previously observed infection phenotype of an *hrpG* mutant strain. Although both the *hrpB* and *hrpG* mutants are completely nonpathogenic, a former report revealed a major phenotypic difference in the root infection process: an *hrpG*-deficient strain was found to be incapable of reaching the plant vascular system, whereas an *hrpB* mutant could still traverse the endodermis [34]. The diminished cellulase and pectinolytic activities detected in the *hrpG* mutant may explain its compromised invading capacity and suggests that contribution of these plant cell wall–degrading enzymes to colonisation is prevalent over a nutritional role. This hypothesis is also supported by the recent finding that catabolism of the main degradation products of pectin does not contribute significantly to bacterial fitness inside the plant [35].

This work establishes for the first time a direct regulatory link between the expression of the TTSS and that of a wide array of other virulence functions including bacterial production of host hormones. Both ethylene and auxin have long been known to be produced by certain R. solanacearum strains [36,37]. The discovery that production of these phytohormones is controlled by an *hrp* master regulatory gene whose activity is induced in presence of plant cells strongly suggests that these molecules play a key role during the early steps of infection in addition to the TTSS. Interestingly, it is known that the auxin IAA produced by Pseudomonas syringae pv. *savastanoi* is crucial for the inhibition of plant defences [38] and ethylene appears to be implicated in wilt symptom development in the *R. solanacearum/A. thaliana* pathosystem [39] and in infected banana trees [36]. Our data prove that the ethylene produced by strain GMI1000 is sufficient to impact the plant ethylene-responsive pathway. Therefore, bacterial production of ethylene may be a means to unbalance the plant defence responses depending on this hormone in order to favour infection of the pathogen. A recent report indicated that two P. syringae pv. *tomato* TTSS effectors activate a set of plant genes involved in ethylene biosynthesis and signalling and showed that an efficient ethylene production pathway in the host is required for full activity of these bacterial effectors in virulence [40]. In the light of this functional relationship, our observation of a co-ordinated expression of TTSS effector genes and an ethylene production gene in R. solanacearum suggests that hormone production could complement the action of some TTSS effectors. Alternatively, R. solanacearum could have developed a different solution for modulating the ethylene-responsive plant defences by increasing its own ethylene production upon infection of the host plant.

Amongst the other determinants identified in this genome-wide expression analysis for which their *hrpG* dependency suggests that they are important during the infection process is a group of genes governing protective functions. Amongst the genes found in this class are the only predicted catalase enzyme in the genome *(katE)* and five enzymes driving the synthesis of polyamines (Table 2). Interestingly, catalase is also induced in Pseudomonas syringae during plant colonisation [41], and in *Yersinia pestis,* a catalase-peroxidase was found to be co-expressed with the TTSS and to prevent bacterial killing by phagocytes [42]. It seems therefore likely that at the onset of infection, R. solanacearum cells encounter an oxidative environment and require active protective systems against oxidative damage to overcome these conditions. The genetically unlinked HrpG-activated genes involved in ornithine/polyamine metabolism constitute a biochemical pathway under HrpG control that might increase or decrease the polyamine pool, an attractive hypothesis considering that polyamines have been shown to inhibit animal defences against the intestinal pathogen Helicobacter pylori and might also influence plant resistance [43,44].

Finally, another example of genes positively controlled by HrpG indicates that adhesion to plant surfaces may be important for R. solanacearum during the early steps of plant infection. The two characterised lectin genes in strain GMI1000 [45,46] are both under the control of *hrpG*. Structural studies of these lectins recently showed that they strongly bind l-fucose and interact with the plant xyloglucan polysaccharide, which is part of the hemicellulose fraction in plant primary cell walls [46]. It has been shown that adhesion to plant surfaces is important for R. solanacearum pathogenicity, a character which is partially governed by Type IV pili [47], and we speculate that these two lectins could also play a specific role in the process.

This is the first exhaustive genome-wide search in a plant pathogen for the targets of a master regulatory gene in TTSS pathogenicity cascades, and it provides a comprehensive view of large-scale regulation circuits that monitor the expression of multiple virulence factors. It also provides a broad list of hypothetical/unknown HrpG-regulated genes which constitute candidate virulence genes since their expression is specifically induced by contact with the host. Co-regulation of the genes important for pathogenicity may be a widespread property shared by plant and animal bacterial pathogens, as, for instance, TTSS regulators from Salmonella enterica and Pseudomonas syringae pv. *tomato* have been found to alter the transcription of at least one gene involved in virulence and unrelated to the TTSS [11,12]. Moreover, synchronised expression of genes encoding components of the Type III secretion apparatus or the associated effectors and other virulence genes has been detected in several animal pathogens subjected to transcriptomic scrutiny, even if no direct regulatory connections have been yet established [42,48–52]. When DNA microarray technology becomes available for other Type III bacterial plant pathogens, it will be extremely interesting to test whether similar co-regulation circuits between the different virulence loci exist. This will also provide the opportunity to unveil the degree of overlap in the pathogenicity determinants and host adaptation functions co-regulated with the TTSS in different pathogens.

## Materials and Methods

### Plasmids, strains, and growth conditions.

Throughout this study, pL stands for the empty vector pLAFR3 [53], and pBBL12 for a pLAFR3 derivative bearing the *hrpG* gene under the control of its native promoter [17]. Plasmid pLG is a pL derivative that contains a His-tagged version of *hrpG* (which was shown to complement an *hrpG* mutation) transcribed from the isopropyl-β-d-thiogalactopyranoside (IPTG)-inducible *Ptac* promoter. To create pLG, an EcoRI-HindIII fragment containing the coding sequence for 6His-HrpG was cloned into the same sites in pTGm. Plasmid pTGm is a pET-26b(+) derivative (Novagen, Madison, Wisconsin, United States) in which the T7 promoter was replaced by the P*tac* promoter sequence and the kanamycin resistance gene was substituted by the gentamycin resistance cassette (M. Valls, unpublished data). To enable replication in *R. solanacearum,* the pTGm-hrpG vector was linearised by digestion with KpnI and ligated into the unique KpnI site of pLAFR3, to yield pLG.

The wild-type strain GMI1000 of R. solanacearum and its *hrpB*-derivative GMI1525 have been previously described [20,54]. In GMI1525 (hereafter referred as *hrpB::*Ω), the *hrpB* coding region is disrupted by an Ω cassette [55] conferring streptomycin/spectinomycin resistance. Strain GMI1000 *ΔhrpG* bears a deletion encompassing the whole *hrpG* coding region using the cre/lox system described by A. Angot et al. (unpublished data). The R. solanacearum strains presented below were obtained by electroporation with linearised DNA fragments and selection of double recombination events [20]. Strains *efe::lacZ and efe::Ω* carry, respectively, a genomic fusion of a promoterless *lacZ* reporter gene at position 972 of ORF RSp1529 *(efe)* and a disruption of this same coding sequence at position 580 (EcoRI site) by the Ω interposon. In strain *Pkan::hrpB,* the sequence upstream of the predicted Shine-Dalgarno sequence in *hrpB* (from position −13 to −107 with respect to the ATG) was replaced by the constitutively expressed *Pkan* promoter from pME6010 [56]. Likewise, in *Ptac::hrpB* the *hrpB* promoter was replaced by the inducible *Ptac* promoter. All genomic deletions and insertions were confirmed by Southern blotting [57]. All mutants were generated on the wild-type GMI1000 background and subsequently introduced in the strains of interest by natural transformation of genomic DNA and selection with the appropriate antibiotic markers. When required, strains were transformed by electroporation with the empty vector pL, or the *hrpG*-overexpressing vectors pBBL12 or pLG.

R. solanacearum was routinely grown at 28 °C in rich B medium or in minimal medium supplemented with 20 mM glutamate and the required antibiotics [54]. Escherichia coli culture conditions and general molecular biology techniques have been described [57].

### Transcriptome analysis.

The *R. solanacearum* whole-genome DNA microarray, which includes 65/70-mer oligonucleotides specific for the annotated ORFs spotted onto aldehyde-activated glass slides, was used for all transcriptome experiments [13]. RNA extraction, probe labelling, microarray hybridisation, signal quantification, and data analysis were performed as explained elsewhere [13]. RNAs were extracted from cultures grown to OD_600_ = 0.8 in minimal medium with glutamate, and the same reference condition (the GMI1000 wild-type strain) was always used. For each experimental condition, at least two RNA preparations from independent cultures were used, and a minimum of four hybridisations were performed swapping dye labelling. In order to minimise false positives, only genes with high levels of significance (*p* < 0.05 in Student's *t*-test) and an absolute Log_2_ ratio with respect to the reference condition greater than 2 were considered in this study. Exceptionally, ORFs with Log_2_ differences in expression levels greater than 1.5 but that laid in the genome adjacent to and in the same orientation as those previously selected were also retained. Genes belonging to the HrpG regulon were identified as transcripts whose levels were altered beyond the threshold in any of the comparisons between *hrpB::Ω*-pBBL12, GMI1000-pBBL12, or *ΔhrpG*-pL and the reference GMI1000-pL strain. Similarly, the HrpB-independent HrpG regulon was defined as the ORFs in the HrpG regulon shown to be unaffected by HrpB in the previous transcriptomic studies [13]. All primary data from transcriptome experiments as well as experimental protocols used and strain comparisons are available from the ArrayExpress depository (accession number E-MEXP-579 at http://www.ebi.ac.uk/arrayexpress).

### Quantitative RT-PCR.

Quantitative RT-mediated real-time PCR was performed as described [13] using a Light Cycler (Roche, Basel, Switzerland) with SYBR Green chemistry. A. thaliana total leaf RNAs were obtained with the Nucleospin RNAII kit (Macherey-Nagel, Düren, Germany), and R. solanacearum RNA samples were isolated as described above. The sequence of oligonucleotide primers and PCR conditions are available upon request.

### Biochemical assays.

All biochemical assays were performed at least three times from independent cell cultures grown in the HrpG-activating minimal medium supplemented with 100 μM IPTG. For quantification and visualisation of polygalacturonase and endoglucanase activities, supernatants from cultures grown to OD_600_ = 1 were concentrated 10-fold in a centricon with a 3-kDa pore membrane and 90 μl was settled in wells on test plates. Endoglucanase tests were carried out as in [58], and polygalacturonase activity was assayed by staining with 0.1% ruthenium red after 24 h growth on minimal medium plates supplemented with 0.125% polygalacturonic acid. In both cases, enzymatic activities were quantified as the surface of the halos produced by polymer degradation. To visualise catalase activity, bacterial strains grown on minimal medium agar plates for 2 d at 28 °C were resuspended at 10^10^ cells/ml in distilled water, 50 μl of the suspensions was added to 300 μl of 3% H_2_O_2_ deposited on a glass surface, and the reactions were immediately photographed. For a precise measurement of the activity, we developed a simple test to quantify hydrogen gas production. Briefly, the bacterial suspensions mixed with 3% H_2_O_2_ were added with two drops of mineral oil to facilitate foaming, the mix was immediately introduced into a graduated 1-ml plastic pipette with the bottom sealed, and the maximal height attained by the foam was recorded. Exopolysaccharide production was observed as a mucoid halo produced after 2 d of growth of a 10-μl drop of bacteria (10^8^ cells/ml) on minimal medium plates. β-Galactosidase activity was measured from overnight cultures grown in minimal medium with glutamate and IPTG following the method of Miller [59].

### Phytohormone quantification and pathogenicity tests.

The Salkowski colorimetric assay [60] was used to measure auxin produced from supernatants of R. solanacearum grown in minimal medium supplemented with glutamate, IPTG, and the required antibiotics. Auxin production was expressed as the measured OD_530_ divided by the OD_600_ of the original culture. To determine ethylene production, 5 ml of bacterial cultures (OD_600_ of approximately 0.5) were grown in sealed 25-ml flasks. After 12 h, 1 ml of the gas phase was injected in a gas chromatograph coupled to a flame ionisation detector, and ethylene concentrations were calculated comparing the height of the obtained peaks to a 25 ppb standard. Values are the mean of six measurements from three independent cultures and are expressed as 10^−8^ nl of ethylene/hour/cell. The triple response test [61] was performed by injecting 20 ml of gas phases in sealed 50-ml Erlenmeyer flasks containing *A. thaliana* seedlings.

Axenic pathogenicity tests were carried out using 10^8^ bacterial cells to inoculate 1-week-old tomato plants as described [54]. Wilting symptoms were scored over time, and average values for 80 plants were calculated and represented as percentage of disease. For plant gene expression experiments, A. thaliana was inoculated with R. solanacearum following a standard protocol [62]. Symptoms were annotated to check that disease development was similar for all strains tested, and mixtures of five plants were sampled at different times, immediately frozen in liquid nitrogen, and kept at −80 °C for RNA extraction.

## Supporting Information

Table S1 Complete Transcriptome Data for the ORFs Belonging to the HrpG Regulon (HrpB-Dependent and HrpB-Independent) Ratios in each condition are expressed in Log_2_, referred to the wild-type strain GMI1000, and followed by their *p*-value as obtained in a Student's *t*-test. Boxes represent probable operons. For strain names, see the Materials and Methods section.
(130 KB PDF)

Table S2 Whole Transcriptome Data Obtained in This Study for the Annotated ORFs in the R. solanacearum Genome In all cases, RNA levels of the tested *hrpB::Ω*-pBBL12, GMI1000-pBBL12, or *ΔhrpG*-pL strains are expressed as Log2 ratios with respect to the GMI1000 strain bearing the empty vector pL. Results previously obtained in HrpB regulon analyses overexpressing or mutating this regulator (*hrpB::Ω* and GMI1000-pBBL12) [13] are also included. Experimental design, strain comparisons, and original hybridisation data are detailed under accession number E-MEXP-579 at the ArrayExpress depository (http://www.ebi.ac.uk/arrayexpress).
(335 KB PDF)

### Accession Numbers

The EMBL Nucleotide Sequence Database (http://www.ebi.ac.uk/embl)/GenBank (http://www.ncbi.nlm.nih.gov/Genbank) accession numbers used in this paper are HrpG (CAD18003), HrpB (CAD18024), RSp1529 (CAD18680), *avrD*-related gene (CAD14112), *egl* (CAD17313), *pehB* (CAD15458), *katE* (CAD18732), and *epsR* (CAD17489). The GenBank accession numbers are ERF1 (ethylene response factor 1) (AAD03545) and PR4 (pathogen response gene 4) (NP187123). The Swiss-Prot (http://www.ebi.ac.uk/swissprot) accession number is *PR3* (P19173).

Experimental design, strain comparisons, and original hybridisation data are detailed under accession number E-MEXP-579 at the ArrayExpress depository (http://www.ebi.ac.uk/arrayexpress).

## Abbreviations

EPS: exopolysaccharide
IAA: indoleacetic acid
IPTG: isopropyl-β-d-thiogalactopyranoside
TTSS: Type III secretion system
