# Supplementary material for: Integrated Regulation of the Type III Secretion System and Other Virulence Determinants in Ralstonia solanacearum
Source: PLoS Pathog. 2006 Aug 25;2(8):e82. doi: 10.1371/journal.ppat.0020082 (PMC1557829; doi:10.1371/journal.ppat.0020082)
Supplement: Table S1 — Ratios in each condition are expressed in Log2, referred to the wild-type strain GMI1000, and followed by their p-value as obtained in a Student's t-test. Boxes represent probable operons. For strain names, see the Materials and Methods section. (130 KB PDF) [file ppat.0020082.st001.pdf]

>-4	>4
>-3	>3
>-2	>2
>-1	>1
>-0,8	>0,8
>-0,5	>0,5

Boxes represent probable operons

ID	*ΔhrpG*		*hrpB Ω pBBL12*		Gene	Description
**HrpG-activated independently from HrpB**						
RSc0107			2,08	0,00		CONSERVED HYPOTHETICAL PROTEIN
RSc0117			2,16	0,00		CONSERVED HYPOTHETICAL PROTEIN
RSc0118			2,42	0,00		CONSERVED HYPOTHETICAL PROTEIN
RSc0158			2,17	0,00		ARGINASE
RSc0215	-1,72	0,01	2,19	0,01		OXIDOREDUCTASE COG1028
RSc0217			2,82	0,00		OXIDOREDUCTASE COG0667
RSc0232	1,16	0,01	4,53	0,00		HYPOTHETICAL PROTEIN
RSc0293			2,70	0,00		SIGNAL PEPTIDE
RSc0581	-1,11	0,00	1,88	0,00		PHOSPHOGLYCOLATE PHOSPHATASE
RSc0582	-1,98	0,00	1,70	0,00		P. syringae AVRD-RELATED PROTEIN
RSc0720	-3,07	0,00	1,45	0,03		TRANSMEMBRANE TYPE 4 FIMBRIAL BIOGENESIS PROTEIN PILW-RELATED PROTEIN
RSc0766			4,32	0,01	cfa2	CYCLOPROPANE-FATTY-ACYL-PHOSPHOLIPID SYNTHASE
RSc0812			2,45	0,00	sorB	OXIDOREDUCTASE SULFITE:CYTOCHROME C OXIDOREDUCTASE (SUBUNIT A)
RSc0931			2,27	0,00		SIGNAL PEPTIDE
RSc0957	-2,37	0,03				HYPOTHETICAL PROTEIN
RSc1027			2,60	0,00		TRANSMEMBRANE
RSc1197			3,06	0,00		OXIDOREDUCTASE COG1804
RSc1496			4,01	0,02		HYPOTHETICAL PROTEIN
RSc1622			2,33	0,00		TRANSMEMBRANE
RSc1650			2,13	0,00		SIGNAL PEPTIDE
RSc1651			2,14	0,00		TWIN-ARGININE TRANSLOCATION PATHWAY SIGNAL SEQUENCE DOMAIN PROTEIN
RSc1676			2,81	0,00		HYPOTHETICAL PROTEIN
RSc1725			4,00	0,00		HYPOTHETICAL PROTEIN
RSc1726			3,96	0,00		CONSERVED HYPOTHETICAL PROTEIN
RSc1727			3,81	0,00		CONSERVED HYPOTHETICAL PROTEIN
RSc1733			3,44	0,00		SPERMIDINE/PUTRESCINE-BINDING PERIPLASMIC PROTEIN
RSc1756	-3,09	0,00	3,10	0,00	pehB	SIGNAL PEPTIDE EXO-POLY-GALACTURONOSIDASE
RSc1910			2,01	0,00		TRANSMEMBRANE
RSc1926	0,71	0,01	2,13	0,00		TRANSMEMBRANE
RSc1927			1,85	0,00		HYPOTHETICAL PROTEIN
RSc1969			2,08	0,00		TRANSMEMBRANE
RSc2024	-2,10	0,01				TRANSMEMBRANE COG1279
RSc2107			3,02	0,00	rsl	FUCOSE-BINDING LECTIN
RSc2131			3,08	0,00		HYPOTHETICAL PROTEIN
RSc2138			3,26	0,00		TRANSCRIPTION REGULATOR COG1733
RSc2281			2,00	0,03		TRANSMEMBRANE
RSc2282	-1,64	0,04	2,16	0,00		HYPOTHETICAL PROTEIN
RSc2283			1,98	0,00		HYPOTHETICAL PROTEIN
RSc2284	0,49	0,05	2,61	0,12		TRANSMEMBRANE COG3713
RSc2285	-3,43	0,00	3,22	0,00		Agrobacterium VIRK-LIKE PROTEIN
RSc2286	-3,06	0,00	3,58	0,00		CYCLOPROPANE FATTY ACID SYNTHASE
RSc2328	-2,78	0,00				SIGNAL PEPTIDE COG0596
RSc2566			2,27	0,00		METHYLATED-DNA--PROTEIN-CYSTEINE METHYLTRANSFERASE
RSc2858			2,80	0,00		ZINC PROTEASE
RSc2885					trpC1	INDOLE-3-GLYCEROL PHOSPHATE SYNTHASE
RSc2906			2,10	0,00		TRANSMEMBRANE
RSc2910			2,50	0,00		SIGNAL PEPTIDE
RSc3072			2,35	0,00		CONSERVED HYPOTHETICAL PROTEIN
RSc3101	-5,08	0,00	3,43	0,00		SERINE PROTEASE
RSc3288			4,31	0,00		MANNOSE/FUCOSE-BINDING LECTIN
RSc3296			2,20	0,00	sdaA2	L-SERINE AMONYA-LYASE, L-SERINE DEHYDRATASE 2 (L-SERINE DEAMINASE)
RSc3315			2,74	0,00		TRANSMEMBRANE
RSp0002	2,47	0,00	3,41	0,00	cspD3	TRANSCRIPTION REGULATOR COLD SHOCK-LIKE PROTEIN
RSp0071			2,25	0,00		CONSERVED HYPOTHETICAL PROTEIN
RSp0072	-2,60	0,02	2,61	0,00		CONSERVED HYPOTHETICAL PROTEIN
RSp0101			3,14	0,00		CONSERVED HYPOTHETICAL PROTEIN
RSp0161			4,17	0,00		TRANSMEMBRANE
RSp0162			4,20	0,00	egl	ENDOGLUCANASE PRECURSOR (ENDO-1,4-BETA-GLUCANASE)
RSp0198	-2,41	0,00	1,89	0,00		TRANSMEMBRANE
RSp0199						TRANSMEMBRANE
RSp0200			3,23	0,00		ORNITHINE/ACETYLORNITHINE AMINOTRANSFERASE
RSp0201			5,17	0,00		CONSERVED HYPOTHETICAL PROTEIN
RSp0275			4,76	0,00		TRANSMEMBRANE
RSp0294			2,62	0,01		HEMOLYSIN-TYPE CALCIUM-BINDING PROTEIN
RSp0338			2,37	0,00	epsR	TRANSCRIPTION REGULATOR NEGATIVE REGULATOR OF EXOPOLYSACCHARIDE PRODUCTION
RSp0405			2,68	0,00		HYPOTHETICAL PROTEIN
RSp0406			3,32	0,00		HYPOTHETICAL PROTEIN
RSp0466	-3,90	0,01				HYPOTHETICAL PROTEIN
RSp0473			2,10	0,01		TRANSMEMBRANE
RSp0487			3,08	0,00		ACETYLORNITHINE DEACETYLASE
RSp0506			2,14	0,00		TRANSMEMBRANE GLYCIN-RICH PROTEIN
RSp0535			3,37	0,01		TRANSCRIPTION REGULATOR LysR Family
RSp0536	-1,43	0,01	1,53	0,00		TRANSMEMBRANE SUGAR-PROTON SYMPORTER
RSp0596			2,77	0,00		TRANSMEMBRANE
RSp0603	-2,65	0,00	3,13	0,00		SERINE PROTEASE
RSp0676	-2,02	0,00	3,92	0,00	metE	5-METHYLTETRAHYDROPTEROYLTRIGLUTAMATE--HOMOCYSTEINE METHYLTRANSFERASE
RSp0678	3,80	0,00	2,65	0,00	pdxH2	OXIDOREDUCTASE PYRIDOXAMINE 5'-PHOSPHATE OXIDASE (PNP/PMP OXIDASE)
RSp0679	4,24	0,00	2,12	0,00		DIAMINOPIMELATE DECARBOXYLASE
RSp0680	3,26	0,00	2,18	0,00	trpC2	INDOLE-3-GLYCEROL PHOSPHATE SYNTHASE
RSp0681	1,47	0,00	1,20	0,00	trpD2	ANTHRANILATE PHOSPHORIBOSYLTRANSFERASE (GLYCOSYLTRANSFERASE)
RSp0682	4,94	0,00	2,72	0,00		OXIDOREDUCTASE TRIMETHYLAMINE DEHYDROGENASE
RSp0683	4,26	0,00	2,58	0,00		COG0325, Predicted enzyme with a TIM-barrel fold
RSp0684 RS	4,35	0,00	2,69	0,00		CONSERVED HYPOTHETICAL PROTEIN
RSp0685	3,93	0,00	3,83	0,00		TRANSMEMBRANE
RSp0881						TRANSMEMBRANE GLYCOSYL HYDROLASE
RSp0883	-4,56	0,02	3,11	0,00		CHAPERONIN GroEL-RELATED PROTEIN
RSp0898	-3,53	0,02			dsbA2	SIGNAL PEPTIDE THIOL:DISULFIDE INTERCHANGE PROTEIN
RSp0906	-0,55	0,03	2,54	0,00		SIGNAL PEPTIDE
RSp0957			1,20	0,00		HYPOTHETICAL PROTEIN
RSp0983			2,38	0,02		TRANSCRIPTION REGULATOR LuxR family
RSp1112	-2,47	0,03				TRANSMEMBRANE TRANSMEMBRANE MULTIDRUG EFFLUX SYSTEM PROTEIN
RSp1124	-2,66	0,00	2,61	0,00		OXIDOREDUCTASE
RSp1125	-3,26	0,00	1,96	0,02		HYPOTHETICAL PROTEIN
RSp1260			5,10	0,00		HYPOTHETICAL PROTEIN
RSp1364	-4,71	0,02			sucA	OXIDOREDUCTASE 2-OXOGLUTARATE DEHYDROGENASE E1 DECARBOXYLASE COMPONENT
RSp1417	-1,10	0,00	2,21	0,00		PERMEASE
RSp1418	-3,49	0,00	3,02	0,00		HYDROLASE
RSp1419	-3,09	0,00	2,80	0,00		PEPTIDE SYNTHASE
RSp1425			1,77	0,00	pvdA	OXIDOREDUCTASE L-ORNITHINE 5-MONOOXYGENASE
RSp1428			2,99	0,02		COG0607, Rhodanese-related sulfurtransferase
RSp1447			4,92	0,00		ABC TRANSPORTER

RSp1461	-3,58	0,00	3,63	0,00		SIGNAL PEPTIDE
RSp1462			3,96	0,00		OUTER-MEMBRANE EFFLUX PROTEIN
RSp1463			3,88	0,00		ABC TRANSPORTER HlyD family secretion protein
RSp1464			3,23	0,00		ABC TRANSPORTER ABC-type transport system, ATPase component
RSp1466			4,56	0,00		SIGNAL PEPTIDE
RSp1467			2,99	0,00		SIGNAL PEPTIDE
RSp1529	-1,23	0,01	4,00	0,00		ETHYLENE-FORMING-ENZYME (EC 1.14.11.-)
RSp1581	-1,97	0,00	2,67	0,00	katE	OXIDOREDUCTASE CATALASE hydroperoxidase HPII
RSp1604			1,49	0,00		TRANSMEMBRANE
RSp1610	-4,43	0,00	2,68	0,00		HYPOTHETICAL PROTEIN
RSp1670			2,43	0,00		SIGNAL PEPTIDE

HrpG-repressed independently from HrpB

RSc0018	-0,28	0,42	-2,06	0,00		TRANSCRIPTION REGULATOR RESPONSE REGULATOR
RSc0380					nasE	ABC TRANSPORTER NITRATE TRANSMEMBRANE PROTEIN
RSc0392						SIGNAL PEPTIDE PROLIN-RICH PROTEIN
RSc0416			-3,07	0,00		COG0693, Intracellular protease/amidase
RSc0599						TRANSCRIPTION REGULATOR (GNTR FAMILY)
RSc0823						HYPOTHETICAL PROTEIN
RSc0831					secA2	PREPROTEIN TRANSLOCASE SUBUNIT SecA (ATPase, RNA helicase) - RELATED PROTEIN
RSc0921			-2,06	0,00		ABC TRANSPORTER TRANSMEMBRANE PROTEIN
RSc0922			-3,54	0,00		ABC TRANSPORTER COG1464, ABC-type metal ion transport system, periplasmic component/surface antigen
RSc0962			-3,16	0,00		TRANSCRIPTION REGULATOR BACTERIOPHAGE DNA-BINDING PROTEIN
RSc1013						RIBOKINASE
RSc1023	0,25	0,89	-2,07	0,00	hscB	CHAPERONE PROTEIN
RSc1024	0,19	0,81	-2,22	0,00	hscA	CHAPERONE PROTEIN
RSc1025	0,42	0,53	-2,04	0,00	fdx	FERREDOXIN [2FE-2S]
RSc1026			-2,09	0,00		CONSERVED HYPOTHETICAL PROTEIN
RSc1040	0,10	0,74	-0,71	0,00	rne	RIBONUCLEASE E (RNASE E)
RSc1171	-0,03	0,65	-2,51	0,00	lysC	ASPARTATE KINASE
RSc1287	-0,25	0,61	-2,00	0,05		CONSERVED HYPOTHETICAL PROTEIN
RSc1341	0,14	0,98	-2,21	0,01	ssuB	ABC TRANSPORTER ALIPHATIC SULFONATES ATP-BINDING PROTEIN
RSc1673			-2,93	0,00		HYPOTHETICAL PROTEIN
RSc1743						HYPOTHETICAL PROTEIN
RSc1863	5,13	0,00				CONSERVED HYPOTHETICAL PROTEIN
RSc2050			-2,98	0,04	nuoM	OXIDOREDUCTASE TRANSMEMBRANE NADH DEHYDROGENASE I (CHAIN M)
RSc2068	0,15	0,54	-2,48	0,00		SIGNAL PEPTIDE
RSc2678	0,32	0,18	-2,75	0,01		TRANSMEMBRANE TYPE 4 FIMBRIAL BIOGENESIS PILV-RELATED PROTEIN
RSc2680			-2,22	0,00	pilE2	TRANSMEMBRANE TYPE 4 FIMBRIAL BIOGENESIS PROTEIN
RSc2731			-2,32	0,00	panD	ASPARTATE 1-DECARBOXYLASE PRECURSOR
RSc2861					fruB	MULTIPHOSPHORYL TRANSFER PROTEIN MTP
RSc2919			-3,16	0,01		TRANSMEMBRANE IRON TRANSPORT - SENSORY TRANSDUCTION PROTEIN
RSc2955	0,26	0,62	-2,36	0,02		ABC TRANSPORTER COG0842, ABC-type multidrug transport system, permease component
RSc3027	-0,20	0,40	-2,35	0,04		SIGNAL PEPTIDE
RSc3067	0,07	0,65	-2,05	0,00		TRANSMEMBRANE
RSc3168	3,16	0,00	-0,43	0,03	SRso1	ISRso13-TRANSPOSASE
RSc3179	2,22	0,00				TRANSPOSASE
RSc3386			-1,74	0,01		ABC TRANSPORTER COG1464, ABC-type metal ion transport system, periplasmic component/surface antigen
RSc3420						HYPOTHETICAL PROTEIN
RSp0005			-2,11	0,00		CONSERVED HYPOTHETICAL PROTEIN
RSp0025			-1,40	0,01		HYPOTHETICAL PROTEIN
RSp0147						HYPOTHETICAL PROTEIN
RSp0150						TRANSMEMBRANE GENERAL SECRETION PATHWAY GSPG-RELATED PROTEIN
RSp0175			-2,12	0,00		VGR-RELATED PROTEIN
RSp0176						CONSERVED HYPOTHETICAL PROTEIN
RSp0178			-2,74	0,00		HYPOTHETICAL PROTEIN
RSp0244	-0,86	0,05	-3,14	0,01		SIGNAL PEPTIDE OUTER MEMBRANE HEMIN RECEPTOR
RSp0285						TRANSCRIPTION REGULATOR (LUXR FAMILY)
RSp0332	-0,28	0,11	-2,35	0,00	acnB	TRANSMEMBRANE ACONITATE HYDRATASE 2
RSp0456			-2,28	0,03		SIGNAL PEPTIDE
RSp0513						TRANSMEMBRANE
RSp0545	-0,10	0,59	-2,17	0,00		CONSERVED HYPOTHETICAL PROTEIN
RSp0630	0,33	0,22	-3,27	0,01		PHOSPHOLIPASE
RSp0736	0,39	0,91	-2,39	0,02		TRANSMEMBRANE
RSp0741			-1,40	0,00		TRANSMEMBRANE
RSp0747			-2,23	0,00		CONSERVED HYPOTHETICAL PROTEIN
RSp0749			-2,31	0,00		CLPA/B-TYPE CHAPERONE
RSp0753			-2,31	0,00		TRANSMEMBRANE
RSp0754						HYPOTHETICAL PROTEIN
RSp0756			-2,99	0,00		HYPOTHETICAL PROTEIN
RSp0773			-2,57	0,00		SIGNAL PEPTIDE
RSp1098			-2,13	0,01		TRANSCRIPTION REGULATOR HNS-LIKE PROTEIN
RSp1108	3,10	0,02				SIGNAL PEPTIDE LIPASE
RSp1147 RS	-0,58	0,55	-2,32	0,00		OXIDOREDUCTASE COG2085, Predicted dinucleotide-binding enzymes
RSp1254					pseudoge	HIPA PROTEIN RELATED(FRAGMENT)
RSp1317						TRANSCRIPTION REGULATOR (LRP/ASNC FAMILY)
RSp1322	-0,14	0,41	-2,14	0,00		COG0251, Translation initiation inhibitor
RSp1323	0,16	0,58	-2,92	0,01		HYPOTHETICAL PROTEIN
RSp1329			-2,34	0,01		TRANSMEMBRANE
RSp1353			-2,72	0,00		COG0251, Translation initiation inhibitor
RSp1354	0,16	0,92	-2,09	0,01		HYPOTHETICAL PROTEIN
RSp1373					nosL	NOSL LIPOPROTEIN
RSp1413					flhD	TRANSCRIPTION REGULATOR FLAGELLAR TRANSCRIPTIONAL ACTIVATOR
RSp1550			-2,31	0,00		HYPOTHETICAL PROTEIN
RSp1642			-2,29	0,00		TRANSMEMBRANE
