# Supplementary material for: Integrated Regulation of the Type III Secretion System and Other Virulence Determinants in Ralstonia solanacearum
Source: PLoS Pathog. 2006 Aug 25;2(8):e82. doi: 10.1371/journal.ppat.0020082 (PMC1557829; doi:10.1371/journal.ppat.0020082)
Supplement: Table S2 — In all cases, RNA levels of the tested hrpB::Ω-pBBL12, GMI1000-pBBL12, or ΔhrpG-pL strains are expressed as Log2 ratios with respect to the GMI1000 strain bearing the empty vector pL. Results previously obtained in HrpB regulon analyses overexpressing or mutating this regulator (hrpB::Ω and GMI1000-pBBL12) [13] are also included. Experimental design, strain comparisons, and original hybridisation data are detailed under accession number E-MEXP-579 at the ArrayExpress depository (http://www.ebi.ac.uk/arrayexpress). (335 KB PDF) [file ppat.0020082.st002.pdf]

ID	AC	∆hrpG		hrpBΩ		WT pAM5		WTpBBL12		hrpBΩ pBBL1		Gene	Description	Class
RSc0001	RS06178					-0,30	0,04			-1,09	0,00	rpmH	50S RIBOSOMAL PROTEIN L34	III.C.2
RSc0002	RS01824					-0,57	0,01			-1,52	0,01	rnpA	RIBONUCLEASE P PROTEIN COMPONENT	II.A.2
RSc0003	RS01825			0,38	0,04	-0,38	0,05			-1,06	0,00		CONSERVED HYPOTHETICAL PROTEIN	VI.C
RSc0004	RS01826							0,89	0,00	-0,58	0,02	yidC	TRANSMEMBRANE COG0706, Preprotein translocase subunit YidC	IV.E.2
RSc0005	RS01827			0,46	0,02							thdF	THIOPHENE AND FURAN OXIDATION PROTEIN	IV.G.4
RSc0006	RS01828			0,73	0,03								COG4551, Predicted protein tyrosine phosphatase	VI.C
RSc0007	RS01829												HYPOTHETICAL PROTEIN	VI.B
RSc0008	RS01830					-0,33	0,01					rpsU	30S RIBOSOMAL SUBUNIT PROTEIN S21	III.C.2
RSc0009	RS01831												DRUG EFFLUX LIPOPROTEIN	IV.D
RSc0010	RS01832					-0,22	0,01	1,88	0,02			acrB	TRANSMEMBRANE ACRIFLAVIN RESISTANCE PROTEIN	IV.D
RSc0011	RS01833									0,69	0,04	acrA	ACRIFLAVIN RESISTANCE LIPOPROTEIN A PRECURSOR	IV.G.2
RSc0012	RS01834					-0,54	0,00	-0,91	0,02	1,24	0,00	acrR	TRANSCRIPTION REGULATOR ACRAB OPERON REPRESSOR	IV.G.2
RSc0013	RS01835					-0,44	0,00	1,43	0,01				CONSERVED HYPOTHETICAL PROTEIN	VI.C
RSc0014	RS01836			-0,90	0,00							gor	OXIDOREDUCTASE GLUTATHIONE REDUCTASE	I.B.6
RSc0015	RS01837	-0,51	0,01	0,63	0,02	-0,74	0,00						CONSERVED HYPOTHETICAL PROTEIN	VI.C
RSc0016	RS01838					-0,30	0,01					serA	OXIDOREDUCTASE D-3-PHOSPHOGLYCERATE DEHYDROGENASE	I.A.10
RSc0017	RS01839			1,06	0,00			1,46	0,00			pckA	PHOSPHOENOLPYRUVATE CARBOXYKINASE	I.F.13
RSc0018	RS01840					-0,99	0,00			-2,06	0,00		TRANSCRIPTION REGULATOR RESPONSE REGULATOR	VI.A
RSc0019	RS01841					-0,82	0,00	-1,37	0,00	-1,94	0,00		CONSERVED HYPOTHETICAL PROTEIN	VI.C
RSc0020	RS01842			1,25	0,00	-0,87	0,01						COG3622, Hydroxypyruvate isomerase	I
RSc0021	RS01843												TRANSCRIPTION REGULATOR TRANSMEMBRANE SENSOR HISTIDINE KINASE	VI.A
RSc0022	RS01844					-0,82	0,00	-1,01	0,01	-1,24	0,00	cstA	TRANSMEMBRANE CARBON STARVATION PROTEIN A	IV.F
RSc0023	RS01845					-1,06	0,00	-1,21	0,00				CONSERVED HYPOTHETICAL PROTEIN	VI.C
RSc0024	RS01846			1,34	0,00	-1,31	0,00	-0,85	0,01				SIGNAL PEPTIDE	VI.C
RSc0025	RS01848							0,81	0,00	0,72	0,04	phaP2	POLYHYDROXYBUTYRATE GRANULE-ASSOCIATED PROTEIN (PHASIN) PHAP2	II.B
RSc0026	RS01849			0,72	0,00							metW	METHIONINE BIOSYNTHESIS PROTEIN METW	I.A.12
RSc0027	RS01850											metX	HOMOSERINE O-ACETYLTRANSFERASE	I.A.12
RSc0028	RS01851					-0,72	0,05					gabD1	OXIDOREDUCTASE SUCCINATE-SEMIALDEHYDE DEHYDROGENASE	I.F.18
RSc0029	RS01852					-1,43	0,00			1,83	0,00	goaG	4-AMINOBUTYRATE AMINOTRANSFERASE	I.F.18
RSc0030	RS01853												TRANSCRIPTION REGULATOR	VI.A
RSc0031	RS01854					-0,64	0,00	-0,42	0,04				TRANSCRIPTION REGULATOR	VI.A
RSc0032	RS01855							-1,32	0,00				COG1011, Hydrolase	I.I
RSc0033	RS01856	-0,77	0,02	0,91	0,04			0,99	0,01			argB	ACETYLGLUTAMATE KINASE	I.A.17
RSc0034	RS01857							0,91	0,01				TRANSCRIPTION REGULATOR ECF-FAMILY RNA POLYMERASE SIGMA FACTOR	I.E.1
RSc0035	RS01858	0,34	0,02			-0,61	0,00			1,27	0,00		CONSERVED HYPOTHETICAL PROTEIN	VI.C
RSc0036	RS01859												SIGNAL PEPTIDE	VI.D
RSc0037	RS01860					-0,49	0,03			1,78	0,05		HYPOTHETICAL PROTEIN	VI.D
RSc0038	RS01861			0,47	0,01	-0,68	0,01			0,90	0,03		TRANSMEMBRANE	VI.C
RSc0039	RS01862												TRANSCRIPTION REGULATOR TRANSMEMBRANE SENSOR HISTIDINE KINASE	VI.A
RSc0040	RS01863							0,40	0,05				TRANSCRIPTION REGULATOR RESPONSE REGULATOR	VI.A
RSc0041	RS01864			-1,92	0,00	2,19	0,00	2,43	0,00				HYPOTHETICAL PROTEIN	VI.D
RSc0042	RS01865			1,12	0,03					-1,45	0,00	hslU	HEAT SHOCK PROTEIN CHAPERONE	IV.F.1
RSc0043	RS01866									-0,72	0,00	hslV	HEAT SHOCK PROTEIN	II.A.4
RSc0044	RS01867					-0,53	0,02						ABC TRANSPORTER SUBSTRATE-BINDING PERIPLASMIC PROTEIN (PBP)	IV.D
RSc0045	RS01868			1,01	0,02	-0,93	0,00						COG0523, GTPase	VI.C
RSc0046	RS01869											dksA	DNAK SUPPRESSOR PROTEIN	II.B.8
RSc0047	RS01870					-1,36	0,00						COG0523, GTPase	VI.C
RSc0048	RS01871					-0,94	0,00						TRANSCRIPTION REGULATOR	VI.A
RSc0049	RS01872					-0,41	0,00						3-CARBOXYMUCONATE CYCLASE	I
RSc0050	RS01873	-0,60	0,04			-1,01	0,00						OXIDOREDUCTASE COG0277, FAD/FMN-containing dehydrogenase	I
RSc0051	RS01874			1,47	0,01	-1,08	0,00						COG1092, Predicted SAM-dependent methyltransferase	I
RSc0052	RS01875			0,47	0,05	-0,41	0,00						SITE SPECIFIC INTEGRASE/RECOMBINASE	V.D
RSc0053	RS01876			0,81	0,00	-0,46	0,01	0,94	0,02				CONSERVED HYPOTHETICAL PROTEIN	VI.C
RSc0054	RS01877							0,53	0,03			gatB	GLU-TRNAGLN AMIDOTRANSFERASE SUBUNIT B	II.D.1
RSc0055	RS01878												SIGNAL PEPTIDE COG0412, Dienelactone hydrolase and related enzymes	I
RSc0056	RS01879					-0,51	0,04						LIPOPROTEIN	VI.C
RSc0057	RS01880											gatA	GLU-TRNA (GLN) AMIDOTRANSFERASE (SUBUNIT A)	II.D.1
RSc0058	RS01881					-0,27	0,05					gatC	GLUTAMYL-TRNA GLN AMIDOTRANSFERASE SUBUNIT C	III.C.2
RSc0059	RS01882							0,76	0,01			mreB	ROD SHAPE-DETERMINING PROTEIN	III.B.3
RSc0060	RS01883											mreC	TRANSMEMBRANE ROD SHAPE-DETERMINING PROTEIN MREC	III.B.3
RSc0061	RS01884			0,69	0,00							mreD	TRANSMEMBRANE ROD SHAPE-DETERMINING PROTEIN MRED	III.B.3
RSc0062	RS05767					-0,55	0,00	-1,14	0,03				TRANSMEMBRANE SUBSTRATE-BINDING PROTEIN	III.B.3
RSc0063	RS05768											mdrB	TRANSMEMBRANE ROD SHAPE-DETERMINING PROTEIN (RODA PROTEIN)	IV.A
RSc0064	RS05769												LONG-CHAIN-FATTY-ACID--CoA LIGASE	I.I.1
RSc0065	RS02244					-0,63	0,00						TRANSCRIPTION REGULATOR	VI.A
RSc0066	RS02245			0,98	0,01	-0,61	0,04	0,81	0,00			topB	DNA TOPOISOMERASE III	II.B.8
RSc0067	RS02246			1,05	0,05	-0,67	0,00						THIOREDOXIN	II.B.7
RSc0068	RS02247							-0,75	0,03			smf	SMF PROTEIN (COG0758 Predicted Rossmann fold nucleotide-binding protein involv	VI.C
RSc0069	RS02271			0,53	0,05					-0,39	0,02		SIGNAL PEPTIDE	VI.C
RSc0070	RS02248			0,34	0,01	-0,40	0,04	-0,52	0,04	-0,58	0,04	def	POLYPEPTIDE DEFORMYLASE	II.B.7
RSc0071	RS02249			1,06	0,01	-0,40	0,00						TRANSMEMBRANE	VI.C
RSc0072	RS02250			0,62	0,00							fmt	METHIONYL-TRNA FORMYLTRANSFERASE	II.D.1
RSc0073	RS02251			-1,26	0,00	1,87	0,00	1,91	0,00				TRANSMEMBRANE TRANSPORTER	IV.D
RSc0074	RS02252							-0,71	0,01			htpX	TRANSMEMBRANE METALLOPROTEASE ZINC	II.A.4
RSc0075	RS02273	-0,82	0,02			-0,30	0,01					rrmB	RNA METHYLTRANSFERASE (SUN PROTEIN)	II.B.6
RSc0076	RS02253			0,44	0,05	-0,61	0,00			-0,04	0,04		SIGNAL PEPTIDE PROLINE RICH PROTEIN	VI.C
RSc0077	RS02275					-0,75	0,02						TRANSCRIPTION REGULATOR TWO COMPONENT SENSOR HISTIDINE KINASE	VI.A
RSc0078	RS02276					-0,51	0,00						TRANSCRIPTION REGULATOR TWO COMPONENT RESPONSE REGULATOR	VI.A
RSc0079	RS02277					-0,42	0,01						TRANSMEMBRANE	VI.D
RSc0080	RS02254					-0,32	0,02	-0,24	0,05				TRANSMEMBRANE	VI.D
RSc0081	RS02255							-1,35	0,03				TRANSCRIPTION REGULATOR COG1737, Transcriptional regulator	VI.A
RSc0082	RS02256												COG0161, Adenosylmethionine-8-amino-7-oxononanoate aminotransferase	I
RSc0083	RS02257					0,15	0,05						CONSERVED HYPOTHETICAL PROTEIN	VI.C
RSc0084	RS02258												ABC TRANSPORTER AMINO-ACID-BINDING PERIPLASMIC PROTEIN (PBP)	IV.D.2
RSc0085	RS02259			0,32	0,03	-0,31	0,05					cca	tRNA NUCLEOTIDYLTRANSFERASE	II.D.1
RSc0086	RS02260					-0,25	0,05					gstA	GLUTATHIONE S-TRANSFERASE	II.B.7
RSc0087	RS02261			0,45	0,05								OXIDOREDUCTASE NADH-UBIQUINONE OXIDOREDUCTASE	I.H
RSc0088	RS02278											slt	TRANSMEMBRANE SOLUBLE LYTIC MUREIN TRANSGLYCOSYLASE PRECURSO	III.B.3
RSc0089	RS02262					-0,43	0,03						COG0212, 5-formyltetrahydrofolate cyclo-ligase	I
RSc0090	RS02263					0,82	0,00			-0,88	0,01		TRANSCRIPTION REGULATOR COG3070, Regulator of competence-specific genes	VI.A
RSc0091	RS02264					-0,45	0,02			-0,80	0,01	metF	OXIDOREDUCTASE 5,10-METHYLENETETRAHYDROFOLATE REDUCTASE	I.A.12
RSc0092	RS02279									-0,69	0,05		TRANSMEMBRANE	VI.C
RSc0093	RS02265					-0,63	0,00			-1,52	0,00	ahcY	ADENOSYLHOMOCYSTEINASE (S-ADENOSYL-L-HOMOCYSTEINE HYDROLASE	I.F
RSc0094	RS02282			0,48	0,03	-0,40	0,05						TRANSMEMBRANE LIPOPROTEIN	VI.D
RSc0095	RS02285												CONSERVED HYPOTHETICAL PROTEIN	VI.C
RSc0096	RS06003			-2,44	0,00	3,20	0,00	1,32	0,00				HYPOTHETICAL PROTEIN	VI.D
RSc0097	RS02267			1,02	0,05								TRANSMEMBRANE ALKALINE PHOSPHATASE LIPOPROTEIN	I.F.20
RSc0098	RS02280					-0,60	0,03						SIGNAL PEPTIDE ALKALINE PHOSPHATASE	I.F.20
RSc0099	RS02268												SIGNAL PEPTIDE	VI.B
RSc0100	RS02611					-0,33	0,04					thiD	BIFUNCTIONAL PROTEIN: HYDROXY-PHOSPHOMETHYLPYRIMIDINE KINASE A	I.B.8
RSc0101	RS02270												TRANSMEMBRANE LIPOPROTEIN	VI.C
RSc0102	RS05630			-1,54	0,00	1,62	0,01	-1,01	0,01				CALCIUM BINDING HEMOLYSIN	IV.G.3
RSc0104	RS00975	-0,75	0,01			-0,55	0,05						HEMOLYSIN-TYPE CALCIUM-BINDING PROTEIN	IV.G.3
RSc0105	RS01614												HYPOTHETICAL PROTEIN	VI.D
RSc0106	RS01696			0,38	0,03								SIGNAL PEPTIDE	VI.D
RSc0107	RS00977					-0,61	0,01			2,08	0,00		CONSERVED HYPOTHETICAL PROTEIN	VI.C
RSc0108	RS00978											thiE1	THIAMINE-PHOSPHATE PYROPHOSPHORYLASE	I.B.8
RSc0109	RS00979					-0,35	0,03	-1,03	0,01			thiG	THIG PROTEIN THIAMINE BIOSYNTHESIS	I.B.8
RSc0111	RS00981					-0,36	0,04					thiS	Sulfur transfer protein ThiS	I.B.8
RSc0112	RS00982							-1,16	0,05	-0,60	0,02		OXIDOREDUCTASE D-AMINO ACID OXIDASE FLAVOPROTEIN	I

RSc0113	RS00983			-0,56 0,00		-1,60 0,00	thiC	THIAMINE BIOSYNTHESIS PROTEIN THIC THIAMINE BIOSYNTHESIS	I.B.8
RSc0114	RS00984							OXIDOREDUCTASE DIOXYGENASE related to 2-nitropropane dioxygenase	I
RSc0115	RS00985		0,79 0,00	-1,00 0,00	1,83 0,02			HEMAGGLUTININ-RELATED AUTOTRANSPORTER PROTEIN	III.A
RSc0116	RS00986	0,50 0,01		-0,52 0,04		1,36 0,00		CONSERVED HYPOTHETICAL PROTEIN	VI.C
RSc0117	RS00987			-0,71 0,00		2,16 0,00		CONSERVED HYPOTHETICAL PROTEIN	VI.C
RSc0118	RS00988			-0,59 0,00	1,22 0,00	2,42 0,00		CONSERVED HYPOTHETICAL PROTEIN	VI.C
RSc0119	RS00989							HYPOTHETICAL/UNKNOWN	VI.C
RSc0120	RS00990			-0,42 0,04				ACETYLTRANSFERASE	VI.C
RSc0121	RS00991		0,42 0,01	-0,52 0,00				OXIDOREDUCTASE ACYL-COA DEHYDROGENASE	I
RSc0122	RS00992		0,32 0,04	-0,67 0,00		-1,25 0,00		ABC TRANSPORTER SUBSTRATE-BINDING PERIPLASMIC PROTEIN (PBP)	IV.D
RSc0123	RS00993			-0,44 0,02				OXIDOREDUCTASE COG2303, Choline dehydrogenase and related flavoproteins	I
RSc0124	RS00994							CONSERVED HYPOTHETICAL PROTEIN	VI.C
RSc0125	RS00995		-1,40 0,05					TRANSCRIPTION REGULATOR COG2944, Transcriptional regulator	VI.A
RSc0126	RS00996		-0,86 0,05	0,96 0,01				HYPOTHETICAL/UNKNOWN	VI.D
RSc0127	RS00997			-0,20 0,03				HEMAGGLUTININ-RELATED PROTEIN	VI.C
RSc0128	RS00998			-0,56 0,01				TRANSCRIPTION REGULATOR	VI.A
RSc0129	RS00999						gstN	GLUTATHIONE S-TRANSFERASE	II.B.7
RSc0130	RS01000							TRANSMEMBRANE COG3448, CBS-domain-containing membrane protein	VI.A
RSc0131	RS01001							LIPOPROTEIN	VI.C
RSc0132	RS01002			-0,49 0,00				TRANSCRIPTION REGULATOR	VI.A
RSc0133	RS01003							CONSERVED HYPOTHETICAL PROTEIN	VI.C
RSc0134	RS01004			-0,46 0,00	-0,64 0,00		metK	S-ADENOSYLMETHIONINE SYNTHETASE	I.F
RSc0135	RS01005		0,57 0,02					TRANSMEMBRANE ACYLTRANSFERASE	II.B
RSc0136	RS01006							ACYLTRANSFERASE	II.B
RSc0137	RS01049		0,65 0,04	-0,39 0,04			dapF	DIAMINOPIMELATE EPIMERASE	I.A.13
RSc0138	RS01008							CONSERVED HYPOTHETICAL PROTEIN	VI.C
RSc0139	RS01009						pyrE	OROTATE PHOSPHORIBOSYLTRANSFERASE	I.D.1
RSc0140	RS01010			-0,29 0,02				EXODEOXYRIBONUCLEASE III	II.A.1
RSc0141	RS01011			-0,44 0,03				TRANSMEMBRANE	VI.C
RSc0142	RS01012			-0,60 0,00			argC	OXIDOREDUCTASE N-ACETYL-GAMMA-GLUTAMYL-PHOSPHATE REDUCTASE	I.A.17
RSc0143	RS01013			-0,70 0,03				CONSERVED HYPOTHETICAL PROTEIN	VI.C
RSc0144	RS01014			-0,58 0,05				TRANSCRIPTION REGULATOR	VI.A
RSc0145	RS01015							TRANSMEMBRANE COG0330, Membrane protease subunit, stomatin/prohibitin homc	II.A.4
RSc0146	RS01016		0,63 0,00	-0,56 0,00				TRANSMEMBRANE LIPOPROTEIN	VI.C
RSc0147	RS01017			-0,75 0,01				SIGNAL PEPTIDE HYPOTHETICAL	VI.B
RSc0148	RS01018							HYPOTHETICAL/UNKNOWN	VI.D
RSc0149	RS01019			-0,57 0,01				TRANSCRIPTION REGULATOR	VI.A
RSc0150	RS01020			-0,74 0,02	-0,44 0,03			CONSERVED HYPOTHETICAL PROTEIN	VI.C
RSc0151	RS01021			-1,73 0,02	-1,63 0,00			TRANSMEMBRANE	VI.C
RSc0152	RS01022			-0,68 0,00				LIPOPROTEIN	VI.D
RSc0153	RS01023							SIGNAL PEPTIDE COG0501, Zn-dependent protease with chaperone function	II.A.4
RSc0154	RS01024						ampG	TRANSMEMBRANE TRANSPORT protein	IV.D
RSc0155	RS01025							CONSERVED HYPOTHETICAL PROTEIN	VI.C
RSc0156	RS01026		0,40 0,03	-0,27 0,02		-0,46 0,01		OXIDOREDUCTASE COG1028, Dehydrogenases with different specificities (related tc	I
RSc0157	RS01027							HYPOTHETICAL PROTEIN	I.F
RSc0158	RS01028			-1,24 0,00		2,17 0,00		ARGINASE	I.I.3
RSc0159	RS01029			-1,33 0,00		0,69 0,00		ACETYLORNITHINE AMINOTRANSFERASE	I.I.3
RSc0160	RS01030			0,81 0,01				TRANSCRIPTION REGULATOR	VI.A
RSc0161	RS01031							OXIDOREDUCTASE TRANSMEMBRANE ALDEHYDE DEHYDROGENASE	I
RSc0162	RS01032		-1,59 0,00	0,91 0,00	-1,71 0,00	-0,35 0,02		TRANSCRIPTION REGULATOR	VI.A
RSc0163	RS01033			-1,64 0,00	-1,17 0,00			CONSERVED HYPOTHETICAL PROTEIN	VI.C
RSc0164	RS01034			-0,87 0,00				ABC TRANSPORTER ATP-BINDING PROTEIN	IV.D
RSc0165	RS01035		0,58 0,04	-0,44 0,00				ABC TRANSPORTER ABC-type multidrug transport system, permease component	IV.D
RSc0166	RS01036							Outer membrane channel lipoprotein	IV.D
RSc0167	RS01037		0,59 0,01	-0,88 0,00				SIGNAL PEPTIDE	VI.C
RSc0168	RS01038		1,19 0,01	-0,51 0,00				CONSERVED HYPOTHETICAL PROTEIN	VI.C
RSc0169	RS01039		1,13 0,01					COG0451, Nucleoside-diphosphate-sugar epimerase	I
RSc0170	RS01040		0,53 0,03					TRANSMEMBRANE	VI.C
RSc0171	RS01042							OXIDOREDUCTASE COG0300, Short-chain dehydrogenases of various substrate spe	I
RSc0172	RS01043		1,97 0,04					TRANSMEMBRANE COG0277, FAD/FMN-containing dehydrogenase	I
RSc0173	RS01044		0,73 0,02	-0,24 0,04				TRANSMEMBRANE PROTEIN WITH COG0382, 4-hydroxybenzoate polyprenyltransfe	I
RSc0174	RS01045			-0,62 0,02				OXIDOREDUCTASE COG1893, Ketopantoate reductase	I
RSc0175	RS01046			-0,20 0,01				COG0037, Predicted ATPase of the PP-loop superfamily implicated in cell cycle contr	IV.A
RSc0176	RS01047		-0,55 0,01					LIPOPROTEIN	VI.C
RSc0177	RS01048		0,58 0,00		0,98 0,02	-0,24 0,01	glmU	UDP-N-ACETYLGLUCOSAMINE PYROPHOSPHORYLASE	I.F.10
RSc0178	RS00609		0,53 0,00				glmS	GLUCOSAMINE--FRUCTOSE-6-PHOSPHATE AMINOTRANSFERASE	I.F.8
RSc0179	RS00610							COG0589, Universal stress protein UspA and related nucleotide-binding proteins	VI.C
RSc0180	RS00611							PSEUDOGENE (ALCOHOL DEHYDROGENASE C-TERMINAL FRAGMENT)	VI.C
RSc0181	RS00612							PSEUDOGENE (ALCOHOL DEHYDROGENASE N-TERMINAL FRAGMENT)	VI.C
RSc0182	RS00613			1,38 0,00	1,90 0,01			TRANSMEMBRANE	VI.C
RSc0183	RS00614							Signal-transduction protein containing a CBS domain	VI.A
RSc0184	RS00615							COG0589, Universal stress protein UspA and related nucleotide-binding proteins	VI.C
RSc0185	RS00616			-0,24 0,05				Universal stress protein UspA and related nucleotide-binding proteins	VI.C
RSc0186	RS00617							COG2823, Periplasmic or secreted lipoprotein	VI.C
RSc0187	RS00618							COG0716, Flavodoxin	I
RSc0188	RS00619							HYPOTHETICAL PROTEIN	VI.B
RSc0189	RS00621			-1,12 0,05				COG2823, Periplasmic or secreted lipoprotein	VI.C
RSc0190	RS00622							TRANSCRIPTION REGULATOR	VI.A
RSc0191	RS00623							TRANSCRIPTION REGULATOR TRANSMEMBRANE TWO COMPONENT SYSTEM	VI.A
RSc0192	RS00624							TRANSCRIPTION REGULATOR RESPONSE REGULATOR	VI.A
RSc0193	RS00625							HYPOTHETICAL PROTEIN	VI.D
RSc0194	RS00626							OXIDOREDUCTASE ZINC-DEPENDENT ALCOHOL DEHYDROGENASE	I
RSc0195	RS00627							OXIDOREDUCTASE RIBONUCLEOTIDE REDUCTASE, alpha subunit	I
RSc0196	RS00628		-1,14 0,02	-0,49 0,02				SIGNAL PEPTIDE CYTOCHROME C1	I.G.1
RSc0197	RS00629			-0,85 0,03				SIGNAL PEPTIDE CYTOCHROME C	I.G.1
RSc0198	RS00630			-0,77 0,01				TRANSMEMBRANE	VI.C
RSc0199	RS00631							TRANSMEMBRANE CYTOCHROME B561 CYTOCHROME	I.G.1
RSc0200	RS00632		-2,35 0,05					SMALL HEAT SHOCK PROTEIN	IV.F.1
RSc0201	RS00633							COG1236, Exonuclease of the beta-lactamase fold involved in RNA processing	II.B.6
RSc0202	RS00634							RIBOSE-PHOSPHATE PYROPHOSPHOKINASE	I.D.2
RSc0203	RS00635							CONSERVED HYPOTHETICAL PROTEIN	VI.C
RSc0204	RS00636	-1,39 0,05					deoA	THYMIDINE/PYRIMIDINE-NUCLEOSIDE PHOSPHORYLASE	I.F.1
RSc0205	RS00637		0,40 0,05	-0,74 0,01				TRANSCRIPTION REGULATOR	VI.A
RSc0206	RS00638				-0,77 0,05			SIGNAL PEPTIDE COG1073, Hydrolase of the alpha/beta superfamily	I.I
RSc0207	RS00639							TRANSMEMBRANE TRANSPORTER	IV.D
RSc0209	RS00641							TRANSMEMBRANE	VI.D
RSc0210	RS00642							TRANSCRIPTION REGULATOR	VI.A
RSc0211	RS00643							TRANSMEMBRANE	VI.D
RSc0212	RS00644			-0,45 0,00	0,87 0,01			ASPARTATE RACEMASE	I.A.15
RSc0213	RS00645			-0,77 0,03				GLUTAMINE AMIDOTRANSFERASE	I.D.2
RSc0214	RS00646							TRANSCRIPTION REGULATOR	VI.A
RSc0215	RS00647	-1,72 0,01			3,01 0,00	2,19 0,01		OXIDOREDUCTASE COG1028, Dehydrogenases with different specificities (related tc	I
RSc0216	RS00648			-1,19 0,00	4,10 0,00			TRANSMEMBRANE TRANSPORT PROTEIN	IV.D
RSc0217	RS00649			-0,87 0,03	3,20 0,00	2,82 0,00		OXIDOREDUCTASE COG0667, Oxidoreductase (related to aryl-alcohol dehydrogena:	I
RSc0218	RS00650			-1,02 0,00				HYPOTHETICAL/UNKNOWN	VI.D
RSc0219	RS00651			-0,68 0,02				COG1254, ACYLPHOSPHATASE	I.H
RSc0220	RS00652							COG4101, Predicted mannose-6-phosphate isomerase	I.F.4
RSc0221	RS00653			-1,91 0,00	-1,83 0,01			TRANSMEMBRANE COG1752, Esterase of the alpha-beta hydrolase superfamily	I
RSc0222	RS00654						rtcR	TRANSCRIPTION REGULATOR SIGMA-54 INTERACTING PROTEIN	II.B.6
RSc0223	RS00655			-1,23 0,00	-1,32 0,00	-0,90 0,01		HYPOTHETICAL/UNKNOWN	VI.D
RSc0224	RS00656			-0,91 0,00	-0,37 0,00		rtcB	RTCB PROTEIN	VI.C
RSc0225	RS00657			-0,80 0,00				COG3541, Nucleotidyltransferase	I.D

RSc0226	RS00658						rtcA	RNA 3'-TERMINAL PHOSPHATE CYCLASE	II.B.6
RSc0227	RS06016		0,64 0,00	-0,45 0,00				REMNANT OF A AVRPMAA1/AVRPPIA1 GENE HOMOLOG	VI.B
RSc0228	RS00660							REMNANT OF A AVRPMAA1/AVRPPIA1 GENE HOMOLOG	VI.B
RSc0229	RS00661		0,42 0,00	-0,55 0,00				TRANSCRIPTION REGULATOR COG1167, Transcriptional regulators containing a DI	VI.A
RSc0230	RS00663			-0,58 0,04				TRANSMEMBRANE	VI.C
RSc0231	RS00662		-0,54 0,03		1,15 0,00			TRANSMEMBRANE (HOMOSERINE/HOMOSERINE LACTONE) EFFLUX PROTEIN	IV.D.2
RSc0232	RS00664	1,16 0,01		-1,13 0,00	1,89 0,00	4,53 0,00		HYPOTHETICAL PROTEIN	VI.B
RSc0233	RS00665							TRANSMEMBRANE INTEGRAL MEMBRANE PROTEIN	VI.C
RSc0234	RS00666			-0,90 0,00	-0,96 0,02			TRANSCRIPTION REGULATOR	VI.A
RSc0235	RS00667							CONSERVED HYPOTHETICAL PROTEIN	VI.C
RSc0236	RS00668		0,39 0,02	-0,65 0,02	1,53 0,01			SIGNAL PEPTIDE HYPOTHETICAL/UNKNOWN	VI.D
RSc0237	RS00669							METHYLMALONYL-CoA MUTASE	I
RSc0238	RS00670			-1,06 0,01	1,31 0,00			TRANSMEMBRANE PUTATIVE PERMEASE	IV.D
RSc0239	RS00671							COG1574, Predicted metal-dependent hydrolase with the TIM-barrel fold	I.I
RSc0240	RS00672							TRANSMEMBRANE	VI.C
RSc0241	RS00673		-0,71 0,03					TRANSMEMBRANE	VI.C
RSc0242	RS00674							COG1335, Amidase related to nicotinamidase	I.I
RSc0243	RS00675				-0,96 0,05			TRANSCRIPTION REGULATOR TRANSCRIPTIONAL REGULATOR	VI.A
RSc0244	RS00676		0,68 0,00	-0,77 0,00			paaH1	OXIDOREDUCTASE 3-HYDROXYBUTYRYL-COA DEHYDROGENASE	I.I
RSc0245	RS00677		-3,22 0,00	3,59 0,00	3,16 0,00		ripB	TYPE III EFFECTOR RIPB	VI.E
RSc0246	RS00678							CALCIUM BINDING HEMOLYSIN	IV.G.3
RSc0247	RS00679		0,65 0,04	-0,86 0,00	-0,96 0,01		tISRso6	ISRso6-TRANSPOSASE ORFA	V.B
RSc0248	RS00680		0,68 0,05	-0,29 0,00			tISRso6	ISRso6-TRANSPOSASE ORFB	V.B
RSc0249	RS00683			-0,38 0,04				CALCIUM BINDING HEMOLYSIN	IV.G.3
RSc0250	RS00684							CONSERVED HYPOTHETICAL PROTEIN	VI.C
RSc0251	RS00685		-1,29 0,02	1,16 0,00				SIGNAL PEPTIDE	VI.D
RSc0253	RS00688							COG0384 PREDICTED EPIMERASE (PHENAZINE BIOSYNTHESIS PROTEIN)	I
RSc0254	RS00689			-0,55 0,00	0,71 0,03			TRANSMEMBRANE	VI.C
RSc0255	RS00690			-0,65 0,00				COG2070, Dioxygenases related to 2-nitropropane dioxygenase	I
RSc0256	RS00691			-0,84 0,00			dehH	HALOACETATE DEHALOGENASE H-1	I.I.4
RSc0257	RS00692		-4,07 0,00	6,58 0,00	5,13 0,00			ANKYRIN REPEAT HARBORING PROTEIN	VI.B
RSc0258	RS00693							BETA LACTAMASE	IV.G.4
RSc0259	RS00694							COG3313, Fe-S protein	VI.C
RSc0260	RS00695							CONSERVED HYPOTHETICAL PROTEIN	VI.C
RSc0261	RS00696		0,53 0,05					CONSERVED HYPOTHETICAL PROTEIN	VI.C
RSc0262	RS00697		-0,53 0,01					OXIDOREDUCTASE COG0111, Phosphoglycerate dehydrogenase and related dehyd	I
RSc0263	RS00698							HYPOTHETICAL PROTEIN	VI.D
RSc0264	RS00699		0,32 0,03					HYPOTHETICAL PROTEIN	VI.B
RSc0265	RS05771							COG4770, Acetyl/propionyl-CoA carboxylase, alpha subunit	I
RSc0266	RS03233		0,56 0,05				bioB	BIOTIN SYNTHASE	I.B.15
RSc0267	RS03234			-0,72 0,03	-0,34 0,03			ENOYL-COA HYDRATASE	I.I.4
RSc0268	RS03235							SIGNAL PEPTIDE COG0412, Dienelactone hydrolase and related enzymes	I
RSc0269	RS03236		0,81 0,00	-0,34 0,03				COG4799, Acetyl-CoA carboxylase, carboxyltransferase component (subunits alpha a	I
RSc0270	RS03237			-0,26 0,01	0,88 0,04			CONSERVED HYPOTHETICAL PROTEIN	VI.C
RSc0271	RS03238		0,70 0,05					COG0637, Phosphatase/phosphohexomutase	I
RSc0272	RS03239							COG0607, Rhodanese-related sulfurtransferase	I
RSc0273	RS03240		0,95 0,04					2-HYDROXYCHROMENE-2-CARBOXYLATE ISOMERASE	I.I.4
RSc0274	RS03241	-1,05 0,01				0,76 0,00		OXIDOREDUCTASE ACYL CoA DEHYDROGENASE	I
RSc0275	RS03242		0,72 0,02					OXIDOREDUCTASE COG0300, Short-chain dehydrogenases of various substrate spe	I
RSc0276	RS03243							ACETYL-COA ACETYLTRANSFERASE (ACETOACETYL-COA THIOLASE)	I.I.1
RSc0277	RS03244		0,70 0,02					CARBONIC ANHYDRASE	I.F.18
RSc0278	RS03245			-0,26 0,00			aceK	TRANSMEMBRANE MULTIFUNCTIONAL ENZYME : ISOCITRATE DEHYDROGENA	I.F.17
RSc0279	RS03246							OXIDOREDUCTASE ACYL-COA DEHYDROGENASE	I.I.3
RSc0280	RS03247			-0,51 0,00				TRANSCRIPTION REGULATOR	VI.A
RSc0281	RS03248		1,00 0,01					COG0491, Zn-dependent hydrolases, including glyoxylases	I.I
RSc0282	RS03249							CONSERVED HYPOTHETICAL PROTEIN	VI.C
RSc0283	RS03250			-0,36 0,00				ABC TRANSPORTER COG0614, ABC-type Fe3+-hydroxamate transport system, peri	IV.D.5
RSc0284	RS03251			-0,41 0,00				OXIDOREDUCTASE COG0300, Short-chain dehydrogenases of various substrate spe	I
RSc0285	RS03252			-0,40 0,00			dsbA1	SIGNAL PEPTIDE THIOL:DISULFIDE INTERCHANGE PROTEIN	II.B.7
RSc0286	RS03253		0,60 0,04	-0,37 0,01	0,59 0,02		ftsN	TRANSMEMBRANE Cell division protein FtsN	IV.A
RSc0287	RS03254						argS	ARGINYL-TRNA SYNTHETASE (ARGININE--TRNA LIGASE)	II.D.1
RSc0288	RS03255			-0,42 0,04		0,61 0,00		CONSERVED HYPOTHETICAL PROTEIN	VI.C
RSc0289	RS03256			-0,70 0,01			vsrA	TRANSCRIPTION REGULATOR TRANSMEMBRANE SENSOR KINASE VSRA	I.E.1
RSc0290	RS03257		-0,31 0,01	-0,34 0,04				COG5510 : Predicted small secreted protein	III.A
RSc0291	RS03258			-0,51 0,00				TRANSCRIPTION REGULATOR	VI.A
RSc0292	RS03259			-0,52 0,00			vsrD	TRANSCRIPTION REGULATOR RESPONSE REGULATOR	I.E.1
RSc0293	RS03260			-0,45 0,01	1,13 0,00	2,70 0,00		SIGNAL PEPTIDE	VI.D
RSc0294	RS03261						metHb	5-METHYLTETRAHYDROFOLATE--HOMOCYSTEINE METHYLTRANSFERASE (MI	I.A.12
RSc0295	RS03262		0,41 0,01	-0,38 0,05			metHa	5-METHYLTETRAHYDROFOLATE--HOMOCYSTEINE METHYLTRANSFERASE (MI	I.A.12
RSc0296	RS03263			-0,66 0,01	-0,66 0,02	1,52 0,02		COG0607, Rhodanese-related sulfurtransferase	I
RSc0297	RS03264			-1,66 0,00	-1,94 0,00			CONSERVED HYPOTHETICAL PROTEIN	VI.C
RSc0298	RS03265		0,32 0,03		-1,11 0,05		pcaD2	B-KETOADIPATE ENOL-LACTONE HYDROLASE	I.I.4
RSc0299	RS03266		0,88 0,01	-0,27 0,05	0,91 0,04			SIGNAL PEPTIDE PROLIN-RICH PROTEIN	VI.C
RSc0300	RS03267			-0,35 0,00				TRANSCRIPTION REGULATOR	VI.A
RSc0301	RS03268		1,75 0,01	-0,34 0,02				OXIDOREDUCTASE COG0179, 2-keto-4-pentenoate hydratase/2-oxohepta-3-ene-1,7	I
RSc0302	RS03269			-1,17 0,00				TRANSCRIPTION REGULATOR	VI.A
RSc0303	RS03270			-1,52 0,00	-1,62 0,01			CONSERVED HYPOTHETICAL PROTEIN	VI.C
RSc0304	RS03271		0,65 0,02					ENOYL-COA HYDRATASE	I.I.4
RSc0305	RS03272			0,45 0,04	1,53 0,00			CONSERVED HYPOTHETICAL PROTEIN	VI.C
RSc0306	RS03273							COG1752, Esterase of the alpha-beta hydrolase superfamily	I
RSc0307	RS03274			-0,58 0,00				GLUTATHIONE PEROXIDASE	II.B.7
RSc0308	RS03275		0,43 0,01	-0,55 0,04				CONSERVED HYPOTHETICAL PROTEIN	VI.C
RSc0309	RS03276							CONSERVED HYPOTHETICAL PROTEIN	VI.C
RSc0310	RS03277							SIGNAL PEPTIDE	VI.C
RSc0311	RS03278			-0,39 0,01	-0,61 0,02			TRANSCRIPTION REGULATOR TRANSCRIPTION REGULATION ACCESSORY FA	VI.A
RSc0312	RS03279						birA	BIFUNCTIONAL PROTEIN: BIOTIN OPERON REPRESSOR AND BIOTIN--[ACETYL	I.B.15
RSc0313	RS03280		0,41 0,02			-0,48 0,04		ABC TRANSPORTER COG0767, ABC-type transport system involved in resistance to	IV.D
RSc0314	RS03281							ABC TRANSPORTER COG1127, ABC-type transport system involved in resistance to	IV.D
RSc0315	RS03282		0,59 0,01					ABC TRANSPORTER COG1463, ABC-type transport system involved in resistance to	IV.D
RSc0316	RS03283				-0,65 0,04			ABC TRANSPORTER COG3218, ABC-type uncharacterized transport system, auxiliar	IV.D
RSc0317	RS03284	0,47 0,02		-0,50 0,02				TRANSMEMBRANE	VI.C
RSc0318	RS03285				-0,75 0,03			HYPOTHETICAL PROTEIN	VI.D
RSc0319	RS03286						plcN	SIGNAL PEPTIDE NON-HEMOLYTIC PHOSPHOLIPASE C (PHOSPHATIDYLCHOLI	I.I.1
RSc0320	RS03287		1,15 0,02	-0,60 0,00	-1,80 0,00			SIGNAL PEPTIDE	VI.C
RSc0321	RS03288		-2,23 0,00	3,22 0,00	2,96 0,00			HYPOTHETICAL PROTEIN	VI.D
RSc0322	RS03289			-0,65 0,02			lipA	LIPOIC ACID SYNTHETASE (LIP-SYN) (LIPOATE SYNTHASE)	I.B.13
RSc0323	RS03290			-0,58 0,00			lipB	LIPOATE-PROTEIN LIGASE B	I.B.13
RSc0324	RS03291			-0,54 0,00		1,12 0,00		HYPOTHETICAL PROTEIN	VI.B
RSc0325	RS03292			-0,65 0,01				TRANSCRIPTION REGULATOR	VI.A
RSc0326	RS03293			-0,27 0,01				CONSERVED HYPOTHETICAL PROTEIN	VI.C
RSc0327	RS03294			-0,52 0,01			dac	TRANSMEMBRANE PENICILLIN-BINDING PROTEIN (D-ALANYL-D-ALANINE CARE	IV.A
RSc0328	RS03295		0,46 0,02			-0,49 0,05		TRANSMEMBRANE COG2945, Hydrolase of the alpha/beta superfamily	I.I
RSc0329	RS03296		0,38 0,02	-0,44 0,00				FERREDOXIN 2FE-2S	I.G.1
RSc0330	RS03297		0,38 0,01	-1,24 0,00			dctA1	TRANSMEMBRANE C4-DICARBOXYLATE TRANSPORT PROTEIN	IV.D.3
RSc0331	RS03298				-1,69 0,02		dctB2	TRANSCRIPTION REGULATOR C4-DICARBOXYLATE TRANSPORT SENSOR KIN/	IV.D.3
RSc0332	RS03299				-0,47 0,02		dctD2	TRANSCRIPTION REGULATOR C4-DICARBOXYLATE TRANSPORT RESPONSE R	IV.D.3
RSc0333	RS03300			-0,82 0,01				TRANSMEMBRANE COG0526, Thiol-disulfide isomerase and thioredoxins	II.B.7
RSc0334	RS03301			-0,66 0,00	-0,60 0,04			COG2110, Phosphatase homologous to the C-terminal domain of histone macroH2A1	VI.C
RSc0335	RS03302			-0,49 0,01				TRANSMEMBRANE	VI.C
RSc0336	RS03303			-0,72 0,00				SIGNAL PEPTIDE	VI.D
RSc0337	RS03304			-0,88 0,01				OXIDOREDUCTASE POLYPHENOL OXIDASE B PRECURSOR (CATECHOL OXIDA	I.I.4
RSc0338	RS03305			-0,54 0,03				COG0606, ATPase with chaperone activity	II.B.7

RSc0339	RS03306					-0,57	0,00			0,74	0,00		TRANSMEMBRANE	VI.C
RSc0340	RS03307							0,67	0,01	0,57	0,02		CONSERVED HYPOTHETICAL PROTEIN	VI.C
RSc0341	RS03308					-0,39	0,01	-1,51	0,02				SIGNAL PEPTIDE	VI.C
RSc0342	RS03309											glnK	TRANSCRIPTION REGULATOR NITROGEN REGULATORY PROTEIN P-II	I.A.18
RSc0343	RS03310					-0,45	0,01					amtB	TRANSMEMBRANE AMMONIUM TRANSPORTER	I.F.18
RSc0344	RS03311												CONSERVED HYPOTHETICAL PROTEIN	VI.C
RSc0345	RS03312											gshB	GLUTATHIONE SYNTHETASE	I.B.6
RSc0346	RS03313					-0,53	0,04			-1,85	0,04		COG2893, Phosphotransferase system, mannose/fructose-specific component IIA	IV.D.3
RSc0347	RS03314			0,55	0,03							pstH	PHOSPHOCARRIER PROTEIN HPR (phosphohistidinoprotein-hexose phosphotransf	IV.D.3
RSc0348	RS03315	0,68	0,02	0,43	0,03	-0,42	0,00					ptsI	TRANSMEMBRANE PHOSPHOENOLPYRUVATE-PROTEIN PHOSPHOTRANSFER	IV.D.3
RSc0349	RS03316					-1,63	0,00	-1,44	0,00	-0,80	0,02	bfd	BACTERIOFERRITIN-ASSOCIATED FERREDOXIN	IV.F.1
RSc0350	RS03317					-0,24	0,02	-0,78	0,00	-0,78	0,00	bfr	BACTERIOFERRITIN BFR (CYTOCHROME B-1) (CYTOCHROME B-557)	IV.F.1
RSc0351	RS03318			0,64	0,01							thiF	TRANSMEMBRANE THIF PROTEIN	I.B.8
RSc0352	RS03319			0,34	0,02	-0,46	0,00						TRANSMEMBRANE COG0793, Periplasmic protease	II.A.4
RSc0353	RS03320							0,81	0,00			gpmA	PHOSPHOGLYCERATE MUTASE 1 (PHOSPHOGLYCEROMUTASE 1)	I.H.8
RSc0354	RS03321			0,72	0,03								TRANSMEMBRANE COG0607, Rhodanese-related sulfurtransferase	I
RSc0355	RS03322					-0,48	0,01					grxC	GLUTAREDOXIN 3 (GRX3)	I.B.6
RSc0356	RS03323			0,50	0,03							secB	PROTEIN-EXPORT PROTEIN SECB	IV.E.2
RSc0357	RS03324			1,07	0,00								OXIDOREDUCTASE GLYCEROL-3-PHOSPHATE DEHYDROGENASE (NAD(P)+)	I.H.3
RSc0358	RS03326											yibK	TRNA/RRNA METHYLTRANSFERASE	II.B.6
RSc0359	RS03327												COMPETENCE PROTEIN F-RELATED PROTEIN	IV.E
RSc0360	RS03328			0,89	0,04							bioC	BIOTIN SYNTHESIS PROTEIN (METHYLTRANSFERASE)	I.B.15
RSc0361	RS03329	0,39	0,04	0,39	0,02			0,87	0,00	0,85	0,01		TRANSMEMBRANE	VI.C
RSc0362	RS03330			0,74	0,00			0,82	0,01	1,02	0,01	coxB	TRANSMEMBRANE CYTOCHROME C OXIDASE POLYPEPTIDE II PRECURSOR ((	I.G.1
RSc0363	RS03331			0,67	0,05			1,23	0,00	1,74	0,00	coxA	TRANSMEMBRANE CYTOCHROME C OXIDASE POLYPEPTIDE I (CYTOCHROME	I.G.1
RSc0364	RS03332							1,11	0,00	1,46	0,00		TRANSMEMBRANE	VI.D
RSc0365	RS03333			0,81	0,00			0,75	0,02	1,36	0,00	ctaG	TRANSMEMBRANE CYTOCHROME C OXIDASE ASSEMBLY PROTEIN	I.H.3
RSc0366	RS03334			0,34	0,00	-0,30	0,03			1,11	0,00		TRANSMEMBRANE	VI.C
RSc0367	RS03335			0,57	0,00					1,16	0,00	coxC	TRANSMEMBRANE CYTOCHROME C OXIDASE SUBUNIT III	I.G.1
RSc0368	RS03336												TRANSMEMBRANE	VI.C
RSc0369	RS03337												TRANSMEMBRANE TRANSMEMBRANE CYTOCHROME OXIDASE COMPLEX BIO	I.H.3
RSc0370	RS03338			0,72	0,04								TRANSMEMBRANE	VI.C
RSc0371	RS03339			1,08	0,05	-0,52	0,02					ctaA	TRANSMEMBRANE HEME O OXYGENASE (CYTOCHROME AA3-CONTROLLING F	I.H.3
RSc0372	RS03340			0,91	0,00	-0,31	0,00					ctaB	TRANSMEMBRANE PROTOHEME IX FARNESYLTRANSFERASE (HEME O SYNTH	I.H.3
RSc0373	RS03341			0,44	0,00	-0,67	0,00	0,72	0,03	1,22	0,00		LIPOPROTEIN	VI.C
RSc0374	RS03342					-0,49	0,00					rpoH	TRANSCRIPTION REGULATOR RNA POLYMERASE SIGMA H (SIGMA-32) FACTO	I.E.1
RSc0375	RS03343					-0,76	0,05						TRANSMEMBRANE SIGNAL TRANSDUCTION PROTEIN GGDEF DOMAIN	VI.A
RSc0376	RS03344			0,35	0,01								COG1765, Redox protein, regulator of disulfide bond formation	II.B.7
RSc0377	RS03345												PIRIN-RELATED PROTEIN	VI.C
RSc0378	RS03346			0,33	0,05	-0,35	0,02					trpD3	ANTHRANILATE PHOSPHORIBOSYLTRANSFERASE	I.A.6
RSc0379	RS03347											nasD	ABC TRANSPORTER NITRATE ATP-BINDING PROTEIN	IV.D.1
RSc0380	RS03348							-3,61	0,04			nasE	ABC TRANSPORTER NITRATE TRANSMEMBRANE PROTEIN	IV.D.1
RSc0381	RS03349											nasF	NITRATE TRANSPORTER	IV.D.1
RSc0382	RS03350							-1,43	0,00				TRANSCRIPTION REGULATOR RESPONSE REGULATOR	VI.A
RSc0383	RS03351							1,48	0,01			mhpD	OXIDOREDUCTASE COG0179, 2-keto-4-pentenoate hydratase / 2-oxohepta-3-ene-1	I
RSc0384	RS03352											gstB	GLUTATHIONE S-TRANSFERASE	II.B.7
RSc0385	RS03353			0,44	0,01								TRANSMEMBRANE COG1279, LysE-type efflux permease	IV.D
RSc0386	RS03354			0,66	0,03							ftsY	CELL DIVISION PROTEIN	IV.A
RSc0387	RS03355					-0,61	0,00	0,73	0,05				COG0612, Predicted Zn-dependent peptidases	I.I.3
RSc0388	RS03356					-0,43	0,00			-0,40	0,02		SIGNAL PEPTIDE COG0612, Predicted Zn-dependent peptidases	I.I.3
RSc0389	RS03357			1,02	0,01	-0,51	0,01						COG0742, N6-adenine-specific methylase	II.B.8
RSc0390	RS03358					-0,51	0,00					kdtB	PHOSPHOPANTETHEINE ADENYLYLTRANSFERASE (PANTETHEINE- PHOSPHA	III.A.1
RSc0391	RS03359					-1,05	0,00	-0,80	0,00				FERREDOXIN	I.G.1
RSc0392	RS03360					-0,76	0,00	-2,25	0,00				SIGNAL PEPTIDE PROLIN-RICH PROTEIN	VI.D
RSc0393	RS03361							-0,77	0,05	-0,75	0,01	pth	PEPTIDYL-TRNA HYDROLASE	II.D.1
RSc0394	RS03362			0,88	0,02					-1,04	0,00	rplY	50S RIBOSOMAL SUBUNIT PROTEIN L25	III.C.2
RSc0395	RS03363					-0,36	0,00			-1,86	0,00	prsA	RIBOSE-PHOSPHATE PYROPHOSPHOKINASE	I.D.2
RSc0396	RS03364											ipk	ISOPENTENYL MONOPHOSPHATE KINASE (4-CYTIDINE-5'-DIPHOSPHO-2-C-ME	I.F
RSc0397	RS03365											lolB	COG3017, Outer membrane lipoprotein involved in outer membrane biogenesis	III.B.2
RSc0398	RS03366					-0,56	0,01	1,26	0,01				TRANSMEMBRANE	VI.C
RSc0399	RS03367			0,37	0,01	-0,49	0,00					mutM	FORMAMIDOPYRIMIDINE-DNA GLYCOSYLASE	II.B.8
RSc0400	RS03368					-0,40	0,00	-0,42	0,05	-0,35	0,03		COG0699, Predicted GTPase (dynamin-related)	VI.C
RSc0401	RS03369											mutY	A/G-SPECIFIC ADENINE GLYCOSYLASE	II.B.8
RSc0402	RS03370					-0,42	0,02						CONSERVED HYPOTHETICAL PROTEIN	VI.C
RSc0403	RS03371					-0,59	0,05						COG1660, Predicted P-loop-containing kinase	VI.C
RSc0404	RS03372												SIGNAL PEPTIDE	VI.C
RSc0405	RS03373									-0,37	0,01	ptsK	HPR KINASE AND PHOSPHATASE	I.F
RSc0406	RS03374					-0,89	0,00					ptsN	TRANSCRIPTION REGULATOR NITROGEN REGULATORY IIA PROTEIN (ENZYME	I.E.1
RSc0407	RS03375					-0,86	0,01	-0,84	0,02				SIGMA-54 MODULATION PROTEIN	I.E.1
RSc0408	RS03376					-1,46	0,00					rpoN1	TRANSCRIPTION REGULATOR RNA POLYMERASE SIGMA N (SIGMA 54) FACTO	I.E.1
RSc0409	RS03377			0,69	0,01								ABC TRANSPORTER ATP-BINDING PROTEIN	IV.D
RSc0410	RS03378					-0,43	0,02						SIGNAL PEPTIDE	VI.C
RSc0411	RS03379					-0,69	0,02						TRANSMEMBRANE	VI.C
RSc0412	RS03380					-0,53	0,03					yrbI	3-DEOXY-D-MANNO-OCTULOSONATE 8-PHOSPHATE(KDO 8-P)PHOSPHATASE	II.B.2
RSc0413	RS03381			0,59	0,04	-0,38	0,02						COG0794, Predicted sugar phosphate isomerase involved in capsule formation	III.B
RSc0414	RS03382					-0,22	0,02						TRANSMEMBRANE GLUTATHIONE-REGULATED POTASSIUM-EFFLUX SYSTEM I	IV.D.4
RSc0415	RS03383	-0,86	0,04	-1,19	0,02	-0,87	0,00	-1,33	0,00	-1,55	0,00		CONSERVED HYPOTHETICAL PROTEIN	VI.C
RSc0416	RS03384			-1,49	0,02	-0,44	0,02	-3,55	0,00	-3,07	0,00		COG0693, Intracellular protease/amidase	I.I
RSc0417	RS03385					-0,36	0,02					apt	ADENINE PHOSPHORIBOSYLTRANSFERASE (APRT)	I.F.1
RSc0418	RS03386												TRANSMEMBRANE TRANSPORT PROTEIN	IV.D
RSc0419	RS03387					-1,60	0,00	-1,21	0,00				TRANSMEMBRANE	VI.C
RSc0420	RS03388			0,84	0,00							uvrA1	ABC TRANSPORTER EXCINUCLEASE ABC SUBUNIT A (DNA REPAIR ATP-BINDIN	II.B.8
RSc0421	RS03389												TRANSMEMBRANE MULTIDRUG-EFFLUX TRANSPORTER	IV.D
RSc0422	RS03390											ssb	SINGLE-STRAND BINDING PROTEIN (HELIX-DESTABILIZING PROTEIN)	II.B.8
RSc0423	RS03392					-0,45	0,02						REMNANT OF A TRANSPOSASE	V.A
RSc0424	RS03393												SIGNAL PEPTIDE	VI.C
RSc0425	RS03394												COG4706, 3-hydroxylacyl-(acyl carrier protein) dehydratase	I
RSc0426	RS03395			0,66	0,01								CONSERVED HYPOTHETICAL PROTEIN	VI.C
RSc0427	RS03396							0,68	0,01				3-OXOACYL-[ACYL-CARRIER-PROTEIN] SYNTHASE (BETA- KETOACYL-ACP SYI	I.B.1
RSc0428	RS03397												TRANSMEMBRANE Polysaccharide deacetylase	I.I.4
RSc0429	RS03398												TRANSMEMBRANE COG4258, Predicted exporter	VI.C
RSc0430	RS03399												SIGNAL PEPTIDE	VI.B
RSc0431	RS03400												COG4261 : Predicted acyltransferase	I
RSc0432	RS03401												COG0365, Acyl-coenzyme A synthetases/AMP-(fatty) acid ligases	I
RSc0433	RS03402					-0,49	0,00						TRANSMEMBRANE	VI.C
RSc0434	RS03403					-0,64	0,00			1,08	0,01		COG0236, Acyl carrier protein	I
RSc0435	RS03404									0,71	0,04		OXIDOREDUCTASE 3-OXOACYL-[ACYL-CARRIER-PROTEIN] REDUCTASE	I.B.1
RSc0436	RS03405												COG0463, Glycosyltransferase	I
RSc0437	RS03406					-0,52	0,01						SIGNAL PEPTIDE	VI.C
RSc0438	RS03407												SIGNAL PEPTIDE	VI.C
RSc0439	RS03408			0,86	0,02	-0,41	0,00						LIPASE/ESTERASE	I.I.1
RSc0440	RS04467					-0,94	0,00	-0,84	0,01				SIGNAL PEPTIDE	VI.B
RSc0441	RS04466					-0,46	0,00					gntK	THERMORESISTANT GLUCONOKINASE (GLUCONATE KINASE 2)	I.F.4
RSc0442	RS04463												TRANSMEMBRANE FATTY-ACID--COA LIGASE	I.I.1
RSc0443	RS04459												SIGNAL PEPTIDE GLYCIN-RICH PROTEIN	VI.D
RSc0444	RS04458					-0,59	0,04	-0,86	0,02				SIGNAL PEPTIDE	VI.C
RSc0445	RS04457					-0,33	0,02						SIGNAL PEPTIDE COG3271, Predicted double-glycine peptidase	I.I.3
RSc0446	RS04456												LIPOPROTEIN	VI.B
RSc0447	RS04455					-2,70	0,00	-1,84	0,04				CONSERVED HYPOTHETICAL PROTEIN	VI.C
RSc0448	RS04454			0,83	0,01								CONSERVED HYPOTHETICAL PROTEIN	VI.C
RSc0449	RS04453			0,82	0,00							ilvA	TRANSMEMBRANE THREONINE AMONIA-LYASE (THREONINE DEHYDRATASE, '	I.A.2
RSc0450	RS04452			1,04	0,00	-0,66	0,00						TRANSMEMBRANE ATP-SENSITIVE INWARD RECTIFIER POTASSIUM CHANNEL	IV.D

RSc0451	RS04451			-0,91	0,00	-1,33	0,00				TRANSMEMBRANE	VI.D
RSc0452	RS04450			1,85	0,00						TRANSMEMBRANE	VI.C
RSc0453	RS04448			-0,54	0,04						TRANSMEMBRANE	VI.C
RSc0454	RS04446										OXIDOREDUCTASE COG0247, Fe-S OXIDOREDUCTASE AND COG0277, FAD/FMI	I
RSc0455	RS04445										COG0537, Diadenosine tetraphosphate (Ap4A) hydrolase and other HIT family hydrol	I
RSc0456	RS04444										ABC TRANSPORTER ATP-BINDING TRANSPORT PROTEIN	IV.D
RSc0457	RS04443			-0,44	0,01	0,60	0,00				CONSERVED HYPOTHETICAL PROTEIN	VI.C
RSc0458	RS04441					-0,68	0,00	-0,27	0,04	ubiE	UBIQUINONE/MENAQUINONE BIOSYNTHESIS METHYLTRANSFERASE	I.B.5
RSc0459	RS04440			-0,70	0,00	-0,54	0,04				TRANSMEMBRANE	VI.C
RSc0460	RS04439			-0,45	0,01			-1,61	0,01		CONSERVED HYPOTHETICAL PROTEIN	VI.C
RSc0461	RS04437			-0,72	0,03					aarF	TRANSMEMBRANE UBIQUINONE BIOSYNTHESIS PROTEIN	I.B.5
RSc0462	RS04436	0,71	0,04								CONSERVED HYPOTHETICAL PROTEIN	VI.C
RSc0463	RS04434	0,85	0,01	-0,49	0,00						TRANSMEMBRANE SODIUM/SOLUTE SYMPORTER	IV.D
RSc0464	RS04433	0,49	0,01	-0,34	0,00			-0,36	0,04		CONSERVED HYPOTHETICAL PROTEIN	VI.C
RSc0465	RS04432			-0,51	0,01	-0,64	0,03	-0,45	0,04		TRANSMEMBRANE	VI.C
RSc0466	RS04431	0,90	0,00	-0,35	0,01					aspS	ASPARTYL-TRNA SYNTHETASE	II.D.1
RSc0467	RS04430			-0,57	0,02						TRANSMEMBRANE	VI.C
RSc0468	RS04429			-0,46	0,00					ntpA	DATP PYROPHOSPHOHYDROLASE	I.F.9
RSc0469	RS04427			-0,53	0,00			-0,73	0,01		COG3568, Metal-dependent hydrolase	I.I
RSc0470	RS04425										COG1502, Phosphatidylserine/phosphatidylglycerophosphate/cardiolipin synthases a	I
RSc0471	RS04424	0,73	0,02	-0,49	0,03						COG4670, Acyl CoA:acetate/3-ketoacid CoA transferase	I
RSc0472	RS04423	0,99	0,00	-1,42	0,00	-0,89	0,00				TRANSCRIPTION REGULATOR	VI.A
RSc0473	RS04422	0,77	0,01	-0,72	0,00	-1,11	0,05	-0,78	0,00		OXIDOREDUCTASE ACYL-CoA DEHYDROGENASE	I.I.1
RSc0474	RS04421	0,90	0,01	-0,48	0,01						OXIDOREDUCTASE 3-HYDROXYACYL-COA DEHYDROGENASE	I.I.1
RSc0475	RS04420	0,80	0,01	-0,56	0,00						ACETYL-COA ACYLTRANSFERASE	I.C
RSc0476	RS04419										ENOYL-COA HYDRATASE	I.I.4
RSc0477	RS04418			-0,79	0,00						COG1607, Acyl-CoA hydrolase	I
RSc0478	RS04417			-0,66	0,01						ABC TRANSPORTER COMPOSITE ATP-BINDING TRANSMEMBRANE PROTEIN	IV.D
RSc0479	RS04416			-0,47	0,01						TRANSCRIPTION REGULATOR	VI.A
RSc0480	RS04415	0,49	0,01	-0,36	0,04					gdhA	OXIDOREDUCTASE GLUTAMATE DEHYDROGENASE (NAD(P)+)	I.A.19
RSc0481	RS04394	0,36	0,05	-0,54	0,00	0,82	0,00				ABC TRANSPORTER AMINO-ACID-BINDING PERIPLASMIC PROTEIN (PBP)	IV.D
RSc0482	RS04391	0,72	0,00	-0,36	0,00	0,95	0,03	-0,62	0,01	gltJ	ABC TRANSPORTER GLUTAMATE/ASPARTATE TRANSMEMBRANE PROTEIN	IV.D.2
RSc0483	RS04390	0,88	0,00	-0,27	0,02			-1,31	0,01	gltK	ABC TRANSPORTER GLUTAMATE/ASPARTATE TRANSMEMBRANE PROTEIN	IV.D.2
RSc0484	RS04389			-0,41	0,00			-0,88	0,00	gltL	ABC TRANSPORTER GLUTAMATE/ASPARTATE TRANSPORT ATP-BINDING PRO	IV.D.2
RSc0485	RS04388			-0,79	0,00	-0,89	0,00				COG0121, Predicted glutamine amidotransferase	I
RSc0486	RS05749			-0,30	0,01					ttuD2	OXIDOREDUCTASE HYDROXYPYRUVATE REDUCTASE	I.I.4
RSc0487	RS05822	1,20	0,01							pyrC	DIHYDROOROTASE	I.D.1
RSc0488	RS05751										CONSERVED HYPOTHETICAL PROTEIN	VI.C
RSc0489	RS05752	0,59	0,03	-0,63	0,00	-0,64	0,00				COG1765, Predicted redox protein, regulator of disulfide bond formation	II.B.7
RSc0490	RS05035							-1,05	0,01	rplM	50S RIBOSOMAL PROTEIN L13	III.C.2
RSc0491	RS05034	0,88	0,03					-0,98	0,04	rpsI	30S RIBOSOMAL PROTEIN S9	III.C.2
RSc0492	RS05033			-0,49	0,00			-0,94	0,00		TRANSMEMBRANE	VI.C
RSc0493	RS05032			-0,42	0,01					ccmA	COG1664, Integral membrane protein CcmA involved in cell shape determination	III.B
RSc0494	RS05030			-0,22	0,01	-0,97	0,00				CONSERVED HYPOTHETICAL PROTEIN	VI.C
RSc0495	RS05029			-0,63	0,00						COG2377, Predicted molecular chaperone distantly related to HSP70-fold metalloprot	II.B.7
RSc0496	RS05028	0,82	0,05	-0,17	0,05	1,21	0,01			tyrS	TYROSYL-TRNA SYNTHETASE	II.D.1
RSc0497	RS05026	0,52	0,04			0,89	0,01				COG1490, D-Tyr-tRNAtyr deacylase	II.D.1
RSc0498	RS05025										HYPOTHETICAL PROTEIN	VI.D
RSc0499	RS05023							-0,97	0,02	gpmB	PHOSPHOGLYCERATE MUTASE 2	I.H.8
RSc0500	RS05022	0,75	0,05			0,88	0,02			ruvB	HOLLIDAY JUNCTION DNA HELICASE	II.B.8
RSc0501	RS05021									ruvA	HOLLIDAY JUNCTION DNA HELICASE	II.B.8
RSc0502	RS05020			-0,73	0,00					glpQ	GLYCEROPHOSPHORYL DIESTER PHOSPHODIESTERASE, PERIPLASMIC PRE	I.F.11
RSc0503	RS05019			-0,44	0,01					ruvC	HOLLIDAY JUNCTION ENDODEOXYRIBONUCLEASE	II.B.8
RSc0504	RS05018							-1,20	0,00	purH	TRANSMEMBRANE BIFUNCTIONAL PROTEIN : PHOSPHORIBOSYLAMINOIMIDAZ	I.D.2
RSc0505	RS05017			-0,52	0,00					fis	TRANSCRIPTION REGULATOR FACTOR-FOR-INVERSION-STIMULATION PROTE	II.C
RSc0506	RS05002							-0,39	0,01		COG0042, tRNA-dihydrouridine synthase	II.D.1
RSc0507	RS05000										TRANSMEMBRANE AMINO ACID DEHYDROGENASE	I.I.3
RSc0508	RS04999									ubiH	OXIDOREDUCTASE 2-OCTAPRENYL-6-METHOXYPHENOL HYDROXYLASE	I.H.3
RSc0509	RS04998							0,68	0,00	pepP	XAA-PRO AMINOPEPTIDASE (AMINOPEPTIDASE P II)	II.A.4
RSc0510	RS04997										TRANSMEMBRANE SIGNAL TRANSDUCTION PROTEIN EAL - GGDEF DOMAINS	VI.A
RSc0511	RS04996	0,51	0,02								COG1208, Nucleoside-diphosphate-sugar pyrophosphorylase involved in lipopolysacc	VI.C
RSc0512	RS04995	-0,52	0,02	0,99	0,00						TRANSMEMBRANE	VI.C
RSc0513	RS04992	1,01	0,01								TRANSMEMBRANE COG1296, Predicted branched-chain amino acid permease (azal	IV.D.2
RSc0514	RS04986	0,47	0,01								COG3178, Predicted phosphotransferase related to Ser/Thr protein kinases	VI.C
RSc0515	RS04980			-0,32	0,02						TRANSMEMBRANE ORGANIC SOLVENT TOLERANCE PROTEIN	IV.F.1
RSc0516	RS04977			-0,35	0,02			-0,93	0,00	surA	PEPTIDYL-PROLYL CIS-TRANS ISOMERASE (ROTAMASE C)	IV.B
RSc0517	RS04975			-0,38	0,01					pdxA	PYRIDOXAL PHOSPHATE BIOSYNTHETIC PROTEIN	I.B.10
RSc0518	RS04973			-0,21	0,02					ksgA	DIMETHYLADENOSINE TRANSFERASE (16S RRNA DIMETHYLASE)	IV.G.2
RSc0519	RS04969										TRANSMEMBRANE	VI.C
RSc0520	RS04961	0,88	0,02							gloA	LACTOYLGLUTATHIONE LYASE (METHYLGLYOXALASE)	I.F.17
RSc0521	RS04960	-0,74	0,04								COG1451, Predicted metal-dependent hydrolase	I.I
RSc0522	RS04959	0,72	0,00	-0,32	0,05						TRANSMEMBRANE PHOSPHATE ACYLTRANSFERASE	I
RSc0523	RS04957	1,24	0,00								COG0241, Histidinol phosphatase and related phosphatases	I
RSc0524	RS04944			-0,30	0,00			-0,74	0,00	glyS	GLYCYL-TRNA SYNTHETASE BETA CHAIN	II.D.1
RSc0525	RS04943										SIGNAL PEPTIDE	VI.C
RSc0526	RS04942					1,07	0,03	-1,33	0,03	glyQ	GLYCYL-TRNA SYNTHETASE ALPHA CHAIN	II.D.1
RSc0527	RS04941	1,13	0,00	-0,73	0,00	-0,88	0,03			lnt	TRANSMEMBRANE APOLIPOPROTEIN N-ACYLTRANSFERASE	IV.F.1
RSc0528	RS04940	0,49	0,02	-0,60	0,00						COG4535, Putative Mg2+ and Co2+ transporter	IV.D
RSc0529	RS04939										COG0319, Predicted metal-dependent hydrolase	I.I
RSc0530	RS04938									phoH	Phosphate starvation-inducible protein PhoH, predicted ATPase	IV.F.1
RSc0531	RS04937			-0,29	0,00						COG0621, 2-methylthioadenine synthetase	II.B.7
RSc0532	RS04936			-0,43	0,03						SIGNAL PEPTIDE	VI.C
RSc0533	RS04935										OXIDOREDUCTASE COG3380, Predicted NAD/FAD-dependent oxidoreductase	I
RSc0534	RS04933	0,46	0,03	-0,71	0,01						TRANSMEMBRANE LIPOPROTEIN	VI.C
RSc0535	RS04931	0,92	0,04								SIGNAL PEPTIDE	VI.B
RSc0536	RS04930										TRANSMEMBRANE SHORT-CHAIN DEHYDROGENASE	I.I
RSc0537	RS04929			-1,04	0,00						OXIDOREDUCTASE TRANSMEMBRANE ACYL-COA DEHYDROGENASE	I.I
RSc0538	RS04928			-1,23	0,01						TRANSCRIPTION REGULATOR	VI.A
RSc0539	RS04927			-0,50	0,01	-0,41	0,00	-0,50	0,00	rhlE1	ATP-DEPENDENT RNA HELICASE	II.B.6
RSc0540	RS04926	0,90	0,03								COG2334, Putative homoserine kinase type II (protein kinase fold)	VI.C
RSc0541	RS04925	1,04	0,01	-1,65	0,00						PIRIN-LIKE PROTEIN	VI.C
RSc0542	RS04924					-1,08	0,00				TRANSCRIPTION REGULATOR	VI.A
RSc0543	RS04923			-0,67	0,00						TRANSCRIPTION REGULATOR RESPONSE REGULATOR	VI.A
RSc0544	RS04922			-0,35	0,00						CONSERVED HYPOTHETICAL PROTEIN	VI.C
RSc0545	RS04921			-0,67	0,04						OXIDOREDUCTASE	I.I
RSc0546	RS04920										TRANSMEMBRANE	VI.C
RSc0547	RS04919										TRANSMEMBRANE SUGAR TRANSPORTER	IV.D.3
RSc0548	RS04918										TRANSMEMBRANE	VI.C
RSc0549	RS04917					-0,59	0,05				TRANSCRIPTION REGULATOR TRANSMEMBRANE SENSOR HISTIDINE KINASE	VI.A
RSc0550	RS04916	0,39	0,05	-0,70	0,01						TRANSCRIPTION REGULATOR RESPONSE REGULATOR	VI.A
RSc0551	RS04915							-0,59	0,02	recA	DNA STRAND EXCHANGE AND RECOMBINATION PROTEIN	II.B.8
RSc0552	RS04913			-0,36	0,01					recX	REGULATORY PROTEIN RECX	VI.A
RSc0553	RS04912			-0,47	0,02	-1,23	0,02	-1,74	0,00		CONSERVED HYPOTHETICAL PROTEIN	VI.C
RSc0554	RS04911							-1,54	0,00	sucC	SUCCINYL-COA SYNTHETASE BETA CHAIN	I.H.6
RSc0555	RS04910	1,17	0,04					-1,68	0,00	sucD	SUCCINYL-COA SYNTHETASE ALPHA CHAIN	I.H.6
RSc0556	RS04909			-0,65	0,02						TRANSMEMBRANE COG0861, Membrane protein TerC, possibly involved in tellurium	III.B.1
RSc0557	RS04908			-0,29	0,00					plpA	SIGNAL PEPTIDE TYPE 4 FIMBRIAL PILIN-LIKE PROTEIN	III.A.2
RSc0558	RS04907	0,79	0,01	-0,62	0,00					pilA	SIGNAL PEPTIDE TYPE 4 FIMBRIAL PILIN	III.A.2
RSc0559	RS04906	0,84	0,02	-0,41	0,03						TRANSMEMBRANE COG3307, Lipid A core - O-antigen ligase and related enzymes	III.A.1
RSc0560	RS04905									moaC	MOLYBDENUM COFACTOR BIOSYNTHESIS PROTEIN C	I.B.12
RSc0561	RS04904	0,98	0,01								SIGNAL PEPTIDE COG4783, Putative Zn-dependent protease, contains TPR repeats	II.A.4
RSc0562	RS04903			-0,56	0,00						CONSERVED HYPOTHETICAL PROTEIN	VI.C

RSc0563	RS04902	1,16 0,01	0,53 0,02	-0,73 0,00				COG0429, Predicted hydrolase of the alpha/beta-hydrolase fold	I.I
RSc0564	RS04901							CONSERVED HYPOTHETICAL PROTEIN	VI.C
RSc0565	RS04900		0,61 0,01	-0,41 0,03			rfaF	ADP-HEPTOSE--LIPOPOLYSACCHARIDE HEPTOSYLTRANSFERASE II	II.B.2
RSc0566	RS04899				1,11 0,02			CONSERVED HYPOTHETICAL PROTEIN	VI.C
RSc0567	RS04898			-0,46 0,00				BRANCHED-CHAIN AMINO ACID AMINOTRANSFERASE	IV.D
RSc0568	RS04897			-0,60 0,00				TRANSMEMBRANE COG1296, Predicted branched-chain amino acid permease (azal	IV.D
RSc0569	RS04896			-0,58 0,01				TRANSMEMBRANE	VI.C
RSc0570	RS04895							TRANSMEMBRANE COG2962, Predicted permeases	IV.D
RSc0571	RS04894		0,53 0,01		0,95 0,02	-0,48 0,01	pgk	PHOSPHOGLYCERATE KINASE	I.H.8
RSc0572	RS04893		0,59 0,00	-0,17 0,05			pykA1	PYRUVATE KINASE II	I.H.8
RSc0573	RS04892		0,76 0,01				fbaA	FRUCTOSE-BISPHOSPHATE ALDOLASE	I.I.4
RSc0574	RS04891		0,73 0,00			-0,63 0,00	purC	PHOSPHORIBOSYLAMINOIMIDAZOLE-SUCCINOCARBOXAMIDE SYNTHASE	I.D.2
RSc0575	RS04890		0,91 0,01				purE	PHOSPHORIBOSYLAMINOIMIDAZOLE CARBOXYLASE CATALYTIC SUBUNIT	I.D.2
RSc0576	RS04889		0,78 0,01		0,50 0,03	-0,60 0,01	purK	PHOSPHORIBOSYLAMINOIMIDAZOLE CARBOXYLASE ATPASE SUBUNIT	I.D.2
RSc0577	RS04888		0,45 0,04					COG0009, Putative translation factor (SUA5)	III.C.2
RSc0580	RS04885			-0,64 0,04				LIPASE / ESTERASE	I.I.1
RSc0581	RS04884	-1,11 0,00	0,61 0,01		1,33 0,00	1,88 0,00		PHOSPHOGLYCOLATE PHOSPHATASE	I.I.4
RSc0582	RS04881	-1,98 0,00				1,70 0,00		AVRD-RELATED PROTEIN	VI.C
RSc0583	RS04880						mobB2	MOLYBDOPTERIN-GUANINE DINUCLEOTIDE BIOSYNTHESIS PROTEIN B	I.F.9
RSc0584	RS04879							SIGNAL PEPTIDE D-ALANYL-D-ALANINE CARBOXYPEPTIDASE	II.A.4
RSc0585	RS04878		0,72 0,04	-0,53 0,01				TRANSMEMBRANE TRANSPORT PROTEIN	IV.D
RSc0586	RS04876					0,62 0,02		CONSERVED HYPOTHETICAL PROTEIN	VI.C
RSc0587	RS04875		-1,01 0,00	1,20 0,00				TRANSMEMBRANE	VI.C
RSc0588	RS04873		0,73 0,02					SIGNAL TRANSDUCTION PROTEIN EAL - GGDEF DOMAINS	VI.A
RSc0589	RS04871		0,34 0,02	-0,49 0,00				COG0154, Asp-tRNAAsn/Glu-tRNAGln amidotransferase A subunit and related amida	II.D.1
RSc0590	RS04869			-0,41 0,00		-1,24 0,00		TRANSCRIPTION REGULATOR	VI.A
RSc0591	RS04867			-1,08 0,00				CONSERVED HYPOTHETICAL PROTEIN	VI.C
RSc0592	RS04854			-0,59 0,05	-1,29 0,01			COG2206, HD-GYP domain	VI.A
RSc0593	RS04853			-0,94 0,00	-1,71 0,00			HYPOTHETICAL PROTEIN	VI.D
RSc0594	RS04852			-3,19 0,00		-0,80 0,01		TRANSMEMBRANE	VI.D
RSc0595	RS04851			-2,11 0,00		-2,49 0,00		SIGNAL PEPTIDE OUTER MEMBRANE RECEPTOR	III.B.2
RSc0596	RS04849			-1,82 0,00				HYPOTHETICAL PROTEIN	VI.D
RSc0597	RS04848							HYPOTHETICAL PROTEIN	VI.D
RSc0598	RS04847				-0,53 0,00			HYPOTHETICAL PROTEIN	VI.D
RSc0599	RS04846		0,46 0,01	-0,48 0,00	-2,78 0,03			TRANSCRIPTION REGULATOR	VI.A
RSc0600	RS04845							CONSERVED HYPOTHETICAL PROTEIN	VI.C
RSc0601	RS04844							TRANSMEMBRANE	VI.C
RSc0602	RS04843		2,31 0,05					TRANSMEMBRANE	VI.C
RSc0603	RS04842			-0,45 0,02				TRANSMEMBRANE	VI.B
RSc0604	RS04840			-0,17 0,02				OXIDOREDUCTASE HYDROLASE	I.I
RSc0605	RS04838		0,57 0,01			1,24 0,00	adhC2	OXIDOREDUCTASE BIFUNCTIONAL PROTEIN: GLUTATHIONE-DEPENDENT FOR	I.H
RSc0606	RS04837							TRANSMEMBRANE METHYL-ACCEPTING CHEMOTAXIS PROTEIN	IV.C
RSc0607	RS04835		0,67 0,01	-0,32 0,01			gstC	GLUTATHIONE S-TRANSFERASE	II.B.7
RSc0608	RS04833	-2,92 0,00	-7,38 0,00	6,84 0,00	3,70 0,00	-3,81 0,00	avrA	TYPE III EFFECTOR PROTEIN AVRA	VI.E
RSc0609	RS04832		-4,65 0,02	1,56 0,00	2,32 0,00			LIPOPROTEIN	VI.D
RSc0610	RS01511			1,25 0,00	1,62 0,00			SIGNAL PEPTIDE	VI.C
RSc0611	RS01512		-0,98 0,03	0,91 0,00	2,41 0,00			TRANSMEMBRANE ABPE FAMILY PROTEIN	VI.C
RSc0612	RS01513			1,41 0,01				TRANSMEMBRANE	VI.D
RSc0613	RS01514			-0,88 0,01	0,78 0,00	1,73 0,00		HYPOTHETICAL PROTEIN	VI.D
RSc0614	RS01515			1,21 0,00				TRANSMEMBRANE PERMEASE	IV.D
RSc0615	RS01516							TRANSCRIPTION REGULATOR	VI.A
RSc0616	RS01517	-3,31 0,00	-6,95 0,00	8,05 0,00	4,41 0,00	-4,64 0,00		CONSERVED HYPOTHETICAL PROTEIN	VI.C
RSc0617	RS01518	-2,60 0,01	-6,69 0,00	7,94 0,00	4,64 0,00	-3,09 0,00		SIGNAL PEPTIDE	VI.C
RSc0618	RS01519		0,52 0,03		-0,79 0,01		rdgC	RECOMBINATION ASSOCIATED PROTEIN RDGC	II.B.8
RSc0619	RS01520			-0,73 0,03				OXIDOREDUCTASE TRANSMEMBRANE HYDROGENASE CYTOCHROME B-TYPE	I.G
RSc0620	RS01521					1,59 0,01		COG2041, Sulfite oxidase and related enzymes	VI.C
RSc0621	RS01522				1,46 0,01			CONSERVED HYPOTHETICAL PROTEIN	VI.C
RSc0622	RS01523						dbpA	ATP-DEPENDENT RNA HELICASE	II.B.6
RSc0623	RS01524							CONSERVED HYPOTHETICAL PROTEIN	VI.C
RSc0624	RS01526		-0,91 0,00	1,45 0,00				OXIDOREDUCTASE ZINC-DEPENDENT ALCOHOL DEHYDROGENASE	I
RSc0625	RS01527							TRANSCRIPTION REGULATOR	VI.A
RSc0626	RS01528			-0,54 0,00	-0,49 0,04			OXIDOREDUCTASE ALDEHYDE DEHYDROGENASE (SUBUNIT III)	I.I.4
RSc0627	RS01529							TRANSMEMBRANE DEHYDROGENASE LARGE CHAIN	I.I.4
RSc0628	RS01530							COG1975, Xanthine and CO dehydrogenases maturation factor	II.B.7
RSc0629	RS01531							SIGNAL PEPTIDE OXIDOREDUCTASE CYTOCHROME C552 PRECURSOR	I.G.1
RSc0630	RS01532							CONSERVED HYPOTHETICAL PROTEIN	VI.C
RSc0631	RS01533			-0,39 0,00				TRANSMEMBRANE	VI.C
RSc0632	RS01534				1,70 0,00			OXIDOREDUCTASE COG1064, Zn-dependent alcohol dehydrogenases	VI.C
RSc0633	RS01535							COG0655, Multimeric flavodoxin	VI.C
RSc0634	RS01536			-0,54 0,01				TRANSCRIPTION REGULATOR	VI.A
RSc0635	RS01538		0,98 0,01					TRANSCRIPTION REGULATOR	VI.A
RSc0636	RS01542			-0,58 0,00				TRUNCATED OXIDOREDUCTASE (N-TERMINAL PART)	VI.B
RSc0638	RS01540							TRUNCATED OXIDOREDUCTASE (C-TERMINAL PART)	VI.B
RSc0639	RS01543			-0,53 0,04				HEAT SHOCK PROTEIN	IV.B
RSc0640	RS01544							HYPOTHETICAL PROTEIN	VI.D
RSc0641	RS01545			0,72 0,00	0,53 0,01		mopB	10 KDA CHAPERONIN (PROTEIN CPN10) (PROTEIN GROES)	IV.B
RSc0642	RS01546		0,80 0,00	0,50 0,04	0,89 0,00		mopA	HAPERONIN (PROTEIN CPN60) (GROEL PROTEIN) (AMS)	IV.B
RSc0643	RS01547			-0,59 0,00				TRANSMEMBRANE	VI.C
RSc0644	RS01548		0,59 0,02	-0,32 0,00				TRANSMEMBRANE	VI.C
RSc0645	RS01549							CONSERVED HYPOTHETICAL PROTEIN	VI.C
RSc0646	RS01550			-0,92 0,05				CONSERVED HYPOTHETICAL PROTEIN	VI.C
RSc0647	RS01551			-1,80 0,00	-2,45 0,05			TRANSMEMBRANE	VI.C
RSc0648	RS01552							TRANSMEMBRANE	VI.C
RSc0649	RS01553						tadG1	TRANSMEMBRANE PILUS ASSEMBLY PROTEIN TADG	III.A.2
RSc0650	RS01554						tadC1	TRANSMEMBRANE PILUS ASSEMBLY PROTEIN TADC	III.A.2
RSc0651	RS01555						tadB1	TRANSMEMBRANE PILUS ASSEMBLY PROTEIN TADB	III.A.2
RSc0652	RS01556						cpaF1	SECRETION ATPase	III.A.2
RSc0653	RS01557	-1,41 0,01			0,65 0,05		cpaE1	PILUS ASSEMBLY PROTEIN	III.A.2
RSc0654	RS01558		0,78 0,03					LIPOPROTEIN	VI.D
RSc0655	RS01559						cpaC1	PILUS ASSEMBLY PROTEIN, SECRETIN CPAC	III.A.2
RSc0656	RS01560						cpaB1	TRANSMEMBRANE PILUS ASSEMBLY PROTEIN CPAB	III.A.2
RSc0657	RS01561				0,65 0,02		cpaA1	TRANSMEMBRANE PREPILIN PEPTIDASE	III.A.2
RSc0658	RS01562							TRANSMEMBRANE PILIN	III.A.2
RSc0659	RS01563		0,69 0,02					PILIN	III.A.2
RSc0660	RS01564		0,42 0,02	-0,35 0,00				TRANSMEMBRANE PILIN	III.A.2
RSc0661	RS01566							TRANSMEMBRANE PILIN	III.A.2
RSc0662	RS01567			-0,57 0,00		-0,36 0,01		CONSERVED HYPOTHETICAL PROTEIN	VI.C
RSc0663	RS01568		0,39 0,04	-0,74 0,01				TRANSMEMBRANE COG1512, Beta-propeller domains of methanol dehydrogenase t	VI.C
RSc0664	RS01569			-0,69 0,01				LIPOPROTEIN	VI.C
RSc0665	RS01570			-0,73 0,00				TRANSMEMBRANE DIHYDRODIPICOLINATE SYNTHASE-RELATED PROTEIN	I.A.13
RSc0666	RS01571		0,58 0,03				hemL	GLUTAMATE-1-SEMIALDEHYDE 2,1-AMINOMUTASE	I.B.4
RSc0667	RS01572							RUBREDOXIN	I.G.1
RSc0668	RS01573		0,21 0,00	-0,47 0,02			pilG	TRANSCRIPTION REGULATOR TWITCHING MOTILITY TWO-COMPONENT RESP	IV.C
RSc0669	RS01574			-0,34 0,00			pilH	TRANSCRIPTION REGULATOR TWITCHING MOTILITY TWO-COMPONENT RESP	IV.C
RSc0670	RS01575			-0,43 0,01	0,68 0,03		pilI	TWITCHING MOTILITY PROTEIN	IV.C
RSc0671	RS01576		0,39 0,02	-0,38 0,02	0,37 0,02		pilJ	TRANSMEMBRANE TWITCHING MOTILITY PROTEIN	IV.C
RSc0672	RS01577			-0,36 0,01		-0,93 0,05		TRANSCRIPTION REGULATOR COMPOSITE TWO COMPONENT REGULATORY F	VI.A
RSc0673	RS01578							HYPOTHETICAL PROTEIN	VI.D
RSc0674	RS01579			-0,40 0,05			rsuA	RIBOSOMAL SMALL SUBUNIT PSEUDOURIDINE SYNTHASE A	II.B.6
RSc0675	RS01580			-0,64 0,02	-0,72 0,02			COG1678, Putative transcriptional regulator	VI.A
RSc0676	RS01581		0,54 0,00	-0,46 0,00				COG0816, Predicted endonuclease involved in recombination	II.B.8
RSc0677	RS01582					-1,45 0,00	pyrR	PYRIMIDINE OPERON REGULATORY PROTEIN PYRR	I.D.1

RSc0678	RS01583			0,77	0,00							pyrB	ASPARTATE CARBAMOYLTRANSFERASE (CATALYTIC CHAIN)	I.D.1
RSc0679	RS01584			0,58	0,00	-0,25	0,00			-0,59	0,03	pyrX	DIHYDROOROTASE-LIKE PROTEIN	I.D.1
RSc0680	RS01585	-0,31	0,04							-0,70	0,00	plsC	TRANSMEMBRANE 1-ACYL-SN-GLYCEROL-3-PHOSPHATE ACYLTRANSFERASE	II.B.1
RSc0681	RS01586			0,48	0,01							apaH	BIS(5'-NUCLEOSYL)-TETRAPHOSPHATASE (SYMMETRICAL)	I.F.1
RSc0682	RS01587			0,58	0,00	-0,26	0,00	0,77	0,00			rfbB	DTDP-GLUCOSE 4,6-DEHYDRATASE	I.F.10
RSc0683	RS01588			0,80	0,00			0,45	0,02	0,67	0,01	rfbD	OXIDOREDUCTASE DTDP-4-DEHYDRORHAMNOSE REDUCTASE	I.F.10
RSc0684	RS01589			0,50	0,04	-0,36	0,01	0,61	0,03			rfbA	GLUCOSE-1-PHOSPHATE THYMIDYLYLTRANSFERASE	I.F.10
RSc0685	RS01590			1,02	0,00	-0,37	0,00					rfbC	DTDP-4-DEHYDRORHAMNOSE 3,5-EPIMERASE	I.F.10
RSc0686	RS01591					-0,52	0,00						TRANSMEMBRANE LIPOPOLYSACCHARIDE O-SIDE CHAIN BIOSYNTHESIS PRO	II.B.2
RSc0687	RS01592			0,39	0,00	-0,46	0,04					rfbF	DTDP-RHAMNOSYL TRANSFERASE RFBF	II.B.2
RSc0688	RS01593			0,57	0,05								GLYCOSYL TRANSFERASE	II.B.2
RSc0689	RS01594					-0,25	0,02					wecA	TRANSMEMBRANE UNDECAPRENYL PHOSPHATE N-ACETYLGLUCOSAMINYLTI	II.B.2
RSc0690	RS01595			0,50	0,04	-0,39	0,00						TRANSMEMBRANE	VI.C
RSc0691	RS01596			0,48	0,02	-0,30	0,00						PHOSPHOMANNOMUTASE OR PHOSPHOGLUCOMUTASE	I.F.18
RSc0692	RS01597			0,67	0,00	-0,32	0,01					rfaC1	LIPOPOLYSACCHARIDE HEPTOSYLTRANSFERASE	II.B.2
RSc0693	RS01598							-1,04	0,02			kdtA	TRANSMEMBRANE 3-DEOXY-D-MANNO-OCTULOSONIC-ACID TRANSFERASE	II.B.2
RSc0694	RS01599					-0,70	0,01	-0,98	0,05	-0,99	0,00		TRANSMEMBRANE COG3187, Heat shock protein	II.B.7
RSc0695	RS01600												ABC TRANSPORTER PORIN (OMP)	IV.D
RSc0696	RS01601			0,81	0,00								COG0607, Rhodanese-related sulfurtransferase	I
RSc0697	RS01602			0,65	0,03			0,71	0,02			pcm	PROTEIN-L-ISOASPARTATE O-METHYLTRANSFERASE	II.B.7
RSc0698	RS01603			0,35	0,00	-0,64	0,01			1,02	0,00		TRANSCRIPTION REGULATOR	VI.A
RSc0699	RS01604					-0,48	0,01						OXIDOREDUCTASE COG0727: Predicted Fe-S-cluster oxidoreductase	I
RSc0700	RS01605					0,38	0,00						TRANSMEMBRANE	VI.C
RSc0701	RS01606					-0,33	0,02					bacA	TRANSMEMBRANE BACITRACIN RESISTANCE PROTEIN PUTATIVE UNDECAPRI	IV.G.2
RSc0702	RS01607	-0,45	0,05										OXIDOREDUCTASE COG1902, NADH:flavin oxidoreductases	I
RSc0703	RS01608			0,77	0,05								COG0220, Predicted S-adenosylmethionine-dependent methyltransferase	VI.C
RSc0704	RS01609			0,52	0,01								COG0388, Predicted amidohydrolase	VI.C
RSc0705	RS01610					0,76	0,00						TRANSMEMBRANE	VI.D
RSc0706	RS05147			0,32	0,03	-0,53	0,00						CONSERVED HYPOTHETICAL PROTEIN	VI.C
RSc0707	RS05146					-0,87	0,01						HYPOTHETICAL PROTEIN	VI.D
RSc0708	RS05145			0,78	0,01	-0,68	0,01					ubiD	3-OCTAPRENYL-4-HYDROXYBENZOATE CARBOXY-LYASE (POLYPRENYL P-HY	I.B.5
RSc0709	RS05144							1,02	0,00				TRANSMEMBRANE TRANSGLYCOSYLASE	VI.B
RSc0710	RS05143					0,54	0,01						AMINOTRANSFERASE	I.F.14
RSc0711	RS05142			0,49	0,02	-0,11	0,02	0,89	0,05			nusB	TRANSCRIPTION REGULATOR N UTILIZATION SUBSTANCE PROTEIN B (TRANS	II.B.6
RSc0712	RS05141											ribH	RIBOFLAVIN SYNTHASE (BETA CHAIN) : 6,7-DIMETHYL-8-RIBITYLLUMAZINE SY	I.B.7
RSc0713	RS05140			0,35	0,03	-0,34	0,01	-0,71	0,00			ribAB	RIBOFLAVIN BIOSYNTHESIS BIFUNCTIONAL PROTEIN : GTP CYCLOHYDROLAS	I.B.7
RSc0714	RS05139											ribE	RIBOFLAVIN SYNTHASE (ALPHA CHAIN)	I.B.7
RSc0715	RS05138											ribD	OXIDOREDUCTASE RIBOFLAVIN BIOSYNTHESIS BIFUNCTIONAL PROTEIN RIBD	I.B.7
RSc0716	RS05137					-0,78	0,01						TRANSMEMBRANE TYPE 4 FIMBRIAL PILIN RELATED PROTEIN	III.A.2
RSc0717	RS05136												SIGNAL PEPTIDE TYPE-4 FRIMBRIAL PILIN-RELATED PROTEIN	III.A.2
RSc0718	RS05135					-0,44	0,03						TRANSMEMBRANE TYPE 4 FIMBRIAL BIOGENESIS PROTEIN PILY1-RELATED PI	III.A.2
RSc0719	RS05134					0,94	0,00						SIGNAL PEPTIDE TYPE 4 FIMBRIAL BIOGENESIS PILX-RELATED PROTEIN	III.A.2
RSc0720	RS05133	-3,07	0,00	-1,35	0,00	1,81	0,00			1,45	0,03		TRANSMEMBRANE TYPE 4 FIMBRIAL BIOGENESIS PROTEIN PILW-RELATED PR	III.A.2
RSc0721	RS05132												TRANSMEMBRANE	VI.D
RSc0722	RS05131												TRANSMEMBRANE TYPE 4 FIMBRIAL PILIN RELATED PROTEIN	III.A.2
RSc0723	RS05130			0,38	0,02								TRANSMEMBRANE TYPE-4 FIMBRIAL PILIN-RELATED PROTEIN	III.A.2
RSc0724	RS05129					-0,55	0,01	1,64	0,05				SIGNAL PEPTIDE TYPE-4 FIMBRIAL BIOGENESIS PILY1-RELATED PROTEIN	III.A.2
RSc0725	RS05128					-0,40	0,00			-0,39	0,02		TRANSMEMBRANE TYPE 4 FIMBRIAL BIOGENESIS PROTEIN PILX-RELATED PR	III.A.2
RSc0726	RS05127			-0,61	0,00	-0,20	0,01	1,40	0,00				TRANSMEMBRANE TYPE 4 FIMBRIAL BIOGENESIS PROTEIN PILW RELATED PR	III.A.2
RSc0727	RS05126					-0,67	0,04						TRANSMEMBRANE TYPE-4 FIMBRIAL BIOGENESIS PILV-RELATED PROTEIN	III.A.2
RSc0728	RS05125					-0,67	0,00	0,72	0,04				COG1327, Predicted transcriptional regulator, consists of a Zn-ribbon and ATP-cone (	VI.A
RSc0729	RS05124					-0,96	0,00	1,12	0,01	0,99	0,02	glyA1	SERINE HYDROXYMETHYLTRANSFERASE	I.A.11
RSc0730	RS05123			0,43	0,04								OXIDOREDUCTASE	I
RSc0731	RS05122					-0,66	0,01						THIOESTERASE	I
RSc0732	RS05121					-0,56	0,01					tolQ	TRANSMEMBRANE TOLQ-RELATED TRANSPORT PROTEIN	IV.E
RSc0733	RS05120					-0,62	0,00					tolR	TRANSMEMBRANE TOLR-RELATED PROTEIN TRANSPORT	IV.E
RSc0734	RS05119					-0,74	0,00					tolA	TRANSMEMBRANE TOLA-RELATED TRANSPORT PROTEIN	IV.E
RSc0735	RS05118					-0,60	0,00	0,42	0,05			tolB	TOLB -RELATED TRANSPORT PROTEIN	IV.E
RSc0736	RS05117					-0,47	0,00					pal	PEPTIDOGLYCAN-ASSOCIATED LIPOPROTEIN PRECURSOR	III.B.3
RSc0737	RS05116					-0,30	0,00						TRANSMEMBRANE	VI.C
RSc0738	RS05115					-0,30	0,04						SIGNAL PEPTIDE COG0603, Predicted PP-loop superfamily ATPase	VI.C
RSc0739	RS05114			0,44	0,01								TRANSMEMBRANE	VI.C
RSc0740	RS05113			0,56	0,02					-0,69	0,02		TRANSMEMBRANE	VI.D
RSc0741	RS05112			0,45	0,05	-0,67	0,02	-0,82	0,00	0,99	0,00	che	TRANSCRIPTION REGULATOR CHEMOTAXIS RESPONSE REGULATOR PROTEII	IV.C
RSc0742	RS05111			0,68	0,01	-1,10	0,00					cheZ2	CHEMOTAXIS PROTEIN CHEZ	IV.C
RSc0743	RS05110			0,53	0,00	-0,39	0,01					aroG2	PHOSPHO-2-DEHYDRO-3-DEOXYHEPTONATE ALDOLASE, PHE-SENSITIVE (DA	I.A.5
RSc0744	RS05109					-0,51	0,00						DNA/RNA NON-SPECIFIC ENDONUCLEASE	II.A
RSc0745	RS05108												COG3613, Nucleoside 2-deoxyribosyltransferase	I
RSc0746	RS05107			0,66	0,01							tauD	OXIDOREDUCTASE ALPHA-KETOGLUTARATE-DEPENDENT TAURINE DIOXYGE	I.I.2
RSc0747	RS05106												CONSERVED HYPOTHETICAL PROTEIN	VI.C
RSc0748	RS05105					-0,36	0,03	-1,03	0,05	-0,69	0,00		ABC TRANSPORTER COG0390, ABC-type uncharacterized transport system, permea	IV.D
RSc0749	RS05104					-0,33	0,02						ABC TRANSPORTER ATP-BINDING PROTEIN	IV.D
RSc0750	RS05103					-1,20	0,00						TRANSMEMBRANE	VI.B
RSc0751	RS05102					-0,80	0,00						CONSERVED HYPOTHETICAL PROTEIN	VI.C
RSc0752	RS05101					-0,41	0,00						TRANSMEMBRANE	VI.C
RSc0753	RS05100												TRANSMEMBRANE	VI.C
RSc0754	RS05099					-0,27	0,02	-0,59	0,02				OXIDOREDUCTASE ANTIOXIDANT PROTEIN (PEROXIDASE)	IV.G.4
RSc0755	RS05098												TRANSMEMBRANE COG2252, Permeases	IV.D
RSc0756	RS05097			0,62	0,02							gcdH	OXIDOREDUCTASE GLUTARYL-CoA DEHYDROGENASE	I.I.3
RSc0757	RS05096												TRANSCRIPTION REGULATOR	VI.A
RSc0758	RS05095					0,34	0,01					kynA	OXIDOREDUCTASE Tryptophan 2,3-dioxygenase	I.I.3
RSc0759	RS05094					0,43	0,01					kynU	KYNURENINASE	I.I.3
RSc0760	RS05093											kynB	KYNURENINE FORMIDASE	I.I.3
RSc0761	RS05092					-0,77	0,00						TRANSCRIPTION REGULATOR	VI.A
RSc0762	RS05091			0,85	0,03	-0,43	0,02						CONSERVED HYPOTHETICAL PROTEIN	VI.C
RSc0763	RS05090			0,24	0,05								OXIDOREDUCTASE MONOOXYGENASE	I
RSc0764	RS05089	-0,54	0,04			-0,65	0,04					msrA	OXIDOREDUCTASE PEPTIDE METHIONINE SULFOXIDE REDUCTASE	IV.B
RSc0765	RS05088												CONSERVED HYPOTHETICAL PROTEIN	VI.C
RSc0766	RS05087					-0,80	0,00			4,32	0,01	cfa2	CYCLOPROPANE-FATTY-ACYL-PHOSPHOLIPID SYNTHASE	I.C
RSc0767	RS05086			0,75	0,05							pdxH1	OXIDOREDUCTASE PYRIDOXAMINE 5'-PHOSPHATE OXIDASE (PNP/PMP OXIDA	I.B.10
RSc0768	RS05151												HYPOTHETICAL PROTEIN	VI.D
RSc0769	RS05085			0,84	0,01								HYDROLASE GLYCOSIDASE	I.I.4
RSc0770	RS05084					-1,00	0,01						OXIDOREDUCTASE TRANSMEMBRANE DEHYDROGENASE (SMALL SUBUNIT)	I
RSc0771	RS05083												OXIDOREDUCTASE TRANSMEMBRANE DEHYDROGENASE (LARGE SUBUNIT)	I
RSc0772	RS05082												SIGNAL PEPTIDE OXIDOREDUCTASE DEHYDROGENASE (CYTOCHROME C SUI	I.G.1
RSc0773	RS05081											asmA	TRANSMEMBRANE COG2982, Uncharacterized protein involved in outer membrane I	VI.C
RSc0774	RS05080												TRANSMEMBRANE COG1179, Dinucleotide-utilizing enzyme involved in molybdopter	I.B
RSc0775	RS05079			0,80	0,00	0,32	0,02					katGb	OXIDOREDUCTASE PEROXIDASE/CATALASE (CATA PROTEIN)	IV.G.4
RSc0777	RS05077												COG2703, Hemerythrin	IV.D.5
RSc0778	RS05076					-0,28	0,05						COG3531, Predicted protein-disulfide isomerase	II.B.7
RSc0779	RS05075			0,82	0,01								THIOREDOXIN	I.B.6
RSc0780	RS05058					-0,57	0,03					fadD2	LONG-CHAIN-FATTY-ACID--CoA LIGASE	I.I.1
RSc0781	RS05057												SIGNAL PEPTIDE ANKYRIN REPEAT HARBORING PROTEIN	VI.C
RSc0782	RS05056					-0,78	0,01	-0,44	0,02				COG3703, Uncharacterized protein involved in cation transport	IV.D.4
RSc0783	RS05055					-0,94	0,05						TRANSMEMBRANE	VI.D
RSc0784	RS05054			0,48	0,01							fkpA	FKBP-TYPE PEPTIDYL-PROLYL CIS-TRANS ISOMERASE (PPIASE)(IMMUNOPHII	IV.B
RSc0785	RS05053			0,66	0,01								CONSERVED HYPOTHETICAL PROTEIN	VI.C
RSc0786	RS05051					-0,94	0,00						CONSERVED HYPOTHETICAL PROTEIN	VI.C
RSc0787	RS05050					-0,63	0,03						TRANSMEMBRANE	VI.C
RSc0788	RS05049							0,49	0,02				TRANSMEMBRANE	VI.C
RSc0789	RS05048			0,50	0,05					-0,25	0,04		CONSERVED HYPOTHETICAL PROTEIN	VI.C
RSc0790	RS05047			0,76	0,00								SIGNAL PEPTIDE	VI.C

RSc0791	RS05046					-0,35	0,00	0,84	0,00			glnS	GLUTAMINYL-tRNA SYNTHETASE	II.D.1
RSc0792	RS05045												CONSERVED HYPOTHETICAL PROTEIN	VI.C
RSc0793	RS05044			1,60	0,00	-0,60	0,00						TRANSMEMBRANE	VI.C
RSc0794	RS05043			1,60	0,02	-0,85	0,02						TRANSMEMBRANE	VI.C
RSc0795	RS05042			0,54	0,04	-0,34	0,01						TRANSMEMBRANE	VI.C
RSc0796	RS05041			0,68	0,04	-0,39	0,02						TRANSMEMBRANE COG1804, Predicted acyl-CoA transferases/carnitine dehydratas	I
RSc0797	RS05040			0,43	0,02					-0,77	0,03	alaS	ALANYL-TRNA SYNTHETASE	II.D.1
RSc0798	RS05039					-0,64	0,00						PURINE NUCLEOSIDE PERMEASE	IV.D.6
RSc0799	RS05038			0,40	0,02	-0,40	0,02						PURINE NUCLEOSIDE PERMEASE	IV.D.6
RSc0800	RS05037									-1,93	0,05		SIGNAL PEPTIDE FERRISIDEROPHORE RECEPTOR	IV.D.5
RSc0801	RS05036			0,70	0,02	-1,05	0,00						TRANSMEMBRANE LIPOPROTEIN	VI.C
RSc0802	RS05031												TRANSMEMBRANE COG0330, Membrane protease subunit, stomatin/prohibitin homo	II.A.4
RSc0803	RS05027	-0,62	0,02			-0,54	0,00						TRANSMEMBRANE COG1030, Membrane-bound serine protease (ClpP class)	II.A.4
RSc0804	RS05024					-0,62	0,00						TRANSCRIPTION REGULATOR	VI.A
RSc0805	RS05564			-0,59	0,00	0,88	0,00						CONSERVED HYPOTHETICAL PROTEIN	VI.C
RSc0806	RS05016												ALDOLASE	I
RSc0807	RS05015												TAUTOMERASE (4-OXALOCROTONATE TAUTOMERASE)	I.I.4
RSc0808	RS05014			0,55	0,02							ltaA	LOW SPECIFICITY L-THREONINE ALDOLASE	I.A.11
RSc0809	RS05013					-0,53	0,00						HYDROLASE	I.I
RSc0810	RS05012					-0,39	0,02	-0,71	0,01	-0,46	0,02	sirA	COG0425, Predicted redox protein, regulator of disulfide bond formation	II.B.7
RSc0811	RS05011											sorB	OXIDOREDUCTASE SULFITE:CYTOCHROME C OXIDOREDUCTASE (SUBUNIT B)	I.F.3
RSc0812	RS05010					1,40	0,00			2,45	0,00	sorAb	OXIDOREDUCTASE SULFITE:CYTOCHROME C OXIDOREDUCTASE (SUBUNIT A)	I.F.3
RSc0813	RS05009			0,51	0,00	0,49	0,00			0,73	0,02	sorAa	OXIDOREDUCTASE SULFITE:CYTOCHROME C OXIDOREDUCTASE (SUBUNIT A)	I.F.3
RSc0814	RS05008					-0,83	0,00						TRANSMEMBRANE COG1280, Threonine efflux protein	IV.D.2
RSc0815	RS05007					-1,18	0,00						TRANSMEMBRANE	VI.B
RSc0816	RS05006												TRANSCRIPTION REGULATOR COG1733, Transcriptional regulator	VI.A
RSc0817	RS05005					-0,48	0,00					gstD	GLUTATHIONE S-TRANSFERASE	II.B.7
RSc0818	RS05004												COG4305, Endoglucanase C-terminal domain/subunit and related proteins	II.A.3
RSc0819	RS05003					1,52	0,00						COG5485, Ester cyclase	I
RSc0820	RS05179			1,53	0,04								TRANSCRIPTION REGULATOR	VI.A
RSc0821	RS05178												NADPH NITROREDUCTASE	I
RSc0822	RS05171												CONSERVED HYPOTHETICAL PROTEIN	VI.B
RSc0823	RS05169					-0,89	0,00	-2,07	0,01				HYPOTHETICAL PROTEIN	VI.D
RSc0824	RS05166			-1,73	0,02	2,39	0,00	1,70	0,01				CONSERVED HYPOTHETICAL PROTEIN	VI.C
RSc0825	RS06018	-1,32	0,05	-2,16	0,00	3,98	0,00	2,18	0,00				HYPOTHETICAL PROTEIN	VI.D
RSc0826	RS05162			-1,73	0,00	3,60	0,00			-0,38	0,00	popP1	TYPE III EFFECTOR PROTEIN POPP1	VI.E
RSc0827	RS05161												CONSERVED HYPOTHETICAL PROTEIN	VI.C
RSc0830	RS05157												CONSERVED HYPOTHETICAL PROTEIN	VI.C
RSc0831	RS05156					-0,34	0,01	-2,37	0,02			secA2	Preprotein translocase subunit SecA (ATPase, RNA helicase) - related protein	IV.E.2
RSc0832	RS05155					-0,52	0,00						HYPOTHETICAL PROTEIN	VI.D
RSc0833	RS04994					-1,17	0,01						HYPOTHETICAL PROTEIN	VI.B
RSc0834	RS04993												HYPOTHETICAL PROTEIN	VI.B
RSc0835	RS04991												CONSERVED HYPOTHETICAL PROTEIN	VI.C
RSc0836	RS04990					-0,63	0,00						CONSERVED HYPOTHETICAL PROTEIN	VI.C
RSc0837	RS04989												CONSERVED HYPOTHETICAL PROTEIN	VI.C
RSc0838	RS04988					-1,01	0,04						HYPOTHETICAL PROTEIN	VI.D
RSc0839	RS04987					-0,70	0,00						BACTERIOPHAGE-RELATED PROTEIN	V.D
RSc0840	RS04985			-2,87	0,03								CONSERVED HYPOTHETICAL PROTEIN	VI.C
RSc0841	RS04984												CONSERVED HYPOTHETICAL PROTEIN	VI.C
RSc0842	RS05150					-1,04	0,00	-1,40	0,00	-1,99	0,01		HYPOTHETICAL PROTEIN	VI.D
RSc0844	RS04982												COG0863, DNA modification methylase	II.B.8
RSc0845	RS04981												COG0863, DNA modification methylase	II.B.8
RSc0846	RS04979												HYPOTHETICAL PROTEIN	VI.D
RSc0847	RS04978												HYPOTHETICAL PROTEIN	VI.B
RSc0848	RS04976	0,54	0,05										CONSERVED HYPOTHETICAL PROTEIN	VI.C
RSc0849	RS04974												CONSERVED HYPOTHETICAL PROTEIN	VI.C
RSc0850	RS04972					-0,56	0,00			0,90	0,02		CONSERVED HYPOTHETICAL PROTEIN	VI.C
RSc0851	RS04971					-0,51	0,03						HYPOTHETICAL PROTEIN	VI.D
RSc0852	RS04970			-1,88	0,03	0,60	0,03	0,47	0,01				BACTERIOPHAGE-RELATED PROTEIN	V.D
RSc0853	RS04968					-0,72	0,03						BACTERIOPHAGE-RELATED PROTEIN	V.D
RSc0854	RS04967												HYPOTHETICAL PROTEIN	VI.D
RSc0855	RS04966							0,55	0,01				HYPOTHETICAL PROTEIN	VI.D
RSc0856	RS04965												HYPOTHETICAL PROTEIN	VI.D
RSc0857	RS04964												BACTERIOPHAGE-RELATED PROTEIN	V.D
RSc0858	RS04963												BACTERIOPHAGE-RELATED PROTEIN	V.D
RSc0859	RS04962					-1,00	0,02						BACTERIOPHAGE-RELATED PROTEIN	V.D
RSc0860	RS04958					-1,14	0,01						BACTERIOPHAGE-RELATED PROTEIN	V.D
RSc0861	RS04956												HYPOTHETICAL PROTEIN	VI.D
RSc0862	RS04955												SIGNAL PEPTIDE	VI.D
RSc0863	RS04954												CONSERVED HYPOTHETICAL PROTEIN	VI.C
RSc0864	RS04952												CONSERVED HYPOTHETICAL PROTEIN	VI.C
RSc0865	RS04953												HYPOTHETICAL PROTEIN	VI.D
RSc0866	RS04951												HYPOTHETICAL PROTEIN	VI.B
RSc0867	RS04950					0,55	0,02			-0,56	0,05		TRANSCRIPTION REGULATOR HNS-LIKE PROTEIN	VI.A
RSc0868	RS04882			-3,39	0,00	4,28	0,00	2,87	0,00			popP2	TYPE III EFFECTOR PROTEIN POPP2	VI.E
RSc0869	RS04946												BACTERIOPHAGE CYTOSINE-SPECIFIC METHYLTRANSFERASE	II.B.8
RSc0870	RS04945												HYPOTHETICAL PROTEIN	VI.B
RSc0871	RS04877					-0,33	0,00	-0,57	0,05	-0,19	0,02		PLASMID STABILITY-LIKE PROTEIN	V.B
RSc0872	RS04874							-0,89	0,00	-0,51	0,01		CONSERVED HYPOTHETICAL PROTEIN	VI.C
RSc0873	RS04872												TRANSMEMBRANE BACTERIOPHAGE-RELATED PROTEIN	V.D
RSc0874	RS04870												TRANSMEMBRANE	VI.D
RSc0875	RS04868												CONSERVED HYPOTHETICAL PROTEIN	VI.C
RSc0876	RS04866												CONSERVED HYPOTHETICAL PROTEIN	VI.C
RSc0877	RS04865												HYPOTHETICAL PROTEIN	VI.D
RSc0878	RS04864												HYPOTHETICAL PROTEIN	VI.D
RSc0879	RS04863							-0,56	0,05				TRANSMEMBRANE	VI.B
RSc0880	RS04862					-0,42	0,03						CONSERVED HYPOTHETICAL PROTEIN	VI.C
RSc0881	RS04861												TRANSMEMBRANE	VI.C
RSc0882	RS04860												TRANSMEMBRANE	VI.C
RSc0883	RS04859												LYSOZYME (ENDOLYSIN)	II.A.4
RSc0884	RS04858												CONSERVED HYPOTHETICAL PROTEIN	VI.C
RSc0885	RS04857					-0,45	0,01						CONSERVED HYPOTHETICAL PROTEIN	VI.C
RSc0886	RS04856			1,11	0,00	-0,60	0,02			-1,97	0,00		CONSERVED HYPOTHETICAL PROTEIN	VI.C
RSc0887	RS04855												HEMAGGLUTININ-RELATED PROTEIN	VI.C
RSc0888	RS04850					-0,61	0,00						ACTIVATION/SECRETION PROTEIN	IV.E.2
RSc0889	RS04841												COG3677, Transposase and inactivated derivatives	V.A
RSc0890	RS04839					-1,50	0,01						BACTERIOPHAGE RELATED PROTEIN	V.D
RSc0891	RS04522							-1,26	0,00				SITE-SPECIFIC INTEGRASE/RECOMBINASE	V
RSc0892	RS04521												PHAGE-RELATED PROTEIN	V.D
RSc0893	RS04520			-1,35	0,00	3,00	0,00	2,22	0,00				TRANSPOSASE	V.A
RSc0894	RS05563	-4,45	0,00	-7,01	0,00	7,46	0,00	4,65	0,00				SIGNAL PEPTIDE	VI.C
RSc0895	RS04524	-1,47	0,00	-4,18	0,00	3,88	0,00	2,98	0,00	-1,36	0,00		HYPOTHETICAL PROTEIN	VI.B
RSc0896	RS04519					-0,55	0,01						TRANSMEMBRANE	VI.C
RSc0897	RS04518					-0,33	0,00						COG0546, Phosphatase	I
RSc0898	RS04517											ubiG	3-DEMETHYLUBIQUINONE-9 3-METHYLTRANSFERASE	I.B.5
RSc0899	RS04516			-0,46	0,03					0,80	0,01		TRANSMEMBRANE OUTER MEMBRANE LIPOPROTEIN	III.B.2
RSc0900	RS04515			0,37	0,00	-0,25	0,05	0,51	0,02				SIGNAL PEPTIDE COG2885, Outer membrane protein and related peptidoglycan-ass	III.B.2
RSc0901	RS04514	-0,38	0,05	0,56	0,01							gyrA	DNA GYRASE (SUBUNIT A)(TYPE II TOPOISOMERASE)	II.B.8
RSc0902	RS04513					-0,48	0,00						SIGNAL PEPTIDE	VI.C
RSc0903	RS04512	-0,24	0,03	0,30	0,00					-0,70	0,00	serC	PHOSPHOSERINE AMINOTRANSFERASE (PSAT)	I.A.10
RSc0904	RS04511			0,71	0,01	-0,38	0,00			-0,66	0,04	pheA	BIFUNCTIONAL PROTEIN: CHORISMATE MUTASE AND PREPHENATE DEHYDR/	I.F.14
RSc0905	RS04510			0,67	0,00							hisC2	HISTIDINOL-PHOSPHATE AMINOTRANSFERASE	I.A.4

RSc0906	RS04509		0,49 0,01	-0,59 0,00		-0,68 0,03	tyrA	OXIDOREDUCTASE PREPHENATE DEHYDROGENASE	I.A.7
RSc0907	RS04508		0,77 0,00	-0,29 0,01			aroA	3-PHOSPHOSHIKIMATE 1-CARBOXYVINYLTRANSFERASE (EPSP SYNTHASE)	I.A.5
RSc0908	RS04507		0,89 0,01	-0,28 0,04			cmk	CYTIDYLATE KINASE	I.D.1
RSc0909	RS04506		0,45 0,02	-0,30 0,01	0,80 0,00	-0,49 0,00	rpsA	30S RIBOSOMAL SUBUNIT PROTEIN S1	III.C.2
RSc0910	RS04505					-0,84 0,05	ihfB	INTEGRATION HOST FACTOR BETA-SUBUNIT (IHF-BETA)	V.D
RSc0911	RS04504			-0,42 0,00				TRANSMEMBRANE	VI.C
RSc0912	RS04503			-0,42 0,02		-1,03 0,02		TRANSMEMBRANE COG2956, Predicted N-acetylglucosaminyl transferase	II
RSc0913	RS04502			-0,73 0,01	-1,37 0,00	-0,50 0,01	ugd	OXIDOREDUCTASE UDP-GLUCOSE 6-DEHYDROGENASE (UDG PROTEIN)	III.A.1
RSc0914	RS04501			-0,63 0,04		-0,57 0,00	rfaE	ADP-HEPTOSE SYNTHASE	II.B.2
RSc0915	RS04500				-0,44 0,01	-0,72 0,00	rfaD	ADP-L-GLYCERO-D-MANNO-HEPTOSE-6-EPIMERASE	I.B
RSc0916	RS04499			-0,79 0,00	-0,53 0,04	-1,22 0,00		SIGNAL PEPTIDE	VI.C
RSc0917	RS04497						cysM	CYSTEINE SYNTHASE B (CSASE B)	I.A.9
RSc0918	RS04496		0,52 0,00				mltB	MEMBRANE-BOUND LYTIC MUREIN TRANSGLYCOSYLASE B	III.B.3
RSc0919	RS04494	0,88 0,03						COG0123, Deacetylase, including acetoin utilization protein	I
RSc0920	RS04495							ABC TRANSPORTER ATP-BINDING PROTEIN	IV.D
RSc0921	RS04492				-1,72 0,00	-2,06 0,00		ABC TRANSPORTER TRANSMEMBRANE PROTEIN	IV.D
RSc0922	RS04493		1,08 0,03		-1,56 0,00	-3,54 0,00		ABC TRANSPORTER COG1464, ABC-type metal ion transport system, periplasmic c	IV.D.5
RSc0923	RS04491				-0,56 0,00	-0,48 0,01	etfB	ELECTRON TRANSFER FLAVOPROTEIN (BETA-SUBUNIT)	I.G.1
RSc0924	RS04490		0,53 0,01	-0,49 0,01		-1,03 0,00	etfA	ELECTRON TRANSFER FLAVOPROTEIN (ALPHA-SUBUNIT)	I.G.1
RSc0925	RS04489							OXIDOREDUCTASE ACYL-CoA DEHYDROGENASE	I
RSc0926	RS04488	1,11 0,03					dadA	SIGNAL PEPTIDE OXIDOREDUCTASE INNER MEMBRANE-BOUND D-AMINO ACI	I.I.3
RSc0927	RS04487			-0,68 0,00			lrp	TRANSCRIPTION REGULATOR LEUCINE-RESPONSIVE REGULATORY DNA-BIN	I.E.1
RSc0928	RS04486			-0,41 0,03				COG1533, DNA repair photolyase	II.B.8
RSc0929	RS04485			-0,54 0,00				TRANSMEMBRANE	VI.C
RSc0930	RS04484			-0,53 0,00				TRANSMEMBRANE	VI.D
RSc0931	RS04483			-1,15 0,00		2,27 0,00		SIGNAL PEPTIDE	VI.D
RSc0932	RS04482		0,58 0,00	-1,37 0,00				OXIDOREDUCTASE COG2133, Glucose/sorbosone dehydrogenase	I.F
RSc0933	RS04481			-0,44 0,02			rpsP	30S RIBOSOMAL SUBUNIT PROTEIN S16 (DNA-BINDING CATION-DEPENDENT	III.C.2
RSc0934	RS04480			-0,46 0,00		-0,46 0,04	rimM	16S RRNA PROCESSING PROTEIN	III.C.1
RSc0935	RS04479			-0,45 0,03	0,78 0,00	-0,72 0,01	trmB	tRNA (GUANINE-N1)-METHYLTRANSFERASE	II.D.1
RSc0936	RS04478		0,63 0,04	-0,43 0,01		-0,71 0,00	rplS	50S RIBOSOMAL SUBUNIT PROTEIN L19	III.C.2
RSc0937	RS04477		0,82 0,03	-0,35 0,01				COG0494, NTP pyrophosphohydrolase including oxidative damage repair enzymes	VI.C
RSc0938	RS04475			-0,51 0,00				TRANSMEMBRANE COBALAMIN BIOSYNTHESIS COBD-RELATED PROTEIN	I.B.3
RSc0939	RS04472			-0,47 0,05	-0,97 0,01			CONSERVED HYPOTHETICAL PROTEIN	VI.C
RSc0940	RS04469		0,74 0,00	0,46 0,03				COG1162, Predicted GTPase	VI.C
RSc0941	RS04465		0,93 0,00					TRANSMEMBRANE INTEGRAL MEMBRANE PROTEASE	II.A.4
RSc0942	RS04464			-0,32 0,00		-1,02 0,00	orn	OLIGORIBONUCLEASE	II.A.2
RSc0943	RS04462		0,46 0,01				mog	MOLYBDOCHETALASE IN MOLYBDOPTERIN BIOSYNTHESIS	I.B.12
RSc0944	RS04461							CONSERVED HYPOTHETICAL PROTEIN	VI.C
RSc0945	RS04460						pmbA	PMBA PROTEIN (TLDE PROTEIN)	II.B.7
RSc0946	RS04449			-0,44 0,01			folA	OXIDOREDUCTASE DIHYDROFOLATE REDUCTASE	I.F
RSc0947	RS04447		1,21 0,01	-0,46 0,01	0,69 0,02		thyA	THYMIDYLATE SYNTHASE (TRANSLATION REGULATION REPRESSOR RNA-BIN	I.F.2
RSc0948	RS04442			-0,46 0,00				AMINO-ACID DEHYDRATASE	I.A
RSc0949	RS04438		1,09 0,01	-0,71 0,00				TRANSMEMBRANE COG0861, Membrane protein TerC	III.B.1
RSc0950	RS04435							CONSERVED HYPOTHETICAL PROTEIN	VI.C
RSc0951	RS04428							HYPOTHETICAL PROTEIN	VI.B
RSc0952	RS04426			-0,60 0,00	0,57 0,05	-1,25 0,02		ATP-DEPENDENT RNA HELICASE	II.B.6
RSc0953	RS04413			-1,26 0,00	-1,56 0,00			TRANSMEMBRANE	VI.D
RSc0954	RS04412				-1,04 0,01			HYPOTHETICAL PROTEIN	VI.D
RSc0955	RS04411			-0,65 0,00	-0,42 0,05	1,24 0,02		TRANSMEMBRANE	VI.D
RSc0956	RS04410	0,52 0,02		-0,58 0,02	-1,16 0,04			HYPOTHETICAL PROTEIN	VI.D
RSc0957	RS04409	-2,37 0,03		-1,46 0,00				HYPOTHETICAL PROTEIN	VI.D
RSc0958	RS04407			-1,41 0,00				VGR-RELATED PROTEIN	VI.C
RSc0959	RS04408			-1,38 0,00	-0,55 0,04			TRANSMEMBRANE	VI.C
RSc0960	RS04406			-1,29 0,00				CONSERVED HYPOTHETICAL PROTEIN	VI.C
RSc0961	RS04405		0,62 0,03	-0,76 0,02				CONSERVED HYPOTHETICAL PROTEIN	VI.C
RSc0962	RS04402			-1,40 0,00	-1,79 0,00	-3,16 0,00		TRANSCRIPTION REGULATOR BACTERIOPHAGE DNA-BINDING PROTEIN	V.D
RSc0963	RS04401			-0,88 0,00	-0,61 0,04	-0,83 0,01		HYPOTHETICAL PROTEIN	VI.D
RSc0964	RS04403			-1,58 0,02				TRANSCRIPTION REGULATOR BACTERIOPHAGE TRANSCRIPTIONAL ACTIVAT	V.D
RSc0965	RS04404		0,29 0,01	-0,70 0,00	-1,42 0,01			HYPOTHETICAL PROTEIN	VI.D
RSc0966	RS04400			-0,52 0,00				CONSERVED HYPOTHETICAL PROTEIN	VI.C
RSc0967	RS04399							CONSERVED HYPOTHETICAL PROTEIN	VI.C
RSc0968	RS04398			-0,45 0,03	-1,07 0,01			CP4-LIKE INTEGRASE	V.A
RSc0969	RS04395							TRANSMEMBRANE	VI.C
RSc0970	RS04396		-1,24 0,02	1,14 0,00				SIGNAL PEPTIDE	VI.D
RSc0971	RS04393				-0,47 0,01			HYPOTHETICAL PROTEIN	VI.B
RSc0972	RS04392							CONSERVED HYPOTHETICAL PROTEIN	VI.C
RSc0973	RS04319			-0,77 0,02				ABC TRANSPORTER AMINO-ACID-BINDING PERIPLASMIC PROTEIN (PBP)	IV.D.2
RSc0974	RS04318			-1,67 0,00	-1,81 0,00	0,75 0,00		TRANSMEMBRANE	VI.D
RSc0975	RS04385							ABC TRANSPORTER ATP-BINDING PROTEIN	IV.D
RSc0976	RS04316		0,91 0,00				parE	DNA TOPOISOMERASE IV (SUBUNIT B)	II.B.8
RSc0977	RS04315			-0,63 0,01				SIGNAL PEPTIDE TRANSGLYCOSYLASE	VI.B
RSc0978	RS04314		0,89 0,02				parC	DNA TOPOISOMERASE IV (SUBUNIT A)	II.B.8
RSc0979	RS04313		1,07 0,03					TRANSMEMBRANE	VI.C
RSc0980	RS04312		1,51 0,01					TRANSMEMBRANE	VI.C
RSc0981	RS04311			-0,68 0,02			lldP	TRANSMEMBRANE L-LACTATE PERMEASE	IV.D.3
RSc0982	RS04310			-0,66 0,00	-0,91 0,05			TRANSMEMBRANE	VI.C
RSc0983	RS04309			-0,58 0,00				CONSERVED HYPOTHETICAL PROTEIN	VI.C
RSc0984	RS04308						gstE	GLUTATHIONE S-TRANSFERASE	II.B.7
RSc0985	RS04307							TRANSCRIPTION REGULATOR COG1167, Transcriptional regulators containing a D	VI.A
RSc0986	RS04306		0,39 0,02					COG0251, Translation initiation inhibitor	II.B.7
RSc0987	RS04305			-0,45 0,00				TRANSMEMBRANE	VI.C
RSc0988	RS04304							COG2872, Predicted metal-dependent hydrolases related to alanyl-tRNA synthetase I	I.I
RSc0989	RS04303			-0,58 0,01				TRANSCRIPTION REGULATOR	VI.A
RSc0990	RS04302		0,68 0,01		0,62 0,00	-0,86 0,00	hptG	CHAPERONE (HEAT SHOCK PROTEIN HTPG)	IV.B
RSc0991	RS04301						mug	COG3663, G:T/U mismatch-specific DNA glycosylase	II.B.8
RSc0992	RS04300						gstF	TRANSMEMBRANE GLUTATHIONE S-TRANSFERASE WITH COG3832 (Uncharact	II.B.7
RSc0993	RS04299			-0,59 0,00	-1,19 0,05			TRANSCRIPTION REGULATOR TRANSCRIPTIONAL REGULATORY DNA-BINDING	VI.A
RSc0994	RS04298		0,63 0,03					TRANSMEMBRANE	VI.C
RSc0995	RS04297							COG0421, Spermidine synthase	I.F.15
RSc0996	RS04296				0,86 0,01			CONSERVED HYPOTHETICAL PROTEIN	VI.C
RSc0997	RS04295							COG1670, Acetyltransferase	I
RSc0998	RS04294			-0,34 0,00				COG0714, MoxR-like ATPase	VI.C
RSc0999	RS04293				0,56 0,00	1,28 0,00		SIGNAL PEPTIDE PERIPLASMIC CYTOCHROME TYPE-C OXIDOREDUCTASE	I.G.1
RSc1000	RS04292			0,15 0,01	0,58 0,01	1,12 0,00		SIGNAL PEPTIDE PERIPLASMIC CYTOCHROME TYPE-C OXIDOREDUCTASE	I.G.1
RSc1001	RS04291							CONSERVED HYPOTHETICAL PROTEIN	VI.C
RSc1002	RS04290		-0,43 0,03	0,86 0,00				TRANSMEMBRANE	VI.C
RSc1003	RS04289		0,84 0,01					CARBOXYPEPTIDASE	II.A.4
RSc1004	RS04288		0,46 0,00	-0,37 0,00				COG0778, Nitroreductase	I
RSc1005	RS04287			-0,41 0,00	-0,44 0,05		nth	ENDONUCLEASE III	II.A.1
RSc1006	RS04286							FERREDOXIN [4Fe-4S]	I.G.1
RSc1007	RS04285		0,51 0,02		-0,69 0,01			TRANSCRIPTION REGULATOR	VI.A
RSc1008	RS04284			-0,43 0,01		-0,85 0,00	phaZ	POLY[D(-)-3-HYDROXYBUTYRATE] DEPOLYMERASE	II.A
RSc1010	RS04280		0,58 0,00			-0,64 0,03	tyrB	AROMATIC-AMINO-ACID AMINOTRANSFERASE	I.A
RSc1011	RS04277						uvrB	EXCINUCLEASE ABC SUBUNIT B (SOS RESPONSE EXCISION NUCLEASE DNA	II.B.8
RSc1012	RS04272				-0,73 0,03			TRANSMEMBRANE SITE-SPECIFIC RECOMBINASE	V.A
RSc1013	RS04269		0,83 0,03		-3,20 0,00			RIBOKINASE	I
RSc1014	RS04266		0,54 0,00	-0,54 0,01				TRANSCRIPTION REGULATOR TRANSCRIPTIONAL REGULATORY DNA-BINDING	VI.A
RSc1015	RS04264							ABC TRANSPORTER RIBOSE ABC TRANSPORTER, PERMEASE PROTEIN	IV.D.3
RSc1016	RS04262		0,24 0,02		-1,22 0,02			ABC TRANSPORTER RIBOSE ABC TRANSPORTER, ATP-BINDING PROTEIN	IV.D.3
RSc1017	RS04260			-0,27 0,03	-0,56 0,02			ABC TRANSPORTER RIBOSE ABC TRANSPORTER, PERIPLASMIC RIBOSE-BIND	IV.D.3
RSc1018	RS04257			-0,53 0,00	-1,08 0,00	-1,43 0,00		TRANSCRIPTION REGULATOR COG1959, Transcriptional regulator	VI.A

RSc1019	RS04253											iscS	Cysteine sulfinate desulfinase / cysteine desulfurase	I
RSc1020	RS04249							-0,66	0,01	-0,85	0,00	iscU	IRON-BINDING PROTEIN	I.H
RSc1021	RS04236									-1,15	0,02	iscA	CONSERVED HYPOTHETICAL PROTEIN	VI.C
RSc1022	RS04245									-0,99	0,02		CONSERVED HYPOTHETICAL PROTEIN	VI.C
RSc1023	RS04234									-2,07	0,00	hscB	CHAPERONE PROTEIN	IV.B
RSc1024	RS04231									-2,22	0,00	hscA	CHAPERONE PROTEIN	IV.B
RSc1025	RS04229									-2,04	0,00	fdx	FERREDOXIN [2FE-2S]	I.G.1
RSc1026	RS04227							-1,41	0,01	-2,09	0,00		CONSERVED HYPOTHETICAL PROTEIN	VI.C
RSc1027	RS04225			-1,37	0,00	0,71	0,00	0,94	0,00	2,60	0,00		TRANSMEMBRANE	VI.C
RSc1028	RS04223							0,96	0,01	-0,77	0,00	lysS	LYSYL-TRNA SYNTHETASE	II.D.1
RSc1029	RS04221							-0,42	0,04	-2,12	0,00		OXIDOREDUCTASE SHORT-CHAIN ALCOHOL DEHYDROGENASE	I
RSc1030	RS05824					-0,44	0,01			-0,67	0,00	prfB	PEPTIDE CHAIN RELEASE FACTOR 2 (RF-2)	II.B.7
RSc1031	RS04219			0,57	0,03	-0,33	0,03					pncB	NICOTINATE PHOSPHORIBOSYLTRANSFERASE (NAPRTASE)	I.B
RSc1032	RS04218			0,64	0,02			-0,48	0,00				TRANSMEMBRANE	VI.C
RSc1033	RS04217					-0,46	0,01						TRANSMEMBRANE COG1055, Na+/H+ antiporter NhaD and related arsenite permea	IV.D
RSc1034	RS04216					-0,48	0,02	-0,40	0,00				OXIDOREDUCTASE COG1052, Lactate dehydrogenase and related dehydrogenases	I
RSc1035	RS04215							-1,37	0,03				CONSERVED HYPOTHETICAL PROTEIN	VI.C
RSc1036	RS04214			0,49	0,00	-0,64	0,00	-1,21	0,04				TRANSMEMBRANE	VI.C
RSc1037	RS04213			0,70	0,05							moeA1	MOLYBDOPTERIN BIOSYNTHESIS MOEA PROTEIN	I.B.12
RSc1038	RS04212					-0,17	0,05					mobA	MOLYBDOPTERIN-GUANINE DINUCLEOTIDE BIOSYNTHESIS PROTEIN A (PROT	I.B.12
RSc1039	RS04211							0,74	0,01			moaA	MOLYBDENUM COFACTOR BIOSYNTHESIS PROTEIN A	I.B.12
RSc1040	RS04210									-0,71	0,00	rne	RIBONUCLEASE E (RNASE E)	II.A.2
RSc1041	RS04757					-0,22	0,02					rluC	RIBOSOMAL LARGE SUBUNIT PSEUDOURIDINE SYNTHASE C	III.C.1
RSc1042	RS04208												PHOSPHOGLYCOLATE PHOSPHATASE	I.I.4
RSc1043	RS04207			0,44	0,03	-0,29	0,02						COG2146, Ferredoxin subunits of nitrite reductase and ring-hydroxylating dioxygenas	I
RSc1044	RS04206					-0,51	0,04			-0,38	0,05		TRANSMEMBRANE PROTEASE	II.A.4
RSc1045	RS04205			0,95	0,00								TRANSMEMBRANE COG0313, Methyltransferase	VI.C
RSc1046	RS04204			1,22	0,02	-0,47	0,01						COG0424, Nucleotide-binding protein implicated in inhibition of septum formation	IV.A
RSc1047	RS04197					-0,53	0,00			-0,59	0,00		COG1399, Predicted metal-binding, possibly nucleic acid-binding protein	VI.C
RSc1048	RS04194									-1,40	0,04	rpmF	50S RIBOSOMAL SUBUNIT PROTEIN L32	III.C.2
RSc1049	RS04184					-0,44	0,05			-1,11	0,00	plsX	FATTY ACID/PHOSPHOLIPID BIOSYNTHESIS ENZYME	I
RSc1050	RS04180			0,63	0,02					-0,69	0,00	fabH	3-OXOACYL-[ACYL-CARRIER-PROTEIN] SYNTHASE III	I.B.1
RSc1051	RS04178					0,70	0,00	2,67	0,00	-1,36	0,00	fabD	MALONYL COA-[ACYL-CARRIER-PROTEIN] TRANSACYLASE	I.B.1
RSc1052	RS04176			0,37	0,05					-0,76	0,01	fabG1	OXIDOREDUCTASE 3-OXOACYL-[ACYL-CARRIER-PROTEIN] REDUCTASE	I.B.1
RSc1053	RS04175											acpP	ACYL CARRIER PROTEIN	I.B.1
RSc1054	RS04165							0,58	0,03			fabF1	3-OXOACYL-[ACYL-CARRIER-PROTEIN] SYNTHASE II	I.B.1
RSc1055	RS04163					-0,78	0,00	-0,65	0,00			rpoE1	TRANSCRIPTION REGULATOR RNA POLYMERASE SIGMA-E FACTOR (SIGMA-24	II.B.6
RSc1056	RS04161					-0,80	0,02	-0,72	0,00			rseA	TRANSCRIPTION REGULATOR TRANSMEMBRANE SIGMA-E FACTOR NEGATIVE	II.B.6
RSc1057	RS04159					-0,59	0,01					rseB	TRANSCRIPTION REGULATOR SIGMA-E FACTOR REGULATORY PROTEIN (NEG	II.B.6
RSc1058	RS04156			0,34	0,01	-0,49	0,00			-0,52	0,00		SIGNAL PEPTIDE PERIPLASMIC PROTEASE	II.A.4
RSc1059	RS04149			0,32	0,05	-0,48	0,00			-0,81	0,05		CONSERVED HYPOTHETICAL PROTEIN	VI.C
RSc1060	RS04148			0,42	0,05	-0,41	0,02	-0,83	0,03	-1,13	0,01	lepA	GTP-BINDING ELONGATION FACTOR	II.B.7
RSc1061	RS04147			0,44	0,05	-0,38	0,03	-0,56	0,01			lepB	TRANSMEMBRANE SIGNAL PEPTIDASE I (SPASE I)	II.B.7
RSc1062	RS04145			0,36	0,01	-0,49	0,00			-0,89	0,01		TRANSMEMBRANE	VI.C
RSc1063	RS04144					-0,39	0,03			-0,62	0,00	rnc	RIBONUCLEASE III (RNASE III)	II.A.2
RSc1064	RS04143									-1,28	0,01	era	GTP-BINDING PROTEIN	IV.A
RSc1065	RS04139			0,45	0,01					-0,92	0,00	recO	DNA REPAIR PROTEIN RECO (RECOMBINATION PROTEIN O)	II.B.8
RSc1066	RS04138									-0,72	0,00	pdxJ	PYRIDOXAL PHOSPHATE BIOSYNTHETIC PROTEIN	I.B
RSc1067	RS04136			0,34	0,01	-0,43	0,00			-1,23	0,00	acpS	HOLO-[ACYL-CARRIER-PROTEIN] SYNTHASE	I.B.1
RSc1068	RS04135												HEXOSAMINIDASE	I.I.4
RSc1069	RS04133					-0,41	0,02			-0,55	0,00	efp	ELONGATION FACTOR P	II.B.7
RSc1070	RS04117												CONSERVED HYPOTHETICAL PROTEIN	VI.C
RSc1071	RS04116					-0,58	0,04					uvrC	EXCINUCLEASE ABC SUBUNIT C (SOS RESPONSE DNA REPAIR PROTEIN)	II.A.1
RSc1072	RS04115					-0,35	0,04					pgsA	TRANSMEMBRANE CDP-DIACYLGLYCEROL--GLYCEROL-3-PHOSPHATE 3-PHOS	I.B.1
RSc1073	RS04114					-1,02	0,00						TRANSCRIPTION REGULATOR TWO-COMPONENT SYSTEM RESPONSE REGUL	VI.A
RSc1074	RS04113					-0,76	0,00						SIGNAL PEPTIDE	VI.C
RSc1075	RS04112					-0,72	0,00	-1,68	0,03				TRANSCRIPTION REGULATOR TWO-COMPONENT SYSTEM SENSOR HISTIDINE	VI.A
RSc1076	RS04111			1,38	0,01	-1,25	0,00						TRANSCRIPTION REGULATOR TWO-COMPONENT SYSTEM RESPONSE REGUL	VI.A
RSc1077	RS04110					-1,18	0,00						TRANSMEMBRANE	VI.C
RSc1078	RS04109					-0,60	0,00	-1,23	0,03				TRANSCRIPTION REGULATOR TRANSCRIPTIONAL REGULATORY DNA-BINDING	VI.A
RSc1079	RS04108			0,56	0,00	-0,76	0,00					gudD1	GLUCARATE DEHYDRATASE	I.I.4
RSc1080	RS04107					-0,82	0,01					exuT2	TRANSMEMBRANE HEXURONATE TRANSPORTER	IV.D
RSc1081	RS04106					-0,96	0,00						GLUCOSIDASE	I
RSc1082	RS04105					-0,73	0,00						OUTER MEMBRANE PORIN	III.B.2
RSc1083	RS04104			-2,07	0,00	1,71	0,00						COG2017, GALACTOSE MUTAROTASE AND RELATED ENZYMES	I
RSc1084	RS04103												TRANSMEMBRANE COG3203, Outer membrane protein (porin)	III.B.2
RSc1085	RS04102											nagL	GLUTATHIONE S-TRANSFERASE	I.I
RSc1086	RS04101			-1,36	0,05							nagK	ISOMERASE-DECARBOXYLASE HOMOLOG	I.I
RSc1087	RS04100			0,68	0,00							nagI	OXIDOREDUCTASE GENTISATE 1,2-DIOXYGENASE	I.I
RSc1088	RS04099											nagAb	OXIDOREDUCTASE Ferredoxin subunit of a ring-hydroxylating dioxygenase	I.I
RSc1089	RS04098											nagH	OXIDOREDUCTASE SALICYLATE-5-HYDROXYLASE SMALL OXYGENASE COMP(	I.I
RSc1090	RS04097											nagG	OXIDOREDUCTASE SALICYLATE-5-HYDROXYLASE LARGE OXYGENASE COMP(	I.I
RSc1091	RS04096											nagAa	OXIDOREDUCTASE FERREDOXIN OXIDOREDUCTASE	I.I
RSc1092	RS04095					-0,82	0,00						TRANSCRIPTION REGULATOR TRANSCRIPTIONAL REGULATORY DNA-BINDING	VI.A
RSc1093	RS04094											pcaK	TRANSMEMBRANE 4-HYDROXYBENZOATE TRANSPORTER	IV.D.3
RSc1094	RS04084							-1,06	0,02				TRANSCRIPTION REGULATOR TRANSCRIPTIONAL REGULATORY DNA-BINDING	VI.A
RSc1095	RS04073					-0,90	0,00						TRANSMEMBRANE COG0679, Predicted permease	IV.D
RSc1096	RS04070					-0,71	0,00					aspC	ASPARTATE AMINOTRANSFERASE	I.A.15
RSc1097	RS04067			0,34	0,04	-0,51	0,01	0,40	0,04				TRANSCRIPTION REGULATOR	VI.A
RSc1098	RS04066							-1,11	0,04				TRANSCRIPTION REGULATOR TRANSCRIPTIONAL REGULATORY DNA-BINDING	VI.A
RSc1099	RS04061					-0,58	0,03					sda2a	INACTIVATED L-SERINE AMONYA-LYASE, L-SERINE DEHYDRATASE 2 (L-SERIN	I.I.3
RSc1100	RS04059					-0,39	0,00					sda2b	INACTIVATED L-SERINE AMONYA-LYASE, L-SERINE DEHYDRATASE 2 (L-SERIN	I.I.3
RSc1101	RS04056											soxB2	OXIDOREDUCTASE SARCOSINE OXIDASE (BETA SUBUNIT)	I.I
RSc1102	RS04055											soxD2	OXIDOREDUCTASE SARCOSINE OXIDASE (DELTA SUBUNIT)	I.I
RSc1103	RS04054											soxA2	OXIDOREDUCTASE SARCOSINE OXIDASE (ALPHA SUBUNIT)	I.I
RSc1104	RS04053							-1,21	0,02			soxG2	OXIDOREDUCTASE SARCOSINE OXIDASE (GAMMA SUBUNIT)	I.I
RSc1105	RS04050											folB2	DIHYDRONEOPTERIN ALDOLASE	I.B.14
RSc1106	RS04039					-0,33	0,05					purU3	FORMYLTETRAHYDROFOLATE DEFORMYLASE	I.D.2
RSc1107	RS04032												TRANSMEMBRANE PSEUDOGENE (ABC TRANSPORTER N-TERMINAL FRAGMEI	VI.D
RSc1108	RS04035					-0,55	0,00						TRANSCRIPTION REGULATOR TRANSCRIPTIONAL REGULATORY DNA-BINDING	VI.A
RSc1109	RS04024			-0,66	0,05			-0,66	0,04				PSEUDOGENE (DEHYDROGENASE FRAGMENT)	VI.D
RSc1110	RS04021	-1,54	0,01			-0,82	0,00						TRANSCRIPTION REGULATOR TRANSCRIPTIONAL REGULATORY DNA-BINDING	VI.A
RSc1111	RS04015												TRANSMEMBRANE	VI.C
RSc1112	RS04011					-0,61	0,00						TRANSCRIPTION REGULATOR	VI.A
RSc1113	RS04007					-1,48	0,00			1,20	0,00		CONSERVED HYPOTHETICAL PROTEIN	VI.C
RSc1114	RS04006							-0,79	0,01			fdxA	FERREDOXIN	I.G.1
RSc1115	RS05755											recJ	SINGLE-STRANDED-DNA-SPECIFIC EXONUCLEASE	II.A.1
RSc1116	RS05756			0,56	0,03	-0,49	0,00						SIGNAL PEPTIDE HYPOTHETICAL PROTEIN	VI.B
RSc1117	RS05757					-0,47	0,00	0,54	0,02				TRANSMEMBRANE LIPOPROTEIN RELEASING SYSTEM PROTEIN	IV.E.2
RSc1118	RS05758							0,55	0,02			lolD	ABC TRANSPORTER LIPOPROTEIN RELEASING SYSTEM ATP-BINDING PROTEI	IV.E.2
RSc1119	RS04635					-0,44	0,00			-1,14	0,00		DEOXYRIBONUCLEASE	II.A.1
RSc1120	RS04634												TRANSMEMBRANE COG2333, Predicted hydrolase (metallo-beta-lactamase superfa	I.I
RSc1121	RS04633					-0,47	0,00						CONSERVED HYPOTHETICAL PROTEIN	VI.C
RSc1122	RS04632					-0,58	0,02			0,48	0,05		CONSERVED HYPOTHETICAL PROTEIN	VI.C
RSc1123	RS04630					-0,33	0,00						COG1305, Transglutaminase-like enzyme, putative cysteine protease	I
RSc1124	RS04629					-0,60	0,05						COG3484, Predicted proteasome-type protease	II.A.4
RSc1125	RS04628					-0,49	0,03						COG0596, Hydrolase or acyltransferase (alpha/beta hydrolase superfamily)	I
RSc1126	RS04627			0,61	0,01					-0,29	0,04	pyrG	CTP SYNTHASE	I.D.1
RSc1127	RS04626			0,88	0,00					-0,48	0,00	kdsA	2-DEHYDRO-3-DEOXYPHOSPHOOCTONATE ALDOLASE	III.A.1
RSc1128	RS04625			0,66	0,02	-0,32	0,01						CONSERVED HYPOTHETICAL PROTEIN	VI.C
RSc1129	RS04624			0,78	0,00	-0,26	0,01			-0,32	0,04	eno	ENOLASE (2-PHOSPHOGLYCERATE DEHYDRATASE)	I.H.8
RSc1130	RS04623			0,35	0,01	-0,17	0,04						TRANSMEMBRANE COG2919, Septum formation initiator	IV.A

RSc1131	RS04622			-0,53 0,00				TRANSMEMBRANE	VI.C
RSc1132	RS04621		0,69 0,00			-0,62 0,00		REDOX REGULATED MOLECULAR CHAPERONE HEAT-SHOCK-LIKE PROTEIN	IV.B
RSc1133	RS04620							COG0663, Carbonic anhydrase/acetyltransferase	I
RSc1134	RS04619							CONSERVED HYPOTHETICAL PROTEIN	VI.C
RSc1135	RS04618			-0,98 0,00	-1,24 0,01			COG0596, Hydrolase or acyltransferase (alpha/beta hydrolase superfamily)	I
RSc1136	RS04617				-0,83 0,00			CONSERVED HYPOTHETICAL PROTEIN	VI.C
RSc1137	RS04616			0,76 0,02				CONSERVED HYPOTHETICAL PROTEIN	VI.C
RSc1138	RS04615			-0,33 0,01	-0,61 0,04			OXIDOREDUCTASE COG1748: Saccharopine dehydrogenase and related proteins	I
RSc1139	RS04614							OXIDOREDUCTASE THIOREDOXIN REDUCTASE	I.F.2
RSc1140	RS04613			-0,54 0,00				COG0613, Predicted metal-dependent phosphoesterase	I
RSc1141	RS04612							COG0009, Translation factor	II.B.7
RSc1142	RS04611		0,52 0,01			-1,13 0,00		TRANSMEMBRANE COG1994, Zn-dependent protease	II.A.4
RSc1143	RS04610		0,63 0,03	-0,30 0,00			trpS	TRYPTOPHANYL-TRNA SYNTHETASE (SYW PROTEIN)	II.D.1
RSc1144	RS04609				-0,92 0,00	-0,61 0,00		CONSERVED HYPOTHETICAL PROTEIN	VI.C
RSc1145	RS04756			-0,46 0,00		-0,19 0,00	dapA	DIHYDRODIPICOLINATE SYNTHASE	I.A.13
RSc1146	RS04607			-0,48 0,04	-0,64 0,03	-0,74 0,00		TRANSMEMBRANE	VI.C
RSc1147	RS04606			-0,49 0,01		-0,78 0,00		COG1235, Metal-dependent hydrolase of the beta-lactamase superfamily I	I.I
RSc1148	RS04604		0,66 0,01					CONSERVED HYPOTHETICAL PROTEIN	VI.C
RSc1149	RS04602			-0,64 0,00	-0,59 0,01		slyD	FKBP-TYPE PEPTIDYL-PROLYL CIS- TRANS ISOMERASE (ROTAMASE)	IV.B
RSc1150	RS04601							TRANSMEMBRANE	VI.C
RSc1151	RS04600				0,42 0,04		mutS	DNA MISMATCH REPAIR PROTEIN	II.B.8
RSc1152	RS04599							TRANSCRIPTION REGULATOR	VI.A
RSc1153	RS04598							TRANSMEMBRANE TRANSMEMBRANE EFFLUX PROTEIN	IV.D
RSc1154	RS04597			-0,44 0,05				OXIDOREDUCTASE COG0656, Aldo/keto reductases, related to diketogulonate reduc	I
RSc1155	RS04596		0,33 0,05					TRANSMEMBRANE METHYL-ACCEPTING CHEMOTAXIS TRANSDUCER PROTEI	IV.C
RSc1156	RS04595							TRANSMEMBRANE METHYL-ACCEPTING CHEMOTAXIS TRANSDUCER PROTEI	IV.C
RSc1157	RS04594		-0,55 0,01	1,25 0,00				CONSERVED HYPOTHETICAL PROTEIN	VI.C
RSc1158	RS04593			-1,93 0,00				COG0518, GMP synthase - Glutamine amidotransferase domain	I.D
RSc1159	RS04592				-0,83 0,01			TRANSCRIPTION REGULATOR	VI.A
RSc1160	RS04755			-0,61 0,03	-0,28 0,05		suhB	INOSITOL MONOPHOSPHATASE (EXTRAGENIC SUPPRESSOR PROTEIN)	II.B.6
RSc1161	RS04590				-0,56 0,00			TRNA/RRNA METHYLTRANSFERASE	II.B.6
RSc1162	RS04589						cysE2	O-ACETYLSERINE SYNTHASE (SERINE ACETYLTRANSFERASE)	I.A.9
RSc1163	RS04585						lpxH	UDP-2,3-DIACYLGLUCOSAMINE HYDROLASE	II.B.2
RSc1164	RS04581		0,45 0,01		-0,92 0,00		ppiB	PEPTIDYL-PROLYL CIS-TRANS ISOMERASE B (PPIASE B) (ROTAMASE B)(CYPE	IV.B
RSc1165	RS04579		0,35 0,02				ppiA	SIGNAL PEPTIDE PEPTIDYL-PROLYL CIS-TRANS ISOMERASE A (PPIASE A)(ROT	IV.B
RSc1166	RS04578			-0,50 0,00	0,39 0,02			SIGNAL PEPTIDE TPR DOMAIN PROTEIN	VI.B
RSc1167	RS04577				0,53 0,04		cysS	CYSTEINYL-TRNA SYNTHETASE (SYC PROTEIN)	II.D.1
RSc1168	RS04576							COG0122, 3-methyladenine DNA glycosylase/8-oxoguanine DNA glycosylase	II.B.8
RSc1169	RS04560		0,48 0,00		0,69 0,01		accA	ACETYL-COENZYME A CARBOXYLASE CARBOXYL TRANSFERASE (ALPHA SUE	I.B.1
RSc1170	RS04559			-0,68 0,04			mesJ	CELL CYCLE PROTEIN	IV.A
RSc1171	RS04558		0,72 0,00	-0,31 0,02	-0,37 0,05	-2,51 0,00	lysC	ASPARTATE KINASE	I.A.13
RSc1172	RS04754					0,89 0,00	fabI	OXIDOREDUCTASE ENOYL-[ACYL-CARRIER-PROTEIN] REDUCTASE [NADH]	I.B.1
RSc1173	RS04555							ABC TRANSPORTER SUBSTRATE-BINDING PERIPLASMIC PROTEIN (PBP)	IV.D
RSc1174	RS04551			-0,50 0,00				ABC TRANSPORTER TRANSMEMBRANE PROTEIN	IV.D
RSc1175	RS04550				0,49 0,04			ABC TRANSPORTER TRANSMEMBRANE PROTEIN	IV.D
RSc1176	RS04549		0,63 0,03					ABC TRANSPORTER ATP-BINDING PROTEIN	IV.D
RSc1177	RS04548			-0,51 0,00		-0,44 0,01		TRANSMEMBRANE COG0791, Cell wall-associated hydrolase	III.B
RSc1178	RS04543		0,51 0,02					TRANSMEMBRANE COG1752, Esterase of the alpha-beta hydrolase superfamily	I
RSc1179	RS04541			-0,37 0,04		-0,86 0,02	gltX	GLUTAMATE-tRNA SYNTHETASE (CATALYTIC SUBUNIT)(SYE PROTEIN)	II.D.1
RSc1180	RS05712				-0,56 0,02			TRANSMEMBRANE COG0534, Na+-driven multidrug efflux pump	IV.D
RSc1181	RS05713			-0,38 0,00				TRANSMEMBRANE COG1807, 4-amino-4-deoxy-L-arabinose transferase and related	II.B
RSc1182	RS05714			-0,41 0,05		-0,95 0,00	rpmE	50S RIBOSOMAL SUBUNIT PROTEIN L31	III.C.2
RSc1183	RS05715			-0,44 0,02				SIGNAL PEPTIDE	VI.D
RSc1184	RS05716		0,65 0,03	-0,78 0,00				CONSERVED HYPOTHETICAL PROTEIN	VI.C
RSc1185	RS05717			-0,93 0,00		1,21 0,01		TRANSCRIPTION REGULATOR	VI.A
RSc1186	RS05718			-0,80 0,00				TRANSCRIPTION REGULATOR SIGMA-54 INTERACTING PROTEIN	VI.A
RSc1187	RS05719		0,60 0,00	-0,27 0,02		-0,84 0,00	rho	TRANSCRIPTION TERMINATION FACTOR RHO (HELICASE)	II.B.6
RSc1188	RS05720		0,47 0,05	-0,56 0,00	-0,76 0,00		trxA	THIOREDOXIN 1 (REDOX FACTOR)	I.B.6
RSc1189	RS05721							COG3893, Inactivated superfamily I helicase AND COG2887, RecB family exonuclea	II.B.8
RSc1190	RS05722		1,48 0,03				recB	ATP-dependent exoDNase (exonuclease V) beta subunit (contains helicase and exon	II.B.8
RSc1191	RS05723		0,56 0,01				dnaX	DNA POLYMERASE III (SUBUNITS TAU AND GAMMA)	II.B.8
RSc1192	RS05724		0,76 0,03					CONSERVED HYPOTHETICAL PROTEIN	VI.C
RSc1193	RS05725		0,53 0,01		-0,50 0,05			HYPOTHETICAL PROTEIN	VI.D
RSc1194	RS05726		0,48 0,02	-0,11 0,04	0,42 0,05		recR	DNA REPAIR DNA RECOMBINATION ZINC-FINGER PROTEIN RECR	II.B.8
RSc1195	RS05727							CONSERVED HYPOTHETICAL PROTEIN	VI.C
RSc1196	RS05728				-0,46 0,04			TRANSCRIPTION REGULATOR DNA-BINDING TRANSCRIPTIONAL REGULATORY	VI.A
RSc1197	RS05729		-0,35 0,02			3,06 0,00		OXIDOREDUCTASE COG1804, Acyl-CoA transferases/carnitine dehydratase	I
RSc1198	RS05730		0,64 0,00	-0,44 0,04	0,91 0,01			ABC TRANSPORTER COG0715, ABC-type nitrate/sulfonate/bicarbonate transport sys	IV.D
RSc1199	RS02665		0,65 0,01					ABC TRANSPORTER COG1116, ABC-type nitrate/sulfonate/bicarbonate transport sys	IV.D
RSc1200	RS02669			-0,20 0,05				ABC TRANSPORTER COG0600, ABC-type nitrate/sulfonate/bicarbonate transport sys	IV.D
RSc1201	RS02672	-1,58 0,04		-1,55 0,00	-1,85 0,00			TRANSCRIPTION REGULATOR	VI.A
RSc1202	RS02674	-1,11 0,04		-1,11 0,00				OXIDOREDUCTASE FLAVOPROTEIN NADH-DEPENDENT	I.F
RSc1203	RS02677			-1,14 0,00				OXIDOREDUCTASE	I
RSc1204	RS02681		0,37 0,05	-0,53 0,00	0,42 0,04		surE	STATIONARY-PHASE SURVIVAL PROTEIN	IV.G
RSc1205	RS02685					-0,85 0,00		COG2518, Protein-L-isoaspartate carboxylmethyltransferase	II.B.7
RSc1206	RS02686			-0,62 0,00	-0,92 0,03			LIPOPROTEIN	VI.C
RSc1207	RS02688			-0,31 0,04			rpoS	TRANSCRIPTION REGULATOR RNA POLYMERASE SIGMA S (SIGMA-38) FACTO	I.E.1
RSc1208	RS02691			-0,57 0,01				COG3568, Metal-dependent hydrolase	I.I
RSc1209	RS02696		0,91 0,01					RNA METHYLTRANSFERASE	II.B.6
RSc1210	RS02699							TRANSMEMBRANE	VI.C
RSc1211	RS02703		0,61 0,01		0,51 0,03	-0,45 0,02	ndk	NUCLEOSIDE-DIPHOSPHATE KINASE PROTEIN	I.D.2
RSc1212	RS02704		0,42 0,03			-0,93 0,02		COG0820, Fe-S-cluster redox enzyme	I
RSc1213	RS02705		0,34 0,04	-0,41 0,00		-0,32 0,02	pilF	TYPE 4 FIMBRIAL BIOGENESIS PROTEIN	III.A.2
RSc1214	RS02709			-0,34 0,00				TRANSMEMBRANE	VI.C
RSc1215	RS02710			-0,47 0,00			gcpE	COG0821, GcpE enzyme involved in the deoxyxylulose pathway of isoprenoid biosyn	I
RSc1216	RS02790		0,51 0,01			-0,63 0,00	hisS	HISTIDINE-TRNA SYNTHETASE	II.D.1
RSc1217	RS02712							TRANSMEMBRANE	VI.C
RSc1218	RS02713		0,48 0,02	-0,28 0,01		-0,52 0,00		TRANSMEMBRANE COG1520, FOG: WD40-like repeat	VI.C
RSc1219	RS02721							COG1160, GTPase	VI.C
RSc1220	RS02722			-0,23 0,04	-0,75 0,02		hfq	HOST FACTOR-I PROTEIN	V.D
RSc1221	RS02723		0,51 0,03	-0,56 0,00		-0,96 0,00	hflX	COG2262, GTPase	VI.C
RSc1222	RS02724			-0,68 0,00			hflK	TRANSMEMBRANE COG0330, Membrane protease subunits, stomatin/prohibitin hom	II.B.7
RSc1223	RS02730		0,51 0,01	-0,48 0,00		-0,41 0,00		TRANSMEMBRANE SERINE PROTEASE	II.A.4
RSc1224	RS02731							TRANSMEMBRANE	VI.C
RSc1225	RS02732		0,63 0,02				hisZ	COG3705, ATP phosphoribosyltransferase involved in histidine biosynthesis	I.A.4
RSc1226	RS02733				-0,41 0,01		purA	ADENYLOSUCCINATE SYNTHETASE	I.D.2
RSc1227	RS02734							NUCLEOTIDE PHOSPHORIBOSYLTRANSFERASE	I.F.9
RSc1228	RS02735		0,66 0,02			-0,66 0,05	rnr	EXORIBONUCLEASE RNase R (VACB PROTEIN)	II.A.2
RSc1229	RS02736		0,51 0,02					TRNA/RRNA METHYLTRANSFERASE PROTEIN	II.B.6
RSc1230	RS02737							COG0846, NAD-dependent protein deacetylase	II.B.6
RSc1231	RS02738				1,48 0,00		tal	TRANSALDOLASE	I.F.19
RSc1232	RS02791		0,91 0,01	-0,34 0,04		-0,95 0,02	rpiA	RIBOSE 5-PHOSPHATE ISOMERASE	I.F.19
RSc1233	RS02740							TRANSMEMBRANE COG1953, Cytosine/uracil/thiamine/allantoin permease	IV.D
RSc1234	RS02741			0,35 0,00				TRANSMEMBRANE METHYL-ACCEPTING CHEMOTAXIS PROTEIN	IV.C
RSc1235	RS02742				-1,34 0,01	-1,13 0,03		CONSERVED HYPOTHETICAL PROTEIN	VI.C
RSc1236	RS02744							OUTER MEMBRANE PROTEIN (PORIN)	III.B.2
RSc1237	RS02745			-0,99 0,02			iolB	MYO-INOSITOL CATABOLISM PROTEIN	I.I.4
RSc1238	RS02746			-0,43 0,04			iolE	MYO-INOSITOL CATABOLISM PROTEIN	I.I.4
RSc1239	RS02747						iolD	ACETOLACTATE SYNTHASE	I.I.4
RSc1240	RS02748						iolC	TRANSFERASE KINASE	I.I.4
RSc1241	RS02749						mocB	SIGNAL PEPTIDE RHIZOPINE-BINDING PROTEIN PRECURSOR	I.I.4
RSc1242	RS02750							ABC TRANSPORTER SUGAR ATP-BINDING PROTEIN	IV.D.3

RSc1243	RS02751							ABC TRANSPORTER SUGAR TRANSMEMBRANE PROTEIN	IV.D.3
RSc1244	RS02752			-0,26 0,05			iolH	PROTEIN IMPLICATED IN MYO-INOSITOL CATABOLIQUE PATHWAY	I.I.4
RSc1245	RS02753						iolI	PROTEIN IMPLICATED IN MYO-INOSITOL CATABOLIQUE PATHWAY	I.I.4
RSc1246	RS02754						iolG	SIGNAL PEPTIDE OXIDOREDUCTASE MYO-INOSITOL 2-DEHYDROGENASE	I.I.4
RSc1247	RS02755			-1,04 0,03			iolE	COG1082, Sugar phosphate isomerases/epimerases	I
RSc1248	RS02756			-0,62 0,03				OXIDOREDUCTASE COG0673, Dehydrogenases and related proteins	I
RSc1249	RS02757			-0,93 0,02	-0,88 0,01			TRANSCRIPTION REGULATOR	VI.A
RSc1250	RS02762	1,56 0,04					argA	AMINO-ACID ACETYLTRANSFERASE	I.A.17
RSc1251	RS02767			-0,34 0,00			hrpA	ATP-DEPENDENT RNA HELICASE	II.B.8
RSc1252	RS02768							SIGNAL PEPTIDE	VI.C
RSc1253	RS02769			-0,46 0,02				SIGNAL PEPTIDE COG0726, Xylanase/chitin deacetylase	II.A.3
RSc1254	RS02770			-0,40 0,03				TRANSMEMBRANE COG3000, Sterol desaturase	I
RSc1255	RS02771		0,43 0,02	-0,38 0,01	1,01 0,00	1,55 0,00		TRANSMEMBRANE	VI.C
RSc1256	RS02772		0,65 0,01		-0,58 0,02			COG1058, Nucleotide-utilizing enzyme related to molybdopterin-biosynthesis enzyme	VI.C
RSc1257	RS02773							COG0607, Rhodanese-related sulfurtransferase	I
RSc1258	RS02774		0,44 0,02	-0,52 0,03	0,92 0,01	-0,75 0,00	glnA1	GLUTAMINE SYNTHETASE	I.A.18
RSc1259	RS02776			-0,45 0,01				SIGNAL PEPTIDE	VI.C
RSc1260	RS02784		0,38 0,02	-0,47 0,00			ntrB	TRANSCRIPTION REGULATOR NITROGEN REGULATION PROTEIN (SENSOR PR	I.F.5
RSc1261	RS02778		0,45 0,04	-0,43 0,01			ntrC	TRANSCRIPTION REGULATOR NITROGEN ASSIMILATION REGULATORY PROTE	I.F.5
RSc1262	RS02779							CONSERVED HYPOTHETICAL PROTEIN	VI.C
RSc1263	RS02780		0,57 0,01					COG0561,Hydrolase of the HAD superfamily	I.I
RSc1264	RS02781						ugpB	SIGNAL PEPTIDE GLYCEROL-3-PHOSPHATE-BINDING PERIPLASMIC LIPOPROT	IV.D.3
RSc1265	RS02782						ugpA	ABC TRANSPORTER SN-GLYCEROL-3-PHOSPHATE TRANSMEMBRANE PROTEI	I.F.11
RSc1267	RS05781						upgC	ABC TRANSPORTER SN-GLYCEROL-3-PHOSPHATE TRANSPORT ATP-BINDING	I.F.11
RSc1268	RS05782		2,20 0,01				ugpQ	GLYCEROPHOSPHORYL DIESTER PHOSPHODIESTERASE	I.F.4
RSc1269	RS05783		1,12 0,01				odhA	OXIDOREDUCTASE OXOGLUTARATE DEHYDROGENASE	I.H.8
RSc1270	RS02808		1,11 0,01		0,51 0,02		sucB	DIHYDROLIPOAMIDE SUCCINYLTRANSFERASE (COMPONENT OF 2-OXOGLUT/	I.H.6
RSc1271	RS02809		1,22 0,00			-0,76 0,00		OXIDOREDUCTASE DIHYDROLIPOAMIDE DEHYDROGENASE (COMPONENT OF	I.H.7
RSc1272	RS02810							COG1485, ATPase	VI.C
RSc1273	RS02811			-1,22 0,04				COG2045, Phosphosulfolactate phosphohydrolase and related enzymes	I
RSc1274	RS02812							TRANSMEMBRANE CATION-TRANSPORTING ATPASE LIPOPROTEIN	IV.D.4
RSc1275	RS02813							SIGNAL PEPTIDE	VI.C
RSc1276	RS02814							TRANSMEMBRANE CYTOCHROME C OXIDASE (SUBUNIT I)	I.G.1
RSc1277	RS02815							TRANSMEMBRANE CYTOCHROME C OXIDASE (SUBUNIT II)	I.G.1
RSc1278	RS02816							OXIDOREDUCTASE COG4736, Cbb3-type cytochrome oxidase, subunit 3	I
RSc1279	RS02792							TRANSMEMBRANE CYTOCHROME C OXIDASE (SUBUNIT III)	I.G.1
RSc1280	RS02793			-0,87 0,02				TRANSMEMBRANE IRON-SULFUR 4Fe-4S FERREDOXIN	I.G.1
RSc1281	RS02794							TRANSMEMBRANE LIPOPROTEIN	VI.D
RSc1282	RS02795							TRANSMEMBRANE	VI.C
RSc1283	RS02796	-1,22 0,03		-0,33 0,03			fnr	TRANSCRIPTION REGULATOR FUMARATE AND NITRATE REDUCTION REGULA	I.E.1
RSc1284	RS02797			-0,55 0,02				TRANSMEMBRANE	VI.C
RSc1285	RS02798		0,63 0,01					TRANSCRIPTION REGULATOR COG1386, Transcriptional regulator containing the H	VI.A
RSc1286	RS02799		0,85 0,00	-0,53 0,05		-0,28 0,05		COG1187, 16S rRNA uridine-516 pseudouridylate synthase and related pseudouridyla	III.C.1
RSc1287	RS02964					-2,00 0,05		CONSERVED HYPOTHETICAL PROTEIN	VI.C
RSc1288	RS02801		0,59 0,01				nusA	TRANSCRIPTION REGULATOR N UTILIZATION SUBSTANCE PROTEIN	II.B.6
RSc1289	RS02965		0,98 0,00		0,58 0,03		infB	COG0532, Translation initiation factor 2 (IF-2 GTPase)	II.B.7
RSc1290	RS02803					-1,09 0,00	rbfA	RIBOSOME-BINDING FACTOR A	II.B.7
RSc1291	RS02804		0,62 0,01	-0,38 0,02		-0,45 0,02	truB	TRNA PSEUDOURIDINE SYNTHASE B	II.D.1
RSc1292	RS02805		0,86 0,00				emrB	TRANSMEMBRANE MULTIDRUG RESISTANCE PROTEIN B (TRANSLOCASE)	IV.G.2
RSc1293	RS02806						emrA	TRANSMEMBRANE MULTIDRUG RESISTANCE PROTEIN A (TRANSLOCASE)	IV.D
RSc1294	RS02807			-1,06 0,00				OUTER MEMBRANE CHANEL LIPOPROTEIN	IV.D
RSc1295	RS02817							TRANSCRIPTION REGULATOR	VI.A
RSc1296	RS02818		0,53 0,04				typA	COG1217, Membrane GTPase TypA involved in stress response	IV.F
RSc1297	RS02819							TRANSMEMBRANE PSEUDOGENE (Sterol desaturase FRAGMENT)	VI.B
RSc1298	RS02820		0,65 0,00	-0,48 0,00				ENOYL(3-HYDROXYISOBUTYRYL)-COENZYME A HYDRATASE	I.I.4
RSc1299	RS02821		0,68 0,02		0,50 0,00	-0,88 0,02		COG0042, tRNA-dihydrouridine synthase	III.C.1
RSc1300	RS02822		0,53 0,00	-0,35 0,03				TRANSMEMBRANE	VI.B
RSc1301	RS02823			-0,34 0,01				CONSERVED HYPOTHETICAL PROTEIN	VI.C
RSc1302	RS02824						pepV	AMINOACYL-HISTIDINE DIPEPTIDASE (CARNOSINASE) (XAA-HIS DIPEPTIDASE	I.F.14
RSc1303	RS02825			-0,34 0,01				HYPOTHETICAL PROTEIN	VI.D
RSc1304	RS02826			-0,41 0,02			lexA	TRANSCRIPTION REGULATOR TRANSCRIPTION REGULATION REPRESSOR	I.E.1
RSc1305	RS02827				-1,13 0,02		fpr	OXIDOREDUCTASE FERREDOXIN--NADP REDUCTASE	I.F.18
RSc1306	RS02828		0,52 0,01	-0,29 0,03			ansB	SIGNAL PEPTIDE L-ASPARAGINASE II PRECURSOR	I.I.3
RSc1307	RS02829			-0,44 0,00		-0,79 0,00	rpsF	30S RIBOSOMAL SUBUNIT PROTEIN S6	III.C.2
RSc1308	RS02830			-0,29 0,02	1,03 0,05		priB	Primosomal replication protein N	II.B.8
RSc1309	RS02831		0,55 0,03	-0,40 0,00	0,67 0,00	-0,80 0,00	rpsR	PRIMOSOMAL REPLICATION PROTEIN	III.C.2
RSc1310	RS02832			-0,38 0,00	0,68 0,03	-0,74 0,00	rplI	50S RIBOSOMAL SUBUNIT PROTEIN L9	III.C.2
RSc1311	RS02833		0,75 0,01	-0,34 0,01			dnaB	REPLICATIVE DNA HELICASE	II.B.8
RSc1312	RS02834		0,86 0,01	-0,31 0,03	0,50 0,05	0,56 0,00		COG1392, Phosphate transport regulator (distant homolog of PhoU)	IV.D
RSc1313	RS02835						pit	TRANSMEMBRANE LOW-AFFINITY PHOSPHATE TRANSPORTER LIPOPROTEIN	IV.D.1
RSc1314	RS02836			-0,85 0,00	-0,53 0,04	0,58 0,01		TRANSMEMBRANE COG0791, Cell wall-associated hydrolase	III.B
RSc1315	RS02837			-0,49 0,02				SIGNAL PEPTIDE	VI.C
RSc1316	RS02966			-0,68 0,00				COG1875, ATPase related to phosphate starvation-inducible protein PhoH	VI.C
RSc1317	RS02839		0,55 0,01	-0,41 0,00		-0,76 0,00	bcp	OXIDOREDUCTASE BACTERIOFERRITIN COMIGRATORY PROTEIN	II.B.7
RSc1318	RS02840			-0,37 0,05				COG0726, Xylanase/chitin deacetylase	II.A.3
RSc1319	RS02842		0,53 0,00	-0,40 0,05				OXIDOREDUCTASE COG0451, Nucleoside-diphosphate-sugar epimerases	I
RSc1320	RS02841		0,70 0,01					COG0223, Methionyl-tRNA formyltransferase	II.B.7
RSc1321	RS02843		0,66 0,00				pmrF	TRANSMEMBRANE POLYMIXIN RESISTANCE GLYCOSYLTRANSFERASE PROTE	IV.G.2
RSc1322	RS02844		0,24 0,05	-0,36 0,00				COG0399, Pyridoxal phosphate-dependent enzyme apparently involved in regulation	VI.C
RSc1323	RS02845		0,45 0,01	-0,37 0,02		0,48 0,05		TRANSMEMBRANE Small multidrug resistance protein	IV.G.2
RSc1324	RS02846	0,53 0,04	0,71 0,01	-0,35 0,01				TRANSMEMBRANE COG1807, 4-amino-4-deoxy-L-arabinose transferase and related	I
RSc1325	RS02847	0,50 0,01		-0,36 0,00				CONSERVED HYPOTHETICAL PROTEIN	VI.C
RSc1326	RS02848		0,68 0,01	-0,36 0,01				ASPARTATE AMINOTRANSFERASE	I.A
RSc1327	RS02849							OXIDOREDUCTASE HOMOSERINE DEHYDROGENASE	I.A.12
RSc1328	RS02850		0,67 0,01	-0,38 0,00		-1,66 0,00	thrC	THREONINE SYNTHASE	I.A.12
RSc1329	RS02851		0,27 0,04	-0,43 0,00				COG1573, Uracil-DNA glycosylase	II.B.8
RSc1330	RS02852			-0,30 0,05			moeA2	MOLYBDOPTERIN BIOSYNTHESIS MOEA PROTEIN	I.B.12
RSc1331	RS02853		0,63 0,02	-0,22 0,03			moaD	MOLYBDOPTERIN MPT CONVERTING FACTOR (SUBUNIT 1)	I.B.12
RSc1332	RS02854						moaE	MOLYBDOPTERIN MPT CONVERTING FACTOR (SUBUNIT 2)	I.B.12
RSc1333	RS02855		0,40 0,05				crcB	TRANSMEMBRANE	IV.A
RSc1334	RS02856					-0,84 0,00		TRANSMEMBRANE D--3-HYDROXYBUTYRATE OLIGOMER HYDROLASE LIPOPR	I.I.4
RSc1335	RS02857		0,59 0,02	-0,18 0,03		-0,36 0,04	clpB	ATP-DEPENDENT PROTEASE (HEAT SHOCK PROTEIN)	II.A.4
RSc1336	RS02858		0,89 0,00	0,39 0,02			sbp	SIGNAL PEPTIDE SULFATE-BINDING PROTEIN PRECURSOR	IV.E.2
RSc1337	RS02859		1,19 0,03		-2,02 0,01			OXIDOREDUCTASE NADPH-DEPENDENT FMN REDUCTASE	I.G.1
RSc1338	RS02860		0,75 0,01		-1,88 0,00	-1,83 0,00	ssuA1	SIGNAL PEPTIDE ALKANESULFONATES BINDING PROTEIN PRECURSOR	I.F.20
RSc1339	RS02861						ssuD	OXIDOREDUCTASE ALKANESULFONATE MONOOXYGENASE	I.F.3
RSc1340	RS02862	-0,67 0,05	1,02 0,00		-0,92 0,05		ssuC	ABC TRANSPORTER ALIPHATIC SULFONATES TRANSMEMBRANE PROTEIN	IV.D.3
RSc1341	RS02863		1,20 0,00		-0,69 0,04	-2,21 0,01	ssuB	ABC TRANSPORTER ALIPHATIC SULFONATES ATP-BINDING PROTEIN	IV.D.3
RSc1342	RS02864		1,04 0,00	-0,26 0,01	-1,31 0,00	-0,57 0,01	ssuF	MOLYBDOPTERIN-BINDING PROTEIN	I.F.3
RSc1343	RS02865		0,72 0,00					SIGNAL TRANSDUCTION PROTEIN EAL DOMAIN	VI.A
RSc1344	RS02866		-1,18 0,00	2,23 0,00				COG3545, Predicted esterase of the alpha/beta hydrolase fold	I
RSc1345	RS02868		1,01 0,01				cysU	ABC TRANSPORTER SULFATE TRANSPORT PROTEIN	I.F.3
RSc1346	RS02869		0,97 0,01	0,93 0,00			cysW	ABC TRANSPORTER SULFATE TRANSPORT PROTEIN	I.F.3
RSc1347	RS02870		0,78 0,00	0,64 0,00	-1,54 0,01		cysA	ABC TRANSPORTER SULFATE TRANSPORT ATP-BINDING PROTEIN	I.F.3
RSc1348	RS02871		0,67 0,01			-1,30 0,00	cysB1	TRANSCRIPTION REGULATOR CYS REGULON TRANSCRIPTIONAL ACTIVATOR	I.A.9
RSc1349	RS02872		-2,61 0,00	3,08 0,00	1,47 0,01			HYPOTHETICAL PROTEIN	VI.B
RSc1350	RS02873		-0,82 0,03					TRANSMEMBRANE	VI.B
RSc1351	RS02874		1,25 0,02	-0,49 0,02	-1,08 0,00			TRANSCRIPTION REGULATOR TRANSMEMBRANE SENSOR HISTIDINE KINASE	VI.A
RSc1352	RS02875				-1,52 0,01			TRANSCRIPTION REGULATOR TWO-COMPONENT RESPONSE REGULATOR	VI.A
RSc1353	RS05753			-0,19 0,03			ilvG	ACETOLACTATE SYNTHASE ISOZYME II (LARGE SUBUNIT)	I.A.2
RSc1354	RS04636						menG	S-ADENOSYLMETHIONINE:2-DEMETHYLMENAQUINONE METHYLTRANSFERAS	I.B.5
RSc1355	RS04637							CONSERVED HYPOTHETICAL PROTEIN	VI.C

RSc1356	RS04638			-4,22	0,00	4,62	0,00	4,71	0,00			ripG	TYPE III EFFECTOR PROTEIN GALA6	VI.E
RSc1357	RS04639	-3,02	0,00	-6,06	0,00	6,84	0,00	4,26	0,00				LRR-GALA FAMILY TYPE III EFFECTOR PROTEIN (GALA 7)	VI.C
RSc1358	RS04640	0,64	0,05	-0,33	0,05	0,49	0,00	0,78	0,04	0,76	0,00	aceA	ISOCITRATE LYASE	I.F.17
RSc1359	RS04641												COG0589, Universal stress protein UspA and related nucleotide-binding proteins	IV.G
RSc1360	RS04642					-0,57	0,03						TRANSCRIPTION REGULATOR	VI.A
RSc1361	RS04643			-0,57	0,03	0,54	0,02						TRANSMEMBRANE	VI.D
RSc1362	RS04644					-0,51	0,01	-1,11	0,00				COG1011, Predicted hydrolase	I.I
RSc1363	RS04645							0,96	0,05			aceB	MALATE SYNTHASE A (MSA OR MASY PROTEIN)	I.F.17
RSc1364	RS04646					-0,36	0,00						CONSERVED HYPOTHETICAL PROTEIN	VI.C
RSc1365	RS04647			0,94	0,03								TRANSMEMBRANE MULTIDRUG RESISTANCE-LIKE EFFLUX PROTEIN	IV.D.7
RSc1366	RS04648					-0,72	0,00						ACYL-COA-BINDING PROTEIN	IV.D.7
RSc1367	RS04650												TRANSMEMBRANE COG1214, Inactive homolog of metal-dependent proteases, puta	VI.C
RSc1368	RS04651					-0,14	0,01						ACETYLTRANSFERASE	I
RSc1369	RS04652					-0,36	0,00			-1,24	0,01		COG1573, Uracil-DNA glycosylase	II.B.8
RSc1370	RS04653					-0,62	0,01			-0,57	0,01		TRANSMEMBRANE	VI.C
RSc1371	RS04748											dadX	ALANINE RACEMASE	I.A.3
RSc1372	RS04749					-0,36	0,02			-0,58	0,01	radA	ATP-DEPENDENT PROTEASE, DNA REPAIR (ATP-BINDING DNA-BINDING) PROT	II.A.4
RSc1373	RS04656											dsbB	OXIDOREDUCTASE TRANSMEMBRANE DISULFIDE BOND FORMATION PROTEIN	II.B.7
RSc1374	RS04657			0,31	0,04	-0,44	0,02			-1,03	0,05		CONSERVED HYPOTHETICAL PROTEIN	VI.C
RSc1375	RS04658					-0,61	0,00						TRANSMEMBRANE COG1804, Predicted acyl-CoA transferases/carnitine dehydratas	I
RSc1376	RS04659							0,54	0,05				ABC TRANSPORTER ATP-BINDING PROTEIN	IV.D
RSc1377	RS04660					-1,53	0,00	-2,74	0,00				TRANSCRIPTION REGULATOR	VI.A
RSc1378	RS04661					-0,97	0,00						L-ASPARAGINASE PRECURSOR	I
RSc1379	RS04662					-0,73	0,00						ABC TRANSPORTER ATP-BINDING PROTEIN	IV.D
RSc1380	RS04663					-0,74	0,01						ABC TRANSPORTER SUBSTATE-BINDING PERIPLASMIC PROTEIN (PBP)	IV.D
RSc1381	RS04750			0,34	0,03	-0,55	0,02						ABC TRANSPORTER TRANSMEMBRANE PROTEIN	IV.D
RSc1382	RS04665			0,33	0,03								ABC TRANSPORTER TRANSMEMBRANE PROTEIN	IV.D
RSc1383	RS04666					-0,69	0,05						COG3191, L-aminopeptidase/D-esterase	II.A.4
RSc1384	RS04667	-0,56	0,05			-0,88	0,04			-1,29	0,01		COG2362, D-aminopeptidase	II.A.4
RSc1385	RS04668					-0,20	0,03						TRANSMEMBRANE LIPOPROTEIN	VI.D
RSc1386	RS04669			-3,07	0,00	3,72	0,00	3,95	0,00				CONSERVED HYPOTHETICAL PROTEIN	VI.C
RSc1387	RS04670									-0,71	0,00	folE	GTP CYCLOHYDROLASE I	I.B.14
RSc1388	RS04671	-0,52	0,04	0,47	0,02					-0,86	0,00		METHYLASE/METHYLTRANSFERASE	II.B.8
RSc1389	RS04672			0,68	0,02								COG1437, Adenylate cyclase, class 2 (thermophilic)	I
RSc1390	RS04673			0,69	0,04					-0,72	0,04	dapE	SUCCINYL-DIAMINOPIMELATE DESUCCINYLASE	I.A.13
RSc1391	RS04674	-1,05	0,01	0,82	0,00								COG1393, Arsenate reductase and related proteins	I
RSc1392	RS04675												TRANSMEMBRANE	VI.C
RSc1393	RS04676			1,02	0,00	-0,15	0,03	-0,59	0,04	-0,52	0,00	dapD	2,3,4,5-TETRAHYDROPYRIDINE-2-CARBOXYLATE N-SUCCINYLTRANSFERASE	I.A.13
RSc1394	RS04677					-0,43	0,01	0,85	0,00			dapC	SUCCINYLDIAMINOPIMELATE AMINOTRANSFERASE	I.A.13
RSc1395	RS05759			0,49	0,02								TRANSMEMBRANE	VI.C
RSc1396	RS05760					-0,73	0,01						TRANSMEMBRANE COG0596, Predicted hydrolases or acyltransferases (alpha/beta	I
RSc1397	RS05762			0,54	0,00			1,03	0,00				TRANSMEMBRANE	VI.C
RSc1398	RS05294											ligA	DNA LIGASE (POLYDEOXYRIBONUCLEOTIDE SYNTHASE [NAD+])	II.B.8
RSc1399	RS05293			0,39	0,01	-0,66	0,00	-0,88	0,05				POLYPEPTIDE DEFORMYLASE	II.B.7
RSc1400	RS05292												TRANSMEMBRANE	VI.C
RSc1401	RS05291												HYPOTHETICAL PROTEIN	VI.B
RSc1402	RS05290			0,57	0,01	-0,43	0,02					glnD	PROTEIN-PII URIDYLYLTRANSFERASE	I.A.18
RSc1403	RS05289											map	METHIONINE AMINOPEPTIDASE	II.B.7
RSc1404	RS05288			0,80	0,05					-1,11	0,02	rpsB	30S RIBOSOMAL PROTEIN S2	III.C.2
RSc1405	RS05287			0,73	0,01					-0,94	0,00	tsf	ELONGATION FACTOR TS (EF-TS)	II.B.7
RSc1406	RS05286			0,71	0,02					-0,64	0,05	pyrH	URIDYLATE KINASE	I.F.9
RSc1407	RS05285					-0,50	0,02			0,99	0,00	rrf	RIBOSOME RECYCLING FACTOR (RIBOSOME RELEASING FACTOR)	II.B.7
RSc1408	RS05284							0,63	0,01	0,64	0,04	uppS	UNDECAPRENYL PYROPHOSPHATE SYNTHETASE	III.B.3
RSc1409	RS05283					0,37	0,04					cdsA	TRANSMEMBRANE PHOSPHATIDATE CYTIDYLYLTRANSFERASE	I.B.1
RSc1410	RS05282											dxr	OXIDOREDUCTASE 1-DEOXY-D-XYLULOSE 5-PHOSPHATE REDUCTOISOMERA	I.B.5
RSc1411	RS05281					-0,65	0,00						TRANSMEMBRANE COG0750, Predicted membrane-associated Zn-dependent prote	II.A.4
RSc1412	RS05280			0,54	0,00								SIGNAL PEPTIDE COG4775, Outer membrane protein	III.B.2
RSc1413	RS05279			0,50	0,00			0,42	0,00				TRANSMEMBRANE COG2825, OUTER MEMBRANE PROTEIN	III.B.2
RSc1414	RS05278									-0,56	0,00	lpxD	UDP-3-O-3-HYDROXYMYRISTOYL GLUCOSAMINE N-ACYLTRANSFERASE	III.A.1
RSc1415	RS05277									-0,50	0,03	fabZ	3R-HYDROXYMYRISTOYL-ACYL CARRIER PROTEIN DEHYDRATASE	III.A.1
RSc1416	RS05276									-0,68	0,01	lpxA	ACYL-ACYL-CARRIER-PROTEIN--UDP-N-ACETYLGLUCOSAMINE O-ACYLTRANS	III.A.1
RSc1417	RS05275	-0,29	0,04	0,62	0,01	-0,23	0,00	-0,69	0,04	-1,51	0,00	lpxB	LIPID-A-DISACCHARIDE SYNTHASE	III.A.1
RSc1418	RS05274							-0,84	0,03			rnhB	RIBONUCLEASE HII	II.A.2
RSc1419	RS05273							-1,40	0,02				COG0566, rRNA methylases	III.C.1
RSc1420	RS05272									-0,74	0,03		ATP-BINDING PROTEIN	VI.C
RSc1421	RS05271											ppsA	PHOSPHOENOLPYRUVATE SYNTHASE	I.F.13
RSc1422	RS05270					-0,38	0,00						TRANSMEMBRANE COG1585, Membrane protein implicated in regulation of membra	III.B.1
RSc1423	RS05269					-0,57	0,02						TRANSMEMBRANE COG0330, Membrane protease subunits, stomatin/prohibitin hom	III.B.1
RSc1424	RS05268					-0,23	0,01			-0,74	0,00	smpB	tmRNA-BINDING PROTEIN (SMALL PROTEIN B)	IV.B
RSc1425	RS05267									-0,91	0,05		COG2867, Oligoketide cyclase/lipid transport protein	I
RSc1426	RS05266												CONSERVED HYPOTHETICAL PROTEIN	VI.C
RSc1427	RS05265					-0,50	0,03						TRANSMEMBRANE	VI.C
RSc1428	RS05264					-0,33	0,00						TRANSMEMBRANE	VI.C
RSc1429	RS05263			0,35	0,05					-0,82	0,00	guaB	OXIDOREDUCTASE INOSINE-5'-MONOPHOSPHATE DEHYDROGENASE	I.D.2
RSc1430	RS05261			0,70	0,02			0,85	0,01				TRANSMEMBRANE	VI.C
RSc1431	RS05262			0,65	0,01	-0,14	0,03	0,89	0,00			guaA	GMP SYNTHASE GLUTAMINE-HYDROLYZING (GLUTAMINE AMIDOTRANSFERA	I.D.2
RSc1432	RS05734					-0,49	0,00						TRANSCRIPTION REGULATOR	VI.A
RSc1433	RS05735					-0,40	0,04						TRANSMEMBRANE	VI.D
RSc1440	RS03867											pcaQ	TRANSCRIPTION REGULATOR TRANSCRIPTIONAL REGULATORY DNA-BINDING	I.I.4
RSc1441	RS03866					-0,42	0,00					pcaH	OXIDOREDUCTASE PROTOCATECHUATE 3,4-DIOXYGENASE (BETA CHAIN)	I.I.4
RSc1442	RS03865											pcaG	OXIDOREDUCTASE PROTOCATECHUATE 3,4-DIOXYGENASE (ALPHA CHAIN)	I.I.4
RSc1443	RS03864												2H PHOSPHOESTERASE (2'-5' RNA LIGASE)	II.B.6
RSc1444	RS03863							0,83	0,04				COG3531, Predicted protein-disulfide isomerase	II.B.7
RSc1445	RS03862												TRANSMEMBRANE COG2814, Arabinose efflux permease	IV.D.3
RSc1446	RS03861	1,29	0,05	0,35	0,01	-0,87	0,00	-1,44	0,02			hipA	TRANSCRIPTION REGULATOR HIPA PROTEIN	III.B.3
RSc1447	RS03860							-0,82	0,05				TRANSCRIPTION REGULATOR	VI.A
RSc1448	RS03859			0,30	0,01								COG2513, PEP phosphonomutase and related enzymes	I
RSc1449	RS03858					-0,59	0,00						COG0602, Organic radical activating enzymes	II.B.7
RSc1450	RS03857			0,70	0,01					-0,96	0,01		6-PYRUVOYL TETRAHYDROBIOPTERIN SYNTHASE	I
RSc1451	RS03856					-0,34	0,01			-0,51	0,00		COG0590, Cytosine/adenosine deaminase	I
RSc1452	RS03855			0,79	0,00			-1,84	0,01				TRANSMEMBRANE	VI.C
RSc1453	RS03854			0,66	0,00	-0,30	0,04						ABC TRANSPORTER ATP-BINDING PROTEIN	IV.D
RSc1454	RS03853					-0,63	0,02						SIGNAL PEPTIDE	VI.D
RSc1455	RS03852												PROLINE-RICH PROTEIN	VI.B
RSc1456	RS03851											betB	OXIDOREDUCTASE BETAINE ALDEHYDE DEHYDROGENASE (BADH)	I.F
RSc1457	RS03850												COG3313, Predicted Fe-S protein	VI.C
RSc1458	RS03849					-0,35	0,03						COG3387, Glucoamylase and related glycosyl hydrolases	II.A.3
RSc1459	RS03848					-0,38	0,01						CONSERVED HYPOTHETICAL PROTEIN	VI.C
RSc1460	RS03847							-0,65	0,03				TRANSMEMBRANE METHYL-ACCEPTING CHEMOTAXIS TRANSDUCER PROTEIN	IV.C
RSc1461	RS03846												TRANSMEMBRANE TRANSPORTER PROTEIN	IV.D
RSc1462	RS03845												SIGNAL PEPTIDE COG0451, Nucleoside-diphosphate-sugar epimerase	I
RSc1463	RS03844												COG1975: Xanthine and CO dehydrogenases maturation factor	II.B.7
RSc1464	RS03843												TRANSMEMBRANE	VI.B
RSc1465	RS03842												COG3552, Protein containing von Willebrand factor type A (vWA) domain	VI.C
RSc1466	RS03841					0,41	0,02						COG0714 : MoxR- like ATPase	VI.C
RSc1467	RS03840												OXIDOREDUCTASE COG1319, Aerobic-type carbon monoxide dehydrogenase, midd	I
RSc1468	RS03839												OXIDOREDUCTASE COG1529, Aerobic-type carbon monoxide dehydrogenase, large	I
RSc1469	RS03838												OXIDOREDUCTASE COG2080, Aerobic-type carbon monoxide dehydrogenase, smal	I
RSc1470	RS03837					-0,92	0,00	-0,88	0,03				HYPOTHETICAL PROTEIN	VI.D
RSc1471	RS03836											creA	SIGNAL PEPTIDE CREA PROTEIN	VI.C
RSc1472	RS03835												TRANSCRIPTION REGULATOR	VI.A
RSc1473	RS03834					-0,53	0,01						TRANSMEMBRANE MULTIDRUG RESISTANCE-LIKE PROTEIN	IV.D.7

RSc1474	RS03833			-5,74	0,00	6,60	0,00	4,12	0,00				HYPOTHETICAL PROTEIN	VI.D
RSc1475	RS03832	-1,11	0,00	-3,21	0,00	4,06	0,00	4,07	0,00				TRANSMEMBRANE	VI.D
RSc1476	RS03830					-0,28	0,04	1,00	0,05				TRANSMEMBRANE MEMBRANE CARBOXYPEPTIDASE (penicillin-binding protein)	III.B.2
RSc1477	RS03829					-0,21	0,05					bioA	ADENOSYLMETHIONINE-8-AMINO-7-OXONONANOATE AMINOTRANSFERASE	I.B.15
RSc1478	RS03827			0,58	0,00							bioF	8-AMINO-7-OXONONANOATE SYNTHASE	I.B.15
RSc1479	RS03828											bioD	DETHIOBIOTIN SYNTHETASE	I.B.15
RSc1480	RS03826							-0,99	0,05	-0,39	0,01		TRANSMEMBRANE LIPOPROTEIN	VI.C
RSc1481	RS03825			-0,34	0,03	-0,89	0,00						TRANSMEMBRANE	VI.D
RSc1482	RS03824			0,81	0,00								CONSERVED HYPOTHETICAL PROTEIN	VI.C
RSc1483	RS03823			0,34	0,04			-0,60	0,00				CONSERVED HYPOTHETICAL PROTEIN	VI.C
RSc1485	RS03821												HYPOTHETICAL PROTEIN	VI.D
RSc1486	RS03820												CONSERVED HYPOTHETICAL PROTEIN	VI.C
RSc1487	RS03819												CONSERVED HYPOTHETICAL PROTEIN	VI.C
RSc1488	RS03818									-0,68	0,01		CONSERVED HYPOTHETICAL PROTEIN	VI.C
RSc1489	RS03817					-0,30	0,02						PROPHAGE PROTEIN	V.D
RSc1490	RS03816					-1,08	0,00	-1,53	0,00				TRANSMEMBRANE	VI.D
RSc1491	RS03831												PHAGE-RELATED PROTEIN	V.D
RSc1494	RS03811												INTEGRASE-LIKE PROTEIN	V.D
RSc1495	RS03810					-0,79	0,02						HYPOTHETICAL PROTEIN	VI.D
RSc1496	RS03809					-0,98	0,00			4,01	0,02		HYPOTHETICAL PROTEIN	VI.D
RSc1497	RS03808					-0,90	0,00	0,95	0,01				CONSERVED HYPOTHETICAL PROTEIN	VI.C
RSc1498	RS03807					-0,78	0,05						HYPOTHETICAL PROTEIN	VI.D
RSc1499	RS03806					-0,59	0,01						HYPOTHETICAL PROTEIN	VI.D
RSc1500	RS03805			-0,43	0,00	-0,70	0,01						OXIDOREDUCTASE COG0656, Aldo/keto reductases, related to diketogulonate reduc	I
RSc1501	RS03804					-0,77	0,00	0,89	0,00	0,96	0,03		OXIDOREDUCTASE TYROSINASE (MONOPHENOL MONOOXYGENASE)	VI.C
RSc1502	RS03803							0,52	0,04	1,05	0,00		HYPOTHETICAL PROTEIN	VI.D
RSc1503	RS03802							1,10	0,02	1,68	0,00		TRANSMEMBRANE	VI.B
RSc1504	RS03801												COG0589, Universal stress protein UspA and related nucleotide-binding proteins	IV.G
RSc1505	RS03800												OXIDOREDUCTASE COG1064, Zn-dependent alcohol dehydrogenases	I.H
RSc1506	RS03799												TRANSMEMBRANE	VI.C
RSc1507	RS03796												HYPOTHETICAL PROTEIN	VI.D
RSc1508	RS06024			-0,67	0,02								HYPOTHETICAL PROTEIN	VI.D
RSc1509	RS04827					-0,51	0,00						CONSERVED HYPOTHETICAL PROTEIN	VI.C
RSc1510	RS03794					-0,52	0,02						HYPOTHETICAL PROTEIN	VI.D
RSc1511	RS03793			-0,68	0,03								CONSERVED HYPOTHETICAL PROTEIN	VI.C
RSc1512	RS03792									0,58	0,01		HYPOTHETICAL PROTEIN	VI.D
RSc1513	RS03791					-0,37	0,00					rnhA	RIBONUCLEASE HI	II.A.2
RSc1514	RS03790												HYPOTHETICAL PROTEIN	VI.B
RSc1515	RS03789					-0,29	0,02					gloB	HYDROXYACYLGLUTATHIONE HYDROLASE	I.F
RSc1516	RS03787			0,97	0,04	-0,26	0,03						MEMBRANE-BOUND LYTIC MUREIN TRANSGLYCOSYLASE-LIKE LIPOPROTEIN	VI.C
RSc1517	RS03788			-1,97	0,00	2,62	0,00	0,61	0,02				HYPOTHETICAL PROTEIN	VI.D
RSc1518	RS03786											prpE	PROPIONYL-COENZYME A SYNTHETASE PRPE	I.C
RSc1519	RS03785									-1,19	0,00	carA	CARBAMOYL-PHOSPHATE SYNTHASE (SMALL CHAIN)	I.A.17
RSc1520	RS03784									-1,92	0,00		TRANSMEMBRANE	VI.C
RSc1521	RS03783	-0,98	0,01					0,58	0,04			carB	CARBAMOYL-PHOSPHATE SYNTHASE (LARGE CHAIN)	I.A.17
RSc1522	RS03782					-0,33	0,05					greA	TRANSCRIPTION ELONGATION FACTOR (TRANSCRIPT CLEAVAGE FACTOR)	II.B.6
RSc1523	RS03781												TRANSMEMBRANE	VI.C
RSc1524	RS03780					-0,35	0,02	-0,41	0,02				HYPOTHETICAL PROTEIN	VI.B
RSc1525	RS04752					-0,51	0,00					rrmJ	RIBOSOMAL RNA LARGE SUBUNIT METHYLTRANSFERASE J (CELL DIVISION P	II.B.6
RSc1526	RS03778											hflB	TRANSMEMBRANE ATP-DEPENDENT ZINC METALLOPEPTIDASE (CELL DIVISIO	II.A.4
RSc1527	RS03777					-0,37	0,01	-0,85	0,04			folP	7,8-DIHYDROPTEROATE SYNTHASE	I.B.14
RSc1528	RS03776											mrsA	PHOSPHOGLUCOSAMINE MUTASE (MRSA PROTEIN)	II.B.2
RSc1529	RS03775			0,93	0,02	-0,55	0,01					pstS1	ABC TRANSPORTER PHOSPHATE-BINDING PERIPLASMIC PROTEIN (PBP)	I.F.20
RSc1530	RS03774			0,78	0,02							pstC	ABC TRANSPORTER PHOSPHATE TRANSMEMBRANE PROTEIN	I.F.20
RSc1531	RS03773											pstA	ABC TRANSPORTER PHOSPHATE TRANSMEMBRANE PROTEIN	I.F.20
RSc1532	RS03772					-0,44	0,04					pstB	ABC TRANSPORTER PHOSPHATE ATP-BINDING PROTEIN	I.F.20
RSc1533	RS03771											phoU	PHOSPHATE TRANSPORT SYSTEM PROTEIN	I.F.20
RSc1534	RS03769					-0,42	0,02					phoB	TRANSCRIPTION REGULATOR PHOSPHATE REGULON TWO-COMPONENT RES	I.F.20
RSc1535	RS03768							-0,72	0,01			phoR	TRANSCRIPTION REGULATOR PHOSPHATE REGULON TWO-COMPONENT SEN	I.F.20
RSc1536	RS03767					-0,56	0,00	-0,70	0,01			ppk	POLYPHOSPHATE KINASE	I.F.20
RSc1537	RS03765							-0,74	0,02			ppx	EXOPOLYPHOSPHATASE	I.F.20
RSc1538	RS03764					-0,67	0,01						CONSERVED HYPOTHETICAL PROTEIN	VI.C
RSc1539	RS03763					-0,72	0,01						HYDROLASE	VI.C
RSc1540	RS03762			0,59	0,00	-0,56	0,02						TRANSMEMBRANE TRANSFERASE	VI.C
RSc1541	RS03761					-0,56	0,00						TRANSMEMBRANE	VI.C
RSc1542	RS03759					-1,14	0,00						TRANSMEMBRANE	VI.C
RSc1543	RS05230			-0,98	0,02	-0,36	0,02						TRANSCRIPTION REGULATOR	VI.A
RSc1544	RS05231			-0,47	0,03	-0,40	0,01						CONSERVED HYPOTHETICAL PROTEIN	VI.C
RSc1545	RS05232												TRANSMEMBRANE SIGNAL TRANSDUCTION PROTEIN EAL - GGDEF DOMAINS	VI.A
RSc1546	RS05233			0,73	0,02								COG1882, Pyruvate-formate lyase AND COG2837, Predicted iron-dependent peroxid	VI.C
RSc1547	RS05234												HYPOTHETICAL PROTEIN	VI.B
RSc1548	RS05235												TRANSPOSASE-RELATED PROTEIN	V.A
RSc1551	RS05238							1,88	0,00	0,93	0,02		HYPOTHETICAL PROTEIN	VI.D
RSc1552	RS05239							1,05	0,00				HYPOTHETICAL PROTEIN	VI.D
RSc1553	RS05240					-0,54	0,04						TRANSGLYCOLSYLASE	III.B.3
RSc1554	RS05241					-0,49	0,01						INTEGRASE/RECOMBINASE	V.A
RSc1555	RS05242												TRANSMEMBRANE	VI.D
RSc1556	RS05243												OXIDOREDUCTASE	I
RSc1557	RS05244												TRANSMEMBRANE	VI.C
RSc1558	RS05245					-0,47	0,01					wrbA	TRP REPRESSOR BINDING PROTEIN	I.A.6
RSc1559	RS05246							-1,25	0,00			rbn	TRANSMEMBRANE RIBONUCLEASE BN	II.B.6
RSc1560	RS05247					-0,52	0,00						TRANSMEMBRANE	VI.C
RSc1561	RS05248												HYDROLASE	I.I
RSc1562	RS05249			0,70	0,02					0,75	0,05	metY	O-ACETYLHOMOSERINE (THIOL)-LYASE (METHIONINE/HOMOCYSTEINE SYNTH	I.A.12
RSc1563	RS05250					-0,48	0,03						CONSERVED HYPOTHETICAL PROTEIN	VI.C
RSc1564	RS05251			0,54	0,01	-0,34	0,02						TRANSMEMBRANE	VI.C
RSc1565	RS05252			0,38	0,01	-0,49	0,00						SIGNAL PEPTIDE FKBP-TYPE PEPTIDYL-PROLYL CIS-TRANS ISOMERASE	IV.B
RSc1566	RS05253			0,46	0,01					-0,77	0,02	aroC	CHORISMATE SYNTHASE	I.A.5
RSc1567	RS05254			0,75	0,01							etf	OXIDOREDUCTASE ELECTRON TRANSFER FLAVOPROTEIN-UBIQUINONE	I.G.1
RSc1568	RS05255												OXIDOREDUCTASE	I
RSc1569	RS05256												TRANSCRIPTION REGULATOR	VI.A
RSc1570	RS05257					-0,66	0,00					lpsI	FESUCCINYL-COA:3-KETOACID-COENZYME A TRANSFERASE SUBUNIT A	II.B.2
RSc1571	RS05258					-0,61	0,00			-0,97	0,01	lpsJ	SUCCINYL-COA:3-KETOACID-COENZYME A TRANSFERASE SUBUNIT B	II.B.2
RSc1572	RS05259			0,55	0,00	-0,48	0,01						OXIDOREDUCTASE D-BETA-HYDROXYBUTYRATE DEHYDROGENASE (BDH)	I.I.1
RSc1573	RS05260					-0,30	0,00	0,67	0,01				CONSERVED HYPOTHETICAL PROTEIN	VI.C
RSc1574	RS05784					-0,30	0,02						HYDROLASE	I
RSc1575	RS05785												CONSERVED HYPOTHETICAL PROTEIN	VI.C
RSc1576	RS05786			0,34	0,02	-0,28	0,02					relA	GTP PYROPHOSPHOKINASE (ATP:GTP 3'-PYROPHOSPHOTRANSFERASE)	I.F.9
RSc1577	RS05787											thrS	THREONYL-TRNA SYNTHETASE (THREONINE--TRNA LIGASE)	II.D.1
RSc1578	RS05788					-0,35	0,01	-0,34	0,02	-0,91	0,05	infC	TRANSLATION INITIATION FACTOR	II.B.7
RSc1579	RS03941									-0,74	0,00	rpmI	50S RIBOSOMAL SUBUNIT PROTEIN L35 (RIBOSOMAL SUBUNIT PROTEIN A)	III.C.2
RSc1580	RS03942	0,52	0,05							-0,70	0,00	rplT	50S RIBOSOMAL SUBUNIT PROTEIN L20 (AND POSTTRANSCRIPTIONAL AUTOR	III.C.2
RSc1581	RS03943			0,64	0,02					-1,23	0,00	pheS	PHENYLALANYL-TRNA SYNTHETASE ALPHA CHAIN	II.D.1
RSc1582	RS05825			0,75	0,03			0,66	0,03	-1,24	0,00	pheT	PHENYLALANYL-TRNA SYNTHETASE BETA CHAIN	II.D.1
RSc1583	RS03945							-0,37	0,04			ihfA	INTEGRATION HOST FACTOR ALPHA-SUBUNIT (IHF-ALPHA)	II.C.1
RSc1584	RS03946			0,55	0,01	-0,46	0,02						TRANSCRIPTION REGULATOR	VI.A
RSc1585	RS03947					0,26	0,04						CONSERVED HYPOTHETICAL PROTEIN	VI.C
RSc1586	RS03948												LIPOPROTEIN	VI.D
RSc1587	RS03949					-0,50	0,00					dinP	DNA-DAMAGE-INDUCIBLE PROTEIN P	II.B.8
RSc1588	RS03950					0,79	0,00						TRANSMEMBRANE AMINO-ACID TRANSPORTER	IV.D.2
RSc1589	RS03951					0,71	0,02	1,76	0,00				HYPOTHETICAL PROTEIN	VI.B
RSc1590	RS03952												HYPOTHETICAL PROTEIN	VI.B

RSc1591	RS03953					-0,95	0,00					hpal1	OXIDOREDUCTASE 2,4-DIHYDROXYHEPT-2-ENE-1,7-DIOIC ACID ALDOLASE	I.I.4
RSc1592	RS03954					-1,08	0,00						TRANSCRIPTION REGULATOR	VI.A
RSc1593	RS03955											xthA	EXODEOXYRIBONUCLEASE III	II.A.1
RSc1594	RS03956					-0,38	0,03					codA	CYTOSINE DEAMINASE (CYTOSINE AMINOHYDROLASE)	I.F.9
RSc1595	RS03957			0,53	0,01					-0,34	0,02	prlC	OLIGOPEPTIDASE A (OPDA PROTEIN)	II.A.4
RSc1596	RS03958											folD	OXIDOREDUCTASE BIFUNCTIONAL PROTEIN : METHYLENETETRAHYDROFOLA	I.B.14
RSc1597	RS03959			0,31	0,04	-0,41	0,03	-0,74	0,04				TRANSCRIPTION REGULATOR RESPONSE REGULATOR	VI.A
RSc1598	RS03960												TRANSCRIPTION REGULATOR TWO-COMPONENT TRANSMEMBRANE SENSOR	VI.A
RSc1599	RS03961												HYPOTHETICAL PROTEIN	VI.D
RSc1600	RS03962			0,89	0,03	-0,57	0,01			-0,71	0,02	aceE	OXIDOREDUCTASE PYRUVATE DEHYDROGENASE E1 COMPONENT	I.H.7
RSc1601	RS03963			0,78	0,00	-0,65	0,01			-0,73	0,04	aceF	DIHYDROLIPOAMIDE ACETYLTRANSFERASE COMPONENT OF PYRUVATE DEH	I.H.7
RSc1602	RS03964			0,77	0,01	-0,68	0,00			-0,62	0,02		CONSERVED HYPOTHETICAL PROTEIN	VI.C
RSc1603	RS03965			1,12	0,00	-0,70	0,00			-1,28	0,00	lpdA	DIHYDROLIPOAMIDE DEHYDROGENASE (E3 COMPONENT OF PYRUVATE AND	I.H.7
RSc1604	RS03966					-0,68	0,00	-0,45	0,04				TRANSMEMBRANE	VI.D
RSc1605	RS03967							0,85	0,01			phaP1	POLYHYDROXYBUTYRATE GRANULE-ASSOCIATED PROTEIN (PHASIN) PHAP1	II.B
RSc1606	RS03969			0,39	0,05								CONSERVED HYPOTHETICAL PROTEIN	VI.C
RSc1607	RS03968			0,60	0,05	-0,49	0,03						TRANSMEMBRANE	VI.C
RSc1608	RS03971												OXIDOREDUCTASE	VI.C
RSc1609	RS03972												D-ALANYL-D-ALANINE- ENDOPEPTIDASE (PENICILLIN-BINDING PROTEIN)	IV.A
RSc1610	RS03973					-0,50	0,00	-1,08	0,01	-1,15	0,00		TRANSCRIPTION REGULATOR	VI.A
RSc1611	RS03974												CONSERVED HYPOTHETICAL PROTEIN	VI.C
RSc1612	RS03975			1,25	0,02								IRON-SULFUR BINDING PROTEIN	VI.C
RSc1613	RS03976												HYPOTHETICAL PROTEIN	VI.D
RSc1614	RS03977			-0,99	0,01	1,28	0,00						TRANSMEMBRANE	VI.B
RSc1615	RS03978							-0,42	0,05				TRANSMEMBRANE	VI.D
RSc1616	RS03979												CONSERVED HYPOTHETICAL PROTEIN	VI.C
RSc1617	RS03980					-0,84	0,00	-1,13	0,00			aat	LEUCYL/PHENYLALANYL-TRNA--PROTEIN TRANSFERASE	II.A.4
RSc1618	RS03981					-0,38	0,05	0,58	0,03				HYPOTHETICAL PROTEIN	VI.B
RSc1619	RS03982											pyrD	OXIDOREDUCTASE DIHYDROOROTATE DEHYDROGENASE	I.D.1
RSc1620	RS03983					-0,67	0,00						TRANSMEMBRANE	VI.D
RSc1621	RS03984					-0,60	0,00					kup2	TRANSMEMBRANE POTASSIUM UPTAKE PROTEIN	IV.D.4
RSc1622	RS03985									2,33	0,00		TRANSMEMBRANE	VI.D
RSc1623	RS03986			1,07	0,04	-0,53	0,02					tex	TRANSCRIPTION ACCESSORY PROTEIN	VI.C
RSc1624	RS03987					-0,42	0,03						CONSERVED HYPOTHETICAL PROTEIN	VI.C
RSc1625	RS03988					-0,70	0,00	-0,71	0,00				CONSERVED HYPOTHETICAL PROTEIN	VI.C
RSc1626	RS03989												ATP-DEPENDENT DNA HELICASE-RELATED PROTEIN	II.B.8
RSc1627	RS03990									-0,30	0,02		TRANSMEMBRANE	VI.C
RSc1628	RS03991											rluD	RIBOSOMAL LARGE SUBUNIT PSEUDOURIDINE SYNTHASE D	III.C.1
RSc1629	RS03992												CONSERVED HYPOTHETICAL PROTEIN	VI.C
RSc1630	RS03993					-0,45	0,03	-0,73	0,00	-0,70	0,00		TRANSMEMBRANE	VI.D
RSc1631	RS03994			0,57	0,00	-0,38	0,03	0,85	0,02			phbC	TRANSMEMBRANE POLY-BETA-HYDROXYBUTYRATE POLYMERASE	II.B
RSc1632	RS03995			0,63	0,04							phbA	ACETYL-COA ACETYLTRANSFERASE (ACETOACETYL-COA THIOLASE)	II.B
RSc1633	RS03996							0,71	0,04			phbB	OXIDOREDUCTASE ACETOACETYL-COA REDUCTASE	II.B
RSc1634	RS03997							-0,57	0,01				CONSERVED HYPOTHETICAL PROTEIN	VI.C
RSc1635	RS03998												CONSERVED HYPOTHETICAL PROTEIN	VI.C
RSc1636	RS03999			1,54	0,00								CONSERVED HYPOTHETICAL PROTEIN	VI.C
RSc1637	RS05826			0,48	0,01	-0,35	0,01					bktB	BETA-KETOTHIOLASE	II.B
RSc1638	RS04012									1,37	0,02		SIGNAL PEPTIDE	VI.D
RSc1639	RS04013			0,65	0,00					0,37	0,01	metC	CYSTATHIONINE BETA-LYASE (CYSTEINE LYASE)	I.A.12
RSc1640	RS04014	-0,61	0,02	1,26	0,01							serB	PHOSPHOSERINE PHOSPHATASE	I.A.10
RSc1641	RS04016							0,87	0,05			argE	ACETYLORNITHINE DEACETYLASE (ACETYLORNITHINASE)	I.A.17
RSc1642	RS04017			0,77	0,00							mfd	TRANSCRIPTION-REPAIR COUPLING FACTOR	II.B.6
RSc1643	RS04018			0,92	0,02								CONSERVED HYPOTHETICAL PROTEIN	VI.C
RSc1644	RS04019			0,61	0,00	-0,36	0,01						CONSERVED HYPOTHETICAL PROTEIN	VI.C
RSc1645	RS04020											ahpD	ANTIOXIDANT PROTEIN	IV.G.4
RSc1646	RS04022											ahpC2	OXIDOREDUCTASE ALKYL HYDROPEROXIDE REDUCTASE (SUBUNIT C)	IV.G.4
RSc1647	RS04023			0,62	0,01							risS	TRANSCRIPTION REGULATOR OXIDATIVE STRESS RESISTANCE TWO-COMPOI	IV.G.4
RSc1648	RS04025					-0,38	0,00					risA	TRANSCRIPTION REGULATOR OXIDATIVE STRESS RESISTANCE TWO-COMPOI	IV.G.4
RSc1649	RS04026					-0,68	0,00			1,48	0,00		TRANSMEMBRANE	VI.D
RSc1650	RS04027							1,15	0,03	2,13	0,00		SIGNAL PEPTIDE	VI.B
RSc1651	RS04028					-0,19	0,00	1,62	0,00	2,14	0,00		CONSERVED HYPOTHETICAL PROTEIN	VI.C
RSc1652	RS04029			0,73	0,01	-0,48	0,00					crtB	PHYTOENE SYNTHASE	I
RSc1653	RS04030			0,66	0,00	-0,43	0,00						TRANSMEMBRANE TRANSPORT/EFFLUX PROTEIN	IV.D
RSc1654	RS04031									0,58	0,03		TRANSMEMBRANE DRUG EFFLUX PROTEIN	IV.D
RSc1655	RS04034					-0,27	0,04						INTEGRASE/RECOMBINASE	V.A
RSc1656	RS04036												HYPOTHETICAL PROTEIN	VI.B
RSc1657	RS04037												HYPOTHETICAL PROTEIN	VI.B
RSc1658	RS04038												JAMM ISOPEPTIDASE	VI.B
RSc1659	RS04040												HYPOTHETICAL PROTEIN	VI.D
RSc1660	RS04041			-0,88	0,03								HYPOTHETICAL PROTEIN	VI.D
RSc1661	RS04042												HYPOTHETICAL PROTEIN	VI.D
RSc1662	RS04043					-0,54	0,04						HYPOTHETICAL PROTEIN	VI.D
RSc1663	RS04044												HYPOTHETICAL PROTEIN	VI.D
RSc1664	RS04045							-1,32	0,01				HYPOTHETICAL PROTEIN	VI.D
RSc1665	RS04046												HYPOTHETICAL PROTEIN	VI.D
RSc1666	RS04047												TRANSMEMBRANE	VI.D
RSc1667	RS04048					-1,23	0,01						HYPOTHETICAL PROTEIN	VI.D
RSc1668	RS04049												HYPOTHETICAL PROTEIN	VI.D
RSc1669	RS04051							-2,00	0,05				TRANSMEMBRANE	VI.D
RSc1670	RS04057			0,79	0,00	-0,68	0,00						HYPOTHETICAL PROTEIN	VI.D
RSc1673	RS04060					-1,01	0,00	-1,06	0,05	-2,93	0,00		HYPOTHETICAL PROTEIN	VI.D
RSc1674	RS04062					-0,52	0,03						TRANSCRIPTION REGULATOR	VI.A
RSc1675	RS04063												HYPOTHETICAL PROTEIN	VI.D
RSc1676	RS04064			-0,68	0,01	-0,44	0,00			2,81	0,00		HYPOTHETICAL PROTEIN	VI.D
RSc1677	RS04065												TRANSMEMBRANE	VI.D
RSc1678	RS04069												HYPOTHETICAL PROTEIN	VI.D
RSc1679	RS04071												HYPOTHETICAL PROTEIN	VI.D
RSc1680	RS04072												PHAGE PHI-105 HOLIN-LIKE PROTEIN	V.D
RSc1681	RS04075												TRANSMEMBRANE	VI.D
RSc1682	RS04076												PHAGE PHI-105 ORF25-LIKE PROTEIN	V.D
RSc1683	RS04077			-1,53	0,03	1,49	0,01						PHAGE PHI-105 ORF26-LIKE PROTEIN	V.D
RSc1684	RS04078												PHAGE PHI-C31 GP36-LIKE PROTEIN	V.D
RSc1685	RS04079			-1,71	0,04								BACTEIOPHAGE-RELATED PROTEIN	V.D
RSc1686	RS04080												phage HK022 GP9-RELATED PROTEIN	V.D
RSc1687	RS04081												HYPOTHETICAL PROTEIN	VI.B
RSc1688	RS04082												HYPOTHETICAL PROTEIN	VI.B
RSc1689	RS04083												HYPOTHETICAL PROTEIN	VI.B
RSc1690	RS04086												PHAGE HK97 TAIL LENGTH TAPE MEASURE-RELATED PROTEIN	V.D
RSc1691	RS04087												PHAGE HK97 GP17-RELATED PROTEIN	V.D
RSc1692	RS04088												HYPOTHETICAL PROTEIN	VI.B
RSc1693	RS04089							-1,66	0,01				TAIL FIBER ASSEMBLY PROTEIN HOMOLOG	V.D
RSc1694	RS04090												PHAGE HK022 GP18-RELATED PROTEIN	V.D
RSc1695	RS04091												PHAGE HK022 GP19-RELATED PROTEIN	V.D
RSc1696	RS04092												PHAGE HK022 GP20-RELATED PROTEIN	V.D
RSc1697	RS04093					-0,51	0,00			-0,67	0,00		CONSERVED HYPOTHETICAL PROTEIN	VI.C
RSc1698	RS02885					-0,46	0,01						BACTERIOPHAGE PROTEIN	V.D
RSc1699	RS02888			0,74	0,05	-0,43	0,05	-0,71	0,03				BACTERIOPHAGE PROTEIN	V.D
RSc1700	RS02889												BACTERIOPHAGE ENDOLYSIN	V.D
RSc1701	RS02890												TRANSMEMBRANE LIPOPROTEIN	VI.D
RSc1702	RS02891					-0,55	0,00	0,64	0,04	1,49	0,01		TRANSMEMBRANE	VI.D
RSc1703	RS02892												SIGNAL PEPTIDE BACTERIOPHAGE-RELATED PROTEIN	V.D
RSc1704	RS02893					-0,41	0,00	0,34	0,04			tISRso1	ISRso18-TRANSPOSASE	V.A

RSc1705	RS02898			1,61	0,04								HYPOTHETICAL PROTEIN	VI.D
RSc1706	RS02899							0,51	0,05	0,85	0,00		HYPOTHETICAL PROTEIN	VI.D
RSc1707	RS02900			0,57	0,04			0,71	0,00	0,92	0,01		TRANSMEMBRANE	VI.D
RSc1708	RS02894					-0,79	0,00	-0,59	0,01	1,66	0,00		HYPOTHETICAL PROTEIN	VI.D
RSc1709	RS02895					-0,48	0,00						LIPOPROTEIN	VI.C
RSc1710	RS02896			0,45	0,04	-0,27	0,01			-0,47	0,02	tig	PEPTIDYL-PROLYL CIS/TRANS ISOMERASE (TRIGGER FACTOR)	IV.A
RSc1711	RS02901			0,49	0,01	-0,36	0,02	0,45	0,03			clpP	TRANSMEMBRANE ATP-DEPENDENT PROTEASE (PROTEOLYTIC SUBUNIT)	II.A.4
RSc1712	RS02902			0,29	0,03	-0,31	0,05					clpX	ATP-DEPENDENT PROTEASE (ATP-BINDING SPECIFICITY SUBUNIT)	II.A.4
RSc1713	RS02903			0,55	0,01							lon	ATP-DEPENDENT PROTEASE LA	II.A.4
RSc1714	RS02904					-0,32	0,03					hupB1	DNA-BINDING PROTEIN HU-BETA	II.C.1
RSc1715	RS02905			0,78	0,01	-0,36	0,00			-0,78	0,00	ppiD	TRANSMEMBRANE PEPTIDYL-PROLYL CIS-TRANS ISOMERASE	IV.B
RSc1716	RS02906												SIGNAL PEPTIDASE I (SPASE I)	IV.E.2
RSc1717	RS02907					-0,48	0,00			0,05	0,03		SIGNAL PEPTIDE ESTERASE	I.I.1
RSc1718	RS02908												ABC TRANSPORTER ATP-BINDING PROTEIN	IV.D
RSc1719	RS02909											pgi	TRANSMEMBRANE GLUCOSE-6-PHOSPHATE ISOMERASE	I.H.8
RSc1720	RS02910												COG0063, Predicted sugar kinase	I
RSc1721	RS02911					-0,29	0,00						TRANSCRIPTION REGULATOR	VI.A
RSc1722	RS02912			0,72	0,00			1,08	0,04	-1,99	0,00	purL	PHOSPHORIBOSYLFORMYLGLYCINAMIDINE SYNTHASE	I.D.2
RSc1723	RS06010			-4,69	0,00	5,19	0,00	6,07	0,01				HYPOTHETICAL PROTEIN	VI.D
RSc1724	RS04177					0,37	0,01			2,20	0,00		NOTHING	
RSc1725	RS02913					0,31	0,03	1,79	0,00	4,00	0,00		HYPOTHETICAL PROTEIN	VI.D
RSc1726	RS02914							2,21	0,00	3,96	0,00		CONSERVED HYPOTHETICAL PROTEIN	VI.C
RSc1727	RS02915							1,85	0,00	3,81	0,00		CONSERVED HYPOTHETICAL PROTEIN	VI.C
RSc1728	RS02916			1,22	0,00	0,86	0,00	1,42	0,00				OXIDOREDUCTASE COG535, PREDICTED Fe-S OXIDOREDUCTASE	VI.B
RSc1729	RS02917					-0,41	0,01			0,84	0,04		TRANSCRIPTION REGULATOR	VI.A
RSc1730	RS02918					-0,40	0,02						OXIDOREDUCTASE COG1028, Dehydrogenases with different specificities (related to	I
RSc1731	RS02919					-0,39	0,01						ABC TRANSPORTER TRANSMEMBRANE PROTEIN	IV.D
RSc1732	RS02920					-1,06	0,02						ABC TRANSPORTER COG1176, ABC-type spermidine/putrescine transport system, p	IV.D.2
RSc1733	RS02921					-0,43	0,00	1,54	0,00	3,44	0,00		SIGNAL PEPTIDE COG0687, Spermidine/putrescine-binding periplasmic protein	IV.D.2
RSc1734	RS02922					-1,25	0,00						ABC TRANSPORTER ATP-BINDING PROTEIN	IV.D
RSc1735	RS02923					-1,47	0,00						TRANSCRIPTION REGULATOR TWO-COMPONENT TRANSMEMBRANE SENSOR	VI.A
RSc1736	RS02924					-0,53	0,04						TRANSCRIPTION REGULATOR TWO-COMPONENT RESPONSE REGULATOR	VI.A
RSc1737	RS02925												SIGNAL PEPTIDE TRANSMEMBRANE OUTER MEMBRANE PORIN	IV.D
RSc1738	RS02926			0,98	0,00			-1,61	0,04				ABC TRANSPORTER ATP-BINDING PROTEIN	IV.D
RSc1739	RS02927												ABC TRANSPORTER TRANSMEMBRANE PROTEIN	IV.D
RSc1740	RS02928			0,76	0,02	-0,63	0,03						ABC TRANSPORTER COG2984, ABC-type uncharacterized transport system, peripla	IV.D
RSc1741	RS02929												TRANSCRIPTION REGULATOR COG1737, Transcriptional regulator	VI.A
RSc1742	RS02930												CONSERVED HYPOTHETICAL PROTEIN	VI.C
RSc1743	RS02931					-0,93	0,00	-4,26	0,00				HYPOTHETICAL PROTEIN	VI.B
RSc1744	RS02932							-0,62	0,00	-0,98	0,00		SIGNAL PEPTIDE ISOMERASE ROTAMASE	IV
RSc1745	RS02933							-0,20	0,03				TRANSCRIPTION REGULATOR	VI.A
RSc1746	RS02934			0,69	0,00							ispZ	TRANSMEMBRANE INTRACELLULAR SEPTATION PROTEIN	IV.A
RSc1747	RS02935												COG0229, Conserved domain frequently associated with peptide methionine sulfoxide	VI.C
RSc1748	RS02936												COG0397, Uncharacterized conserved protein	VI.C
RSc1749	RS03026					-1,26	0,01					alkK	ACYL-COA SYNTHETASE	I.C
RSc1750	RS02938											livJ	SIGNAL PEPTIDE LEU/ILE/VAL-BINDING PROTEIN PRECURSOR	IV.D.2
RSc1751	RS02939											livH1	TRANSMEMBRANE AMINO ACID TRANSMEMBRANE PROTEIN	IV.D.2
RSc1752	RS02940											livM	TRANSMEMBRANE AMINO ACID TRANSPORT SYSTEM PERMEASE LIPOPROTE	IV.D.2
RSc1753	RS02941					-0,35	0,03					livG	ABC TRANSPORTER AMINO-ACID ATP-BINDING PROTEIN	IV.D.2
RSc1754	RS02942			0,90	0,02	-0,40	0,05					livF	ABC TRANSPORTER AMINO-ACID ATP-BINDING PROTEIN	IV.D.2
RSc1755	RS06012												TRANSPOSASE	V.A
RSc1756	RS02943	-3,09	0,00			-0,51	0,00	2,08	0,00	3,10	0,00	pehB	SIGNAL PEPTIDE EXO-POLY-GALACTURONASE	II.A.3
RSc1757	RS02944			0,82	0,00								TRANSMEMBRANE LYSINE-SPECIFIC PERMEASE	IV.D.2
RSc1758	RS02945					-0,63	0,00					lysP	TRANSMEMBRANE LYSINE-SPECIFIC PERMEASE	IV.D.2
RSc1759	RS02946					-1,24	0,00	-1,09	0,00	-0,26	0,03		OXIDOREDUCTASE TRIFUNCTONAL PROTEIN: ENOYL-CoA HYDRATASE AND D	I.I.1
RSc1760	RS02947					-0,87	0,00					pncA	BIFUNCTIONAL PROTEIN: PYRAZINAMIDASE AND NICOTINAMIDASE HYDROLA	I.B.9
RSc1761	RS02948					-0,96	0,00						ACETYL-COA ACETYLTRANSFERASE	I.C
RSc1762	RS02949					-0,56	0,04						OXIDOREDUCTASE ACYL-CoA DEHYDROGENASE	I.I.1
RSc1763	RS02950	0,50	0,03	0,80	0,03	-0,56	0,00						OXIDOREDUCTASE DEHYDROGENASE	I
RSc1764	RS02951							-0,63	0,05				CONSERVED HYPOTHETICAL PROTEIN	VI.C
RSc1765	RS03025			0,58	0,01								6-AMINOHEXANOATE-DIMER HYDROLASE LIPOPROTEIN	I.I.2
RSc1766	RS02953			0,56	0,01								OXIDOREDUCTASE NADP-DEPENDENT OXIDOREDUCTASE	I
RSc1767	RS02954			0,53	0,02								COG0235, Ribulose-5-phosphate 4-epimerase and related epimerases and aldolases	I
RSc1768	RS02955			0,55	0,00							gstG	GLUTATHIONE S-TRANSFERASE	II.B.7
RSc1769	RS02956			0,27	0,05	-0,32	0,05						SIGNAL PEPTIDE OXIDOREDUCTASE DEHYDROGENASE	I
RSc1770	RS02957												COG0596, Predicted hydrolases or acyltransferases (alpha/beta hydrolase superfamil	I
RSc1771	RS02958											gbd1	OXIDOREDUCTASE NAD-DEPENDENT 4-HYDROXYBUTYRATE DEHYDROGENAS	I.I.4
RSc1772	RS02959					-0,40	0,05						ESTERASE/LIPASE	I.I.1
RSc1773	RS02960												THIOESTERASE	I
RSc1774	RS02961												COG2153, Predicted acetyltransferase	I
RSc1775	RS04371												HEMAGGLUTININ-RELATED PROTEIN	VI.B
RSc1776	RS04372			-0,99	0,01	1,45	0,00						HYPOTHETICAL PROTEIN	VI.D
RSc1777	RS04160			0,81	0,00			-1,20	0,00	-1,84	0,00		TRANSMEMBRANE LIPOPROTEIN	VI.C
RSc1778	RS04374	-0,51	0,05					-1,67	0,03	-1,34	0,02		TRANSMEMBRANE	VI.D
RSc1779	RS04166												SIGNAL PEPTIDE ACTIVATION/SECRETION PROTEIN	IV.E.2
RSc1780	RS04168					-0,64	0,01						SIGNAL PEPTIDE	VI.C
RSc1781	RS04171									1,64	0,00		CONSERVED HYPOTHETICAL PROTEIN	VI.C
RSc1782	RS04173			0,57	0,00	-0,21	0,04			-0,83	0,02		COG0354, Predicted aminomethyltransferase	I
RSc1783	RS06023					0,46	0,02			-1,31	0,02		CONSERVED HYPOTHETICAL PROTEIN	VI.C
RSc1784	RS04182			0,54	0,05							tmk	THYMIDYLATE KINASE (DTMP KINASE)	I.F.2
RSc1785	RS04183			1,40	0,03							holB	DNA POLYMERASE III (DELTA' SUBUNIT)	II.B.8
RSc1786	RS04185											pilZ	TYPE 4 FIMBRIAL BIOGENESIS PROTEIN	III.A.2
RSc1787	RS04186			0,55	0,01	-0,27	0,01			-0,97	0,00		COG0084, Mg-dependent deoxyribonuclease	II.A.1
RSc1788	RS04187							0,96	0,01	-0,84	0,00		SIGNAL PEPTIDE ANKYRIN REPEAT HARBORING PROTEIN	VI.C
RSc1789	RS04188												CONSERVED HYPOTHETICAL PROTEIN	VI.C
RSc1790	RS04189							-1,61	0,00				TRANSCRIPTION REGULATOR	VI.A
RSc1791	RS04190												ABC TRANSPORTER SOLUTE-BINDING PERIPLASMIC PROTEIN (PBP)	IV.D
RSc1792	RS04191												ABC TRANSPORTER TRANSPORT SYSTEM PERMEASE TRANSMEMBRANE PRO	IV.D
RSc1793	RS04192												ABC TRANSPORTER PERMEASE TRANSMEMBRANE PROTEIN	IV.D
RSc1794	RS04193					0,99	0,01						ABC TRANSPORTER ATP-BINDING PROTEIN	IV.D
RSc1795	RS04195												COG1409, Predicted phosphohydrolase	I
RSc1796	RS04196					0,24	0,05						N-ACETYLMURAMOYL-L-ALANINE AMIDASE	III.B.3
RSc1797	RS04198					-0,68	0,01	-0,60	0,03				OXIDOREDUCTASE PYRUVATE DEHYDROGENASE E1 COMPONENT (ALPHA SU	I.I.4
RSc1798	RS04199							1,66	0,01				OXIDOREDUCTASE PYRUVATE DECARBOXYLASE E1 (BETA SUBUNIT)	I
RSc1799	RS04200												DIHYDROLIPOAMIDE ACETYLTRANSFERASE (COMPONENT E2 OF PYRUVATE	I.I.4
RSc1800	RS04201	-2,37	0,01	-5,50	0,00	6,18	0,00	3,70	0,00				LRR-GALA FAMILY TYPE III EFFECTOR PROTEIN (GALA 4)	VI.C
RSc1801	RS04202			-1,43	0,00	2,22	0,00	4,06	0,00				LRR-GALA FAMILY TYPE III EFFECTOR PROTEIN (GALA 5)	VI.C
RSc1802	RS04203									-0,51	0,01		TRANSCRIPTION REGULATOR	VI.A
RSc1803	RS04222												TRANSMEMBRANE COG0038, CHLORIDE CHANNEL PROTEIN	IV.D.1
RSc1804	RS04224			-2,90	0,01	-2,05	0,00			-4,64	0,00		PEPTIDE SYNTHASE WITH THIOESTERASE AND PHOSPHOPANTETHEINYL TR/	I
RSc1805	RS04226			-2,81	0,01	-2,06	0,00	-2,25	0,01	-3,98	0,00		SIDEROPHORE-LIKE SYNTHASE	I
RSc1806	RS04228									-1,21	0,00		POLYKETIDE SYNTHASE	I
RSc1807	RS04230			-2,17	0,01	-1,11	0,00	-1,25	0,00	-5,34	0,00		SIDEROPHORE RECEPTOR PROTEIN	IV.D.5
RSc1808	RS04232			-2,26	0,04	-1,30	0,00	-1,41	0,05	-3,97	0,00		ABC TRANSPORTER COMPOSITE ATP-BINDING TRANSMEMBRANE PROTEIN	IV.D
RSc1809	RS04233			-1,79	0,04	-0,99	0,02	-1,23	0,01	-4,88	0,00		ABC TRANSPORTER COMPOSITE ATP-BINDING TRANSMEMBRANE PROTEIN	IV.D
RSc1810	RS04235					-2,47	0,00			-2,04	0,00		POLYKETIDE SYNTHASE	I
RSc1811	RS04237			-1,08	0,02	-2,50	0,00	-0,79	0,02	-2,86	0,00		SIDEROPHORE SYNTHETASE	IV.D.5
RSc1812	RS04238	-0,57	0,00	-1,06	0,06	-3,62	0,00	-0,84	0,01	-2,65	0,00		CONSERVED HYPOTHETICAL PROTEIN	VI.C
RSc1813	RS04239			-0,97	0,01	-2,02	0,00	-0,61	0,04	-2,82	0,00		TRANSCRIPTION REGULATOR	VI.A
RSc1814	RS06019												HYPOTHETICAL PROTEIN	VI.D
RSc1815	RS04241			-3,93	0,00	5,24	0,00	4,12	0,00				AVRBS3 FAMILY TYPE III EFFECTOR PROTEIN	VI.C
RSc1816	RS04242					1,29	0,00						TRANSMEMBRANE COG0456, Acetyltransferase	I

RSc1817	RS04244		-0,42 0,05	1,55 0,00		-1,26 0,03	iucD	OXIDOREDUCTASE L-lysine 6-monooxygenase (Lysine N(6)-hydroxylase)	IV.D.5
RSc1818	RS04246			1,47 0,00	1,20 0,02	-1,06 0,03	metG2	METHIONYL-tRNA SYNTHETASE	II.D.1
RSc1819	RS04247			1,56 0,00	0,94 0,01			COG0662, Mannose-6-phosphate isomerase	I
RSc1820	RS04248							COG1853, Conserved protein/domain typically associated with flavoprotein oxygenase	VI.C
RSc1821	RS04250			-0,41 0,01				TRANSCRIPTION REGULATOR	VI.A
RSc1822	RS04251							MALEATE CIS-TRANS ISOMERASE	I
RSc1823	RS04252				-1,26 0,03			COG0388, Predicted amidohydrolase	I
RSc1824	RS04254							COG0179, 2-keto-4-pentenoate hydratase/2-oxohepta-3-ene-1,7-dioic acid hydratase	I
RSc1825	RS04255							OXIDOREDUCTASE COG3435, Gentisate 1,2-dioxygenase	I
RSc1826	RS04256							OXIDOREDUCTASE COG1902, NADH:flavin oxidoreductases AND COG0654, 2-poly	I
RSc1827	RS04258		0,57 0,00					COG0824, Predicted thioesterase	I
RSc1828	RS04259							OXIDOREDUCTASE Indolepyruvate:ferredoxin oxidoreductase	I
RSc1829	RS04261			0,84 0,03				OXIDOREDUCTASE COG4231, Indolepyruvate ferredoxin oxidoreductase, alpha and	I
RSc1832	RS04267							TRANSCRIPTION REGULATOR	VI.A
RSc1833	RS04268							SIGNAL PEPTIDE COG4313, Protein involved in meta-pathway of phenol degradation	I.I.4
RSc1834	RS04270							TRANSMEMBRANE COG1233, Phytoene dehydrogenase and related proteins	I
RSc1835	RS04271							COG1878, Predicted metal-dependent hydrolase	I
RSc1836	RS04273							,COG1853, Conserved protein/domain typically associated with flavoprotein oxygenas	I
RSc1837	RS04274							OXIDOREDUCTASE COG0654, 2-polyprenyl-6-methoxyphenol hydroxylase and relat	I
RSc1838	RS04275							OXIDOREDUCTASE	I
RSc1839	RS04276			1,22 0,01				SKWP PROTEIN 4	VI.C
RSc1840	RS04278							AMINOTRANSFERASE	I
RSc1841	RS04279		-1,80 0,01	2,33 0,00				COG0596, Hydrolase or acyltransferase	I
RSc1842	RS04283							TRANSPOSASE	V.A
RSc1843	RS05589			-0,39 0,02				TRANSPOSASE	V.A
RSc1844	RS05971			-0,44 0,01				HYPOTHETICAL PROTEIN	VI.D
RSc1845	RS05972		0,26 0,03	-0,22 0,02				COG4688, Uncharacterized protein conserved in bacteria	VI.C
RSc1846	RS05973		0,80 0,00					INTEGRASE/RECOMBINASE	V.A
RSc1847	RS05974			-0,25 0,01				INTEGRASE / RECOMBINASE	V.A
RSc1848	RS05597			1,17 0,00	-1,70 0,01			TRANSMEMBRANE	VI.C
RSc1849	RS05598			-0,46 0,05				TRANSCRIPTION REGULATOR	VI.A
RSc1850	RS05599				-1,10 0,04			TRANSMEMBRANE HYPOTHETICAL	VI.B
RSc1851	RS05601			-0,39 0,02				TRANSCRIPTION REGULATOR	VI.A
RSc1852	RS05603							TRANSMEMBRANE MULTIDRUG RESISTANCE PROTEIN	IV.G.2
RSc1853	RS05604							TRANSMEMBRANE MULTIDRUG RESISTANCE PROTEIN	IV.D
RSc1854	RS05605							OUTER MEMBRANE MULTIDRUG RESISTANCE LIPOPROTEIN	IV.D
RSc1855	RS06005							HYPOTHETICAL PROTEIN	VI.D
RSc1856	RS03411							SIGNAL PEPTIDE	VI.C
RSc1857	RS03409		0,48 0,01	-0,23 0,02				TRANSCRIPTION REGULATOR COG1167, Transcriptional regulators containing a DI	VI.A
RSc1858	RS03418						cyoA2	OXIDOREDUCTASE TRANSMEMBRANE CYTOCHROME O UBIQUINOL OXIDASE	I.H.3
RSc1859	RS03419						cyoB2	OXIDOREDUCTASE TRANSMEMBRANE CYTOCHROME O UBIQUINOL OXIDASE	I.H.3
RSc1860	RS03420						cyoC2	OXIDOREDUCTASE TRANSMEMBRANE CYTOCHROME O UBIQUINOL OXIDASE	I.H.3
RSc1861	RS03421		0,74 0,01				cyoD2	OXIDOREDUCTASE TRANSMEMBRANE CYTOCHROME O UBIQUINOL OXIDASE	I.H.3
RSc1862	RS03422						cysI2	OXIDOREDUCTASE SULFITE REDUCTASE	I.F.3
RSc1863	RS03423	5,13 0,00		-0,95 0,00				CONSERVED HYPOTHETICAL PROTEIN	VI.C
RSc1865	RS03425			-0,53 0,04				CONSERVED HYPOTHETICAL PROTEIN	VI.C
RSc1866	RS03426			-0,61 0,01				HYPOTHETICAL PROTEIN	VI.D
RSc1867	RS03427			-0,27 0,01				HYPOTHETICAL PROTEIN	VI.D
RSc1868	RS03428			-0,33 0,00				CONSERVED HYPOTHETICAL PROTEIN	VI.C
RSc1869	RS03429			-0,41 0,01				HYPOTHETICAL PROTEIN	VI.D
RSc1870	RS06006							HYPOTHETICAL PROTEIN	VI.B
RSc1871	RS03431		-0,55 0,01					INTEGRASE PROPHAGE	V.D
RSc1872	RS03432		0,43 0,02	-0,34 0,00			dnaQ	DNA POLYMERASE III (EPSILON CHAIN)	II.B.8
RSc1873	RS03433		0,74 0,01	-0,27 0,00			purU1	FORMYLTETRAHYDROFOLATE DEFORMYLASE	I.D.2
RSc1874	RS03434			-0,50 0,01				CONSERVED HYPOTHETICAL PROTEIN	VI.C
RSc1875	RS03435							CONSERVED HYPOTHETICAL PROTEIN	VI.C
RSc1876	RS03436	-0,59 0,05	-0,82 0,03	-0,53 0,03				HYPOTHETICAL PROTEIN	VI.D
RSc1877	RS03437							TRANSMEMBRANE COG3555, Aspartyl/asparaginyl beta-hydroxylase and related dio	II.B.7
RSc1878	RS03438			-0,55 0,03	0,39 0,01			LIPOPROTEIN	VI.C
RSc1879	RS03439	0,78 0,02				-1,91 0,00		ABC TRANSPORTER ABC-type branched-chain amino acid transport systems, peripla	IV.D.2
RSc1880	RS03440			-0,48 0,03				TRANSCRIPTION REGULATOR	VI.A
RSc1881	RS03441							TRANSMEMBRANE COG2072, Predicted flavoprotein involved in K+ transport	IV.D
RSc1882	RS03442		0,85 0,00				dppB2	ABC TRANSPORTER DIPEPTIDE TRANSMEMBRANE PROTEIN	IV.D.7
RSc1883	RS03443						dppC2	ABC TRANSPORTER DIPEPTIDE TRANSMEMBRANE PROTEIN	IV.D.7
RSc1884	RS03444			-0,36 0,05			dppD2	ABC TRANSPORTER OLIGOPEPTIDE ATP-BINDING PROTEIN	IV.D.7
RSc1885	RS03446		0,44 0,04	-0,51 0,00			dppF2	ABC TRANSPORTER OLIGOPEPTIDE ATP-BINDING PROTEIN	IV.D.7
RSc1886	RS03448				-0,26 0,05		dppA2	PERIPLASMIC DIPEPTIDE TRANSPORT PROTEIN PRECURSOR	IV.D.7
RSc1887	RS03449							HYPOTHETICAL PROTEIN	VI.B
RSc1888	RS03450							CONSERVED HYPOTHETICAL PROTEIN	VI.C
RSc1889	RS03451		1,15 0,01	-0,33 0,04			iorB2	OXIDOREDUCTASE TRANSMEMBRANE ISOQUINOLINE 1-OXIDOREDUCTASE (B	I.I.4
RSc1890	RS03452						iorA2	OXIDOREDUCTASE ISOQUINOLINE 1-OXIDOREDUCTASE (ALPHA SUBUNIT)	I.I.4
RSc1891	RS03453							HYPOTHETICAL PROTEIN	VI.D
RSc1892	RS03454		0,93 0,00	-2,89 0,00	-1,28 0,00			TRANSCRIPTION REGULATOR TWO-COMPONENT SYSTEM RESPONSE REGUL	VI.A
RSc1893	RS03455		0,46 0,01	-0,55 0,00	-0,34 0,03			TRANSMEMBRANE SIGNAL TRANSDUCTION PROTEIN GGDEF DOMAIN	VI.A
RSc1894	RS03456			-0,92 0,03				TRANSMEMBRANE METHYL-ACCEPTING CHEMOTAXIS TRANSDUCER PROTEIN	IV.C
RSc1895	RS03457		0,89 0,00	-0,24 0,01				TRANSCRIPTION REGULATOR	VI.A
RSc1896	RS03458	0,42 0,04				-1,44 0,05		INTEGRASE PROPHAGE	V.D
RSc1897	RS03459							TRANSCRIPTION REGULATOR PROPHAGE REGULATORY PROTEIN	V.D
RSc1898	RS03464							PHAGE-RELATED PROTEIN	V.D
RSc1899	RS03466			-1,32 0,00	-1,75 0,01			HYPOTHETICAL PROTEIN	VI.D
RSc1900	RS03467		-4,07 0,01	-0,48 0,04				TRANSMEMBRANE	VI.D
RSc1901	RS03468		-2,01 0,00	1,53 0,00				TRANSMEMBRANE	VI.D
RSc1902	RS03472			-0,87 0,00				HYPOTHETICAL PROTEIN	VI.D
RSc1903	RS03469			-1,41 0,00				HYPOTHETICAL PROTEIN	VI.D
RSc1904	RS03470			-1,25 0,00	-1,20 0,00	-1,87 0,00		TRANSCRIPTION REGULATOR TRANSCRIPTIONAL ACTIVATOR	V.D
RSc1905	RS03474			-1,21 0,00	-1,98 0,01			HYPOTHETICAL PROTEIN	VI.D
RSc1906	RS03475			-1,83 0,00	-2,46 0,00	-1,14 0,01		HYPOTHETICAL PROTEIN	VI.D
RSc1907	RS03481			-0,43 0,02				TRANSCRIPTION REGULATOR DNA-BINDING REPRESSOR	VI.C
RSc1908	RS03476			-0,46 0,01				SIGNAL PEPTIDE	VI.D
RSc1910	RS03479					2,01 0,00		TRANSMEMBRANE	VI.D
RSc1911	RS03480							DNA-METHYLTRANSFERASE	II.B.8
RSc1912	RS03471							PHAGE-RELATED PROTEIN	V.D
RSc1913	RS03482							PHAGE-RELATED TAIL PROTEIN	V.D
RSc1914	RS03483							TRANSMEMBRANE PHAGE-RELATED TAIL PROTEIN	V.D
RSc1915	RS03485			-1,29 0,00				PHAGE-RELATED PROTEIN FRAGMENT	V.D
RSc1916	RS03484							PHAGE-RELATED PROTEIN	V.D
RSc1917	RS03486			-2,50 0,01				PHAGE-RELATED PROTEIN (MAJOR TAIL TUBE-LIKE PROTEIN)	V.D
RSc1918	RS03487			-2,36 0,00	-1,07 0,03			PHAGE-RELATED PROTEIN (TAIL SHEATH-LIKE PROTEIN)	V.D
RSc1919	RS03488			-0,89 0,04				HYPOTHETICAL PROTEIN	VI.B
RSc1920	RS03489							TAIL FIBER ASSEMBLY-LIKE PROTEIN	V.D
RSc1921	RS03490		0,54 0,01	-1,20 0,01				TAIL FIBER-RELATED PROTEIN	V.D
RSc1922	RS03491							TAIL-RELATED PROTEIN (TAIL FORMATION-LIKE PROTEIN)	V.D
RSc1923	RS03492			-1,15 0,00				BASEPLATE ASSEMBLY-LIKE PROTEIN	V.D
RSc1924	RS03493							PHAGE-RELATED PROTEIN (BASEPLATE ASSEMBLY-LIKE PROTEIN)	V.D
RSc1925	RS03494			-1,43 0,00	-1,17 0,04			PHAGE-RELATED PROTEIN (BASEPLATE ASSEMBLY-LIKE PROTEIN)	V.D
RSc1926	RS03496	0,71 0,01		-1,72 0,00		2,13 0,00		TRANSMEMBRANE PHAGE-RELATED PROTEIN	V.D
RSc1927	RS03497		0,33 0,05	-1,75 0,00		1,85 0,00		HYPOTHETICAL PROTEIN	VI.B
RSc1928	RS03495							TAIL COMPLETION-LIKE PROTEIN	V.D
RSc1929	RS03499							TAIL COMPLETION-LIKE PROTEIN	V.D
RSc1930	RS03498		1,22 0,01	-0,60 0,03				SIGNAL PEPTIDE	VI.D
RSc1931	RS03500			-1,15 0,01				PHAGE-RELATED PROTEIN (HYDROLASE)	V.D
RSc1932	RS03501							TRANSMEMBRANE PHAGE-RELATED PROTEIN	V.D

RSc1933	RS03502					-0,73	0,01						TRANSMEMBRANE PHAGE-RELATED PROTEIN	V.D
RSc1934	RS03503					-1,29	0,02	-1,91	0,03				BACTERIOPHAGE PROTEIN	V.D
RSc1935	RS03504			-1,17	0,00	1,21	0,00						BACTERIOPHAGE PROTEIN	V.D
RSc1936	RS03505					-1,55	0,00	-1,19	0,05				BACTERIOPHAGE PROTEIN	V.D
RSc1937	RS03506					-1,94	0,00	-1,92	0,00				BACTERIOPHAGE PROTEIN	V.D
RSc1938	RS03507					-2,57	0,00	-0,90	0,04				BACTERIOPHAGE PROTEIN	V.D
RSc1939	RS03508												TERMINASE (ATPase SUBUNIT) RELATED PROTEIN	V.D
RSc1940	RS03509												TERMINASE (ATPase SUBUNIT) RELATED PROTEIN	V.D
RSc1941	RS03510												BACTERIOPHAGE PROTEIN	V.D
RSc1942	RS03511					-2,08	0,00						CONSERVED HYPOTHETICAL PROTEIN	VI.C
RSc1943	RS03513					-2,26	0,00			1,33	0,03		TRANSMEMBRANE	VI.C
RSc1944	RS03512					-1,51	0,00						VGR-RELATED PROTEIN	VI.C
RSc1945	RS03514												HYPOTHETICAL PROTEIN	VI.D
RSc1946	RS03515												HYPOTHETICAL PROTEIN	VI.D
RSc1947	RS03518									1,23	0,00		HYPOTHETICAL PROTEIN	VI.D
RSc1948	RS03519					-0,62	0,00	-0,51	0,01	0,59	0,03		HYPOTHETICAL PROTEIN	VI.D
RSc1949	RS03521												TRANSMEMBRANE GLYCINE-RICH PROTEIN	VI.D
RSc1950	RS03524			-2,82	0,02								TRANSMEMBRANE METHYL-ACCEPTING CHEMOTAXIS TRANSDUCER	IV.C
RSc1951	RS03525			0,57	0,05								TRANSMEMBRANE SOLUTE:NA+ SYMPORTER PERMEASE	IV.D.4
RSc1952	RS03526			0,36	0,05	-0,25	0,05						ACETYL-COENZYME A SYNTHETASE	I.C
RSc1953	RS03527					-0,50	0,00						TRANSMEMBRANE	VI.C
RSc1954	RS03528			0,39	0,03	-0,41	0,01						TRANSMEMBRANE COG2862, Predicted membrane protein	VI.C
RSc1955	RS03529											fumA	FUMARATE HYDRATASE	I.H.6
RSc1956	RS03530												GLUTAMATE RACEMASE	III.B.3
RSc1957	RS03531			0,73	0,00								TRANSMEMBRANE GLUTAMATE SYMPORT PROTEIN	IV.D.2
RSc1958	RS03532					-0,63	0,01	-1,59	0,00	1,62	0,00		CONSERVED HYPOTHETICAL PROTEIN	VI.C
RSc1959	RS03533					-0,44	0,02						CONSERVED HYPOTHETICAL PROTEIN	VI.C
RSc1960	RS03534					-0,93	0,00	-0,45	0,03				TRANSCRIPTION REGULATOR TRANSCRIPTIONAL REGULATORY DNA-BINDINC	VI.A
RSc1961	RS03535					-1,28	0,00						COG0464, ATPases of the AAA+ class	VI.C
RSc1962	RS03536					-1,65	0,00						TRANSMEMBRANE	VI.D
RSc1963	RS03537			-0,41	0,02	-1,58	0,00					tonB	TRANSMEMBRANE TONB PROTEIN	III.B.1
RSc1964	RS03538			0,34	0,03	-1,78	0,00	-0,68	0,00	-1,26	0,00	exbB1	TRANSMEMBRANE BIOPOLYMER TRANSPORT PROTEIN	IV.D
RSc1965	RS03539					-1,68	0,00			-1,03	0,00	exbD1	TRANSMEMBRANE BIOPOLYMER TRANSPORT PROTEIN	IV.D
RSc1966	RS03540					-1,79	0,00			-1,03	0,00	hemP	HEMIN UPTAKE PROTEIN	IV.D.5
RSc1967	RS03541			0,35	0,05			1,02	0,00	0,82	0,02	glcG	GLCG PROTEIN	VI.C
RSc1968	RS03542									0,76	0,05		TRANSMEMBRANE	VI.C
RSc1969	RS03543					-0,94	0,00			2,08	0,00		TRANSMEMBRANE	VI.C
RSc1970	RS03544					-0,75	0,00	-0,62	0,02				HYPOTHETICAL PROTEIN	VI.D
RSc1972	RS03546					-0,54	0,00	0,44	0,04	1,24	0,00	phaP3	POLYHYDROXYBUTYRATE GRANULE-ASSOCIATED PROTEIN (PHASIN) PHAP3	II.B
RSc1973	RS03547											osmB1	TRANSMEMBRANE OSMOTICALLY INDUCIBLE LIPOPROTEIN B1	IV.F.2
RSc1974	RS03548					-0,52	0,01						ABC TRANSPORTER ATP-BINDING PROTEIN	IV.D
RSc1975	RS03549					-0,47	0,01						ABC TRANSPORTER TRANSMEMBRANE PERMEASE PROTEIN	IV.D
RSc1976	RS03550			0,62	0,00	-0,31	0,01			-0,58	0,01	purF	AMIDOPHOSPHORIBOSYLTRANSFERASE	I.D.2
RSc1977	RS03551											cvpA	TRANSMEMBRANE COLICIN V PRODUCTION PROTEIN	V.C
RSc1978	RS03552												TRANSMEMBRANE	VI.B
RSc1979	RS03553									-0,38	0,01	folC	BIFUNCTIONAL PROTEIN: FOLYLPOLYGLUTAMATE SYNTHASE AND DIHYDROF	I.B.14
RSc1980	RS03554			0,31	0,02							accD	ACETYL-COENZYME A CARBOXYLASE CARBOXYL TRANSFERASE (SUBUNIT B	I.C
RSc1981	RS03555											trpA	TRYPTOPHAN SYNTHASE (ALPHA CHAIN)	I.A.6
RSc1982	RS03556					0,26	0,00			-0,27	0,00		DNA-METHYLTRANSFERASE (DNA-MODIFICATION METHYLASE)	II.B.8
RSc1983	RS03412											trpB	TRYPTOPHAN SYNTHASE (BETA CHAIN)	I.A.6
RSc1984	RS03413					0,27	0,03	-0,50	0,04			trpF	N-(5'-PHOSPHORIBOSYL)ANTHRANILATE ISOMERASE	I.A.6
RSc1985	RS03414	1,00	0,03			-0,38	0,00					truA	TRNA PSEUDOURIDINE SYNTHASE	II.D.1
RSc1986	RS03415			0,33	0,01	-0,38	0,01						TRANSMEMBRANE TYPE IV PILUS ASSEMBLY FimV-RELATED PROTEIN	III.A.2
RSc1987	RS03416							0,58	0,05			asd	OXIDOREDUCTASE ASPARTATE-SEMIALDEHYDE DEHYDROGENASE	I.A
RSc1988	RS03417			1,03	0,00	1,12	0,00					leuB1	OXIDOREDUCTASE 3-ISOPROPYLMALATE DEHYDROGENASE	I.A.1
RSc1989	RS03557					0,47	0,00			-0,79	0,05	leuD	3-ISOPROPYLMALATE DEHYDRATASE (SMALL SUBUNIT)	I.A.1
RSc1990	RS03558									-0,65	0,03	leuC	3-ISOPROPYLMALATE DEHYDRATASE (LARGE SUBUNIT)	I.A.1
RSc1991	RS03559			1,00	0,00					-1,08	0,00	gltA	CITRATE SYNTHASE	I.H.6
RSc1992	RS03560			1,13	0,02	-0,37	0,00						CONSERVED HYPOTHETICAL PROTEIN	VI.C
RSc1993	RS03561											sdhB	OXIDOREDUCTASE SUCCINATE DEHYDROGENASE (IRON-SULFUR PROTEIN S	I.H.6
RSc1994	RS03562			0,84	0,01							sdhA	OXIDOREDUCTASE SUCCINATE DEHYDROGENASE (FLAVOPROTEIN SUBUNIT)	I.H.6
RSc1995	RS03563			0,69	0,04			-0,48	0,01			sdhD	OXIDOREDUCTASE TRANSMEMBRANE SUCCINATE DEHYDROGENASE (HYDR	I.H.6
RSc1996	RS03564											sdhC	OXIDOREDUCTASE TRANSMEMBRANE SUCCINATE DEHYDROGENASE (CYTO	I.H.6
RSc1997	RS03565					-0,89	0,00						TRANSCRIPTION REGULATOR	VI.A
RSc1998	RS03566			0,83	0,00	-0,53	0,05					mdh	OXIDOREDUCTASE MALATE DEHYDROGENASE	I.H.6
RSc1999	RS03567					-0,80	0,01						COG2301, Citrate lyase beta subunit	I
RSc2000	RS03568			0,93	0,01	-0,48	0,04					prpB1	CARBOXYVINYL-CARBOXYPHOSPHONATE PHOSPHORYLMUTASE	I.I.4
RSc2001	RS03569			0,46	0,04	-0,22	0,02						SIGNAL PEPTIDE	VI.C
RSc2002	RS03570					-0,32	0,03					prpD	PRPD PROTEIN	I.I.4
RSc2003	RS03571			1,13	0,02							acnA1	ACONITATE HYDRATASE	I.H.6
RSc2004	RS03572					-0,80	0,00						SIGNAL PEPTIDE	VI.D
RSc2005	RS03573												CONSERVED HYPOTHETICAL PROTEIN	VI.C
RSc2006	RS03574			0,64	0,02	-0,57	0,00						ABC TRANSPORTER COG0747, ABC-type dipeptide transport system, periplasmic c	IV.D.2
RSc2007	RS03575			0,53	0,04								TRANSMEMBRANE LIPOPROTEIN	VI.C
RSc2008	RS03576			0,64	0,00	-0,39	0,01						COG2866, Predicted carboxypeptidase	I
RSc2009	RS03577					-0,46	0,00						CONSERVED HYPOTHETICAL PROTEIN	VI.C
RSc2010	RS03578			0,48	0,05								CONSERVED HYPOTHETICAL PROTEIN	VI.C
RSc2011	RS03579					-0,65	0,00						COG0436, Aspartate/tyrosine/aromatic aminotransferase	I
RSc2012	RS03580			0,31	0,04							gstH	GLUTATHIONE S-TRANSFERASE	II.B.7
RSc2013	RS03581											paaH2	OXIDOREDUCTASE 3-HYDROXYBUTYRYL-COA DEHYDROGENASE	I.I.4
RSc2014	RS03582					-0,71	0,02					paaG2	ENOYL-COA HYDRATASE	I.I.4
RSc2015	RS03583					-0,48	0,02						COG0406, Fructose-2,6-bisphosphatase	I
RSc2016	RS03584					-0,68	0,00						COG3173, Predicted aminoglycoside phosphotransferase	VI.C
RSc2017	RS03585					-0,57	0,00			0,52	0,01		CONSERVED HYPOTHETICAL PROTEIN	VI.C
RSc2018	RS03586					-0,51	0,01	-0,59	0,02				TRANSCRIPTION REGULATOR	VI.A
RSc2019	RS03587			0,60	0,05	-0,71	0,01	-1,27	0,02				SIGNAL PEPTIDE COG2050, Uncharacterized protein, possibly involved in aromatic c	VI.C
RSc2020	RS03588												OXIDOREDUCTASE ACYL-CoA DEHYDROGENASE	I
RSc2021	RS03589					-0,65	0,00			0,80	0,01	gstI	GLUTATHIONE-S-TRANSFERASE	II.B.7
RSc2022	RS03590							-0,98	0,03				TRANSMEMBRANE COG2020, Putative protein-S-isoprenylcysteine methyltransferas	II.B.7
RSc2023	RS03591			0,45	0,05	-0,68	0,00						HYPOTHETICAL PROTEIN	VI.D
RSc2024	RS03592	-2,10	0,01	1,08	0,00								TRANSMEMBRANE COG1279, LysE-type efflux permease	IV.D
RSc2025	RS03593	-0,78	0,00	1,05	0,00	-0,50	0,00			1,06	0,01		TRANSMEMBRANE COG3239, Fatty acid desaturase	I
RSc2026	RS03598			0,32	0,01	-1,08	0,00						HYPOTHETICAL PROTEIN	VI.D
RSc2027	RS03594	-1,18	0,05			-0,70	0,00	-0,61	0,03				HYPOTHETICAL PROTEIN	VI.D
RSc2028	RS03599												HYPOTHETICAL PROTEIN	VI.D
RSc2029	RS03595			1,26	0,02							ureG	UREASE ACCESSORY PROTEIN	I.F.5
RSc2030	RS03600			1,11	0,00							ureF	UREASE ACCESSORY PROTEIN UREF	I.F.5
RSc2031	RS03601					-0,52	0,04	-0,99	0,02			ureE	UREASE ACCESSORY PROTEIN UREE	I.F.5
RSc2032	RS02789			0,48	0,01	-0,34	0,00					ureC	UREASE (ALPHA SUBUNIT)	I.F.5
RSc2033	RS02962			0,67	0,00	-0,37	0,02					ureB	UREASE (BETA SUBUNIT)	I.F.5
RSc2034	RS03028			0,64	0,02							ureJ	TRANSMEMBRANE UREASE ACCESSORY PROTEIN UREJ	I.F.5
RSc2035	RS03029					-0,38	0,03					ureA	UREASE (GAMMA SUBUNIT)	I.F.5
RSc2036	RS03030			0,89	0,01	-0,76	0,00	-1,73	0,04			ureD	UREASE ACCESSORY PROTEIN URED	I.F.5
RSc2037	RS03602					-0,47	0,04						ABC TRANSPORTER ATP-BINDING PROTEIN	IV.D
RSc2038	RS03603			-1,76	0,02								ABC TRANSPORTER ATP-BINDING PROTEIN	IV.D
RSc2039	RS03604					-0,94	0,02						TRANSMEMBRANE PERMEASE TRANSPORTER PROTEIN	IV.D
RSc2040	RS03605					-0,67	0,04						TRANSMEMBRANE PERMEASE TRANSPORTER PROTEIN	IV.D
RSc2041	RS03606					-0,65	0,02						ABC TRANSPORTER COG0683, ABC-type branched-chain amino acid transport syst	IV.D
RSc2042	RS03607			0,33	0,05	-0,58	0,03						COG2030, Acyl dehydratase	I
RSc2043	RS03608					-0,49	0,03						COG2030, Acyl dehydratase	I
RSc2044	RS03609			0,70	0,00	-0,64	0,01						OXIDOREDUCTASE ACYL-COA DEHYDROGENASE	I.I.1
RSc2045	RS03610	0,87	0,03			-0,73	0,00						OXIDOREDUCTASE ACYL-COA DEHYDROGENASE	I

RSc2046	RS03611					-0,93	0,00	-0,74	0,01	-1,62	0,00		CONSERVED HYPOTHETICAL PROTEIN	VI.C
RSc2047	RS03612			0,84	0,01								COG0494, NTP pyrophosphohydrolases	I
RSc2048	RS03613			1,31	0,05								TRANSMEMBRANE	VI.C
RSc2049	RS03614	0,76	0,00	1,01	0,00	0,45	0,04					nuoN	OXIDOREDUCTASE TRANSMEMBRANE NADH DEHYDROGENASE I (CHAIN N)	I.H.3
RSc2050	RS03615			1,29	0,00	0,42	0,05			-2,98	0,04	nuoM	OXIDOREDUCTASE TRANSMEMBRANE NADH DEHYDROGENASE I (CHAIN M)	I.H.3
RSc2051	RS03616			0,95	0,02							nuoL	OXIDOREDUCTASE TRANSMEMBRANE NADH DEHYDROGENASE I (CHAIN L)	I.H.3
RSc2052	RS03617	0,40	0,05	0,95	0,01							nuoK	OXIDOREDUCTASE TRANSMEMBRANE NADH DEHYDROGENASE I (CHAIN K)	I.H.3
RSc2053	RS03618			0,76	0,00							nuoJ	OXIDOREDUCTASE TRANSMEMBRANE NADH DEHYDROGENASE I (CHAIN J)	I.H.3
RSc2054	RS03619			0,81	0,02			-0,51	0,05			nuoI	OXIDOREDUCTASE NADH DEHYDROGENASE I (CHAIN I)	I.H.3
RSc2055	RS03620											nuoH	OXIDOREDUCTASE TRANSMEMBRANE NADH DEHYDROGENASE I (CHAIN H)	I.H.3
RSc2056	RS03621			0,63	0,02							nuoG	OXIDOREDUCTASE NADH DEHYDROGENASE I (CHAIN G)	I.H.3
RSc2057	RS03622			0,92	0,00	0,18	0,00					nuoF	OXIDOREDUCTASE NADH DEHYDROGENASE I (CHAIN F)	I.H.3
RSc2058	RS03623			0,53	0,02			0,78	0,01			nuoE	OXIDOREDUCTASE NADH DEHYDROGENASE I (CHAIN E)	I.H.3
RSc2059	RS03624			0,99	0,01	0,14	0,02					nuoD	OXIDOREDUCTASE NADH DEHYDROGENASE I (CHAIN D)	I.H.3
RSc2060	RS03625			0,73	0,00							nuoC	OXIDOREDUCTASE NADH DEHYDROGENASE I (CHAIN C)	I.H.3
RSc2061	RS03626			-0,55	0,00	0,85	0,01					nuoB	OXIDOREDUCTASE NADH DEHYDROGENASE I (CHAIN B)	I.H.3
RSc2062	RS03627			0,54	0,03							nuoA	OXIDOREDUCTASE TRANSMEMBRANE NADH DEHYDROGENASE I (CHAIN A)	I.H.3
RSc2063	RS03628			0,61	0,01	-0,43	0,02			-0,67	0,00	secG	TRANSMEMBRANE PROTEIN-EXPORT MEMBRANE PROTEIN SECG (TRANSLOC	IV.E.2
RSc2064	RS03629			0,67	0,01	-0,39	0,01			-0,52	0,01	tpiA	TRIOSEPHOSPHATE ISOMERASE	I.H.8
RSc2065	RS03630			0,51	0,00	-0,28	0,03						OXIDOREDUCTASE NADPH QUINONE OXIDOREDUCTASE	I.G.1
RSc2066	RS03631			0,92	0,00			2,13	0,00				CONSERVED HYPOTHETICAL PROTEIN	VI.C
RSc2067	RS03632			0,72	0,00					-0,54	0,00	pnp	POLYRIBONUCLEOTIDE NUCLEOTIDYLTRANSFERASE	II.B.6
RSc2068	RS03633									-2,48	0,00		SIGNAL PEPTIDE	VI.D
RSc2069	RS03634									-0,84	0,00	rpsO	30S RIBOSOMAL SUBUNIT PROTEIN S15	III.C.2
RSc2070	RS03635			1,50	0,03	-0,49	0,00						SIGNAL PEPTIDE TRANSPORTER PROTEIN	IV.D
RSc2071	RS03636					-0,70	0,00						TRANSMEMBRANE	VI.C
RSc2072	RS03637			0,69	0,03	-0,18	0,04	0,50	0,04	-0,43	0,02	leuA1	2-ISOPROPYLMALATE SYNTHASE	I.A.1
RSc2073	RS03638			0,63	0,00	-0,34	0,03	0,72	0,05			pssA	CDP-DIACYLGLYCEROL--SERINE O-PHOSPHATIDYLTRANSFERASE	II.B.1
RSc2074	RS03639			0,45	0,02	-0,39	0,01	1,02	0,02				TRANSMEMBRANE COG0688, Phosphatidylserine decarboxylase	I
RSc2075	RS03640									-0,95	0,00	ilvC	OXIDOREDUCTASE KETOL-ACID REDUCTOISOMERASE	I.A.2
RSc2076	RS03641									-0,65	0,00	ilvH	ACETOLACTATE SYNTHASE ISOZYME III (SMALL SUBUNIT)	I.A.2
RSc2077	RS03642			0,66	0,03	-0,18	0,05	0,70	0,01	-1,25	0,00	ilvI	ACETOLACTATE SYNTHASE ISOZYME III (LARGE SUBUNIT)	I.A.2
RSc2078	RS03643					-0,39	0,01						TRANSCRIPTION REGULATOR RNA POLYMERASE SIGMA FACTOR	I.E.1
RSc2079	RS03644					-0,40	0,00			-0,31	0,01		TRANSMEMBRANE	VI.C
RSc2080	RS03645					-0,56	0,00	1,26	0,01	-1,77	0,01		TRANSMEMBRANE	VI.B
RSc2081	RS03646												TRANSMEMBRANE	VI.C
RSc2082	RS03647					-0,26	0,04						TRANSMEMBRANE LIPASE	I.I.1
RSc2083	RS03648												CONSERVED HYPOTHETICAL PROTEIN	VI.C
RSc2084	RS03649												COG0438, Glycosyltransferase	I
RSc2085	RS03650					-0,41	0,00					dgkA	TRANSMEMBRANE DIACYLGLYCEROL KINASE	I.C
RSc2086	RS03651							0,54	0,05	0,72	0,03		TRANSCRIPTION REGULATOR	VI.A
RSc2087	RS03652					-0,74	0,00						CONSERVED HYPOTHETICAL PROTEIN	VI.C
RSc2088	RS03653					-0,20	0,01						TRANSMEMBRANE COG1280, Threonine efflux protein	IV.D.2
RSc2089	RS03654					-0,16	0,03						TRANSMEMBRANE	VI.C
RSc2090	RS03655												OXYGEN-BINDING PROTEIN (GLOBIN)	IV.D
RSc2091	RS03656			-1,21	0,01	1,69	0,00	1,44	0,00	-0,29	0,04		ABC TRANSPORTER COG3127, Predicted ABC-type transport system involved in lys	IV.E
RSc2092	RS03657			-0,95	0,00	1,55	0,00						TRANSCRIPTION REGULATOR	VI.A
RSc2093	RS03658					-0,57	0,02	-1,28	0,00				ABC TRANSPORTER COG2998, ABC-type tungstate transport system, permease cor	IV.D
RSc2094	RS03659												TRANSCRIPTION REGULATOR	VI.A
RSc2095	RS03660											xdhA	OXIDOREDUCTASE XANTHINE DEHYDROGENASE (SUBUNIT A)	I.I.4
RSc2096	RS03661							1,87	0,04			xdhB	OXIDOREDUCTASE XANTHINE DEHYDROGENASE (SUBUNIT B)	I.I.4
RSc2097	RS03662											xdhC	XANTHINE DEHYDROGENASE	I.I.4
RSc2098	RS03663											add	ADENOSINE DEAMINASE	I.F.1
RSc2099	RS03664					0,30	0,00					guaD	GUANINE DEAMINASE (GUANINE AMINOHYDROLASE) (GAH)	I.F.18
RSc2100	RS01504					-0,34	0,01						TRANSMEMBRANE	VI.C
RSc2101	RS01503			-1,57	0,00	4,00	0,00	4,44	0,00				HYPOTHETICAL PROTEIN	VI.D
RSc2102	RS01502												TRANSCRIPTION REGULATOR	VI.A
RSc2103	RS01501												ABC TRANSPORTER AMINO-ACID-BINDING PERIPLASMIC PROTEIN (PBP)	IV.D.2
RSc2104	RS01500			1,28	0,05	-0,42	0,01	0,48	0,02				ABC TRANSPORTER AMINO-ACID TRANSMEMBRANE PROTEIN	IV.D.2
RSc2105	RS01499	-0,34	0,05										ABC TRANSPORTER AMINO-ACID TRANSMEMBRANE PROTEIN	IV.D.2
RSc2106	RS01498												ABC TRANSPORTER AMINO-ACID ATP-BINDING PROTEIN	IV.D.2
RSc2107	RS01497									3,02	0,00	rsl	FUCOSE-BINDING LECTIN	I.F.7
RSc2108	RS01496					-1,18	0,01	-1,40	0,00				SIGNAL PEPTIDE PORIN	IV.D
RSc2109	RS01495			-2,30	0,00	1,98	0,00						TRANSMEMBRANE	VI.C
RSc2110	RS01494					-0,38	0,01					aldH	OXIDOREDUCTASE ALDEHYDE DEHYDROGENASE	I.H
RSc2111	RS01493			0,55	0,02	-0,43	0,00			-0,68	0,00		DIHYDRODIPICOLINATE SYNTHASE (DHDPS)	I.A.13
RSc2112	RS01492												TRANSMEMBRANE METABOLITE TRANSPORT PROTEIN	IV.D
RSc2113	RS01491			-2,01	0,00								OXIDOREDUCTASE COG0665, Glycine/D-amino acid oxidases (deaminating)	I
RSc2114	RS01490					-0,65	0,00	-1,12	0,01				TRANSCRIPTION REGULATOR	VI.A
RSc2115	RS01489					-0,41	0,00						TRANSMEMBRANE	VI.C
RSc2116	RS01488					-0,74	0,01						TRANSTHYRETIN-LIKE PROTEIN	VI.C
RSc2117	RS01487							-0,55	0,02				TRANSCRIPTION REGULATOR	VI.A
RSc2118	RS01486												TRANSMEMBRANE XANTHINE PERMEASE	IV.D.6
RSc2119	RS01485							-1,18	0,05				CHLOROHYDROLASE	IV.G.4
RSc2120	RS01484												COG0726, Predicted xylanase/chitin deacetylase	II.A.3
RSc2121	RS01483												CONSERVED HYPOTHETICAL PROTEIN	VI.C
RSc2122	RS01482					-0,29	0,02	-0,53	0,03				HYPOTHETICAL PROTEIN	VI.B
RSc2123	RS01481					-0,50	0,02			-0,95	0,02	maeB1	OXIDOREDUCTASE NADP-DEPENDENT MALIC ENZYME	I.F.18
RSc2124	RS01480					-1,08	0,00	-0,95	0,01	-0,49	0,04		TRANSMEMBRANE LIPOPROTEIN	VI.C
RSc2125	RS01479			0,29	0,05	-0,31	0,00			-0,17	0,01	pepN	AMINOPEPTIDASE N (ALPHA-AMINOACYLPEPTIDE HYDROLASE) METALLOPRC	II.A.4
RSc2126	RS01478					-0,30	0,01					fbp	FRUCTOSE-1,6-BISPHOSPHATASE	I.F.13
RSc2127	RS01477					-0,33	0,05					dalR	TRANSCRIPTION REGULATOR TRANSCRIPTIONAL REGULATOR (REPRESSOR)	I.F
RSc2128	RS01476			0,86	0,01							dalK	D-ARABINITOL KINASE	I.F
RSc2129	RS01475											dalD	OXIDOREDUCTASE D-ARABINITOL DEHYDROGENASE	I.F
RSc2130	RS01474							2,09	0,00	3,08	0,00		SKWP PROTEIN 6	VI.C
RSc2132	RS01472					-0,51	0,04	-1,32	0,00				HYPOTHETICAL PROTEIN	VI.B
RSc2133	RS01471			-2,72	0,00								TRANSMEMBRANE	VI.B
RSc2134	RS01470					1,09	0,02						HYPOTHETICAL PROTEIN	VI.D
RSc2135	RS01469					1,39	0,00						HYPOTHETICAL PROTEIN	VI.D
RSc2136	RS01467												SIGNAL PEPTIDE PORIN	IV.D
RSc2137	RS01466												HYPOTHETICAL PROTEIN	VI.B
RSc2138	RS01465							1,85	0,00	3,26	0,00		TRANSCRIPTION REGULATOR COG1733	VI.A
RSc2139	RS01464												AWR TYPE III EFFECTOR FAMILY PROTEIN	VI.C
RSc2140	RS01463												ABC TRANSPORTER SUGAR ATP-BINDING PROTEIN	IV.D.3
RSc2141	RS01462			0,43	0,04								TRANSMEMBRANE COG0637, Phosphatase/phosphohexomutase	I
RSc2142	RS01461												ABC TRANSPORTER TRANSMEMBRANE PROTEIN	IV.D.3
RSc2143	RS01460			1,17	0,03								ABC TRANSPORTER TRANSMEMBRANE PROTEIN	IV.D.3
RSc2144	RS01459												SIGNAL PEPTIDE SUGAR-BINDING PERIPLASMIC PROTEIN	IV.D.3
RSc2145	RS01458					-0,59	0,01	-0,65	0,01			agaZ	TAGATOSE 6-PHOSPHATE KINASE	II.A.3
RSc2146	RS01457											cscK	FRUCTOKINASE	I.F.7
RSc2147	RS01456			-3,04	0,00	2,95	0,00					polS	OXIDOREDUCTASE SORBITOL DEHYDROGENASE (L-IDITOL 2-DEHYDROGENAS	I.F
RSc2148	RS01455					-1,49	0,00			1,12	0,01		CONSERVED HYPOTHETICAL PROTEIN	VI.C
RSc2149	RS01454					-0,43	0,00						COG3509, Poly(3-hydroxybutyrate) depolymerase	I.I.4
RSc2150	RS01453							0,56	0,01				HYPOTHETICAL PROTEIN	VI.D
RSc2151	RS01452					-0,55	0,02			1,58	0,00		SIGNAL PEPTIDE	VI.C
RSc2152	RS01451					-0,44	0,02					greB	TRANSCRIPTION ELONGATION FACTOR (TRANSCRIPT CLEAVAGE FACTOR)	II.B.6
RSc2153	RS01611											spoT	BIFUNCTIONAL ENZYME : (p)ppGpp SYNTHETASE II AND GUANOSINE-3',5'-BISD	I.E.1
RSc2154	RS01448							-0,41	0,04			rpoZ	DNA-DIRECTED RNA POLYMERASE (OMEGA CHAIN)	II.B.6
RSc2155	RS01447											gmk	GUANYLATE KINASE (GMP KINASE)	I.D.2
RSc2156	RS01446			0,56	0,01								CONSERVED HYPOTHETICAL PROTEIN	VI.C
RSc2157	RS01445			0,41	0,01	-0,48	0,00						COG0515, Serine/threonine protein kinase	VI.C
RSc2158	RS01444							0,45	0,02				COG0631, Serine/threonine protein phosphatase	VI.C

RSc2159	RS01443			-0,64 0,01	-0,81 0,01		rph	RIBONUCLEASE PH (TRNA NUCLEOTIDYLTRANSFERASE)	II.A.2
RSc2160	RS01442				-0,65 0,03			COG0127, Xanthosine triphosphate pyrophosphatase	I
RSc2161	RS01441		0,89 0,01	-0,51 0,01				OXIDOREDUCTASE COG0635, Coproporphyrinogen III oxidase and related Fe-S oxi	I
RSc2162	RS01439							COG1234, Metal-dependent hydrolases of the beta-lactamase superfamily III	I.I
RSc2163	RS01438			-0,34 0,01		1,36 0,00		CONSERVED HYPOTHETICAL PROTEIN	VI.C
RSc2164	RS01436			-0,23 0,03	0,56 0,02			SIGNAL PEPTIDE	VI.B
RSc2165	RS01434						pcp1	PYRROLIDONE-CARBOXYLATE PEPTIDASE (5-OXOPROLYL-PEPTIDASE)	I.F.14
RSc2166	RS01617			-0,63 0,01	-1,17 0,01			SIGNAL PEPTIDE COG1638, TRAP-type C4-dicarboxylate transport system, periplas	IV.D.3
RSc2167	RS01432	-0,57 0,05	1,02 0,03		0,83 0,00			TRANSMEMBRANE	VI.C
RSc2168	RS01431		0,38 0,01		0,45 0,03			TRANSMEMBRANE	VI.C
RSc2169	RS01430			0,46 0,04				CONSERVED HYPOTHETICAL PROTEIN	VI.C
RSc2170	RS01429		0,68 0,01					COG1984, Allophanate hydrolase subunit 2	I.I
RSc2171	RS01428							COG2049, Allophanate hydrolase subunit 1	I.I
RSc2172	RS01427							TRANSMEMBRANE DRUG TRANSPORT PROTEIN	IV.D
RSc2173	RS01426			-0,40 0,05				ABC TRANSPORTER SUGAR ATP BINDING PROTEIN	IV.D.3
RSc2174	RS01425							ABC TRANSPORTER TRANSMEMBRANE PROTEIN	IV.D
RSc2175	RS01424		0,85 0,02					ABC TRANSPORTER TRANSMEMBRANE PROTEIN	IV.D
RSc2177	RS01422							LIPOPROTEIN	VI.C
RSc2178	RS01421							HYPOTHETICAL PROTEIN	VI.D
RSc2179	RS01420				0,53 0,01			TRANSMEMBRANE	VI.B
RSc2180	RS01419			0,90 0,00		-1,86 0,00		TRANSMEMBRANE	VI.D
RSc2181	RS01418							HYPOTHETICAL PROTEIN	VI.B
RSc2182	RS01417							HYPOTHETICAL PROTEIN	VI.D
RSc2183	RS01416				-0,51 0,02			CONSERVED HYPOTHETICAL PROTEIN	VI.C
RSc2184	RS01415		0,71 0,00	-0,28 0,05			qor	OXIDOREDUCTASE NADPH:QUINONE REDUCTASE, ZETA-CRYSTALLIN HOMOL	I.G.1
RSc2185	RS01414	-0,48 0,00	0,47 0,01		0,48 0,01		mgsA	METHYLGLYOXAL SYNTHASE	I.F.18
RSc2186	RS01413		0,42 0,05	-0,42 0,00				OXIDOREDUCTASE COG1028, Dehydrogenases with different specificities (related t	I
RSc2187	RS01412		0,89 0,00					TRANSMEMBRANE	VI.C
RSc2188	RS01411				0,45 0,01	0,92 0,03	upp	URACIL PHOSPHORIBOSYLTRANSFERASE	I.F.1
RSc2189	RS01410		0,56 0,01		0,75 0,01			COG0433, PREDICTED ATPase	VI.C
RSc2190	RS01409		-1,34 0,01	1,79 0,00				CONSERVED HYPOTHETICAL PROTEIN	VI.C
RSc2191	RS01408		0,74 0,00			-0,30 0,01	purD	PHOSPHORIBOSYLAMINE--GLYCINE LIGASE	I.D.2
RSc2192	RS01407	0,51 0,04		-0,36 0,04			hemF	OXIDOREDUCTASE COPROPORPHYRINOGEN III OXIDASE, AEROBIC	I.B.4
RSc2193	RS01406			-0,38 0,00				COG1057, Nicotinic acid mononucleotide adenylyltransferase	I
RSc2194	RS01405			-0,26 0,03				CONSERVED HYPOTHETICAL PROTEIN	VI.C
RSc2195	RS01404							CONSERVED HYPOTHETICAL PROTEIN	VI.C
RSc2196	RS01403			-0,47 0,00				COG0424, Nucleotide-binding protein implicated in inhibition of septum formation	IV.A
RSc2197	RS01402			-0,24 0,02			cafA	RIBONUCLEASE G (RNASE G) (CYTOPLASMIC AXIAL FILAMENT PROTEIN)	IV.A
RSc2198	RS01401							CONSERVED HYPOTHETICAL PROTEIN	VI.C
RSc2199	RS01400			-0,37 0,02	0,78 0,02			COG2128, Uncharacterized conserved protein	VI.C
RSc2200	RS01399		-1,95 0,00	2,19 0,00				ABC TRANSPORTER PROTEIN ABC-TYPE MULTIDRUG TRANSPORT SYSTEM , A	IV.D
RSc2201	RS01398			-0,27 0,03				SIGNAL PEPTIDE COG1216, Glycosyltransferase	I
RSc2202	RS01397				-0,55 0,04			GLYCOSYL TRANSFERASE	II.B
RSc2203	RS01396							COG1216, Glycosyltransferase	II.B.2
RSc2204	RS01395		0,80 0,05	-0,38 0,01			waaL	TRANSMEMBRANE LIPOPOLYSACCHARIDE O-ANTIGEN LIGASE	II.B.2
RSc2205	RS01394			-0,41 0,00		-0,43 0,01	dnaE1	DNA POLYMERASE III (ALPHA CHAIN)	II.B.8
RSc2206	RS01393				2,13 0,02	-0,18 0,01		COG1054, Predicted sulfurtransferase	I.F.3
RSc2207	RS01392			-0,91 0,00				COG0655, Multimeric flavodoxin WrbA	VI.C
RSc2208	RS01391	-1,58 0,01		-2,18 0,00	0,44 0,04			PIRIN-RELATED PROTEIN	VI.C
RSc2209	RS01390	-1,73 0,00		-0,57 0,00				TRANSCRIPTION REGULATOR TRANSCRIPTIONAL REGULATOR	VI.A
RSc2210	RS01389			-0,52 0,00				COG0008, Glutamyl- and glutaminyl-tRNA synthetases	II.D.1
RSc2211	RS01388			-0,50 0,01			rhlE2	ATP-DEPENDENT RNA HELICASE	II.B.7
RSc2212	RS01387							CONSERVED HYPOTHETICAL PROTEIN	VI.C
RSc2213	RS01386		0,56 0,01			-1,13 0,05		TRANSCRIPTION REGULATOR TRANSCRIPTION REGULATOR	VI.A
RSc2214	RS01385							COG0491, Zn-dependent hydrolase	I.I
RSc2215	RS01384				0,43 0,05		rpoD	TRANSCRIPTION REGULATOR RNA POLYMERASE SIGMA D (SIGMA-70) FACTO	I.E.1
RSc2216	RS01383		0,34 0,02	-0,33 0,03			dnaG	DNA PRIMASE	II.B.8
RSc2217	RS01382		0,41 0,05			-1,12 0,05		CONSERVED HYPOTHETICAL PROTEIN	VI.C
RSc2218	RS01381			-0,46 0,05				TRANSMEMBRANE COG2081, Flavoprotein	VI.C
RSc2219	RS01380						gcp	O-SIALOGLYCOPROTEIN ENDOPEPTIDASE	II.A.4
RSc2220	RS01379			-0,76 0,00				CONSERVED HYPOTHETICAL PROTEIN	VI.C
RSc2221	RS01378			-0,40 0,01			dxs	1-DEOXYXYLULOSE-5-PHOSPHATE SYNTHASE	I.F.18
RSc2222	RS01377		0,46 0,02	-0,47 0,02			ispA	GERANYLTRANSTRANSFERASE (FARNESYL-DIPHOSPHATE SYNTHASE)	I.B.5
RSc2223	RS01376			-0,51 0,00		0,43 0,02	xseB	EXODEOXYRIBONUCLEASE SMALL SUBUNIT	II.A.1
RSc2224	RS01375		0,62 0,01			-0,81 0,00		OXIDOREDUCTASE COG4638, Phenylpropionate dioxygenase and related ring-hydr	I
RSc2225	RS01374			-0,32 0,02				TRANSMEMBRANE	VI.C
RSc2226	RS01373						sseA	THIOSULFATE SULFURTRANSFERASE	I.F.3
RSc2227	RS01372				-0,94 0,02	-1,49 0,00		TRANSMEMBRANE COG0428, Predicted divalent heavy-metal cations transporter	IV.D.4
RSc2228	RS01371			-0,60 0,02				COG0412, Dienelactone hydrolase and related enzymes	I
RSc2229	RS01370				-0,27 0,04		ndh	OXIDOREDUCTASE NADH DEHYDROGENASE, MEMBRANE FLAVOPROTEIN FA	I.H.3
RSc2230	RS01369			-0,27 0,04			polA	DNA POLYMERASE I	II.B.8
RSc2231	RS01368							COG1611, Rossmann fold nucleotide-binding protein	VI.C
RSc2232	RS01367			-0,38 0,02				TRANSMEMBRANE	VI.B
RSc2233	RS01366		0,72 0,02				thrB	HOMOSERINE KINASE	I.A.12
RSc2234	RS01365			-0,39 0,04	-0,41 0,05	-1,26 0,00		TRANSMEMBRANE	VI.C
RSc2235	RS01364		0,44 0,00				uvrD	DNA HELICASE II	II.B.8
RSc2236	RS01363						valS	VALYL-TRNA SYNTHETASE (VALINE--TRNA LIGASE)	II.D.1
RSc2237	RS01362		0,65 0,00				galU1	UTP--GLUCOSE-1-PHOSPHATE URIDYLYLTRANSFERASE	I.I.4
RSc2238	RS01361					-4,12 0,00		TRANSMEMBRANE	VI.C
RSc2239	RS01360			-0,70 0,00		-1,06 0,02	infA2	TRANSLATION INITIATION FACTOR IF-1	II.B.7
RSc2240	RS01359			-1,17 0,00		0,67 0,00		CONSERVED HYPOTHETICAL PROTEIN	VI.C
RSc2241	RS01318		0,89 0,03	-0,46 0,03				COG2342, Extracellular endo alpha-1,4 polygalactosaminidase or related polysacchar	II.A.3
RSc2242	RS01317	-0,77 0,04		-1,61 0,00		-0,36 0,03	pobB	OXIDOREDUCTASE 4-HYDROXYBENZOATE 3-MONOOXYGENASE	I.I.4
RSc2243	RS01316			-0,68 0,03				TRANSCRIPTION REGULATOR	VI.A
RSc2244	RS01315		-1,06 0,00	1,71 0,00	1,00 0,03			ABC TRANSPORTER SUBSTRATE-BINDING PERIPLASMIC PROTEIN	IV.D
RSc2245	RS01314							ABC TRANSPORTER ATP-BINDING PROTEIN	IV.D
RSc2246	RS01313							ABC TRANSPORTER ATP-BINDING PROTEIN	IV.D
RSc2247	RS01312			-0,57 0,03				ABC TRANSPORTER TRANSMEMBRANE PROTEIN	IV.D
RSc2248	RS01311							ABC TRANSPORTER TRANSMEMBRANE PROTEIN	IV.D
RSc2249	RS01310			-0,94 0,00	-0,99 0,01	-0,44 0,02	pcaC	4-CARBOXYMUCONOLACTONE DECARBOXYLASE	I.I.4
RSc2250	RS01309			-0,49 0,00			pcaD	TRANSMEMBRANE B-KETOADIPATE ENOL-LACTONE HYDROLASE	I.I.4
RSc2251	RS01308		0,46 0,01	-0,50 0,02	-0,88 0,03		pcaB	3-CARBOXY-CIS,CIS-MUCONATE CYCLOISOMERASE	I.I.4
RSc2252	RS01307						pacF	BETA-KETOADIPYL COA THIOLASE	I.I.4
RSc2253	RS01306			-0,70 0,01	-1,13 0,00	-0,97 0,00	pcaJ	3-OXOADIPATE COA-TRANSFERASE SUBUNIT B	I.I.4
RSc2254	RS01305			-0,53 0,00	-0,96 0,02		pcaI	3-OXOADIPATE COA-TRANSFERASE SUBUNIT A	I.I.4
RSc2255	RS01304			-0,58 0,01	-0,69 0,04	-1,31 0,02		TRANSCRIPTION REGULATOR	VI.A
RSc2256	RS01303			-1,00 0,00				SIGNAL PEPTIDE	VI.C
RSc2257	RS01302	-0,36 0,05		-0,90 0,00	-1,01 0,00	-1,03 0,00		ABC TRANSPORTER AMINO-ACID-BINDING PERIPLASMIC PROTEIN	IV.D.2
RSc2258	RS01301							ABC TRANSPORTER AMINO ACID TRANSMEMBRANE PROTEIN	IV.D.2
RSc2259	RS01300		0,75 0,00		-1,06 0,02			ABC TRANSPORTER AMINO ACID TRANSMEMBRANE PROTEIN	IV.D.2
RSc2260	RS01299			-0,60 0,00				ABC TRANSPORTER AMINO-ACID ATP-BINDING PROTEIN	IV.D.2
RSc2261	RS01298			-0,48 0,00				ABC TRANSPORTER AMINO-ACID-BINDING PERIPLASMIC PROTEIN (PBP)	IV.D.2
RSc2262	RS01297			-0,71 0,02				OXIDOREDUCTASE TRANSMEMBRANE D-AMINO ACID DEHYDROGENASE (SMA	I.I.3
RSc2263	RS01296			-0,67 0,04				LIPOPROTEIN	VI.C
RSc2264	RS01295				-1,57 0,02			LIPOPROTEIN	VI.D
RSc2267	RS01292			-0,42 0,02	-0,68 0,02	-0,91 0,01	ISRso8	ISRso8-TRANSPOSASE ORFB	V.A
RSc2268	RS01291						TISRso_	ISRso8-TRANSPOSASE ORFA	V.A
RSc2269	RS01290			-0,33 0,02		1,19 0,02		SIGNAL PEPTIDE	VI.D
RSc2270	RS01289		0,43 0,04	-0,41 0,02			ragG	TRANSMEMBRANE POLYSACCHARIDE SYNTHASE	III.A.1
RSc2271	RS01288		0,59 0,01	-0,61 0,00			ragF	GLYCOSYL-TRANSFRASE	III.A.1
RSc2272	RS01287		0,32 0,01	-0,50 0,00	-0,65 0,01		ragE	TRANSMEMBRANE POLYSACCHARIDE TRANSPORT SYSTEM COMPONENT	III.A.1
RSc2273	RS01286				-0,88 0,01		ragD	TRANSMEMBRANE POLYSACCHARIDE TRANSPORT SYSTEM COMPONENT	III.A.1

RSc2274	RS01284			-0,59 0,04	-0,59 0,02		ragC	OUTER MEMBRANE LIPOPROTEIN	III.A.1
RSc2275	RS01283		1,07 0,01	-0,52 0,02			ragB	SIGNAL PEPTIDE POLYSACCHARIDE TRANSPORT SYSTEM COMPONENT	III.A.1
RSc2276	RS01282			-0,86 0,01			ragA	SIGNAL PEPTIDE POLYSACCHARIDE TRANSPORT SYSTEM COMPONENT	III.A.1
RSc2277	RS01281	1,11 0,00		-0,62 0,01				TRANSMEMBRANE	VI.C
RSc2278	RS01280			-0,81 0,01				HYPOTHETICAL PROTEIN	VI.D
RSc2279	RS01279			-0,69 0,00		0,98 0,04		SIGNAL PEPTIDE	VI.D
RSc2280	RS01277			-0,98 0,01	1,11 0,01	1,76 0,00		SIGNAL PEPTIDE	VI.C
RSc2281	RS01276		0,81 0,00			2,00 0,03		TRANSMEMBRANE	VI.D
RSc2282	RS01275	-1,64 0,04		-0,25 0,04	2,29 0,02	2,16 0,00		HYPOTHETICAL PROTEIN	VI.D
RSc2283	RS01274			-0,30 0,05	0,54 0,01	1,98 0,00		HYPOTHETICAL PROTEIN	VI.D
RSc2284	RS01272	0,49 0,05			0,90 0,04			TRANSMEMBRANE COG3713, Outer membrane protein V - related protein	III.B.2
RSc2285	RS01271	-3,43 0,00		-0,91 0,00	3,34 0,00	3,22 0,00		SIGNAL PEPTIDE	VI.C
RSc2286	RS01270	-3,06 0,00		-0,65 0,01	2,22 0,00	3,58 0,00		CONSERVED HYPOTHETICAL PROTEIN	VI.C
RSc2287	RS01269							COG0526, Thiol-disulfide isomerase and thioredoxins	II.B.7
RSc2288	RS01268			-0,72 0,00		1,42 0,01		CONSERVED HYPOTHETICAL PROTEIN	VI.C
RSc2289	RS01267							CYTOCHROME C1 PRECURSOR	I.G.1
RSc2290	RS01265							OXIDOREDUCTASE IRON OXIDASE PRECURSOR	I.G.1
RSc2291	RS01264		-2,30 0,00		-0,93 0,01			TRANSGLYCOSYLASE	VI.C
RSc2292	RS01263				-0,66 0,01			TRANSMEMBRANE	VI.C
RSc2293	RS01262							CONSERVED HYPOTHETICAL PROTEIN	VI.C
RSc2294	RS01261				-0,91 0,02			CONSERVED HYPOTHETICAL PROTEIN	VI.C
RSc2295	RS01259				-0,84 0,02			SIGNAL PEPTIDE HYPOTHETICAL PROTEIN	VI.C
RSc2296	RS01258							TRANSMEMBRANE	VI.C
RSc2297	RS01257		-0,89 0,01	1,03 0,00				SIGNAL PEPTIDE	VI.C
RSc2298	RS01256							SIGNAL PEPTIDE COG2132, Multicopper oxidases	I
RSc2299	RS01255							SIGNAL PEPTIDE COG2132, Multicopper oxidases	I
RSc2300	RS01254	0,48 0,04						SIGNAL PEPTIDE COG2165, Type II secretory pathway, pseudopilin PulG - related pr	III.A.2
RSc2301	RS01252							COG2165, Type II secretory pathway, pseudopilin PulG - related protein	III.A.2
RSc2302	RS01251							SIGNAL PEPTIDE COG2165, Type II secretory pathway, pseudopilin PulG - related pr	III.A.2
RSc2303	RS01250							Type II secretory pathway, GSPD-RELATED PROTEIN	III.A.2
RSc2304	RS01248			-0,80 0,04				TRANSMEMBRANE PROLINE RICH PROTEIN	VI.C
RSc2305	RS01247							TRANSMEMBRANE	VI.C
RSc2306	RS01246							TRANSMEMBRANE	VI.C
RSc2307	RS01245							SIGNAL PEPTIDE	VI.C
RSc2308	RS01244							Type II secretory pathway GSPE - RELATED PROTEIN	III.A.2
RSc2309	RS01243							TRANSMEMBRANE Type II secretory pathway, GSPF-RELATED PROTEIN	III.A.2
RSc2310	RS01241				-1,15 0,01			TRANSMEMBRANE Type II secretory pathway, pseudopilin PulG - related protein	III.A.2
RSc2311	RS01240							TRANSCRIPTION REGULATOR TWO-COMPONENT SENSOR HISTIDINE KINASE	VI.A
RSc2312	RS01239							TRANSCRIPTION REGULATOR TWO-COMPONENT RESPONSE REGULATOR	VI.A
RSc2313	RS01237			-0,78 0,00				TRANSPOSASE	V.A
RSc2314	RS01236			-0,66 0,00		0,65 0,01		TRANSPOSASE	V.A
RSc2315	RS01234			-1,24 0,00	-1,00 0,00	1,13 0,00		CONSERVED HYPOTHETICAL PROTEIN	VI.C
RSc2316	RS01233							TRANSMEMBRANE DRUG TRANSPORT PROTEIN	IV.D
RSc2317	RS01232			-0,96 0,01				COG3545, Esterase of the alpha/beta hydrolase fold	I
RSc2318	RS01231				-0,75 0,05	0,59 0,00		TRANSCRIPTION REGULATOR	VI.A
RSc2319	RS01230		0,49 0,04					COG2267, Lysophospholipase	I
RSc2320	RS01229			-0,87 0,01	-1,63 0,00			CONSERVED HYPOTHETICAL PROTEIN	VI.C
RSc2321	RS01228							OXIDOREDUCTASE COG0300, Short-chain dehydrogenases of various substrate spe	I
RSc2322	RS01227			-0,53 0,02	-1,15 0,00			TRANSCRIPTION REGULATOR	VI.A
RSc2323	RS01226			-0,94 0,00				TRANSMEMBRANE TRANSPORTER PROTEIN	IV.D
RSc2324	RS01224			-0,51 0,01				TRANSMEMBRANE TRANSPORT PROTEIN	IV.D
RSc2325	RS01223			-0,55 0,03	-1,07 0,04			TRANSCRIPTION REGULATOR	VI.A
RSc2326	RS01222		-1,17 0,05	-0,43 0,03				LIPOPROTEIN	VI.B
RSc2327	RS01221			-0,48 0,00				TRANSCRIPTION REGULATOR	VI.A
RSc2328	RS01220	-2,78 0,00		0,14 0,02				SIGNAL PEPTIDE COG0596, Hydrolases or acyltransferases (alpha/beta hydrolase s	I
RSc2329	RS01219			0,57 0,01				COG2249, NADPH-quinone reductase (modulator of drug activity B)	VI.C
RSc2330	RS01218							HYPOTHETICAL PROTEIN	VI.D
RSc2335	RS01214			-0,45 0,00			tISRso1(	REMNANT OF ISRso10-TRANSPOSASE	V.A
RSc2336	RS01210			-0,53 0,00				HYPOTHETICAL PROTEIN	VI.D
RSc2337	RS01209		0,64 0,02	-0,32 0,02		0,54 0,01	serS	SERYL-TRNA SYNTHETASE	II.B.6
RSc2338	RS01208	1,90 0,04						COG2256, ATPase related to the helicase subunit of the Holliday junction resolvase	II.B.8
RSc2339	RS01207			-0,30 0,03		1,13 0,01		TRANSMEMBRANE LIPOPROTEIN	VI.C
RSc2340	RS01206			-0,67 0,00	-0,66 0,01	1,15 0,00		SIGNAL PEPTIDE PERIPLASMIC CARRIER PROTEIN	IV.D
RSc2341	RS01205						ftsK	TRANSMEMBRANE CELL DIVISION PROTEIN FTSK	IV.A
RSc2342	RS01204		0,38 0,01	-0,51 0,02			trxB	OXIDOREDUCTASE THIOREDOXIN REDUCTASE	I.F.2
RSc2343	RS01203							CONSERVED HYPOTHETICAL PROTEIN	VI.C
RSc2344	RS01202				-0,86 0,03			TRANSMEMBRANE	VI.C
RSc2345	RS01201			-0,36 0,02			glnB	TRANSCRIPTION REGULATOR NITROGEN REGULATORY PROTEIN P-II	I.A.18
RSc2346	RS01200		0,52 0,01			1,01 0,00		COG2226, Methylase involved in ubiquinone/menaquinone biosynthesis	VI.B
RSc2347	RS01199						nadE	SIGNAL PEPTIDE NH3-DEPENDENT NAD+ SYNTHETASE	I.B.9
RSc2348	RS01198							CONSERVED HYPOTHETICAL PROTEIN	VI.C
RSc2349	RS01197						ppa	INORGANIC PYROPHOSPHATASE	I.F
RSc2350	RS01196							OXIDOREDUCTASE ALDEHYDE DEHYDROGENASE	I.H
RSc2351	RS01195		0,44 0,05			1,04 0,03	tsaC	OXIDOREDUCTASE TOLUENESULFONATE ZINC-INDEPENDENT ALCOHOL DEH'	I.I
RSc2352	RS01194					0,63 0,00		TRANSMEMBRANE PERMEASE	IV.D
RSc2353	RS01193			-0,72 0,01	-0,83 0,04			SIGNAL PEPTIDE	VI.C
RSc2354	RS01192							OXIDOREDUCTASE CYTOCHROME B	I.G
RSc2355	RS01191						hemY	PROTEIN PORPHYRIN BIOSYNTHESIS	I.B.4
RSc2356	RS01190							TRANSMEMBRANE BIFUNCTIONAL PROTEIN: UROPORPHYRIN-III C-METHYLTR	I.B.4
RSc2357	RS01189		0,46 0,02	-0,36 0,00	0,59 0,01	1,00 0,01	hemC	PORPHOBILINOGEN DEAMINASE	I.B.4
RSc2358	RS01188		0,51 0,02				ppc	PHOSPHOENOLPYRUVATE CARBOXYLASE	I.H
RSc2359	RS01187		-4,01 0,00	3,95 0,00	2,81 0,00			HYPOTHETICAL PROTEIN	VI.D
RSc2360	RS01186							CONSERVED HYPOTHETICAL PROTEIN	VI.C
RSc2361	RS01185			-1,02 0,00	-0,75 0,00			TRANSCRIPTION REGULATOR RNA POLYMERASE SIGMA-E FACTOR SIGMA-24	I.E.1
RSc2362	RS01184							TRANSMEMBRANE	VI.C
RSc2363	RS01183						argH	ARGININOSUCCINATE LYASE	I.A.17
RSc2364	RS01182							2H PHOSPHOESTERASE (2'-5' RNA LIGASE)	II.B.6
RSc2365	RS01181		0,80 0,00				adi	BIODEGRADATIVE ARGININE DECARBOXYLASE	I.I.3
RSc2366	RS01180			-0,25 0,02	0,21 0,03		dcd	DEOXYCYTIDINE TRIPHOSPHATE DEAMINASE	I.F.2
RSc2367	RS01179							FDHD PROTEIN HOMOLOG	I.H.2
RSc2368	RS01178						sodC	SUPEROXIDE DISMUTASE CU-ZN PRECURSOR	IV.G.4
RSc2369	RS01177							SIGNAL PEPTIDE	VI.D
RSc2370	RS01176							TRANSMEMBRANE COG2864, Cytochrome B subunit of formate dehydrogenase	I
RSc2371	RS01175							lipoprotein	VI.C
RSc2372	RS01174	-0,34 0,03						OXIDOREDUCTASE FORMATE DEHYDROGENASE (IRON-SULFUR SUBUNIT)	I.H.2
RSc2373	RS01173							OXIDOREDUCTASE FORMATE DEHYDROGENASE (LARGE SUBUNIT)	I.H.2
RSc2374	RS01172	1,66 0,02		-2,69 0,00	-2,17 0,01	0,77 0,01		TRANSMEMBRANE	VI.D
RSc2375	RS01171			-0,68 0,02				CONSERVED HYPOTHETICAL PROTEIN	VI.C
RSc2376	RS01170		-2,94 0,00	2,62 0,00	4,10 0,00			FERREDOXIN	I
RSc2377	RS01168			-0,63 0,00				PROLINE RICH PROTEIN	VI.C
RSc2378	RS01167			-0,42 0,02				CONSERVED HYPOTHETICAL PROTEIN	VI.C
RSc2379	RS01166						mrp	MRP ATPase involved in chromosome partitioning	IV.A
RSc2380	RS01165			-0,43 0,00				TRANSMEMBRANE COG2885, Outer membrane protein and related peptidoglycan-a	III.B.2
RSc2381	RS01164		0,63 0,04	-0,42 0,00			metG1	METHIONYL-tRNA SYNTHETASE	II.D.1
RSc2382	RS01163		0,41 0,05	-0,54 0,01				LIPOPROTEIN	VI.C
RSc2384	RS02766		0,55 0,03	-0,44 0,03				COG0729, Outer membrane protein	III.B.2
RSc2385	RS02765							CONSERVED HYPOTHETICAL PROTEIN	VI.C
RSc2386	RS02764			-0,24 0,02	1,26 0,00	1,59 0,01		CONSERVED HYPOTHETICAL PROTEIN	VI.C
RSc2387	RS02763		0,57 0,05		0,46 0,04		panC	PANTOATE--BETA-ALANINE LIGASE	I.B.11
RSc2388	RS02761			-0,33 0,00		1,12 0,00		CONSERVED HYPOTHETICAL PROTEIN	VI.C
RSc2389	RS02760					0,56 0,00		PARTITION-RELATED PROTEIN	V.B
RSc2390	RS02759						cobQ	COBYRIC ACID SYNTHASE	I.B.3

RSc2391	RS02758			-0,58 0,00			cobP	COBALAMIN BIOSYNTHESIS BIFUNCTIONAL PROTEIN: COBINAMIDE KINASE AI	I.B.3
RSc2392	RS02743						cobD	TRANSMEMBRANE COBALAMIN BIOSYNTHESIS PROTEIN COBD	I.B.3
RSc2393	RS02729		1,03 0,04				cobC	COBALAMIN BIOSYNTHESIS PROTEIN PYRIDOXAL-PHOSPHATE-DEPENDENT /	I.B.3
RSc2394	RS02728				-0,61 0,03			ABC TRANSPORTER ABC-TYPE TRANSPORTER, PERIPLASMIC COMPONENT	IV.D
RSc2395	RS02727						gpmB	COG0406, Fructose-2,6-bisphosphatase	I
RSc2396	RS02726		0,90 0,00		0,87 0,04		cobS	TRANSMEMBRANE COBALAMIN 5'-PHOSPHATE SYNTHASE EC 2.-.-.-	I.B.3
RSc2397	RS02725						cobU	TRANSMEMBRANE NICOTINATE-NUCLEOTIDE--DIMETHYLBENZIMIDAZOLE PH(	I.B.3
RSc2398	RS02720			0,79 0,00	1,07 0,03			ABC TRANSPORTER ATP-BINDING PROTEIN	IV.D
RSc2399	RS02719		1,23 0,05					ABC TRANSPORTER TRANSMEMBRANE PROTEIN	IV.D
RSc2400	RS02718		0,92 0,00	-0,31 0,02	0,86 0,01			TONB-DEPENDENT RECEPTOR PROTEIN	III.B.2
RSc2401	RS02717			-0,46 0,03	0,86 0,01			CONSERVED HYPOTHETICAL PROTEIN	VI.C
RSc2402	RS02716			-0,53 0,01	0,83 0,00			CONSERVED HYPOTHETICAL PROTEIN	VI.C
RSc2403	RS02715		0,32 0,02	-0,33 0,02				CONSERVED HYPOTHETICAL PROTEIN	VI.C
RSc2404	RS05747			-1,60 0,00	-1,62 0,00			SIGNAL PEPTIDE HYPOTHETICAL	VI.B
RSc2406	RS02787			-0,60 0,00			tISRso14	ISRso14-TRANSPOSASE ORFA	V.A
RSc2407	RS05754			-0,75 0,00				HYPOTHETICAL PROTEIN	VI.D
RSc2410	RS02708			-0,28 0,04	0,68 0,05		lgt	TRANSMEMBRANE PROLIPOPROTEIN DIACYLGLYCERYL TRANSFERASE	II.B.6
RSc2411	RS02707		0,27 0,04	-0,60 0,00	-1,37 0,01	-1,19 0,00		TRANSCRIPTION REGULATOR	VI.A
RSc2412	RS02706						ilvD	DIHYDROXY-ACID DEHYDRATASE	I.A.2
RSc2413	RS02702		0,45 0,02	-0,38 0,02	1,05 0,00			HYPOTHETICAL PROTEIN	VI.B
RSc2414	RS02701			-0,35 0,01	1,35 0,02		holC	DNA POLYMERASE III (CHI SUBUNIT)	II.B.8
RSc2415	RS02700			-0,37 0,02			pepA	CYTOSOL AMINOPEPTIDASE	II.A.4
RSc2416	RS02698		0,76 0,01					TRANSMEMBRANE COG0795, Permease	IV.D
RSc2417	RS02697	0,76 0,05	0,63 0,00					TRANSMEMBRANE COG0795, Permease	IV.D
RSc2418	RS02695			-0,67 0,02				CONSERVED HYPOTHETICAL PROTEIN	VI.C
RSc2419	RS02694						hpaF	5-CARBOXYMETHYL-2-HYDROXYMUCONATE DELTA-ISOMERASE	I.I.4
RSc2420	RS02693						nirE	UROPORPHYRINOGEN-III C-METHYLTRANSFERASE	I.H.3
RSc2421	RS02692		0,89 0,02	0,58 0,03	0,60 0,00	-1,36 0,04	cysN	SULFATE ADENYLYLTRANSFERASE SUBUNIT 1	I.A.9
RSc2422	RS02690		0,92 0,00	0,68 0,00		-0,78 0,00	cysD	SULFATE ADENYLYLTRANSFERASE SUBUNIT 2	I.A.9
RSc2423	RS02689				0,88 0,00	-0,78 0,01	cysH	5' ADENYLYLSULFATE APS REDUCTASE	I.A.9
RSc2424	RS02687							CONSERVED HYPOTHETICAL PROTEIN	VI.C
RSc2425	RS02684			1,07 0,00			cysI1	OXIDOREDUCTASE SULFITE REDUCTASE	I.F.3
RSc2426	RS02683		0,35 0,02		-1,50 0,00	-1,10 0,00		TRANSMEMBRANE COG0730, Permease	VI.C
RSc2427	RS02682			-0,35 0,01		-0,46 0,05	cysB2	TRANSCRIPTION REGULATOR CYS REGULON TRANSCRIPTIONAL ACTIVATOR-	I.A.9
RSc2428	RS02680			0,52 0,00				ABC TRANSPORTER AMINO ACID-BINDING PERIPLASMIC PROTEIN (PBP)	IV.D.2
RSc2429	RS02679		0,59 0,02	-0,46 0,00				OXIDOREDUCTASE COG2084, 3-hydroxyisobutyrate dehydrogenase and related bet	I
RSc2430	RS02678							TRANSCRIPTION REGULATOR	VI.A
RSc2431	RS02676							TRANSMEMBRANE COG0038, Chloride channel protein	IV.D.1
RSc2432	RS02675			-0,60 0,00	-0,76 0,03			TRANSMEMBRANE SIGNAL TRANSDUCTION PROTEIN GGDEF DOMAIN	VI.A
RSc2433	RS02671			-0,72 0,03	-0,98 0,02			COG0451, Nucleoside-diphosphate-sugar epimerase	I
RSc2434	RS02670		0,55 0,00					OXIDOREDUCTASE REDUCTASE	III
RSc2435	RS02668		0,75 0,00	-0,50 0,01		-0,60 0,00	argM	SUCCINYLORNITHINE TRANSAMINASE AND ACETYLORNITHINE AMINOTRANS	I.I.3
RSc2436	RS02667		1,40 0,00	-0,59 0,00				ACETYLTRANSFERASE	I
RSc2437	RS02666		0,92 0,00	-0,41 0,03				ABC TRANSPORTER AMINO ACID ATP-BINDING PROTEIN	IV.D.2
RSc2438	RS02664		0,90 0,00	-0,44 0,04				ABC TRANSPORTER AMINO ACID ATP-BINDING PROTEIN	IV.D.2
RSc2439	RS02663		0,78 0,01					ABC TRANSPORTER AMINO-ACID TRANSMEMBRANE PROTEIN	IV.D.2
RSc2440	RS01355		0,72 0,00		-1,34 0,01		livH2	ABC TRANSPORTER AMINO-ACID TRANSMEMBRANE PROTEIN	IV.D.2
RSc2441	RS01353		0,88 0,01					ABC TRANSPORTER AMINO ACID-BINDING PERIPLASMIC PROTEIN	IV.D.2
RSc2442	RS01352		1,58 0,01				lytB	LYTB PROTEIN	IV.G.2
RSc2443	RS01351			-0,60 0,00			fkpB	FKBP-TYPE 16 KDA PEPTIDYL-PROLYL CIS-TRANS ISOMERASE	II.B.7
RSc2444	RS01350			-0,30 0,01			radC	DNA REPAIR PROTEIN RADC	II.B.8
RSc2445	RS01349			-0,32 0,00		-0,35 0,00	rpmB	50S RIBOSOMAL SUBUNIT PROTEIN L28	III.C.2
RSc2446	RS01348			-0,18 0,03	-0,24 0,01	-0,72 0,01	rpmG	50S RIBOSOMAL SUBUNIT PROTEIN L33	III.C.2
RSc2447	RS01159		0,69 0,04				nadB1	OXIDOREDUCTASE L-ASPARTATE OXIDASE (QUINOLINATE SYNTHETASE B)	I.B.9
RSc2448	RS01158						nadC	NICOTINATE-NUCLEOTIDE PYROPHOSPHORYLASE (CARBOXYLATING) QUINO	I.B.9
RSc2449	RS01157			-0,35 0,00			nadA	QUINOLINATE SYNTHETASE A	I.B.9
RSc2450	RS01156			-0,58 0,01				OXIDOREDUCTASE STEAROYL-COA DESATURASE	I.C
RSc2451	RS01155			-0,40 0,01				TRANSMEMBRANE COG3264, Small-conductance mechanosensitive channel	VI.C
RSc2452	RS01154			-0,33 0,00				ENDONUCLEASE	II.A.1
RSc2453	RS01153			-0,48 0,04				COG0144, tRNA and rRNA cytosine-C5-methylase	II
RSc2454	RS01151		0,48 0,04				purN	PHOSPHORIBOSYLGLYCINAMIDE FORMYLTRANSFERASE	I.D.2
RSc2455	RS01150			-0,61 0,00			vsrC	TRANSCRIPTION REGULATOR RESPONSE REGULATOR VSRC	I.E.1
RSc2456	RS01149			-0,22 0,05			vsrB	TRANSCRIPTION REGULATOR COMPOSITE TWO-COMPONENT REGULATORY F	I.E.1
RSc2457	RS01148			-0,34 0,00			ribF	RIBOFLAVIN BIOSYNTHESIS PROTEIN	I.B.7
RSc2458	RS01147						ileS	ISOLEUCYL-TRNA SYNTHETASE	II.B.6
RSc2459	RS01146					-0,37 0,03	lspA	LIPOPROTEIN SIGNAL PEPTIDASE	II.B.7
RSc2460	RS01145			-0,64 0,01				TRANSMEMBRANE	VI.C
RSc2461	RS01144			-0,46 0,01			dfp	DNA/PANTOTHENATE METABOLISM FLAVOPROTEIN FLAVOPROTEIN	II.B.8
RSc2462	RS01143			-0,52 0,00				OXIDOREDUCTASE COG2141, Coenzyme F420-dependent N5,N10-methylene tetral	I
RSc2463	RS01142						dut	DEOXYURIDINE 5'-TRIPHOSPHATE NUCLEOTIDOHYDROLASE DUTPASE	I.F.2
RSc2464	RS01141						clpA	ATP-DEPENDENT PROTEASE (ATP-BINDING SPECIFICITY SUBUNIT)	II.A.4
RSc2465	RS01140							CONSERVED HYPOTHETICAL PROTEIN	VI.C
RSc2466	RS01139			-0,71 0,00	-0,74 0,00		cspD	TRANSCRIPTION REGULATOR COLD SHOCK-LIKE PROTEIN CSPD	VI.A
RSc2467	RS01138			-1,43 0,00				TRANSMEMBRANE	VI.C
RSc2468	RS01137			-0,98 0,02	-1,11 0,02			TRANSMEMBRANE	VI.D
RSc2469	RS01136							HYPOTHETICAL PROTEIN	VI.D
RSc2470	RS01135							RHS-RELATED PROTEIN	VI.B
RSc2471	RS01134							HYPOTHETICAL PROTEIN	VI.D
RSc2472	RS01133			-0,39 0,04				TRANSMEMBRANE	VI.D
RSc2473	RS01132			-0,30 0,03	-0,66 0,02			HYPOTHETICAL PROTEIN	VI.D
RSc2474	RS01131			-0,38 0,03				HYPOTHETICAL PROTEIN	VI.D
RSc2475	RS01130							HYPOTHETICAL PROTEIN	VI.D
RSc2476	RS01129							BACTERIOPHAGE-RELATED PROTEIN	V.D
RSc2477	RS01127							PROPHAGE PROTEIN	V.D
RSc2478	RS01126			-0,36 0,00				TRANSMEMBRANE PROPHAGE PROTEIN	V.D
RSc2479	RS01124							BACTERIOPHAGE-RELATED PROTEIN	V.D
RSc2480	RS01121			-0,29 0,01				CONSERVED HYPOTHETICAL PROTEIN	VI.C
RSc2481	RS01119		-0,18 0,02	-0,22 0,00				CONSERVED HYPOTHETICAL PROTEIN	VI.C
RSc2482	RS01114			-0,48 0,00				CONSERVED HYPOTHETICAL PROTEIN	VI.C
RSc2483	RS01112							CONSERVED HYPOTHETICAL PROTEIN	VI.C
RSc2484	RS01111							HYPOTHETICAL PROTEIN	VI.D
RSc2485	RS01110			-0,83 0,00				HYPOTHETICAL PROTEIN	VI.D
RSc2486	RS01109				-0,72 0,00	-1,76 0,00		CONSERVED HYPOTHETICAL PROTEIN	VI.C
RSc2487	RS05745			-0,43 0,01	-1,13 0,02			HYPOTHETICAL PROTEIN	VI.D
RSc2488	RS05744			0,74 0,00				HYPOTHETICAL PROTEIN	VI.D
RSc2489	RS01107		0,53 0,02	-0,47 0,00				INTEGRASE	V.D
RSc2490	RS01106		0,33 0,01	-0,20 0,01	0,45 0,01		icd	OXIDOREDUCTASE ISOCITRATE DEHYDROGENASE [NADP]	I.H.6
RSc2491	RS01105				-0,77 0,01	-1,03 0,01		SIGNAL PEPTIDE	VI.C
RSc2492	RS01104							TRANSMEMBRANE	VI.C
RSc2493	RS01103							SIGNAL PEPTIDE COG1858, Cytochrome c peroxidase	I
RSc2494	RS01102		0,41 0,02	-0,41 0,01				SIGNAL PEPTIDE	VI.C
RSc2495	RS01101		0,46 0,03	-0,61 0,01	0,12 0,02			ARYLSULFATASE PRECURSOR (ARYL-SULFATE SULPHOHYDROLASE)	II.A.3
RSc2496	RS01100			-0,74 0,00				SIGNAL PEPTIDE	VI.B
RSc2497	RS01099			-0,34 0,00				TRANSCRIPTION REGULATOR	VI.A
RSc2498	RS01098			-0,41 0,02				TRANSCRIPTION REGULATOR	VI.A
RSc2499	RS01097							MULTIDRUG RESISTANCE PROTEIN	IV.D.7
RSc2500	RS01095			-0,88 0,00	-1,02 0,01			TRANSMEMBRANE	VI.C
RSc2501	RS01094					0,21 0,01		PUTATIVE GAMMA-GLUTAMYLTRANSPEPTIDASE	I.B.6
RSc2502	RS01093							TRANSMEMBRANE	VI.D
RSc2503	RS01092		1,02 0,00					TRANSMEMBRANE COG2321, Predicted metalloprotease	II.A.4
RSc2504	RS01091							OXIDOREDUCTASE COG1028, Dehydrogenases with different specificities (related to	I
RSc2505	RS05743			-0,54 0,05				TRANSCRIPTION REGULATOR	VI.A

RSc2506	RS01080						ogt	METHYLATED-DNA--PROTEIN-CYSTEINE METHYLTRANSFERASE	II.B.8
RSc2507	RS01075							TRANSMEMBRANE	VI.C
RSc2508	RS01074				0,44 0,05			CONSERVED HYPOTHETICAL PROTEIN	VI.C
RSc2509	RS01073			-1,53 0,00	-1,58 0,00	-1,21 0,00		HYPOTHETICAL PROTEIN	VI.D
RSc2510	RS01072		-0,47 0,03	0,56 0,03	-1,16 0,00			SIGNAL PEPTIDE COG1638, TRAP-type C4-dicarboxylate transport system, periplası	IV.D.3
RSc2511	RS01071			-0,81 0,03				SIGNAL PEPTIDE	VI.B
RSc2512	RS01070							SIGNAL PEPTIDE CYCLOHEXADIENYL DEHYDRATASE PRECURSOR	I.A.8
RSc2513	RS01069							HYPOTHETICAL PROTEIN	VI.D
RSc2514	RS01068			-0,71 0,00		0,92 0,00		SIGNAL PEPTIDE	VI.C
RSc2515	RS01067	0,63 0,04		-0,99 0,00	-0,85 0,02			COG2206, HD-GYP domain	VI.A
RSc2516	RS01066			-0,53 0,04	-1,28 0,05			TRANSMEMBRANE ABC TYPE TRANSPORTER : PERIPLASMIC GLYCINE BETAIN	IV.D
RSc2517	RS01065							TRANSMEMBRANE CONSERVED HYPOTHETICAL PROTEIN	VI.C
RSc2518	RS01064	1,10 0,00	0,53 0,03	-1,25 0,01				TRANSMEMBRANE COG2610, H+/gluconate symporter and related permeases	IV.D.3
RSc2519	RS01063			-1,45 0,00	-1,23 0,00			HYPOTHETICAL PROTEIN	VI.D
RSc2520	RS01062	-0,44 0,03						CONSERVED HYPOTHETICAL PROTEIN	VI.C
RSc2521	RS01061				0,90 0,00		hupB2	DNA-BINDING PROTEIN HU-BETA (NS1) (HU-1)	II.C.1
RSc2522	RS01060				-1,47 0,00			CONSERVED HYPOTHETICAL PROTEIN	VI.C
RSc2523	RS01059		0,71 0,00	-0,62 0,00				TRANSMEMBRANE	VI.C
RSc2524	RS01058		0,52 0,05	-0,70 0,01	-1,79 0,00			TRANSMEMBRANE COG1994, Zn-dependent protease	II.A.4
RSc2525	RS01057				1,25 0,04			HYPOTHETICAL PROTEIN	VI.B
RSc2526	RS01055			0,88 0,00	0,89 0,02	0,99 0,00	sodB	SUPEROXIDE DISMUTASE [FE]	IV.G.4
RSc2527	RS01054						xseA	EXODEOXYRIBONUCLEASE VII LARGE SUBUNIT	II.A.1
RSc2528	RS01053			-0,60 0,01			exbB2	TRANSMEMBRANE BIOPOLYMER TRANSPORT EXBB-LIKE PROTEIN	IV.D
RSc2529	RS01052			-0,52 0,02		-0,90 0,02	exbD2	TRANSMEMBRANE BIOPOLYMER TRANSPORT EXBD-RELATED PROTEIN	IV.D
RSc2530	RS01051			-0,41 0,00	0,29 0,04	-0,30 0,00	lpxK	TETRAACYLDISACCHARIDE 4'-KINASE	II.B.2
RSc2531	RS05763		0,41 0,04	-0,45 0,00				CONSERVED HYPOTHETICAL PROTEIN	VI.C
RSc2532	RS05764		0,42 0,01	-0,20 0,01			kdsB	3-DEOXY-MANNO-OCTULOSONATE (CMP-KDO SYNTHETASE)(CKS)	III.A.1
RSc2533	RS05765					-0,96 0,00	adk	ADENYLATE KINASE (ATP-AMP TRANSPHOSPHORYLASE)	I.D.2
RSc2534	RS05766		0,97 0,01					OXIDOREDUCTASE 3-HYDROXYACYL-CoA DEHYDROGENASE TYPE II	I.I.1
RSc2535	RS00706							TRANSCRIPTION REGULATOR	VI.A
RSc2536	RS00704			-1,71 0,00				OXIDOREDUCTASE TRANSMEMBRANE ALDEHYDE DEHYDROGENASE	I.I.4
RSc2537	RS00708			-0,61 0,00	-0,85 0,00			TRANSCRIPTION REGULATOR TRANSCRIPTION REGULATOR	VI.A
RSc2538	RS00712							TRANSMEMBRANE COG0385, Na+-dependent transporter	IV.D
RSc2539	RS00715						amiC	N-ACETYLMURAMOYL-L-ALANINE AMIDASE AMIC PRECURSOR	III.B.3
RSc2540	RS00716			-0,53 0,00				COG0802, ATPase or kinase	VI.C
RSc2541	RS00718							COG1600, Uncharacterized Fe-S protein	I.H
RSc2542	RS00721			-0,50 0,01				TRANSMEMBRANE LIPOPROTEIN	VI.C
RSc2543	RS00722			-0,51 0,00				Methylated-DNA--protein-cysteine methyltransferase	II.B.8
RSc2544	RS00723			-0,41 0,05	-0,46 0,03			INTEGRASE/RECOMBINASE	II.B.8
RSc2545	RS00724	-1,27 0,05						CONSERVED HYPOTHETICAL PROTEIN	VI.C
RSc2546	RS00725		-0,73 0,01	0,73 0,00		-0,29 0,01		TRANSMEMBRANE	VI.C
RSc2547	RS00726		0,38 0,01				aac	TRANSMEMBRANE ACYL-HOMOSERINE LACTONE ACYLASE	I.I
RSc2548	RS00727			-0,52 0,00				TRANSMEMBRANE INOSINE-URIDINE PREFERRING NUCLEOSIDE HYDROLASE	I.F.12
RSc2549	RS00728		0,40 0,00					CONSERVED HYPOTHETICAL PROTEIN	VI.C
RSc2550	RS00729						murB	OXIDOREDUCTASE UDP-N-ACETYLENOLPYRUVOYLGLUCOSAMINE REDUCTAS	III.B.3
RSc2551	RS00730							COG1752, Predicted esterase of the alpha-beta hydrolase superfamily	I
RSc2552	RS00731			-0,30 0,05		-0,53 0,01		CONSERVED HYPOTHETICAL PROTEIN	VI.C
RSc2553	RS00732		0,57 0,02			-0,54 0,01	argG	ARGININOSUCCINATE SYNTHASE	I.A.17
RSc2554	RS00733						argF	ORNITHINE CARBAMOYLTRANSFERASE	I.A.17
RSc2555	RS00734			-0,45 0,03				CONSERVED HYPOTHETICAL PROTEIN	VI.C
RSc2556	RS00735			-0,44 0,00		-0,85 0,00	rpsT	30S RIBOSOMAL SUBUNIT PROTEIN S20	III.C.2
RSc2557	RS00736			-0,52 0,04				TRANSMEMBRANE	VI.C
RSc2558	RS00740							CONSERVED HYPOTHETICAL PROTEIN	VI.C
RSc2559	RS00742					-1,09 0,00	dedA	TRANSMEMBRANE DEDA PROTEIN	VI.C
RSc2560	RS00743	0,69 0,05		-0,70 0,02				TRANSCRIPTION REGULATOR	VI.A
RSc2561	RS00746		1,03 0,02		-1,31 0,01			LIPOPROTEIN	VI.C
RSc2562	RS00750							TRANSMEMBRANE	VI.C
RSc2563	RS00753		0,61 0,01		0,85 0,00	-0,37 0,02	mutL	DNA MISMATCH REPAIR PROTEIN	II.B.8
RSc2564	RS00755						miaA	TRNA DELTA2-ISOPENTENYLPYROPHOSPHATE TRANSFERASE	II.D.1
RSc2565	RS00758		0,22 0,02					ACETYLTRANSFERASE	I
RSc2566	RS00760				1,62 0,00	2,27 0,00		METHYLATED-DNA--PROTEIN-CYSTEINE METHYLTRANSFERASE	II.B.8
RSc2567	RS00763			-1,14 0,00				CONSERVED HYPOTHETICAL PROTEIN	VI.C
RSc2568	RS00764			-0,42 0,00			alkB	ALKYLATED DNA REPAIR	II.B.8
RSc2569	RS00766			-0,56 0,01			alkA	DNA-3-METHYLADENINE GLYCOSYLASE	II.B.8
RSc2570	RS00768		0,83 0,03	-0,30 0,02			ada	TRANSCRIPTION REGULATOR ADA REGULATORY PROTEIN OF ADAPTATIVE R	II.B.8
RSc2571	RS00770							CONSERVED HYPOTHETICAL PROTEIN	VI.C
RSc2572	RS00771							LIPOPROTEIN	VI.D
RSc2573	RS00774			-0,43 0,00			tISRso1	ISRso15-TRANSPOSASE	V.A
RSc2574	RS00781							CONSERVED HYPOTHETICAL PROTEIN	VI.C
RSc2575	RS00938						trbI	TRANSMEMBRANE CONJUGAL TRANSFER PROTEIN TRBI	V.B
RSc2576	RS00782						trbG	CONJUGAL TRANSFER LIPOPROTEIN TRBG	V.B
RSc2577	RS00783			-0,56 0,05			trbF	TRANSMEMBRANE CONJUGAL TRANSFER PROTEIN TRBF	V.B
RSc2578	RS00784						trbL	TRANSMEMBRANE CONJUGAL TRANSFER PROTEIN TRBL	V.B
RSc2579	RS00786							LIPOPROTEIN	VI.C
RSc2580	RS00788						trbJ	SIGNAL PEPTIDE CONJUGAL TRANSFER PROTEIN TRBJ	V.B
RSc2581	RS00789						trbE	CONJUGAL TRANSFER PROTEIN TRBE	V.B
RSc2582	RS00791						trbD	TRANSMEMBRANE CONJUGAL TRANSFER PROTEIN TRBD	V.B
RSc2583	RS00793						trbC	TRANSMEMBRANE CONJUGAL TRANSFER PROTEIN TRBC	V.B
RSc2584	RS00795						trbB	CONJUGAL TRANSFER PROTEIN TRBB	V.B
RSc2585	RS00797							HYPOTHETICAL/UNKNOWN	VI.C
RSc2586	RS00798		0,82 0,02				traG	TRANSMEMBRANE Plasmid Conjugation protein TraG	V.B
RSc2587	RS00835			-0,55 0,00				LIPOPROTEIN	VI.C
RSc2588	RS00836			-0,56 0,00			traR	TRANSCRIPTION REGULATOR PLASMID REPLICATION REGULATORY PROTEIN	V.B
RSc2589	RS00837			-0,65 0,00	-0,96 0,00		limA	LIMONENE-1,2-EPOXIDE HYDROLASE	I.I.4
RSc2590	RS00838			-0,50 0,00			limA2	LIMONENE-1,2-EPOXIDE HYDROLASE	I.I.4
RSc2591	RS00839		0,71 0,01	-0,46 0,00		-1,40 0,00		TRANSCRIPTION REGULATOR TRANSCRIPTIONAL REGULATOR	VI.A
RSc2592	RS00840							CONSERVED HYPOTHETICAL PROTEIN	VI.C
RSc2593	RS00841				-0,72 0,02			COG2272, Carboxylesterase type B	VI.C
RSc2594	RS00842						gstJ	GLUTATHIONE-S-TRANSFERASE	II.B.7
RSc2595	RS00843							CONSERVED HYPOTHETICAL PROTEIN	VI.C
RSc2596	RS00844		0,77 0,02	-0,29 0,01				COG1335, Amidase related to nicotinamidase	I
RSc2597	RS00845							OXIDOREDUCTASE COG1028, Dehydrogenases with different specificities (related tı	I
RSc2598	RS00846							TRANSCRIPTION REGULATOR	VI.A
RSc2599	RS00847			-1,02 0,03				HYPOTHETICAL PROTEIN	VI.B
RSc2600	RS00848			-0,48 0,00				HYPOTHETICAL PROTEIN	VI.B
RSc2601	RS00849							COG3843, Type IV secretory pathway VirD2 (relaxase) - related protein	V.B
RSc2602	RS00850						traF	TRANSMEMBRANE CONJUGAL TRANSFER PROTEIN TRAF	V.B
RSc2603	RS00851							CONSERVED HYPOTHETICAL PROTEIN	VI.C
RSc2604	RS00852							CONSERVED HYPOTHETICAL PROTEIN	VI.C
RSc2605	RS00853				-0,69 0,05		parA3	PARTITION PROTEIN	V.B
RSc2606	RS00854							REPLICATION INITIATION REPA - LIKE PROTEIN	V.B
RSc2607	RS00855			-0,55 0,05				CONSERVED HYPOTHETICAL PROTEIN	VI.C
RSc2608	RS00857			-0,28 0,03		-1,00 0,02		CONSERVED HYPOTHETICAL PROTEIN	VI.C
RSc2609	RS00858			-0,48 0,04				lipoprotein	VI.B
RSc2610	RS00859				-0,49 0,01			TRANSCRIPTION REGULATOR	VI.A
RSc2611	RS00860				-0,54 0,04			CONSERVED HYPOTHETICAL PROTEIN	VI.C
RSc2612	RS00912							TRANSMEMBRANE	VI.C
RSc2613	RS00913							CONSERVED HYPOTHETICAL PROTEIN	VI.C
RSc2614	RS00916							CONSERVED HYPOTHETICAL PROTEIN	VI.C
RSc2615	RS00917				-0,56 0,03	-0,27 0,02		CONSERVED HYPOTHETICAL PROTEIN	VI.C
RSc2616	RS00918			-0,75 0,00				CONSERVED HYPOTHETICAL PROTEIN	VI.C
RSc2617	RS00919				-1,29 0,01			CONSERVED HYPOTHETICAL PROTEIN	VI.C

RSc2618	RS00920												F plasmid gene 32-like protein	VI.C
RSc2619	RS00923												CONSERVED HYPOTHETICAL PROTEIN	VI.C
RSc2620	RS00924											radC2	DNA REPAIR RADC-LIKE PROTEIN	II.B.8
RSc2621	RS00925					-1,00	0,00	-1,27	0,05				CONSERVED HYPOTHETICAL PROTEIN	VI.C
RSc2622	RS00926											int	INTEGRASE-TYROSINE RECOMBINASE	V.A
RSc2623	RS00927									-0,74	0,01	purM	PHOSPHORIBOSYLFORMYLGLYCINAMIDINE CYCLO-LIGASE (AIRS)	I.D.2
RSc2624	RS00928												PERMEASE TRANSMEMBRANE TRANSPORT	IV.D
RSc2625	RS00929			0,49	0,01								COG0593, ATPase involved in DNA replication initiation	II.B.8
RSc2626	RS00930					-0,43	0,00						COG0560, Phosphoserine phosphatase	I
RSc2627	RS00931			0,63	0,04	-0,45	0,00	0,53	0,04			pcnB	POLYNUCLEOTIDE ADENYLYLTRANSFERASE	II.B.6
RSc2628	RS00932					-0,71	0,01			0,48	0,03	folK	2-AMINO-4-HYDROXY-6-HYDROXYMETHYLDIHYDROPTERIDINE PYROPHOSPH	I.B.14
RSc2629	RS00933			0,64	0,03	-0,66	0,01						DEOXYGUANOSINE KINASE (DGUO KINASE) (DGK)	I.F.2
RSc2630	RS00934			1,00	0,00	-0,47	0,00					panB	3-METHYL-2-OXOBUTANOATE HYDROXYMETHYLTRANSFERASE	I.B.11
RSc2631	RS00935			0,99	0,00	-0,45	0,02						ABC TRANSPORTER TRANSMEMBRANE PROTEIN	IV.D
RSc2632	RS00936					-1,03	0,00						ABC TRANSPORTER ATP-BINDING PROTEIN	IV.D
RSc2633	RS05772			0,75	0,03	-0,48	0,02					pabB	PARA-AMINOBENZOATE SYNTHETASE COMPONENT I	I.B.14
RSc2634	RS04588			1,01	0,00			0,68	0,01	-1,77	0,03	dnaJ	CHAPERONE PROTEIN	IV.B
RSc2635	RS04587									-0,73	0,00	dnaK	HEAT SHOCK PROTEIN 70 (HSP70) CHAPERONE	IV.B
RSc2636	RS04586			0,67	0,05							rnk	TRANSCRIPTION REGULATOR REGULATOR OF NUCLEOSIDE DIPHOSPHATE K	VI.A
RSc2637	RS04584												HYPOTHETICAL PROTEIN	VI.D
RSc2638	RS04583									-0,51	0,02		CONSERVED HYPOTHETICAL PROTEIN	VI.C
RSc2639	RS04582			0,41	0,05							grpE	HEAT SHOCK PROTEIN 24 (HSP-70 COFACTOR)	IV.B
RSc2640	RS04580							-1,28	0,02				TRANSPOSASE REMNANT	V.A
RSc2641	RS04575					-1,46	0,00	-1,50	0,00	1,03	0,02		TRANSMEMBRANE	VI.D
RSc2642	RS04574									-0,53	0,03	hslR	HEAT SHOCK PROTEIN 15	IV.F.1
RSc2643	RS04573	-0,64	0,05									hemH	FERROCHELATASE (PROTOHEME FERRO-LYASE)	I.B.4
RSc2644	RS04572											hutI	TRANSMEMBRANE IMIDAZOLONE-5-PROPIONATE HYDROLASE	I.I.3
RSc2645	RS04571												ARGINASE	I.I.3
RSc2646	RS04570											hutH1	HISTIDINE AMMONIA-LYASE (HISTIDASE)	I.I.3
RSc2647	RS04569											hutU	UROCANATE HYDRATASE (UROCANASE)	I.I.3
RSc2648	RS04568							0,48	0,00			hutC	TRANSCRIPTION REGULATOR HISTIDINE UTILIZATION REPRESSOR	I.A.4
RSc2649	RS04567					-0,44	0,00					hrcA	TRANSCRIPTION REGULATOR HEAT-INDUCIBLE TRANSCRIPTION REPRESSOR	IV.B
RSc2650	RS04566			0,52	0,01	-0,15	0,04	0,42	0,01				COG0061, Sugar kinase	I
RSc2651	RS04565											recN	DNA REPAIR PROTEIN	II.B.8
RSc2652	RS04564			-0,95	0,04	-0,51	0,01						LIPOPROTEIN	VI.B
RSc2653	RS04563			0,39	0,02	-0,39	0,00	0,48	0,02				SIGNAL PEPTIDE PROTEASE	II.A.4
RSc2654	RS04562			0,68	0,02								SERINE PROTEASE	II.A.4
RSc2655	RS04561												TRANSMEMBRANE	VI.C
RSc2656	RS04556					-0,23	0,05					glnE	GLUTAMATE-AMMONIA-LIGASE ADENYLYLTRANSFERASE (GLUTAMINE- SYNT	I.F.5
RSc2657	RS04554												TRANSMEMBRANE	VI.C
RSc2658	RS04553					-0,59	0,03						COG0388, Amidohydrolase	I.I
RSc2659	RS04552											tldD	COG0312, Zn-dependent protease	II.A.4
RSc2660	RS04547			0,64	0,02							aroG1	PHOSPHO-2-DEHYDRO-3-DEOXYHEPTONATE ALDOLASE, PHE-SENSITIVE (DA	I.A.5
RSc2661	RS04546			-3,32	0,00	5,15	0,00	3,27	0,00				TRANSCRIPTION REGULATOR	VI.A
RSc2662	RS04545												TRANSMEMBRANE COG0668, Small-conductance mechanosensitive channel	IV.D
RSc2663	RS04544			0,85	0,02								CONSERVED HYPOTHETICAL PROTEIN	VI.C
RSc2664	RS04542											dld	OXIDOREDUCTASE D-LACTATE DEHYDROGENASE (cytochrome)	I
RSc2665	RS04540			0,91	0,04	-1,01	0,02						TRANSCRIPTION REGULATOR	VI.A
RSc2666	RS04539					0,60	0,02					glcD	OXIDOREDUCTASE GLYCOLATE OXIDASE (FAD-LINKED SUBUNIT)	I.I.4
RSc2667	RS04538							-0,31	0,02			glcE	OXIDOREDUCTASE GLYCOLATE OXIDASE SUBUNIT	I.F.18
RSc2668	RS04537							-0,48	0,04			glcF	GLYCOLATE OXIDASE (IRON-SULFUR SUBUNIT)	I.F.18
RSc2669	RS04536					-0,60	0,00					lrgA	TRANSMEMBRANE COG1380, Effector of murein hydrolase LrgA	VI.C
RSc2670	RS04535											lrgB	TRANSMEMBRANE COG1346, Effector of murein hydrolase	VI.C
RSc2671	RS04534							0,68	0,05				COG3324, Enzyme related to lactoylglutathione lyase	I
RSc2672	RS04533			-0,54	0,04	1,44	0,00						CONSERVED HYPOTHETICAL PROTEIN	VI.C
RSc2673	RS00048					-0,91	0,00	-0,96	0,00				TRANSCRIPTION REGULATOR REPRESSOR	VI.A
RSc2674	RS00047					-0,50	0,00			-0,26	0,02		GLUTATHIONE PEROXIDASE	I.B.6
RSc2675	RS00046			0,23	0,04			-0,43	0,01	1,69	0,00		SIGNAL PEPTIDE TYPE 4 FIMBRIAL BIOGENESIS PROTEIN PILY1-RELATED PRO	III.A.2
RSc2676	RS00045							-1,41	0,00				TRANSMEMBRANE	VI.C
RSc2677	RS00044	0,79	0,01					-0,93	0,02				TRANSMEMBRANE TYPE 4 FIMBRIAL BIOGENESIS PROTEIN PILW-RELATED PR	III.A.2
RSc2678	RS00043									-2,75	0,01		TRANSMEMBRANE TYPE 4 FIMBRIAL BIOGENESIS PILV-RELATED PROTEIN	III.A.2
RSc2679	RS00042	0,66	0,02					-0,59	0,04	-1,46	0,00		TRANSMEMBRANE TYPE 4 FIMBRIAL PILIN RELATED PROTEIN	III.A.2
RSc2680	RS00041							-1,38	0,00	-2,22	0,00	pilE2	TRANSMEMBRANE TYPE 4 FIMBRIAL BIOGENESIS PROTEIN	III.A.2
RSc2681	RS00040					-0,34	0,00			-0,68	0,00	pilU	TWITCHING MOBILITY PROTEIN TRANSPORT FIMBRIA	III.A.2
RSc2682	RS00039					-0,84	0,00					pilT	TWITCHING MOBILITY PROTEIN TRANSPORT FIMBRIA	III.A.2
RSc2683	RS00038	0,30	0,03			-0,43	0,02			-0,23	0,01		CONSERVED HYPOTHETICAL PROTEIN	VI.C
RSc2684	RS00037			0,88	0,01							proC	SIGNAL PEPTIDE OXIDOREDUCTASE PYRROLINE-5-CARBOXYLATE REDUCTAS	I.A.16
RSc2685	RS00036					-0,69	0,00	-1,88	0,01				HYPOTHETICAL PROTEIN	VI.D
RSc2686	RS00035			0,47	0,00							ubiA	TRANSMEMBRANE 4-HYDROXYBENZOATE OCTAPRENYLTRANSFERASE	I.B.5
RSc2687	RS00034									-0,93	0,03	dps	TRANSCRIPTION REGULATOR DNA PROTECTION DURING STARVATION OR OX	I.E.1
RSc2688	RS00033					-1,19	0,00			2,82	0,00		TRANSMEMBRANE	VI.D
RSc2689	RS00032					-2,47	0,00			2,41	0,00		HYPOTHETICAL PROTEIN	VI.D
RSc2690	RS00030					-0,82	0,00					oxyR	TRANSCRIPTION REGULATOR HYDROGEN PEROXIDE-INDUCIBLE GENES ACTI	I.E.1
RSc2691	RS00029					0,70	0,00						HYPOTHETICAL PROTEIN	VI.D
RSc2692	RS00028												SIGNAL PEPTIDE	VI.C
RSc2693	RS04379												TRANSMEMBRANE OUTER MEMBRANE USHER PROTEIN	III.B.2
RSc2694	RS00027							0,35	0,01				TRANSMEMBRANE PILI ASSEMBLY CHAPERONE	IV.B
RSc2695	RS00024												SIGNAL PEPTIDE	VI.C
RSc2696	RS00023												SIGNAL PEPTIDE	VI.C
RSc2697	RS00022												COG1305, Transglutaminase-like enzyme, putative cysteine protease	I
RSc2698	RS00021					-0,84	0,00						CONSERVED HYPOTHETICAL PROTEIN	VI.C
RSc2699	RS00020					-1,03	0,00	-0,84	0,00				CONSERVED HYPOTHETICAL PROTEIN	VI.C
RSc2700	RS04382					-0,60	0,00						CONSERVED HYPOTHETICAL PROTEIN	VI.C
RSc2701	RS00019					-0,68	0,00	-1,41	0,02				HYPOTHETICAL PROTEIN	VI.D
RSc2702	RS04381											cspA	TRANSCRIPTION REGULATOR COLD SHOCK-LIKE PROTEIN	VI.A
RSc2703	RS00018			0,99	0,00	-0,69	0,01						HYPOTHETICAL PROTEIN	VI.B
RSc2704	RS04380			0,81	0,02	-0,65	0,01						HYPOTHETICAL PROTEIN	VI.D
RSc2705	RS00017					-0,26	0,01			0,73	0,04		HYPOTHETICAL PROTEIN	VI.D
RSc2706	RS00016					-0,30	0,00	-0,50	0,02			tISRso12	ISRso12-TRANSPOSASE ORFB	V.A
RSc2707	RS00015							-0,59	0,00			tISRso12	ISRso12-TRANSPOSASE ORFA	V.A
RSc2708	RS00014							-1,19	0,05				TRANSCRIPTION REGULATOR	VI.A
RSc2709	RS00013												COG3616, Amino acid aldolase or racemase	I
RSc2710	RS00012												COG4520, Surface antigen	III.A.2
RSc2711	RS00082			0,85	0,02	-0,23	0,01			-1,37	0,00	recG	ATP-DEPENDENT DNA HELICASE	II.B.8
RSc2712	RS00010											tsaA	S-ADENOSYLMETHIONINE:TRNA RIBOSYLTRANSFERASE-ISOMERASE	II.B.6
RSc2713	RS00008			0,72	0,05							tgt	QUEUINE TRNA-RIBOSYLTRANSFERASE	II.D.1
RSc2714	RS00007											yajC	TRANSMEMBRANE COG1862, Preprotein translocase subunit YajC	IV.E.2
RSc2715	RS00081									-0,46	0,00	secD	TRANSMEMBRANE PROTEIN-EXPORT MEMBRANE PROTEIN SECD	IV.E.2
RSc2716	RS00006									-0,32	0,02	secF	TRANSMEMBRANE PROTEIN-EXPORT MEMBRANE	IV.E.2
RSc2717	RS00005					-0,57	0,00			0,57	0,02		SIGNAL PEPTIDE	VI.C
RSc2718	RS00003					-0,86	0,00						SIGNAL PEPTIDE	VI.C
RSc2719	RS00083					-0,83	0,03	-1,04	0,01				TRANSMEMBRANE CYTOCHROME B-561	I.G.1
RSc2720	RS00002											purB	ADENYLOSUCCINATE LYASE	I.D.2
RSc2721	RS00001	-0,34	0,03	0,39	0,02	-0,60	0,00	-0,47	0,03			gstK	GLUTATHIONE S-TRANSFERASE	II.B.7
RSc2722	RS00132					-0,45	0,00						SIGNAL PEPTIDE COG0069, Glutamate synthase domain 2	I.A.19
RSc2723	RS00131			0,38	0,02	-0,48	0,00					trmU	TRNA 5-METHYLAMINOMETHYL-2-THIOURIDYLATE-METHYLTRANSFERASE	II.B.6
RSc2724	RS00130					-0,52	0,00	-0,67	0,00				CONSERVED HYPOTHETICAL PROTEIN	VI.C
RSc2725	RS00129							-0,51	0,03				TRANSCRIPTION REGULATOR	VI.A
RSc2726	RS00128			0,46	0,01								TRANSMEMBRANE MULTIDRUG RESISTANCE TRANSPORT PROTEIN	IV.D
RSc2727	RS00127												TRANSMEMBRANE MULTIDRUG RESISTANCE PROTEIN	IV.G.2
RSc2728	RS00126			1,14	0,03	-1,59	0,00						TRANSMEMBRANE CONSERVED HYPOTHETICAL PROTEIN	VI.C
RSc2729	RS00125												FERRICHROME-IRON RECEPTOR	IV.D.5

RSc2730	RS00124			0,26	0,02	-0,55	0,00	-1,11	0,01			pntAa	OXIDOREDUCTASE NAD(P) TRANSHYDROGENASE (ALPHA SUBUNIT PART 1)	I.F.18
RSc2731	RS00123							-1,30	0,00	-2,32	0,00	panD	ASPARTATE 1-DECARBOXYLASE PRECURSOR	I.B.11
RSc2732	RS00122	0,93	0,03	0,80	0,00							pntAb	TRANSMEMBRANE NAD(P) TRANSHYDROGENASE (ALPHA SUBUNIT PART 2)	I.F.18
RSc2733	RS00121					-0,39	0,03					pntB	OXIDOREDUCTASE TRANSMEMBRANE NADP TRANSHYDROGENASE (BETA SU	I.F.18
RSc2734	RS00120					-0,38	0,02						TRANSCRIPTION REGULATOR RESPONSE REGULATOR	VI.A
RSc2735	RS00119					-0,37	0,01					phcB	REGULATORY PROTEIN	I.E.1
RSc2736	RS00118			0,63	0,02					-0,74	0,02	phcS	TRANSCRIPTION REGULATOR TRANSMEMBRANE SENSOR HISTIDINE KINASE	I.E.1
RSc2737	RS00117					-0,38	0,00			-0,61	0,02	phcR	TRANSCRIPTION REGULATOR TWO COMPONENT RESPONSE REGULATOR	I.E.1
RSc2738	RS00116	-0,24	0,03			-0,41	0,01			-0,53	0,00		TRANSCRIPTION REGULATOR RESPONSE REGULATOR	VI.A
RSc2739	RS00115			0,57	0,03			-0,93	0,03				COG0116, N6-adenine-specific DNA methylase	II.B.8
RSc2740	RS00114												TRANSMEMBRANE	VI.C
RSc2741	RS00113			0,61	0,01					-0,68	0,00	proA	OXIDOREDUCTASE GAMMA-GLUTAMYL PHOSPHATE REDUCTASE (GPR)(GLUT	I.A.16
RSc2742	RS00112											holA	DNA POLYMERASE III (DELTA SUBUNIT)	II.B.8
RSc2743	RS00111											rlpB	LIPOPROTEIN LIPOPROTEIN B PRECURSOR	II.B.4
RSc2744	RS00110			0,75	0,05	-0,25	0,05	0,63	0,03	-0,87	0,01	leuS	LEUCYL-TRNA SYNTHETASE	II.D.1
RSc2745	RS00109									-0,45	0,00	dapB	OXIDOREDUCTASE DIHYDRODIPICOLINATE REDUCTASE	I.A.13
RSc2746	RS00108									-0,37	0,01		TRANSMEMBRANE COG2913, Small protein A (tmRNA-binding) - related protein	II.D.1
RSc2747	RS00107					-0,57	0,00					fur1	TRANSCRIPTION REGULATOR FERRIC UPTAKE TRANSCRIPTIONAL REPRESS	I.E.1
RSc2748	RS00106			0,29	0,01			0,81	0,01			phcA	TRANSCRIPTION REGULATOR	I.E.1
RSc2749	RS00105											gapA	OXIDOREDUCTASE GLYCERALDEHYDE 3-PHOSPHATE DEHYDROGENASE	I.H.8
RSc2750	RS00104					-0,25	0,02			-0,52	0,01	tktA	TRANSKETOLASE	I.F.19
RSc2751	RS00103											dgoAb	GALACTONATE DEHYDRATASE	I.I.4
RSc2752	RS00102											dgoAa	2-DEHYDRO-3-DEOXYPHOSPHOGALACTONATE ALDOLASE	I.I.4
RSc2753	RS00101												OXIDOREDUCTASE COG1028, Dehydrogenases with different specificities (related tc	I
RSc2754	RS00100					-0,45	0,01						COG2017, Galactose mutarotase and related enzymes	I
RSc2755	RS00099			-1,51	0,00	1,53	0,00	2,00	0,00	-1,98	0,03		COG3386, Gluconolactonase	I
RSc2756	RS00098					-0,45	0,03					araH	ABC TRANSPORTER L-ARABINOSE TRANSMEMBRANE PROTEIN	IV.D.3
RSc2757	RS00097			-1,00	0,01	1,48	0,00	1,29	0,00			araG	ABC TRANSPORTER L-ARABINOSE ATP-BINDING PROTEIN	IV.D.3
RSc2758	RS00096							-0,46	0,05			araF	ABC TRANSPORTER L-ARABINOSE-BINDING PERIPLASMIC PROTEIN (PBP)	IV.D.3
RSc2759	RS00095					-0,32	0,02					dgoK	2-DEHYDRO-3-DEOXYGALACTONOKINASE	I.I.4
RSc2760	RS00094					-0,36	0,02						SIGNAL PEPTIDE COG3203, Outer membrane protein (porin)	III.B.2
RSc2761	RS00093	-1,24	0,04							-0,96	0,01		TRANSCRIPTION REGULATOR	VI.A
RSc2762	RS00092			0,50	0,00	-0,65	0,00						CONSERVED HYPOTHETICAL PROTEIN	VI.C
RSc2763	RS00091					-0,42	0,01						CONSERVED HYPOTHETICAL PROTEIN	VI.C
RSc2764	RS00090			0,50	0,01					-0,38	0,01		CONSERVED HYPOTHETICAL PROTEIN	VI.C
RSc2765	RS00089					-0,53	0,00						CONSERVED HYPOTHETICAL PROTEIN	VI.C
RSc2766	RS00088					-0,67	0,00	-0,78	0,01				SIGNAL PEPTIDE GUANYL-SPECIFIC RIBONUCLEASE PRECURSOR	II.A.2
RSc2767	RS00080			0,44	0,01	-0,55	0,00			-0,67	0,01	maeB2	OXIDOREDUCTASE NADP-DEPENDENT MALIC ENZYME	I.F.18
RSc2768	RS00079											thiL	THIAMINE-MONOPHOSPHATE KINASE	I.B.8
RSc2769	RS00078			0,32	0,04							pgpA	TRANSMEMBRANE PHOSPHATIDYL-GLYCEROPHOSPHATASE HYDROLASE	II.B.1
RSc2770	RS00077												CONSERVED HYPOTHETICAL PROTEIN	VI.C
RSc2771	RS00076												CONSERVED HYPOTHETICAL PROTEIN	VI.C
RSc2772	RS00075												TRANSMEMBRANE METAL TRANSPORT PROTEIN	IV.D
RSc2773	RS00074											pyrF	OROTIDINE 5'-PHOSPHATE DECARBOXYLASE	I.D.1
RSc2774	RS00073											corA	TRANSMEMBRANE MAGNESIUM AND COBALT TRANSPORT PROTEIN	IV.D.7
RSc2775	RS00072			-7,03	0,00	8,41	0,00	3,49	0,00			popW	HARPIN-RELATED PROTEIN	VI.C
RSc2776	RS00071											mtgA	TRANSMEMBRANE MONOFUNCTIONAL BIOSYNTHETIC PEPTIDOGLYCAN TRAN	III.B.3
RSc2777	RS00070											aroE1	OXIDOREDUCTASE SHIKIMATE 5-DEHYDROGENASE	I.A.5
RSc2778	RS00069	-0,18	0,05			-0,63	0,00			-0,44	0,01		TRANSMEMBRANE COG0810, Periplasmic TonB - related protein	III.B.1
RSc2779	RS00068			1,58	0,04								RIBONUCLEASE	II.A.2
RSc2780	RS00067							0,40	0,04				CONSERVED HYPOTHETICAL PROTEIN	VI.C
RSc2781	RS00066					-0,13	0,04	0,38	0,03				COG3150, Esterase	I
RSc2782	RS00065			-1,31	0,00	1,45	0,00	0,74	0,03			mpl	TRANSMEMBRANE UDP-N-ACETYLMURAMATE:L-ALANYL-GAMMA-D-GLUTAMYI	III.B.3
RSc2783	RS00064					-0,43	0,00			0,40	0,04		CONSERVED HYPOTHETICAL PROTEIN	VI.C
RSc2784	RS00063												TRANSMEMBRANE COG0526, Thiol-disulfide isomerase and thioredoxins	II.B.7
RSc2785	RS00062			0,85	0,01							aroQ1	3-DEHYDROQUINATE DEHYDRATASE (3-DEHYDROQUINASE) (TYPE II DHQASE	I.A.5
RSc2786	RS00061											accB1	BIOTIN CARBOXYL CARRIER PROTEIN OF ACETYL-COA CARBOXYLASE (BCCF	I.B.17
RSc2787	RS00060			0,99	0,00	-0,23	0,01	0,88	0,02			accC1	BIOTIN CARBOXYLASE	I.B.1
RSc2788	RS00059			0,63	0,05							prmA	RIBOSOMAL PROTEIN L11 METHYLTRANSFERASE	III.C.2
RSc2789	RS00058			0,34	0,04	-0,73	0,04						TRANSMEMBRANE	VI.C
RSc2790	RS00057			0,74	0,00	-0,62	0,01					tpx	OXIDOREDUCTASE THIOL PEROXIDASE - SCAVENGASE P20	IV.G.4
RSc2791	RS00056			0,36	0,04	-0,69	0,00			-0,82	0,00		COG0524, Sugar kinase	I
RSc2792	RS00055					-0,74	0,00						TRANSMEMBRANE OUTER MEMBRANE LIPOPROTEIN	III.B.2
RSc2793	RS00054					-0,37	0,04	0,66	0,00				HISTONE H1	II.C
RSc2794	RS06021												HYPOTHETICAL PROTEIN	VI.D
RSc2795	RS00053												HYPOTHETICAL PROTEIN	VI.D
RSc2796	RS00052					1,34	0,00	1,91	0,00				TRANSMEMBRANE	VI.C
RSc2797	RS05620					0,34	0,05						TRANSMEMBRANE	VI.C
RSc2798	RS05621					-0,42	0,02						SIGNAL PEPTIDE	VI.C
RSc2799	RS05622					-0,47	0,00						TRANSMEMBRANE METHYL-ACCEPTING CHEMOTAXIS PROTEIN	IV.C
RSc2800	RS00305			0,33	0,01								HYPOTHETICAL PROTEIN	VI.D
RSc2801	RS00304			1,19	0,01								TRANSCRIPTION REGULATOR	VI.A
RSc2802	RS00303			-1,21	0,00	1,54	0,00			0,61	0,02		TRANSMEMBRANE COG5006, Permease	IV.D
RSc2803	RS00302												CONSERVED HYPOTHETICAL PROTEIN	VI.C
RSc2804	RS00301											nrdB	OXIDOREDUCTASE TRANSMEMBRANE RIBONUCLEOSIDE REDUCTASE (SMALL	I.F.2
RSc2805	RS00300					-0,37	0,00			-0,69	0,00	nrdA	OXIDOREDUCTASE RIBONUCLEOSIDE REDUCTASE 1 (LARGE CHAIN)	I.F.2
RSc2806	RS00299			0,45	0,02	-0,43	0,04	-0,81	0,03			ampD	AMIDASE	IV.G.4
RSc2807	RS00298											pehR	TRANSCRIPTION REGULATOR RESPONSE REGULATOR	I.E.1
RSc2808	RS00297			-0,45	0,00	0,57	0,01					pehS	TRANSCRIPTION REGULATOR TRANSMEMBRANE SENSOR HISTIDINE KINASE	I.E.1
RSc2809	RS00296							-0,66	0,03				TRANSMEMBRANE	VI.C
RSc2810	RS00295					-0,55	0,01						ABC TRANSPORTER COG4137, ABC-type uncharacterized transport system, permea	IV.D
RSc2811	RS00294			0,46	0,02					-0,91	0,00	ffh	SIGNAL RECOGNITION PARTICLE PROTEIN (FIFTY-FOUR HOMOLOG) (P48).	IV.E.2
RSc2812	RS00293			0,72	0,02			-0,58	0,05			hprT	TRANSMEMBRANE HYPOXANTHINE PHOSPHORIBOSYLTRANSFERASE	I.F.1
RSc2813	RS00292												TRANSMEMBRANE COG2095, Multiple antibiotic transporter	IV.D
RSc2814	RS00291					-0,43	0,01			-0,50	0,00		CONSERVED HYPOTHETICAL PROTEIN	VI.C
RSc2815	RS00290			0,62	0,04	-0,38	0,00						SIGNAL PEPTIDE TRANSGLYCOSYLASE	III.B
RSc2816	RS00289			0,62	0,00							proS	PROLYL-TRNA SYNTHETASE	II.B.7
RSc2817	RS00288			0,45	0,03	-0,37	0,04						COG0494, NTP pyrophosphohydrolase	VI.C
RSc2818	RS00287			0,44	0,05	-0,67	0,05			-1,39	0,05		LIPOPROTEIN	VI.B
RSc2819	RS00286	-0,39	0,02	0,75	0,02	-0,28	0,03			-0,60	0,00	proB	GLUTAMATE 5-KINASE	I.A.16
RSc2820	RS00285					-0,42	0,00			-0,62	0,02		COG0536, GTPase	VI.C
RSc2821	RS00284					-0,34	0,01	-0,37	0,05	-1,03	0,00	rpmA	50S RIBOSOMAL SUBUNIT PROTEIN L27	III.C.2
RSc2822	RS00283									-1,02	0,00	rplU	50S RIBOSOMAL SUBUNIT PROTEIN L21	III.C.2
RSc2823	RS00282					-0,40	0,04					ispB	OCTAPRENYL-DIPHOSPHATE SYNTHASE (OCTAPRENYL PYROPHOSPHATE SY	I.B.5
RSc2824	RS00281					-0,33	0,00			-1,45	0,01		TRANSMEMBRANE COG4536, Mg2+ and Co2+ transporter	IV.D.4
RSc2825	RS00280			0,71	0,01							pilB	TYPE IV PILUS ASSEMBLY PROTEIN	III.A.2
RSc2826	RS00279			0,61	0,00	-0,28	0,01					pilC	TRANSMEMBRANE FIMBRIAL ASSEMBLY PROTEIN	III.A.2
RSc2827	RS00278			0,49	0,00							pilD	TRANSMEMBRANE TYPE 4 PREPILIN PEPTIDASE BIFUNCTIONNAL: LEADER PE	III.A.2
RSc2828	RS00277			1,19	0,00	-0,27	0,00						COG0237, Dephospho-CoA kinase	I
RSc2829	RS00276			0,50	0,01	-0,41	0,02						CONSERVED HYPOTHETICAL PROTEIN	VI.C
RSc2830	RS00275					-0,38	0,00						CONSERVED HYPOTHETICAL PROTEIN	VI.C
RSc2831	RS00274			0,44	0,01			0,52	0,01			mutT	MUTATOR MUTT PROTEIN (7,8-DIHYDRO-8-OXOGUANINE-TRIPHOSPHATASE)	I.F.20
RSc2832	RS00273									-0,75	0,02		ATPase	VI.C
RSc2833	RS00272			0,71	0,00					-0,51	0,01	argJ	ARGININE BIOSYNTHESIS BIFUNCTIONAL PROTEIN: GLUTAMATE N-ACETYLTR	I.A.17
RSc2834	RS00271			0,46	0,01	-0,31	0,01					secA	PREPROTEIN TRANSLOCASE SECA SUBUNIT	IV.E.2
RSc2835	RS00270			0,67	0,01								TRANSMEMBRANE COG0739, Membrane protein related to metalloendopeptidases	VI.C
RSc2836	RS00269												CONSERVED HYPOTHETICAL PROTEIN	VI.C
RSc2837	RS00268					-0,54	0,00					lpxC	UDP-3-O-3-HYDROXYMYRISTOYL N-ACETYLGLUCOSAMINE DEACETYLASE	II.B.2
RSc2838	RS00267					-0,47	0,00						COG0678, Peroxiredoxin	II.B.7
RSc2839	RS00266					-0,20	0,00	1,01	0,00			ftsZ	TRANSMEMBRANE CELL DIVISION PROTEIN FTSZ	IV.A
RSc2840	RS00265											ftsA	CELL DIVISION PROTEIN FTSA	IV.A
RSc2841	RS00264					-0,58	0,00			-1,64	0,02	ftsQ	TRANSMEMBRANE CELL DIVISION PROTEIN	IV.A

RSc2842	RS00263			0,43	0,03	-0,52	0,00			-0,70	0,00	ddlB	D-ALANINE--D-ALANINE LIGASE B	III.B.3
RSc2843	RS00262											murC	UDP-N-ACETYLMURAMATE--ALANINE LIGASE	III.B.3
RSc2844	RS00261			0,32	0,05							murG	UDP-N-ACETYLGLUCOSAMINE--N-ACETYLMURAMYL-PENTAPEPTIDE PYROPH	III.B.3
RSc2845	RS00260					-0,47	0,00			-0,68	0,03	ftsW	TRANSMEMBRANE CELL DIVISION PROTEIN FTSW	IV.A
RSc2846	RS00259									-1,34	0,00	murD	UDP-N-ACETYLMURAMOYLALANINE--D-GLUTAMATE LIGASE	III.B.3
RSc2847	RS00258			0,55	0,00	-0,35	0,00			-1,21	0,00	mraY	TRANSMEMBRANE HOSPHO-N-ACETYLMURAMOYL-PENTAPEPTIDE-TRANSFEF	III.B.3
RSc2848	RS00257											murF	UDP-N-ACETYLMURAMOYLALANYL-D-GLUTAMYL-2, 6-DIAMINOPIMELATE--D-AI	III.B.3
RSc2849	RS00256											murE	UDP-N-ACETYLMURAMOYLALANYL-D-GLUTAMATE--2, 6-DIAMINOPIMELATE LI(	III.B.3
RSc2850	RS00255			0,48	0,04	-0,41	0,02			-1,21	0,00	ftsI	TRANSMEMBRANE PENICILLIN-BINDING PROTEIN 3 PRECURSOR PBP-3	IV.A
RSc2851	RS00254			0,34	0,04	-0,43	0,01			-0,75	0,03	ftsL	TRANSMEMBRANE CELL DIVISION PROTEIN FTSL	IV.A
RSc2852	RS00253											mraW	COG0275, S-adenosylmethionine-dependent methyltransferase involved in cell envel	III.B
RSc2853	RS00252					-0,77	0,00						CONSERVED HYPOTHETICAL PROTEIN	VI.C
RSc2854	RS00251					-0,46	0,01						COG2941, Ubiquinone biosynthesis protein	I.B.5
RSc2855	RS00250					1,26	0,00	1,80	0,00	0,60	0,05		SIGNAL PEPTIDE OUTER MEMBRANE PORIN PROTEIN	IV.D
RSc2856	RS00249					0,38	0,02	0,88	0,02				TRANSMEMBRANE METABOLITE TRANSPORT PROTEIN	IV.D
RSc2857	RS00248			0,96	0,01	-0,31	0,03					fadD1	LONG-CHAIN-FATTY-ACID--COA LIGASE	I.I.1
RSc2858	RS00247			1,78	0,00	-1,63	0,00			2,80	0,00		ZINC PROTEASE	II.A.4
RSc2859	RS00246			0,66	0,02								OXIDOREDUCTASE COG0243, Anaerobic dehydrogenase, typically selenocysteine-c	I
RSc2860	RS00245					-0,36	0,02					fruR	TRANSCRIPTION REGULATOR TRANSCRIPTION REGULATION REPRESSOR	I.H.8
RSc2861	RS00244							-2,28	0,02			fruB	MULTIPHOSPHORYL TRANSFER PROTEIN MTP	I.H.8
RSc2862	RS00243											fruK	1-PHOSPHOFRUCTOKINASE	I.H.8
RSc2863	RS00242											fruA	TRANSMEMBRANE PTS SYSTEM, FRUCTOSE-SPECIFIC IIBC COMPONENT (EIIE	IV.D.3
RSc2864	RS00241			0,60	0,01	-0,52	0,00	0,57	0,04				ABC TRANSPORTER ABC-type branched-chain amino acid transport systems, peripl	IV.D.2
RSc2865	RS00240												HYPOTHETICAL PROTEIN	VI.D
RSc2866	RS00239					-0,36	0,00					dppF1	ABC TRANSPORTER OLIGOPEPTIDE ATP-BINDING PROTEIN	IV.D.7
RSc2867	RS00238			0,53	0,03	-0,38	0,02	0,52	0,02			dppD1	ABC TRANSPORTER OLIGOPEPTIDE ATP-BINDING PROTEIN	IV.D.7
RSc2868	RS00237					-0,23	0,03					dppC1	TRANSMEMBRANE DIPEPTIDE TRANSPORT SYSTEM PERMEASE PROTEIN	IV.D.7
RSc2869	RS00234											dppB1	ABC TRANSPORTER TRANSMEMBRANE PROTEIN (DIPEPTIDE TRANSPORT SY	IV.D.7
RSc2870	RS00233					-0,52	0,00					dppA1	PERIPLASMIC DIPEPTIDE TRANSPORT PROTEIN	IV.D.7
RSc2871	RS00232											hipO	HIPPURATE HYDROLASE	I.I
RSc2872	RS00230											paaF	ENOYL-COA HYDRATASE	I.I.4
RSc2873	RS00229					-0,59	0,00					paaG1	ENOYL-COA HYDRATASE	I.I.4
RSc2874	RS00228					-0,31	0,03	-0,70	0,01			paaI	PHENYLACETIC ACID DEGRADATION PROTEIN	I.I.4
RSc2875	RS00227			0,53	0,02	-0,36	0,00					paaK	PHENYLACETYL-CoA-LIGASE	I.I.4
RSc2876	RS00226			0,51	0,04								TRANSMEMBRANE MEMBRANE-BOUND LYTIC MUREIN TRANSGLYCOSYLASE /	III.B.3
RSc2877	RS00225												TRANSMEMBRANE	VI.C
RSc2878	RS00224							0,59	0,04			apaG	CONSERVED HYPOTHETICAL PROTEIN	VI.C
RSc2879	RS00223			0,57	0,01	-0,21	0,02					rpe	RIBULOSE-PHOSPHATE 3-EPIMERASE	I.F.19
RSc2880	RS00222					-0,47	0,01					gph	PHOSPHOGLYCOLATE PHOSPHATAS	I.I.4
RSc2881	RS00221					0,86	0,00	0,69	0,01			trpE	ANTHRANILATE SYNTHASE COMPONENT I	I.A.6
RSc2882	RS00220					1,19	0,00	0,96	0,01			trpG	PANTHRANILATE SYNTHASE COMPONENT II (GLUTAMINE AMIDO-TRANSFERA	I.A.6
RSc2883	RS00219			-0,78	0,00	1,17	0,00	0,96	0,00				SIGNAL PEPTIDE	VI.C
RSc2884	RS00218					1,13	0,00					trpD1	ANTHRANILATE PHOSPHORIBOSYLTRANSFERASE	I.A.6
RSc2885	RS00217					1,05	0,00	2,84	0,00			trpC1	INDOLE-3-GLYCEROL PHOSPHATE SYNTHASE	I.A.6
RSc2886	RS00215	-1,09	0,05			0,98	0,00	1,04	0,01				CONSERVED HYPOTHETICAL PROTEIN	VI.C
RSc2887	RS00213					0,31	0,03	0,85	0,00			ung	URACIL-DNA GLYCOSYLASE	II.B.8
RSc2888	RS00212							-1,11	0,00	-0,37	0,01		HYPOTHETICAL PROTEIN	VI.B
RSc2889	RS00211							-0,65	0,05				TRANSMEMBRANE	VI.C
RSc2890	RS00209			0,64	0,00	-0,48	0,00						COG3975, Predicted protease with the C-terminal PDZ domain	II.A.4
RSc2891	RS00208			1,14	0,02								OXIDOREDUCTASE COG0604, NADPH:quinone reductase and related Zn-dependen	I
RSc2892	RS00206			0,26	0,04	-0,55	0,00			-0,44	0,00	dsbC	THIOL:DISULFIDE INTERCHANGE PROTEIN	II.B.7
RSc2893	RS00205			0,68	0,02			0,89	0,02				OXIDOREDUCTASE	I
RSc2894	RS00204			-0,62	0,05								TRANSMEMBRANE	VI.D
RSc2895	RS00203			-0,31	0,05	0,47	0,02						SIGNAL PEPTIDE	VI.D
RSc2896	RS00201												CONSERVED HYPOTHETICAL PROTEIN	VI.C
RSc2897	RS00200	-3,14	0,01	-5,22	0,00	4,49	0,00	3,50	0,00				CONSERVED HYPOTHETICAL PROTEIN	VI.C
RSc2898	RS00199			0,64	0,02					-0,27	0,00		COG0012, GTPase, probable translation factor	II.B.7
RSc2899	RS00198					1,01	0,00	1,50	0,00				TRANSMEMBRANE	VI.C
RSc2900	RS00197											hemA	OXIDOREDUCTASE GLUTAMYL-TRNA REDUCTASE	I.B.4
RSc2901	RS00196			0,79	0,02	-0,45	0,02					prfA	PEPTIDE CHAIN RELEASE FACTOR 1 RF-1	II.B.7
RSc2902	RS00193					-0,54	0,01			0,65	0,01		LIPOPROTEIN	VI.C
RSc2903	RS00311			0,87	0,01	-0,54	0,00					hemK	Methylase of polypeptide chain release factors	II.B.7
RSc2904	RS00191			0,56	0,01			0,53	0,05				CONSERVED HYPOTHETICAL PROTEIN	VI.C
RSc2905	RS00189			0,64	0,04							ubiX	3-OCTAPRENYL-4-HYDROXYBENZOATE CARBOXY-LYASE	I
RSc2906	RS00188					-0,89	0,00	0,99	0,00	2,10	0,00		TRANSMEMBRANE	VI.D
RSc2907	RS00187					-0,53	0,01						SIGNAL PEPTIDE	VI.C
RSc2908	RS00186					-0,53	0,01					mdoG	SIGNAL PEPTIDE PERIPLASMIC GLUCANS BIOSYNTHESIS	IV.F.2
RSc2909	RS00341					1,13	0,00					mdoH	TRANSMEMBRANE PERIPLASMIC GLUCANS BIOSYNTHESIS PROTEIN	IV.F.2
RSc2910	RS00185					-0,78	0,00	0,49	0,02	2,50	0,00		SIGNAL PEPTIDE	VI.C
RSc2911	RS00184					-0,58	0,00	0,80	0,01				CONSERVED HYPOTHETICAL PROTEIN	VI.C
RSc2912	RS00183					-0,58	0,01						CONSERVED HYPOTHETICAL PROTEIN	VI.C
RSc2913	RS00182			0,57	0,04	-0,51	0,00						ABC TRANSPORTER ATP-BINDING PROTEIN	IV.D
RSc2914	RS00181			0,77	0,00	-0,83	0,00					cyoD1	OXIDOREDUCTASE TRANSMEMBRANE CYTOCHROME O UBIQUINOL OXIDASE	I.H.3
RSc2915	RS00180			0,91	0,00	-0,80	0,00	0,72	0,00	0,68	0,01	cyoC1	OXIDOREDUCTASE TRANSMEMBRANE CYTOCHROME O UBIQUINOL OXIDASE	I.H.3
RSc2916	RS00178			1,20	0,00	-0,71	0,00	2,11	0,00			cyoB1	OXIDOREDUCTASE TRANSMEMBRANE CYTOCHROME O UBIQUINOL OXIDASE	I.H.3
RSc2917	RS00308			0,84	0,02	-0,94	0,01					cyoA1	OXIDOREDUCTASE TRANSMEMBRANE CYTOCHROME O UBIQUINOL OXIDASE	I.H.3
RSc2918	RS00176					-1,38	0,00			-0,95	0,00		TRANSCRIPTION REGULATOR EXTRACYTOPLASMIC FUNCTION SIGMA FACTO	I.E.1
RSc2919	RS00175			0,61	0,01	-1,10	0,00	-1,08	0,01	-3,16	0,01		TRANSMEMBRANE IRON TRANSPORT - SENSORY TRANSDUCTION PROTEIN	IV.D.5
RSc2920	RS00173												SIGNAL PEPTIDE HYDROXAMATE-TYPE FERRISIDEROPHORE RECEPTOR	IV.D.5
RSc2921	RS00172			0,74	0,02	-0,92	0,01					ggt2	GAMMA-GLUTAMYLTRANSPEPTIDASE PRECURSOR	I.B.6
RSc2922	RS00171					-0,52	0,05						SIGNAL PEPTIDE OUTER MEMBRANE RECEPTOR	III.B.2
RSc2923	RS00170			1,08	0,00	-0,40	0,02						TRANSCRIPTION REGULATOR TRANSMEMBRANE SENSOR HISTIDINE KINASE	VI.A
RSc2924	RS00169												COG0456, Acetyltransferase	I
RSc2925	RS00167			0,88	0,00					-0,79	0,00	sspB	STRINGENT STARVATION PROTEIN B	I.E.1
RSc2926	RS00165			0,82	0,00							sspA	TRANSCRIPTION MODULATOR PROTEIN	I.E.1
RSc2927	RS00164			0,91	0,03							petC	TRANSMEMBRANE CYTOCHROME C1 PRECURSOR	I.G.1
RSc2928	RS00163											petB	TRANSMEMBRANE CYTOCHROME B SUBUNIT	I.G.1
RSc2929	RS00342											petA	OXIDOREDUCTASE TRANSMEMBRANE UBIQUINOL-CYTOCHROME C REDUCTA	I.G.1
RSc2930	RS00160					-0,60	0,00					mscL	TRANSMEMBRANE LARGE-CONDUCTANCE MECHANOSENSITIVE CHANNEL	IV.D
RSc2931	RS00159												CONSERVED HYPOTHETICAL PROTEIN	VI.C
RSc2932	RS00158			0,59	0,00								SIGNAL PEPTIDE HTRA-LIKE SERINE PROTEASE	II.A.4
RSc2933	RS00157					-0,44	0,01						SIGNAL PEPTIDE PORIN	IV.D
RSc2934	RS00156					-0,39	0,00						ABC TRANSPORTER COG1840, ABC-type Fe3+ transport system, periplasmic comp	IV.D.5
RSc2935	RS00154												TRANSCRIPTION REGULATOR RESPONSE REGULATOR	VI.A
RSc2936	RS00153												TRANSCRIPTION REGULATOR TRANSMEMBRANE SENSOR KINASE	VI.A
RSc2937	RS00344												SIGNAL PEPTIDE PERIPLASMIC IRON-BINDING PROTEIN	IV.D.5
RSc2938	RS00151			0,37	0,03								ABC TRANSPORTER TRANSMEMBRANE PROTEIN	IV.D
RSc2939	RS00150					-0,47	0,00						ABC TRANSPORTER ATP-BINDING PROTEIN	IV.D
RSc2940	RS00149			0,47	0,00							tatC	TRANSMEMBRANE SEC-INDEPENDENT PROTEIN TRANSLOCASE PROTEIN TAT	IV.E.2
RSc2941	RS00148					-0,57	0,00	-0,56	0,03			tatB	SEC-INDEPENDENT PROTEIN TRANSLOCASE PROTEIN TATB	IV.E.2
RSc2942	RS00147					0,38	0,00					tatA	SIGNAL PEPTIDE SEC-INDEPENDENT PROTEIN TRANSLOCASE PROTEIN TATA	IV.E.2
RSc2943	RS00144			0,40	0,04	-0,47	0,01			-0,25	0,05		TRANSMEMBRANE	VI.C
RSc2944	RS00143					-0,45	0,03			-0,88	0,01	hisE	PHOSPHORIBOSYL-ATP PYROPHOSPHATASE	I.A.4
RSc2945	RS00142			0,76	0,01					-0,77	0,00	hisI	PHOSPHORIBOSYL-AMP CYCLOHYDROLASE	I.A.4
RSc2946	RS00140			0,65	0,02			0,82	0,01	-0,66	0,00	hisF	CYCLASE (IMIDAZOLE GLYCEROL PHOSPHATE SYNTHASE - SUBUNIT WITH HI	I.A.4
RSc2947	RS00139			0,50	0,04							hisA	PHOSPHORIBOSYLFORMIMINO-5-AMINOIMIDAZOLE CARBOXAMIDE RIBOTIDE	I.A.4
RSc2948	RS00138					-0,53	0,03			0,99	0,03	hisH	AMIDOTRANSFERASE (GLUTAMINE AMIDOTRANSFERASE - SUBUNIT WITH HIS	I.A.4
RSc2949	RS00137												TRANSMEMBRANE COG2095, Multiple antibiotic transporter	IV.D
RSc2950	RS00136			0,38	0,01	-0,36	0,04					hisB	OXIDOREDUCTASE IMIDAZOLEGLYCEROL-PHOSPHATE DEHYDRATASE	I.A.4
RSc2951	RS00135					0,23	0,03					hisC1	HISTIDINOL-PHOSPHATE AMINOTRANSFERASE	I.A.4
RSc2952	RS00134			0,43	0,00	-0,28	0,05					hisD	OXIDOREDUCTASE HISTIDINOL DEHYDROGENASE	I.A.4
RSc2953	RS04753			0,95	0,00	-0,41	0,00	1,05	0,03	-0,33	0,04	murA	UDP-N-ACETYLGLUCOSAMINE 1-CARBOXYVINYLTRANSFERASE	III.B.3

RSc2954	RS01346			-0,35 0,02				TRANSCRIPTION REGULATOR	VI.A
RSc2955	RS01345		0,48 0,03			-2,36 0,02		ABC TRANSPORTER COG0842, ABC-type multidrug transport system, permease cor	IV.D
RSc2956	RS01344							ABC TRANSPORTER ATP-BINDING PROTEIN	IV.D
RSc2957	RS01343							SIGNAL PEPTIDE COG3113, NTP binding protein (contains STAS domain)	VI.C
RSc2958	RS01342					0,99 0,00		SIGNAL PEPTIDE	VI.C
RSc2959	RS01340			0,69 0,00	0,98 0,01			TRANSMEMBRANE COG2853, Surface lipoprotein	III.B.2
RSc2960	RS01338			0,76 0,01				SIGNAL PEPTIDE	VI.C
RSc2961	RS01337			0,63 0,00	0,62 0,01	-0,78 0,00		ABC TRANSPORTER COG0767, ABC-type transport system involved in resistance to	IV.D
RSc2962	RS01335		0,88 0,01	-0,46 0,02				ABC TRANSPORTER ATP-BINDING PROTEIN	IV.D
RSc2963	RS01334							CONSERVED HYPOTHETICAL PROTEIN	VI.C
RSc2964	RS01333						gltD	OXIDOREDUCTASE GLUTAMATE SYNTHASE (SMALL SUBUNIT)	I.A.19
RSc2965	RS01332						gltB	OXIDOREDUCTASE GLUTAMATE SYNTHASE (LARGE SUBUNIT)	I.A.19
RSc2966	RS01331			-0,35 0,02				COG1943, Transposase and inactivated derivatives	V.A
RSc2967	RS01330							SIGNAL PEPTIDE OUTER MEMBRANE PROTEIN W - RELATED PROTEIN	III.B.2
RSc2968	RS01329					-1,84 0,00	dgt	DEOXYGUANOSINE TRIPHOSPHATE TRIPHOSPHOHYDROLASE	I.F.12
RSc2969	RS01328		0,40 0,05	-0,42 0,00			aroB	3-DEHYDROQUINATE SYNTHASE	I.A.5
RSc2970	RS01327			-0,38 0,00	0,56 0,04		aroK	SHIKIMATE KINASE I	I.A.5
RSc2971	RS01326						pilQ	SIGNAL PEPTIDE FIMBRIAL TYPE-4 ASSEMBLY PROTEIN	III.A.2
RSc2972	RS01325				0,47 0,03		pilP	FIMBRIAL TYPE-4 ASSEMBLY LIPOPROTEIN	III.A.2
RSc2973	RS01324		0,30 0,01	-0,37 0,01		-0,58 0,01	pilO	TRANSMEMBRANE FIMBRIAL TYPE-4 ASSEMBLY MEMBRANE PROTEIN	III.A.2
RSc2974	RS01323		0,27 0,05			-0,99 0,01	pilN	TRANSMEMBRANE FIMBRIAL TYPE-4 ASSEMBLY MEMBRANE PROTEIN	III.A.2
RSc2975	RS01321			-0,31 0,03			pilM	FIMBRIAL TYPE-4 ASSEMBLY PROTEIN	III.A.2
RSc2976	RS01357		0,65 0,01				mrcA	TRANSMEMBRANE PENICILLIN-BINDING PROTEIN 1 (PEPTIDOGLYCAN SYNTHE	III.B.3
RSc2977	RS01320						cyaY	CYAY PROTEIN	IV.E.2
RSc2978	RS01319					-0,46 0,02		TRANSMEMBRANE PROLINE-RICH PROTEIN	VI.D
RSc2979	RS01358						lysA	DIAMINOPIMELATE DECARBOXYLASE	I.A.13
RSc2980	RS01266							TRANSMEMBRANE	VI.C
RSc2981	RS01260			-0,50 0,02	-0,65 0,04			OXIDOREDUCTASE COG2041, Sulfite oxidase and related enzymes	I
RSc2982	RS01253							TRANSMEMBRANE COG5662, Transmembrane transcriptional regulator (anti-sigma	VI.A
RSc2983	RS01249						rpoE2	TRANSCRIPTION REGULATOR RNA POLYMERASE SIGMA-E FACTOR (SIGMA-2	II.B.6
RSc2984	RS01242			-0,39 0,02				TRANSMEMBRANE	VI.C
RSc2985	RS01238		0,85 0,00					ABC TRANSPORTER COG0755, ABC-type transport system involved in cytochrome (	IV.D
RSc2986	RS01235		0,77 0,01					TRANSMEMBRANE CYTOCHROME C BIOGENESIS PROTEIN	VI.C
RSc2987	RS01225							SIGNAL PEPTIDE OXIDOREDUCTASE CYTOCHROME C-TYPE	I.G.1
RSc2988	RS01211							GTPase	VI.C
RSc2989	RS01152		0,46 0,01		-0,61 0,01		hemB	DELTA-AMINOLEVULINIC ACID DEHYDRATASE	I.B.4
RSc2990	RS01128		0,41 0,03		0,96 0,01		dsbD	TRANSMEMBRANE THIOL:DISULFIDE INTERCHANGE PROTEIN	I
RSc2991	RS01125			-0,44 0,00			cutA	PERIPLASMIC DIVALENT CATION TOLERANCE PROTEIN	II.B
RSc2992	RS01123		0,86 0,00			-1,46 0,00	rplQ	50S RIBOSOMAL SUBUNIT PROTEIN L17	III.C.2
RSc2993	RS01122		0,80 0,02	-0,39 0,01			rpoA	DNA-DIRECTED RNA POLYMERASE (ALPHA CHAIN)	II.B.6
RSc2994	RS01120				0,60 0,02		rpsD	30S RIBOSOMAL SUBUNIT PROTEIN S4	III.C.2
RSc2995	RS01118					-1,22 0,02	rpsK	30S RIBOSOMAL SUBUNIT PROTEIN S11	III.C.2
RSc2996	RS01356			-0,37 0,00		-0,90 0,01	rpsM	30S RIBOSOMAL SUBUNIT PROTEIN S13	III.C.2
RSc2997	RS01115			-0,26 0,02	0,61 0,03		rpmJ	50S RIBOSOMAL SUBUNIT PROTEIN L36	III.C.2
RSc2998	RS01113				0,86 0,02	-0,88 0,00	infA1	TRANSLATION INITIATION FACTOR IF-1	II.B.7
RSc2999	RS01096					-0,77 0,04	secY	TRANSMEMBRANE PREPROTEIN TRANSLOCASE SECY (MEMBRANE SUBUNIT)	IV.E.2
RSc3000	RS01090			-0,38 0,04			rplO	50S RIBOSOMAL SUBUNIT PROTEIN L15	III.C.2
RSc3001	RS01089		0,86 0,04	-0,32 0,02	0,87 0,00		rpmD	50S RIBOSOMAL SUBUNIT PROTEIN L30	III.C.2
RSc3002	RS01088		0,78 0,02		1,15 0,00		rpsE	30S RIBOSOMAL SUBUNIT PROTEIN S5	III.C.2
RSc3003	RS01087		0,89 0,02		0,98 0,00		rplR	50S RIBOSOMAL SUBUNIT PROTEIN L18	III.C.2
RSc3004	RS01086		0,84 0,00		0,77 0,02	-0,86 0,02	rplF	50S RIBOSOMAL SUBUNIT PROTEIN L6	III.C.2
RSc3005	RS01085		0,69 0,00			-0,88 0,00	rpsH	30S RIBOSOMAL SUBUNIT PROTEIN S8	III.C.2
RSc3006	RS01083		0,63 0,03		0,56 0,01		rpsN	30S RIBOSOMAL SUBUNIT PROTEIN S14	III.C.2
RSc3007	RS01082					-0,84 0,00	rplE	50S RIBOSOMAL SUBUNIT PROTEIN L5	III.C.2
RSc3008	RS01081				0,47 0,03		rplX	50S RIBOSOMAL SUBUNIT PROTEIN L24	III.C.2
RSc3009	RS01079					-0,63 0,00	rplN	50S RIBOSOMAL SUBUNIT PROTEIN L14	III.C.2
RSc3010	RS01078		0,82 0,01				rpsQ	30S RIBOSOMAL SUBUNIT PROTEIN S17	III.C.2
RSc3011	RS01354					-0,59 0,01	rpmC	50S RIBOSOMAL SUBUNIT PROTEIN L29	III.C.2
RSc3012	RS01077		1,03 0,03			-0,69 0,01	rplP	50S RIBOSOMAL SUBUNIT PROTEIN L16	III.C.2
RSc3013	RS01076		0,89 0,05	-0,24 0,02		-0,86 0,00	rpsC	30S RIBOSOMAL SUBUNIT PROTEIN S3	III.C.2
RSc3014	RS04743					-0,50 0,01	rplV	50S RIBOSOMAL SUBUNIT PROTEIN L22	III.C.2
RSc3015	RS04742				0,86 0,00		rpsS	30S RIBOSOMAL SUBUNIT PROTEIN S19	III.C.2
RSc3016	RS04741						rplB	50S RIBOSOMAL SUBUNIT PROTEIN L2	III.C.2
RSc3017	RS04740				0,93 0,02		rplW	50S RIBOSOMAL SUBUNIT PROTEIN L23	III.C.2
RSc3018	RS04739		1,04 0,01			-0,73 0,00	rplD	50S RIBOSOMAL PROTEIN L4 (rRNA-BINDING TRANSLATION REGULATION REF	III.C.2
RSc3019	RS04738			-0,25 0,02		-0,83 0,00	rplC	50S RIBOSOMAL SUBUNIT PROTEIN L3 (rRNA-BINDING METHYLATION)	III.C.2
RSc3020	RS04737		0,81 0,00		0,84 0,00		rpsJ	30S RIBOSOMAL SUBUNIT PROTEIN S10	III.C.2
RSc3022	RS04735						fusA1	ELONGATION FACTOR G (EF-G PROTEIN)	II.B.7
RSc3023	RS04734				2,42 0,03	-0,76 0,00	rpsG	30S RIBOSOMAL SUBUNIT PROTEIN S7	III.C.2
RSc3024	RS04733						rpsL	30S RIBOSOMAL SUBUNIT PROTEIN S12	III.C.2
RSc3025	RS04732		0,43 0,02				recQ	ATP-DEPENDENT DNA HELICASE	II.B.8
RSc3026	RS04731		0,44 0,04					CONSERVED HYPOTHETICAL PROTEIN	VI.C
RSc3027	RS04730		0,90 0,05	-0,56 0,00		-2,35 0,04		SIGNAL PEPTIDE	VI.C
RSc3028	RS04729	-0,19 0,04	0,30 0,02	-0,52 0,00				SIGNAL PEPTIDE	VI.C
RSc3029	RS04728							SIGNAL PEPTIDE	VI.C
RSc3030	RS04727	0,43 0,05						SIGNAL PEPTIDE	VI.C
RSc3031	RS04726		-0,91 0,01	0,60 0,03				CONSERVED HYPOTHETICAL PROTEIN	VI.C
RSc3032	RS04725							TRANSMEMBRANE	VI.C
RSc3033	RS04724		0,74 0,00	-0,19 0,01		-0,66 0,00	rpoC	DNA-DIRECTED RNA POLYMERASE (BETA' CHAIN)	II.B.6
RSc3034	RS04723		0,65 0,04				rpoB	DNA-DIRECTED RNA POLYMERASE (BETA CHAIN)	II.B.6
RSc3035	RS04722					-1,36 0,00	rplL	50S RIBOSOMAL SUBUNIT PROTEIN L7/L12 (ACETYLATION METHYLATION)(L8)	III.C.2
RSc3036	RS04721				0,66 0,03	-1,04 0,00	rplJ	50S RIBOSOMAL SUBUNIT PROTEIN L10 (TRANSLATION REGULATION REPRES	III.C.2
RSc3037	RS04720		0,77 0,02		0,73 0,01		rplA	50S RIBOSOMAL SUBUNIT PROTEIN L1 (rRNA-BINDING TRANSLATION REGULA	III.C.2
RSc3038	RS04747		0,50 0,00			-1,04 0,00	rplK	50S RIBOSOMAL SUBUNIT PROTEIN L11 (METHYLATION RNA-BINDING)	III.C.2
RSc3039	RS04718				0,57 0,03	-1,66 0,00	nusG	TRANSCRIPTION ANTITERMINATION PROTEIN	II.B.6
RSc3040	RS04717			-0,31 0,05	1,08 0,01	-0,70 0,02	secE	TRANSMEMBRANE PREPROTEIN TRANSLOCASE (SECE SUBUNIT)	IV.E.2
RSc3041	RS05596		0,67 0,01		0,89 0,00		tuf2	ELONGATION FACTOR TU (EF-TU PROTEIN)	II.B.7
RSc3042	RS05607							HYPOTHETICAL PROTEIN	VI.D
RSc3043	RS05600							CONSERVED HYPOTHETICAL PROTEIN	VI.C
RSc3044	RS00485			-0,33 0,01	-1,27 0,01		glpR	TRANSCRIPTION REGULATOR GLYCEROL-3-PHOSPHATE REGULON REPRESS	I.H
RSc3045	RS00486			-0,66 0,01			glpD	OXIDOREDUCTASE GLYCEROL-3-PHOSPHATE DEHYDROGENASE	I.H
RSc3046	RS00487			-0,36 0,00				ABC TRANSPORTER SUGAR-PHOSPHATE ATP-BINDING PROTEIN	IV.D.3
RSc3047	RS00488			-0,35 0,02	-1,40 0,01			ABC TRANSPORTER ATP-BINDING PROTEIN	IV.D
RSc3048	RS00494			-0,47 0,01				ABC TRANSPORTER SUGAR ABC TRANSPORTER, PERMEASE PROTEIN	IV.D.3
RSc3049	RS00489		1,60 0,02	-1,24 0,00	-2,41 0,00			ABC TRANSPORTER TRANSMEMBRANE PROTEIN	IV.D
RSc3050	RS00491			-0,80 0,00				TRANSMEMBRANE	VI.C
RSc3051	RS00492			-0,54 0,01				ABC TRANSPORTER COG1653, ABC-type sugar transport system, periplasmic comp	IV.D.3
RSc3052	RS00495						glpK	ATP:GLYCEROL 3-PHOSPHOTRANSFERASE	I.F.11
RSc3053	RS00496				0,71 0,05	1,02 0,02		SIGNAL PEPTIDE	VI.D
RSc3054	RS00497							CONSERVED HYPOTHETICAL PROTEIN	VI.C
RSc3055	RS00498					0,71 0,02		COG5573, Predicted nucleic-acid-binding protein, contains PIN domain	VI.C
RSc3056	RS00499			-0,34 0,00				PEMI-RELATED PROTEIN	VI.A
RSc3057	RS00501			-0,41 0,04				COG3131 Periplasmic glucans biosynthesis protein	IV.F.2
RSc3058	RS00502		1,01 0,03	-0,56 0,00	-1,14 0,05		manA	MANNOSE-6-PHOSPHATE ISOMERASE	I.I.4
RSc3059	RS00517		0,86 0,00					TRANSMEMBRANE SUGAR TRANSPORTER	IV.D
RSc3060	RS00504							TRANSMEMBRANE TRANSPORTER	IV.D
RSc3061	RS00505			-0,55 0,05		0,82 0,04		ABC TRANSPORTER SUBSTRATE-BINDING PERIPLASMIC PROTEIN (PBP)	IV.D
RSc3062	RS00506							TRANSCRIPTION REGULATOR	VI.A
RSc3063	RS00507			-0,56 0,01				TRANSCRIPTION REGULATOR TRANSMEMBRANE SENSOR HISTIDINE KINASE	VI.A
RSc3064	RS00508							TRANSCRIPTION REGULATOR RESPONSE REGULATOR	VI.A
RSc3065	RS00509				-0,50 0,02		ompV	TRANSMEMBRANE COG3713, Outer membrane protein V	III.B.2
RSc3066	RS00510							HYPOTHETICAL PROTEIN	VI.D

RSc3067	RS00511					-1,04	0,00			-2,05	0,00		TRANSMEMBRANE	VI.C
RSc3068	RS00512					-0,39	0,01						TRANSMEMBRANE COG0668, Small-conductance mechanosensitive channel	VI.C
RSc3069	RS00513					-0,70	0,00						TRANSMEMBRANE TRANSPORTER PROTEIN	IV.D
RSc3070	RS00514					-1,14	0,00	-0,77	0,04			gstL	GLUTATHIONE S-TRANSFERASE	II.B.7
RSc3071	RS00520							1,11	0,03	1,27	0,00		CONSERVED HYPOTHETICAL PROTEIN	VI.C
RSc3072	RS00521					-0,90	0,00			2,35	0,00		CONSERVED HYPOTHETICAL PROTEIN	VI.C
RSc3073	RS00522												HYPOTHETICAL PROTEIN	VI.D
RSc3074	RS00523												HYPOTHETICAL PROTEIN	VI.D
RSc3075	RS00524			0,79	0,01	-0,66	0,00						SIGNAL PEPTIDE	VI.D
RSc3076	RS00525	1,13	0,04			-0,96	0,00						HYPOTHETICAL PROTEIN	VI.D
RSc3077	RS00528												TRANSMEMBRANE COG0053, Co/Zn/Cd cation transporters	IV.D.4
RSc3078	RS00529					-0,99	0,01	-1,18	0,02				TRANSCRIPTION REGULATOR RESPONSE REGULATOR	VI.A
RSc3079	RS00530												TRANSCRIPTION REGULATOR COMPOSITE TWO COMPONENT REGULATORY F	VI.A
RSc3080	RS00531												TRANSCRIPTION REGULATOR	VI.A
RSc3081	RS00532					-0,32	0,03						LIPOPROTEIN	VI.D
RSc3082	RS00533												PSEUDOGENE (C-TERMINAL FRAGMENT)	VI.D
RSc3083	RS00535												SIGNAL PEPTIDE	VI.D
RSc3084	RS00536			0,70	0,01	-0,22	0,04			1,25	0,00		TRANSMEMBRANE	VI.C
RSc3085	RS00537					-0,35	0,02						CONSERVED HYPOTHETICAL PROTEIN	VI.C
RSc3086	RS00538					0,35	0,01					hpiS	SIGNAL PEPTIDE HIGH POTENTIAL IRON-SULFUR PROTEIN (HIPIP)	I.H
RSc3087	RS00539					-0,65	0,00					kdpE	TRANSCRIPTION REGULATOR RESPONSE REGULATOR	I.E.1
RSc3088	RS00540			0,54	0,02	0,19	0,04	0,45	0,01				SIGNAL PEPTIDE	VI.C
RSc3089	RS00541					-0,90	0,00	-0,89	0,00			tISRso11	ISRso11-TRANSPOSASE ORFB	V.A
RSc3090	RS00542					-0,57	0,00					tISRso11	ISRso11-Transposase ORFA	V.A
RSc3091	RS00544			0,55	0,00	-0,38	0,01	0,75	0,00	0,74	0,02		SIGNAL PEPTIDE OUTER MEMBRANE PORIN	III.B.2
RSc3092	RS00545					-2,61	0,00	-1,70	0,00	2,44	0,00		SIGNAL PEPTIDE	VI.C
RSc3093	RS00546							-1,15	0,00				TRANSMEMBRANE CARBOXYPEPTIDASE G2 PRECURSOR (FOLATE HYDROLAS	I.I
RSc3094	RS00547					-0,64	0,00						CONSERVED HYPOTHETICAL PROTEIN	VI.C
RSc3095	RS00548					-0,70	0,02						SERINE-RICH PROTEIN	VI.D
RSc3096	RS00549											kefC	TRANSMEMBRANE GLUTATHIONE-REGULATED POTASSIUM-EFFLUX SYSTEM	IV.D.4
RSc3097	RS00550												OXIDOREDUCTASE COG2249, NADPH-quinone reductase	I
RSc3098	RS00551					-0,80	0,00	-1,21	0,04				CONSERVED HYPOTHETICAL PROTEIN	VI.C
RSc3099	RS00552					0,44	0,01	2,25	0,00				SIGNAL PEPTIDE	VI.C
RSc3100	RS00553												COG4319, Ketosteroid isomerase homolog	VI.C
RSc3101	RS00554	-5,08	0,00	0,79	0,04	-1,47	0,00	1,71	0,00	3,43	0,00		SERINE PROTEASE	II.A.4
RSc3102	RS00555			1,07	0,05	-0,61	0,00	-1,04	0,04				TRANSCRIPTION REGULATOR	VI.A
RSc3103	RS00556									1,27	0,00		OXIDOREDUCTASE 4-HYDROXYPHENYLPYRUVATE DIOXYGENASE	I
RSc3104	RS00557					-0,91	0,00	0,55	0,03	1,84	0,00		SIGNAL PEPTIDE CALCIUM-BINDING PROTEIN	VI.C
RSc3105	RS00558			-0,45	0,02	-0,36	0,03					gspC	TRANSMEMBRANE GENERAL SECRETION PATHWAY RELATED PROTEIN	IV.E.2
RSc3106	RS00559							0,79	0,01			gspG	TRANSMEMBRANE GENERAL SECRETION PATHWAY PROTEIN G	IV.E.2
RSc3107	RS00560											gspH	TRANSMEMBRANE GENERAL SECRETORY PATHWAY PROTEIN H	IV.E.2
RSc3108	RS00561											gspI	TRANSMEMBRANE GENERAL SECRETORY PATHWAY PROTEIN I	IV.E.2
RSc3109	RS00562											gspJ	TRANSMEMBRANE GENERAL SECRETORY PATHWAY PROTEIN J	IV.E.2
RSc3110	RS00563			-1,15	0,00	1,41	0,00					gspK	TRANSMEMBRANE GENERAL SECRETORY PATHWAY PROTEIN K	IV.E.2
RSc3111	RS00564					-0,69	0,00	-0,33	0,02			gspL	TRANSMEMBRANE GENERAL SECRETORY PATHWAY PROTEIN L	IV.E.2
RSc3112	RS00565											gspM	TRANSMEMBRANE GENERAL SECRETORY PATHWAY PROTEIN M	IV.E.2
RSc3113	RS00566											gspN	TRANSMEMBRANE GENERAL SECRETORY PATHWAY PROTEIN N	IV.E.2
RSc3114	RS00567					0,94	0,00					gspD	TRANSMEMBRANE GENERAL SECRETORY PATHWAY PROTEIN D	IV.E.2
RSc3115	RS00568			0,80	0,00	-0,34	0,01					gspE	GENERAL SECRETION PATHWAY PROTEIN E	IV.E.2
RSc3116	RS00569			0,53	0,05	-0,35	0,04					gspF	TRANSMEMBRANE GENERAL SECRETORY PATHWAY PROTEIN F	IV.E.2
RSc3117	RS00571					-0,55	0,00	-1,17	0,01	-0,84	0,00		TRANSMEMBRANE	VI.C
RSc3118	RS00572			1,40	0,01	-0,41	0,00						TRANSMEMBRANE	VI.C
RSc3119	RS00574					-0,54	0,02						TRANSCRIPTION REGULATOR	VI.A
RSc3120	RS00575					-0,68	0,03						COG3467, Flavin-nucleotide-binding protein	VI.C
RSc3121	RS00576												HYPOTHETICAL PROTEIN	VI.D
RSc3122	RS00577												OXIDOREDUCTASE COG4231, Indolepyruvate ferredoxin oxidoreductase, alpha and	I
RSc3124	RS00480	0,92	0,01	-1,40	0,00								HYPOTHETICAL PROTEIN	VI.D
RSc3125	RS00478	1,34	0,00			-0,38	0,04	0,77	0,05			sbcB	EXODEOXYRIBONUCLEASE I	II.A.1
RSc3126	RS00477	0,93	0,01									eutC	ETHANOLAMINE AMMONIA-LYASE SMALL SUBUNIT	I.I.2
RSc3127	RS00476	0,98	0,02									eutB	ETHANOLAMINE AMMONIA-LYASE HEAVY CHAIN	I.I.2
RSc3128	RS00475			-1,06	0,02	0,70	0,00	0,95	0,02	-0,52	0,03	exaC	OXIDOREDUCTASE NAD+ DEPENDENT ACETALDEHYDE DEHYDROGENASE	I.I
RSc3129	RS00474	0,93	0,00			-0,64	0,00	0,54	0,04			acoR	TRANSCRIPTION REGULATOR ACETOIN CATABOLISM REGULATORY PROTEIN	I
RSc3130	RS00473	1,15	0,01	0,88	0,03	-0,50	0,00					adhA	OXIDOREDUCTASE PROPANOL-PREFERRING ALCOHOL DEHYDROGENASE	I.H.2
RSc3131	RS00472	1,07	0,01			-0,35	0,04					ldhA	OXIDOREDUCTASE D-LACTATE DEHYDROGENASE (D-LDH)	I.F
RSc3132	RS00471					-0,36	0,00						TRANSCRIPTION REGULATOR	VI.A
RSc3133	RS00470			-2,01	0,00	2,81	0,00	1,58	0,00				ANTIBIOTIC RESISTANCE (ACETYLTRANSFERASE)	IV.G.4
RSc3134	RS00469												TRANSMEMBRANE MSF TRANSPORTER	IV.D
RSc3135	RS00468					-0,52	0,01						CONSERVED HYPOTHETICAL PROTEIN	VI.C
RSc3136	RS00467												TRANSMEMBRANE METHYL-ACCEPTING CHEMOTAXIS PROTEIN	IV.C
RSc3137	RS00466					-0,68	0,00						CONSERVED HYPOTHETICAL PROTEIN	VI.C
RSc3138	RS00465					0,33	0,03						OXIDOREDUCTASE	I.I
RSc3139	RS00464	0,84	0,05			-0,68	0,00						TRANSCRIPTION REGULATOR	VI.A
RSc3140	RS00463	1,30	0,02	0,58	0,00	-0,68	0,02						SERINE PROTEASE	II.A.4
RSc3141	RS00462	1,07	0,04							-1,22	0,05		TRANSMEMBRANE LIPOPROTEIN	VI.D
RSc3142	RS00461												TRANSMEMBRANE TRANSPORT PROTEIN	IV.D
RSc3143	RS00460					-0,75	0,00						TRANSMEMBRANE SIGNAL TRANSDUCTION PROTEIN GGDEF DOMAIN	VI.A
RSc3144	RS00459	0,75	0,03										CONSERVED HYPOTHETICAL PROTEIN	VI.C
RSc3145	RS00458	1,81	0,00			-0,33	0,02					tISRso9	ISRso9-TRANSPOSASE	V.A
RSc3146	RS00457					-0,73	0,01						HYPOTHETICAL PROTEIN	VI.D
RSc3147	RS00456												ABC TRANSPORTER COG0683, ABC-type branched-chain amino acid transport syst	IV.D
RSc3148	RS00455												TRANSMEMBRANE	VI.B
RSc3149	RS00451					-0,90	0,01						TRANSCRIPTION REGULATOR	VI.A
RSc3150	RS00450					-0,29	0,02	1,68	0,00	1,26	0,00		TRANSMEMBRANE	VI.C
RSc3151	RS00448					0,76	0,00						OXIDOREDUCTASE TRYPTOPHAN 2-MONOOXYGENASE	VI.C
RSc3152	RS00447												OXIDOREDUCTASE COG1091, dTDP-4-dehydrorhamnose reductase	III.B
RSc3153	RS00446	0,59	0,01										HYPOTHETICAL PROTEIN	VI.D
RSc3154	RS00444					0,99	0,00						SIGNAL PEPTIDE	VI.C
RSc3155	RS00443			-3,34	0,00	4,17	0,00	4,24	0,00				SIGNAL PEPTIDE HYDROLASE	I.I
RSc3156	RS00442					-0,88	0,02	-1,09	0,01	-1,54	0,00	cspC	TRANSCRIPTION REGULATOR COLD SHOCK-LIKE PROTEIN CSPC	VI.A
RSc3157	RS00441			0,87	0,01								CONSERVED HYPOTHETICAL PROTEIN	VI.C
RSc3158	RS00440												TRANSMEMBRANE COG2081, Flavoprotein	VI.C
RSc3159	RS00439												COG2890, Methylase of polypeptide chain release factors	II.B.7
RSc3160	RS00438												TRANSCRIPTION REGULATOR TWO COMPONENT SENSOR HISTIDINE KINASE	VI.A
RSc3161	RS00437							-0,81	0,05				TRANSCRIPTION REGULATOR TWO COMPONENT RESPONSE REGULATOR	VI.A
RSc3162	RS00436			-2,41	0,00	1,68	0,00	3,61	0,00				HEMAGGLUTININ-RELATED AUTOTRANSPORTER PROTEIN	III.A
RSc3163	RS00435												TPR DOMAIN PROTEIN	VI.C
RSc3164	RS00434												CONSERVED HYPOTHETICAL PROTEIN	VI.C
RSc3165	RS00433												TRANSMEMBRANE	VI.C
RSc3166	RS00432											cysC1	ADENYLYLSULFATE KINASE (APS KINASE)	I.F.3
RSc3167	RS00430			-1,64	0,04								TPR DOMAIN PROTEIN	VI.B
RSc3168	RS00429	3,16	0,00	0,40	0,01	-0,61	0,00			-0,43	0,03	ISRso1	ISRso13-TRANSPOSASE	V.A
RSc3169	RS01160												TRANSMEMBRANE PSEUDOGENE (TRANSPORT PROTEIN, FRAGMENT)	VI.C
RSc3170	RS00428					-0,36	0,01						BACTERIOPHAGE-RELATED PROTEIN	V.D
RSc3171	RS00427					-0,43	0,04						SITE-SPECIFIC INTEGRASE/Resolvase	V
RSc3172	RS00426					-1,93	0,00	-1,36	0,00				BACTERIOPHAGE-RELATED PROTEIN	V.D
RSc3173	RS00425	0,47	0,04	-0,43	0,04	-1,45	0,00	-1,66	0,01				BACTERIOPHAGE-RELATED PROTEIN	V.D
RSc3174	RS04746					2,74	0,00	1,56	0,00				HYPOTHETICAL PROTEIN	VI.B
RSc3175	RS01339							-1,20	0,00	-0,34	0,00		TRANSMEMBRANE	VI.D
RSc3176	RS05984					-0,41	0,00						ACETYLTRANSFERASE	I
RSc3177	RS00423			0,28	0,05	-0,60	0,01						CONSERVED HYPOTHETICAL PROTEIN	VI.C
RSc3178	RS05983					-0,58	0,00			0,88	0,02		CONSERVED HYPOTHETICAL PROTEIN	VI.C
RSc3179	RS00422	2,22	0,00			-0,67	0,00						Transposase	V.A

RSc3180	RS05982			-0,43 0,00				HYPOTHETICAL PROTEIN	VI.D
RSc3181	RS00420							SIGNAL PEPTIDE Peptidyl-prolyl isomerase	II.B.7
RSc3182	RS00419		0,71 0,00	-0,59 0,00		-1,53 0,00		SIGNAL PEPTIDE ACTIVATION/SECRETION PROTEIN	IV.E.2
RSc3183	RS05936			-0,54 0,01				SIGNAL PEPTIDE HEMAGGLUTININ-RELATED PROTEIN	VI.C
RSc3184	RS02401			-0,42 0,00				CONSERVED HYPOTHETICAL PROTEIN	VI.C
RSc3185	RS02403				-1,13 0,05			HYPOTHETICAL PROTEIN	VI.D
RSc3186	RS02404							HYPOTHETICAL PROTEIN	VI.D
RSc3187	RS05978			-0,53 0,00				HYPOTHETICAL PROTEIN	VI.D
RSc3188	RS02405							HEMAGGLUTININ-RELATED PROTEIN	VI.B
RSc3189	RS05979							HYPOTHETICAL PROTEIN	VI.D
RSc3190	RS02407							CONSERVED HYPOTHETICAL PROTEIN	VI.C
RSc3191	RS02408			-0,83 0,01				CONSERVED HYPOTHETICAL PROTEIN	VI.C
RSc3192	RS02409		-0,95 0,04	0,77 0,00				Phage-related lysozyme (muraminidase)	V.D
RSc3193	RS02410			-0,73 0,02				BACTERIOPHAGE T7-RELATED GENE	V.D
RSc3194	RS02411							TRANSMEMBRANE HYPOTHETICAL PROTEIN	VI.C
RSc3195	RS02412			-0,63 0,05				TRANSMEMBRANE	VI.C
RSc3196	RS02413							CONSERVED HYPOTHETICAL PROTEIN	VI.C
RSc3197	RS05980				-1,74 0,01			TRANSMEMBRANE	VI.D
RSc3198	RS02414			-1,32 0,00	-0,70 0,03	-1,14 0,04		CONSERVED HYPOTHETICAL PROTEIN	VI.C
RSc3200	RS02416				-0,84 0,02		pseudoge	PSEUDOGENE (DEHYDROGENASE, FRAGMENT)	VI.C
RSc3201	RS02417			-0,64 0,02			pseudoge	PSEUDOGENE (DEHYDROGENASE, FRAGMENT)	VI.C
RSc3202	RS02419		-1,56 0,04					CONSERVED HYPOTHETICAL PROTEIN	VI.C
RSc3203	RS02420			-0,54 0,00				CONSERVED HYPOTHETICAL PROTEIN	VI.C
RSc3204	RS06022							CONSERVED HYPOTHETICAL PROTEIN	VI.C
RSc3205	RS02421							TRANSMEMBRANE DRUG EFFLUX PROTEIN	IV.D
RSc3206	RS02422			-0,56 0,05				TRANSPORT LIPOPROTEIN	IV.D
RSc3207	RS02423			-0,41 0,03				TRANSCRIPTION REGULATOR	VI.A
RSc3208	RS02424							DRUG EFFLUX LIPOPROTEIN	IV.D
RSc3209	RS02425							OXIDOREDUCTASE COG0300, Short-chain dehydrogenases of various substrate spe	I
RSc3210	RS02427			-0,22 0,04	0,65 0,01			CONSERVED HYPOTHETICAL PROTEIN	VI.C
RSc3211	RS02428				0,79 0,01	-0,44 0,01		CONSERVED HYPOTHETICAL PROTEIN	VI.C
RSc3212	RS02429		-3,35 0,00	2,83 0,00	2,04 0,00		RipT	TYPE III EFFECTOR PROTEIN RIPT	VI.E
RSc3213	RS02430			-0,58 0,03				CONSERVED HYPOTHETICAL PROTEIN	VI.C
RSc3214	RS02431							CONSERVED HYPOTHETICAL PROTEIN	VI.C
RSc3215	RS02432			-0,53 0,04				CONSERVED HYPOTHETICAL PROTEIN	VI.C
RSc3216	RS02433			-1,37 0,00				HYPOTHETICAL PROTEIN	VI.B
RSc3217	RS02434			-2,00 0,00				CONSERVED HYPOTHETICAL PROTEIN	VI.C
RSc3218	RS02435			-1,79 0,00				TRANSMEMBRANE	VI.B
RSc3219	RS02436			-2,08 0,00				TRANSMEMBRANE	VI.B
RSc3220	RS02437			-2,29 0,00				VGR-RELATED PROTEIN	VI.C
RSc3222	RS02439	1,00 0,02		-2,35 0,00				VGR-RELATED PROTEIN	VI.B
RSc3223	RS02440			-0,57 0,00				HYPOTHETICAL PROTEIN	VI.D
RSc3224	RS02441			-0,22 0,05	0,79 0,02			CONSERVED HYPOTHETICAL PROTEIN	VI.C
RSc3225	RS02442							CONSERVED HYPOTHETICAL PROTEIN	VI.C
RSc3226	RS02443							CONSERVED HYPOTHETICAL PROTEIN	VI.C
RSc3227	RS02444							CONSERVED HYPOTHETICAL PROTEIN	VI.C
RSc3228	RS02445							BACTERIOPHAGE-RELATED PROTEIN	V.D
RSc3229	RS02446							BACTERIOPHAGE-RELATED PROTEIN	V.D
RSc3230	RS02447							HYPOTHETICAL PROTEIN	VI.D
RSc3231	RS02448							HYPOTHETICAL PROTEIN	VI.D
RSc3232	RS02449			-0,20 0,05				BACTERIOPHAGE-RELATED PROTEIN	V.D
RSc3233	RS02450							HYPOTHETICAL PROTEIN	VI.D
RSc3234	RS02451							CONSERVED HYPOTHETICAL PROTEIN	VI.C
RSc3235	RS02452			-0,90 0,00				CONSERVED HYPOTHETICAL PROTEIN	VI.C
RSc3236	RS02453			-1,06 0,00				PHAGE INTEGRASE	V.D
RSc3237	RS02454							HYPOTHETICAL PROTEIN	VI.D
RSc3238	RS02455			-0,77 0,00				HYPOTHETICAL PROTEIN	V
RSc3239	RS02456			-0,69 0,00	-0,72 0,02	-0,74 0,00		EXCISIONASE	V.B
RSc3240	RS02457			-0,41 0,00	0,89 0,02	0,86 0,02		TRANSMEMBRANE	VI.D
RSc3241	RS02458			-0,58 0,03			pseudoge	N-TERMINAL PART OF A TRUNCATED YOPP/AVRRVX-RELATED PROTEIN	VI.C
RSc3243	RS02460						pseudoge	C-TERMINAL PART OF A TRUNCATED YOPP/AVRRVX-RELATED PROTEIN	VI.C
RSc3244	RS02461			-0,78 0,03				HYPOTHETICAL PROTEIN	VI.D
RSc3245	RS02463			-0,23 0,04				HYPOTHETICAL PROTEIN	VI.D
RSc3246	RS02464			-0,44 0,04				BACTERIOPHAGE-RELATED PROTEIN	V.D
RSc3247	RS02465			-0,69 0,00				SITE-SPECIFIC INTEGRASE	V.A
RSc3248	RS02466			-2,02 0,00	-1,32 0,00			BACTERIOPHAGE-RELATED PROTEIN	V.D
RSc3249	RS02479			-1,48 0,00		0,76 0,03		SIGNAL PEPTIDE	VI.C
RSc3250	RS02480	0,67 0,03	0,57 0,03	-1,44 0,00		1,13 0,00		SIGNAL PEPTIDE	VI.D
RSc3251	RS02467			-0,43 0,02	-1,05 0,01			CONSERVED HYPOTHETICAL PROTEIN	VI.C
RSc3252	RS02481			-0,37 0,03				COG2856, ZINC PEPTIDASE	II.A.4
RSc3253	RS02482			-0,34 0,04				SIGNAL PEPTIDE SULFUR OXIDATION PROTEIN	I.F.3
RSc3254	RS02483			-0,67 0,01				SIGNAL PEPTIDE COG0526, Thiol-disulfide isomerase and thioredoxins	II.B.7
RSc3255	RS02484		0,54 0,00	-0,45 0,00				TRANSMEMBRANE	VI.C
RSc3256	RS02485			-0,33 0,00				CONSERVED HYPOTHETICAL PROTEIN	VI.C
RSc3257	RS02486			-0,34 0,00				SIGNAL PEPTIDE	VI.C
RSc3258	RS02487				-0,65 0,04			CONSERVED HYPOTHETICAL PROTEIN	VI.C
RSc3259	RS02488			0,75 0,00				TRANSMEMBRANE	VI.C
RSc3260	RS02489			-0,32 0,01				TRANSMEMBRANE CYTOCHROME C551/C552	I.G.1
RSc3261	RS02490		1,05 0,04	-0,65 0,00				TRANSMEMBRANE	VI.C
RSc3262	RS02491			-0,45 0,00			soxF	OXIDOREDUCTASE SULFIDE DEHYDROGENASE FLAVOCYTOCHROME C	I.F.3
RSc3263	RS02492			-0,45 0,01				SIGNAL PEPTIDE COG2863, Cytochrome c553	I.G.1
RSc3264	RS02493			-0,46 0,00				SIGNAL PEPTIDE COG2823, Periplasmic or secreted lipoprotein	VI.C
RSc3265	RS02494			-0,76 0,00				COG0792, Endonuclease distantly related to archaeal Holliday junction resolvase	II.B.8
RSc3266	RS02495							TRANSMEMBRANE COG0313, Methyltransferase	VI.C
RSc3267	RS02496			-0,39 0,01				LIPOPROTEIN	VI.C
RSc3268	RS02497			-0,80 0,02		0,52 0,05		COG0491, Zn-dependent hydrolases, including glyoxylases	I.I
RSc3269	RS02498		0,82 0,00	-0,30 0,01				TRANSMEMBRANE	VI.C
RSc3270	RS02499			-0,57 0,01	-0,44 0,01			TRANSMEMBRANE	VI.C
RSc3271	RS02500			-0,36 0,01		1,90 0,04		OXIDOREDUCTASE ARSENATE REDUCTASE	I
RSc3272	RS02501	-2,42 0,00	-2,70 0,00	2,47 0,00	3,58 0,00			HYPOTHETICAL PROTEIN	VI.D
RSc3273	RS02502							COG0624, Acetylornithine deacetylase/Succinyl-diaminopimelate desuccinylase and r	I
RSc3274	RS02503							ALLANTOICASE (ALLANTOINE AMIDINOHYDROLASE)	I.I.2
RSc3275	RS02504						dctA3	TRANSMEMBRANE C4-DICARBOXYLATE TRANSPORT PROTEIN	IV.D.3
RSc3276	RS02505			-0,55 0,02				TRANSCRIPTION REGULATOR	VI.A
RSc3277	RS02506			-0,45 0,02				CONSERVED HYPOTHETICAL PROTEIN	VI.C
RSc3278	RS02507							TRANSCRIPTION REGULATOR	VI.A
RSc3279	RS02508			-0,56 0,00			relE	Cytotoxic translational repressor of toxin-antitoxin stability system	IV.A
RSc3280	RS02509				-0,83 0,00			TRANSCRIPTION REGULATOR	VI.A
RSc3281	RS02510			-0,67 0,05			gcl	GLYOXYLATE CARBOLIGASE	I.F.18
RSc3282	RS02511				1,70 0,00		hyi	HYDROXYPYRUVATE ISOMERASE	I.F.18
RSc3283	RS02512						glxR	OXIDOREDUCTASE TRANSMEMBRANE 2-HYDROXY-3-OXOPROPIONATE REDU	I.I
RSc3284	RS02513			-0,42 0,04	-0,48 0,03		hemN	OXIDOREDUCTASE OXYGEN-INDEPENDENT COPROPORPHYRINOGEN III OXID	I.B.4
RSc3285	RS02514		-0,62 0,01	-0,31 0,00	-1,94 0,02			SIGNAL PEPTIDE ABC-type branched-chain amino acid transport systems, periplasm	IV.D.2
RSc3286	RS02515			-0,59 0,02			solI	AUTOINDUCER SYNTHESIS PROTEIN SOLI	I.E.1
RSc3287	RS02516			-0,82 0,00			solR	TRANSCRIPTION REGULATOR TRANSCRIPTIONAL ACTIVATOR PROTEIN SOLR	I.E.1
RSc3288	RS02517			-1,25 0,00	1,89 0,01	4,31 0,00		SUGAR-BINDING LECTIN	I.F.7
RSc3289	RS02518				0,81 0,00		rep	ATP-DEPENDENT DNA HELICASE REP	II.B.8
RSc3290	RS02519		-4,05 0,00	4,32 0,00	2,44 0,00			CONSERVED HYPOTHETICAL PROTEIN	VI.C
RSc3291	RS02520			1,39 0,00		1,38 0,04		SIGNAL PEPTIDE	VI.C
RSc3292	RS02521	0,84 0,01		0,50 0,01	1,40 0,00			TRANSMEMBRANE	VI.C
RSc3293	RS02522	1,86 0,03	1,10 0,01				gcvT	AMINOMETHYLTRANSFERASE (GLYCINE CLEAVAGE SYSTEM T PROTEIN)	I.F.18
RSc3294	RS02523		0,96 0,02	-0,65 0,00			gcvH	GLYCINE CLEAVAGE SYSTEM H PROTEIN	I.F.18

RSc3295	RS02524			1,27	0,01	-0,75	0,01			-0,79	0,00	gcvP	OXIDOREDUCTASE TRANSMEMBRANE GLYCINE DEHYDROGENASE [DECARB	I.F.18
RSc3296	RS02527			1,81	0,03	-0,83	0,01			2,20	0,00	sdaA2	L-SERINE AMONYA-LYASE, L-SERINE DEHYDRATASE 2 (L-SERINE DEAMINASE	I.I.3
RSc3297	RS02525					-0,57	0,00						TRANSMEMBRANE	VI.C
RSc3298	RS02526					-0,51	0,01						CONSERVED HYPOTHETICAL PROTEIN	VI.C
RSc3299	RS02528			0,66	0,03			1,20	0,01				TRANSMEMBRANE	VI.C
RSc3300	RS02529			0,76	0,00	-0,99	0,00	0,75	0,01	0,98	0,04		SIGNAL PEPTIDE AMINO-ACID TRANSPORT PROTEIN	IV.D.2
RSc3301	RS02530					-1,08	0,00			0,88	0,04	putA	OXIDOREDUCTASE BIFUNCTIONAL PROTEIN: PROLINE DEHYDROGENASE ANI	I.I.3
RSc3302	RS02531			0,45	0,01	-0,55	0,00	-0,93	0,03			priA	PRIMOSOMAL PROTEIN N' (REPLICATION FACTOR Y)	II.B.8
RSc3303	RS02532			0,59	0,01							hemE	UROPORPHYRINOGEN DECARBOXYLASE	I.B.4
RSc3304	RS02533					-0,52	0,00						SIGNAL PEPTIDE	VI.C
RSc3305	RS02534					-1,14	0,00			0,89	0,02		CONSERVED HYPOTHETICAL PROTEIN	VI.C
RSc3306	RS02535					-1,21	0,00						HYPOTHETICAL PROTEIN	VI.D
RSc3307	RS02536			0,35	0,02	-0,61	0,00						TRANSMEMBRANE METHYL-ACCEPTING CHEMOTAXIS PROTEIN	IV.C
RSc3308	RS02537			0,99	0,01	-0,73	0,04						TRANSCRIPTION REGULATOR	VI.A
RSc3309	RS02538											gstM	GLUTATHIONE S-TRANSFERASE	II.B.7
RSc3310	RS02539											pstS2	ABC TRANSPORTER PHOSPHATE-BINDING PERIPLASMIC PROTEIN PRECURS(	I.F.20
RSc3311	RS02540					-1,29	0,00			0,98	0,01		HYPOTHETICAL PROTEIN	VI.D
RSc3312	RS02542			0,54	0,01								ESTERASE / LIPASE	I.I.1
RSc3313	RS02543					-0,69	0,00			-0,79	0,03		CONSERVED HYPOTHETICAL PROTEIN	VI.C
RSc3314	RS02544					-1,16	0,00						SIGNAL PEPTIDE	VI.D
RSc3315	RS02545									2,74	0,00		TRANSMEMBRANE	VI.C
RSc3316	RS02546			0,83	0,01					-0,60	0,04	atpC1	ATP SYNTHASE EPSILON CHAIN	I.E.2
RSc3317	RS02547			0,90	0,00			1,51	0,00			atpD	ATP SYNTHASE BETA CHAIN	I.E.2
RSc3318	RS02548			0,72	0,00			0,97	0,00			atpG	ATP SYNTHASE GAMMA CHAIN	I.E.2
RSc3319	RS02549	-0,27	0,04	0,89	0,00	-0,22	0,02			-0,76	0,01	atpA	ATP SYNTHASE ALPHA CHAIN	I.E.2
RSc3320	RS02550							1,00	0,00			atpH	ATP SYNTHASE DELTA CHAIN	I.E.2
RSc3321	RS02551			0,61	0,02			0,81	0,00			atpF	TRANSMEMBRANE ATP SYNTHASE B CHAIN	I.E.2
RSc3322	RS02552			0,60	0,00			0,88	0,00			atpE	TRANSMEMBRANE ATP SYNTHASE C CHAIN (LIPID-BINDING PROTEIN)	I.E.2
RSc3323	RS02553			0,87	0,01			1,01	0,00	-0,78	0,00	atpB	TRANSMEMBRANE ATP SYNTHASE A CHAIN	I.E.2
RSc3324	RS02554					-0,32	0,02					atpI	TRANSMEMBRANE F0F1-type ATP synthase, subunit I	I.E.2
RSc3325	RS02555			0,67	0,01	-0,34	0,03					parB2	CHROMOSOME PARTITIONING PROTEIN PARB	IV.A
RSc3326	RS02560			0,60	0,02	-0,27	0,04					parA2	CHROMOSOME PARTITIONING PROTEIN PARA	IV.A
RSc3327	RS02561					-0,36	0,04					gidB	GLUCOSE INHIBITED DIVISION PROTEIN B	IV.A
RSc3328	RS02562			0,89	0,03			0,52	0,04			gidA	GLUCOSE INHIBITED DIVISION PROTEIN A	IV.A
RSc3329	RS02563					-0,83	0,00						ABC TRANSPORTER AMINO-ACID-BINDING PERIPLASMIC PROTEIN (PBP)	IV.D.2
RSc3330	RS02564					-0,56	0,00	-0,64	0,01				SIGNAL PEPTIDE	VI.D
RSc3331	RS02565					-0,45	0,05						COG1028, Dehydrogenases with different specificities (related to short-chain alcohol (	I
RSc3332	RS02566					0,67	0,00	-0,92	0,01				TRANSCRIPTION REGULATOR	VI.A
RSc3333	RS02567									1,33	0,03		CONSERVED HYPOTHETICAL PROTEIN	VI.C
RSc3334	RS02568												TRANSCRIPTION REGULATOR	VI.A
RSc3335	RS02569					-1,24	0,01	1,94	0,00				COG0451, Nucleoside-diphosphate-sugar epimerase	I
RSc3336	RS02570			-0,75	0,02	-0,73	0,00						CONSERVED HYPOTHETICAL PROTEIN	VI.C
RSc3337	RS02571			1,49	0,04	-0,52	0,05						TRANSMEMBRANE COG0730, Permease	IV.D
RSc3338	RS02572					-0,40	0,00						TRANSMEMBRANE	VI.D
RSc3339	RS02574					-1,37	0,00			-0,39	0,04		HYPOTHETICAL PROTEIN	VI.B
RSc3340	RS02575												ABC TRANSPORTER TRANSMEMBRANE PROTEIN	IV.D
RSc3341	RS02576												ABC TRANSPORTER TRANSMEMBRANE PROTEIN	IV.D
RSc3342	RS02578					-0,58	0,00						ABC TRANSPORTER SUBSTRATE-BINDING PERIPLASMIC PROTEIN (PBP)	IV.D
RSc3343	RS02579					-0,70	0,06						ABC TRANSPORTER ATP-BINDING PROTEIN	IV.D
RSc3344	RS02581					-0,74	0,00						ABC TRANSPORTER ATP-BINDING PROTEIN	IV.D
RSc3345	RS02583			0,47	0,04							betA	OXIDOREDUCTASE CHOLINE DEHYDROGENASE LIPOPROTEIN	I.F
RSc3346	RS02584			0,34	0,04	-0,71	0,01						LYSOPHOSPHOLIPASE	I.I.1
RSc3347	RS02622					-1,71	0,00						TRANSCRIPTION REGULATOR	VI.A
RSc3348	RS02623					-1,53	0,00						TRANSMEMBRANE CATION-TRANSPORTING ATPASE	IV.D.4
RSc3349	RS02624					-1,63	0,00	-2,04	0,00			copZ	COG2608, Copper chaperone	IV.G.4
RSc3350	RS02625					-0,71	0,00					ompW	SIGNAL PEPTIDE OUTER MEMBRANE PROTEIN W	III.B.2
RSc3352	RS02627					-0,68	0,00						COG2423, Ornithine cyclodeaminase	I
RSc3353	RS02628			0,55	0,01	-0,60	0,00						TRANSMEMBRANE	VI.C
RSc3354	RS02629					-0,56	0,00			-0,89	0,00		TRANSCRIPTION REGULATOR	VI.A
RSc3355	RS02630					-0,56	0,05					phhA	OXIDOREDUCTASE PHENYLALANINE 4-HYDROXYLASE	I.I.3
RSc3356	RS02631											phhB	PTERIN-4-ALPHA-CARBINOLAMINE DEHYDRATASE	I
RSc3357	RS02632					-0,66	0,00	-0,50	0,02	0,44	0,05		HYPOTHETICAL PROTEIN	VI.D
RSc3358	RS02633												SET DOMAIN PROTEIN	VI.C
RSc3359	RS02634							-0,33	0,05			dbhB	HISTONE-LIKE DNA-BINDING PROTEIN HU-BETA (NS1)	II.C.1
RSc3360	RS02635					-0,67	0,01			-0,32	0,05		TRANSMEMBRANE	VI.C
RSc3361	RS02636					-0,73	0,00						OXIDOREDUCTASE COG1893, Ketopantoate reductase	I
RSc3362	RS02637									0,74	0,01		CONSERVED HYPOTHETICAL PROTEIN	VI.C
RSc3363	RS02638					-0,36	0,05					cpdB	TRANSMEMBRANE 2',3'-CYCLIC-NUCLEOTIDE 2'-PHOSPHODIESTERASE PRECU	I.F.1
RSc3364	RS02639											minC	CELL DIVISION INHIBITOR	IV.A
RSc3365	RS02640					-0,38	0,00	0,32	0,04			minD	SEPTUM SITE-DETERMINING PROTEIN MIND	IV.A
RSc3366	RS02641			0,49	0,00							minE	CELL DIVISION TOPOLOGICAL SPECIFICITY FACTOR	IV.A
RSc3367	RS02642					-0,37	0,00	-1,15	0,00				COG1804, CaiB, Predicted acyl-CoA transferases/carnitine dehydratase	I
RSc3368	RS02643					-0,94	0,00					comJ	COG2961, Protein involved in catabolism of external DNA	II
RSc3369	RS02644			-2,28	0,00	2,63	0,00						AVRPPHE AVIRULENCE PROTEIN-HOMOLOG	VI.B
RSc3370	RS02645					-0,57	0,02	-0,45	0,03	0,97	0,04		COG1254, ACYLPHOSPHATASE	I.H
RSc3371	RS02646			0,71	0,00								ABC TRANSPORTER TRANSMEMBRANE PROTEIN	IV.D
RSc3372	RS02647			0,72	0,00	-0,51	0,03						ABC TRANSPORTER COMPOSITE ATP-BINDING TRANSMEMBRANE PROTEIN	IV.D
RSc3373	RS02648												TRANSCRIPTION REGULATOR	VI.A
RSc3374	RS02649					-0,54	0,00	-0,56	0,01			pfs	BIFUNCTIONAL PROTEIN (MTA/SAH NUCLEOSIDASE) (P46): 5'-METHYLTHIOAD	I.F.9
RSc3375	RS02660					-1,07	0,00			1,26	0,00		CONSERVED HYPOTHETICAL PROTEIN	VI.C
RSc3376	RS02650	0,60	0,04			-1,68	0,00			2,67	0,00		SIGNAL PEPTIDE	VI.C
RSc3377	RS02651	1,53	0,01			-1,80	0,00	1,30	0,00	3,90	0,00		CONSERVED HYPOTHETICAL PROTEIN	VI.C
RSc3378	RS02652					-2,03	0,00	1,10	0,00	3,79	0,00		COG1502, Phosphatidylserine/phosphatidylglycerophosphate/cardiolipin synthases a	I
RSc3379	RS02653					-0,37	0,00						SIGNAL PEPTIDE	VI.C
RSc3380	RS02654					-0,80	0,00	-0,33	0,04				SIGNAL PEPTIDE	VI.D
RSc3381	RS02655					-1,16	0,00						TRANSMEMBRANE	VI.D
RSc3382	RS02656											kdpA	TRANSMEMBRANE POTASSIUM-TRANSPORTING ATPASE A CHAIN	IV.D.4
RSc3383	RS02657											kdpB	TRANSMEMBRANE POTASSIUM-TRANSPORTING ATPASE (B CHAIN)	IV.D.4
RSc3384	RS02658											kdpC	TRANSMEMBRANE POTASSIUM-TRANSPORTING ATPASE C CHAIN	IV.D.4
RSc3385	RS02659											kdpD	TRANSCRIPTION REGULATOR TWO-COMPONENT SENSOR KINASE PROTEIN K	IV.D
RSc3386	RS05666							-2,30	0,02	-1,74	0,01		ABC TRANSPORTER COG1464, ABC-type metal ion transport system, periplasmic c	IV.D.5
RSc3387	RS05667					-0,78	0,00	0,48	0,03				TRANSCRIPTION REGULATOR TWO-COMPONENT RESPONSE REGULATOR WI	VI.A
RSc3388	RS05668												TRANSCRIPTION REGULATOR TWO-COMPONENT RESPONSE REGULATOR	VI.A
RSc3389	RS05670												HYPOTHETICAL PROTEIN	VI.B
RSc3390	RS05671									-1,42	0,00		SIGNAL PEPTIDE PORIN	IV
RSc3391	RS05672												ABC TRANSPORTER COG0715, ABC-type nitrate/sulfonate/bicarbonate transport sy	IV.D
RSc3392	RS05673												TRANSCRIPTION REGULATOR RESPONSE REGULATOR	VI.A
RSc3393	RS05676					-0,60	0,00					tISRso5	ISRso5-TRANSPOSASE	V.A
RSc3394	RS05975												RESTRICTION MODIFICATION ENZYME R SUBUNIT	II.B.8
RSc3395	RS01703												CONSERVED HYPOTHETICAL PROTEIN	VI.C
RSc3396	RS01704												TYPE I RESTRICTION ENZYME M PROTEIN	II.B.8
RSc3397	RS01705					-0,83	0,00	-0,78	0,04				TRANSCRIPTION REGULATOR COG1959	VI.A
RSc3398	RS01706			-0,76	0,03								TRANSMEMBRANE FLAVOHEMOPROTEIN (HEMOGLOBIN-LIKE PROTEIN)	I.G.1
RSc3399	RS01707												TRANSMEMBRANE	VI.C
RSc3400	RS01708			0,75	0,03								TRANSMEMBRANE TRANPORTER	IV.D
RSc3401	RS01709												SKWP PROTEIN 1	VI.C
RSc3402	RS01711			-0,82	0,00	1,07	0,02						TRANSMEMBRANE	VI.B
RSc3403	RS01712					-0,72	0,03						TRANSCRIPTION REGULATOR TWO-COMPONENT SENSOR KINASE	VI.A
RSc3404	RS01713					-0,53	0,01						TRANSCRIPTION REGULATOR RESPONSE REGULATOR	VI.A
RSc3405	RS01714			-0,37	0,03								TRANSMEMBRANE	VI.C
RSc3406	RS01715												COG0596, Predicted hydrolases or acyltransferases (alpha/beta hydrolase superfamil	I
RSc3407	RS01716												OXIDOREDUCTASE COG0665, Glycine/D-amino acid oxidase (deaminating)	I.I.3

RSc3408	RS01717							ABC TRANSPORTER ATP-BINDING PROTEIN	IV.D.2
RSc3409	RS01718							ABC TRANSPORTER AMINO-ACID TRANSMEMBRANE PROTEIN	IV.D.2
RSc3410	RS01719							ABC TRANSPORTER AMINO ACID-BINDING PERIPLASMIC PROTEIN (PBP)	IV.D.2
RSc3411	RS01720			-0,50 0,01				TRANSCRIPTION REGULATOR	VI.A
RSc3412	RS01721			-0,59 0,01			cheD4	TRANSMEMBRANE METHYL-ACCEPTING CHEMOTAXIS PROTEIN I (SERINE CHI	IV.C
RSc3413	RS01722			-0,44 0,01	0,34 0,02			CONSERVED HYPOTHETICAL PROTEIN	VI.C
RSc3414	RS01793				0,74 0,03			TRANSMEMBRANE	VI.C
RSc3415	RS01795							HYPOTHETICAL PROTEIN	VI.B
RSc3416	RS01796				1,05 0,00			TRANSMEMBRANE COG0551, Zn-finger domain associated with topoisomerase type	VI.C
RSc3417	RS01797			0,81 0,01	1,89 0,02			CONSERVED HYPOTHETICAL PROTEIN	VI.C
RSc3418	RS01798							HYPOTHETICAL PROTEIN	VI.D
RSc3419	RS01799			-0,67 0,03				CONSERVED HYPOTHETICAL PROTEIN	VI.C
RSc3420	RS05815			-0,59 0,00	-2,03 0,02			HYPOTHETICAL PROTEIN	VI.D
RSc3422	RS01802		0,80 0,01	-0,62 0,01	-1,99 0,01			HYPOTHETICAL PROTEIN	VI.D
RSc3423	RS01803		1,10 0,05	-0,80 0,03				HYPOTHETICAL PROTEIN	VI.D
RSc3424	RS01804			-0,80 0,00	-1,69 0,00	-1,19 0,00		HYPOTHETICAL PROTEIN	VI.D
RSc3425	RS01805			-0,75 0,00		-0,95 0,05		CONSERVED HYPOTHETICAL PROTEIN	VI.C
RSc3426	RS01806				3,20 0,00			TRANSMEMBRANE	VI.B
RSc3427	RS01807				1,30 0,03			HYPOTHETICAL PROTEIN	VI.B
RSc3428	RS05816			0,31 0,01				CONSERVED HYPOTHETICAL PROTEIN	VI.C
RSc3429	RS01809	0,30 0,04	0,98 0,00	-0,60 0,00		-1,15 0,01		CONSERVED HYPOTHETICAL PROTEIN	VI.C
RSc3430	RS01810							VGR-RELATED PROTEIN	VI.B
RSc3431	RS01811							HYPOTHETICAL PROTEIN	VI.D
RSc3432	RS01812							HYPOTHETICAL PROTEIN	VI.D
RSc3433	RS01813							HYPOTHETICAL PROTEIN	VI.D
RSc3434	RS01814			-0,32 0,02				HYPOTHETICAL PROTEIN	VI.D
RSc3435	RS01815							HYPOTHETICAL PROTEIN	VI.D
RSc3436	RS01817							CONSERVED HYPOTHETICAL PROTEIN	VI.C
RSc3437	RS01818							CONSERVED HYPOTHETICAL PROTEIN	VI.C
RSc3438	RS01819							SITE-SPECIFIC DNA METHYLASE	II.B.8
RSc3439	RS01820					0,42 0,03	vsr	PATCH REPAIR PROTEIN (DNA MISMATCH ENDONUCLEASE)	II.B.8
RSc3440	RS01821						gyrB	DNA GYRASE (SUBUNIT B)	II.B.8
RSc3441	RS01822						dnaN	DNA POLYMERASE III (BETA CHAIN)	II.B.8
RSc3442	RS01823		0,97 0,04				dnaA	CHROMOSOMAL REPLICATION INITIATOR PROTEIN DNAA	II.B.8
RSp0001	RS02238				-1,03 0,01	-1,33 0,01	repA	REPA REPLICASE PROTEIN	V.B
RSp0002	RS05790	2,47 0,00				3,41 0,00	cspD3	TRANSCRIPTION REGULATOR COLD SHOCK-LIKE PROTEIN	VI.A
RSp0003	RS01975					-0,91 0,00	parA	PARTITION PROTEIN	V.B
RSp0004	RS01976			-0,31 0,05			parB	PARTITIONING PROTEIN	II.B.8
RSp0005	RS01977				-1,16 0,01	-2,11 0,00		CONSERVED HYPOTHETICAL PROTEIN	VI.C
RSp0006	RS01978				0,96 0,02		galU2	UTP--GLUCOSE-1-PHOSPHATE URIDYLYLTRANSFERASE UDP-GLUCOSE PYR(	I.I.4
RSp0007	RS01979						kgtP2	TRANSMEMBRANE ALPHA-KETOGLUTARATE PERMEASE	IV.D.3
RSp0008	RS01980						dctB1	TRANSCRIPTION REGULATOR C4-DICARBOXYLATE TRANSPORT SENSOR KIN/	IV.D.3
RSp0009	RS01981		-0,85 0,02				dctD1	TRANSCRIPTION REGULATOR RESPONSE REGULATOR OF THE C4-DICARBOX	IV.D.3
RSp0010	RS01982			-0,66 0,00				TRANSMEMBRANE LIPOPROTEIN	VI.C
RSp0011	RS01983					-1,09 0,00		SIGNAL PEPTIDE	VI.D
RSp0012	RS01984		1,19 0,05	-0,27 0,01				TRANSMEMBRANE SIGNAL TRANSDUCTION PROTEIN GGDEF DOMAIN	VI.A
RSp0013	RS01985		0,89 0,00	-0,61 0,00				ABC TRANSPORTER AMINO ACID-BINDING PERIPLASMIC PROTEIN (PBP)	IV.D.2
RSp0014	RS01986		0,98 0,02	-0,40 0,00				ABC TRANSPORTER AMINO-ACID TRANSMEMBRANE LIPOPROTEIN	IV.D.2
RSp0015	RS01987		1,82 0,01	-0,34 0,00				ABC TRANSPORTER AMINO-ACID TRANSMEMBRANE PROTEIN	IV.D.2
RSp0016	RS01988		1,19 0,00					ABC TRANSPORTER AMINO-ACID ATP-BINDING PROTEIN	IV.D.2
RSp0017	RS01989		0,87 0,01	-0,42 0,01				ABC TRANSPORTER AMINO-ACID ATP-BINDING PROTEIN	IV.D.2
RSp0018	RS01990							TRANSCRIPTION REGULATOR TRANSCRIPTIONAL REGULATOR	VI.A
RSp0019	RS01991							CONSERVED HYPOTHETICAL PROTEIN	VI.C
RSp0020	RS01992			-0,63 0,00				TRANSMEMBRANE CHLORIDE CHANNEL PROTEIN	IV.D
RSp0021	RS01993			-0,60 0,02				HYPOTHETICAL PROTEIN	VI.D
RSp0022	RS01995			-0,60 0,01				HYPOTHETICAL PROTEIN	VI.D
RSp0023	RS01997			-0,95 0,00				SIGNAL PEPTIDE	VI.C
RSp0024	RS01998			-0,52 0,01				COG1881, Phospholipid-binding protein	I
RSp0025	RS06177			-1,03 0,00	-2,14 0,01	-1,40 0,01		HYPOTHETICAL PROTEIN	VI.B
RSp0026	RS02001			-0,64 0,00	-1,72 0,01	-0,53 0,02		HYPOTHETICAL PROTEIN	VI.D
RSp0027	RS02002						mntH1	TRANSMEMBRANE MANGANESE TRANSPORT PROTEIN	IV.D.1
RSp0028	RS02003		-2,73 0,00	1,09 0,00	1,61 0,00			LRR-GALA FAMILY TYPE III EFFECTOR PROTEIN (GALA 3)	VI.C
RSp0029	RS02004				-1,06 0,00	-0,33 0,02		TRANSCRIPTION REGULATOR HNS-LIKE PROTEIN	VI.A
RSp0030	RS02005		0,59 0,03					SIGNAL PEPTIDE OXACILLIN HYDROLASE (CLASS-D BETA-LACTAMASE)	IV.G.4
RSp0031	RS02006		0,36 0,02	-0,65 0,01				TRANSMEMBRANE	VI.C
RSp0032	RS02007			-0,86 0,00	0,45 0,00	-1,46 0,00		OXIDOREDUCTASE PROTEIN WITH COG1902, NADH:flavin oxidoreductases AND (	I
RSp0033	RS02008			-0,28 0,02				OXIDOREDUCTASE	I
RSp0034	RS02009			-0,47 0,02		-1,85 0,01		TRANSCRIPTION REGULATOR	VI.A
RSp0035	RS02010			-0,37 0,00				ENOYL-COA HYDRATASE	I.I.4
RSp0036	RS02011					1,85 0,00		OXIDOREDUCTASE ACYL-CoA DEHYDROGENASE	I.I.1
RSp0037	RS02012							ACETYL-COENZYME A SYNTHETASE (ACETATE--CoA LIGASE)	I.C
RSp0038	RS02013							THIOESTERASE	I.I
RSp0039	RS02014		0,60 0,04	-0,48 0,04				COG0251, Translation initiation inhibitor	II.B.7
RSp0040	RS02015			-0,75 0,01				TRANSMEMBRANE	VI.D
RSp0043	RS02018							HYPOTHETICAL PROTEIN	VI.D
RSp0044	RS02019			-1,16 0,02				TRANSCRIPTION REGULATOR	VI.A
RSp0045	RS02020			-0,61 0,02			sdaA1	L-SERINE DEHYDRATASE (L-SERINE DEAMINASE)	I.I.3
RSp0046	RS02021						soxB1	OXIDOREDUCTASE SARCOSINE OXIDASE (BETA SUBUNIT)	I.I
RSp0047	RS02022						soxD1	OXIDOREDUCTASE SARCOSINE OXIDASE (DELTA SUBUNIT)	I.I
RSp0048	RS02023						soxA1	OXIDOREDUCTASE SARCOSINE OXIDASE (ALPHA SUBUNIT)	I.I
RSp0049	RS02024						soxG1	OXIDOREDUCTASE SARCOSINE OXIDASE (GAMMA SUBUNIT)	I.I
RSp0050	RS02025			-0,40 0,00			folB1	DIHYDRONEOPTERIN ALDOLASE	I.B.14
RSp0051	RS02026							SIGNAL PEPTIDE PORIN	IV.D
RSp0052	RS02027		0,62 0,04					TRANSMEMBRANE METABOLITE TRANSPORT PROTEIN	IV.D
RSp0053	RS02028	-0,72 0,03					fdhA	OXIDOREDUCTASE GLUTATHIONE-INDEPENDENT FORMALDEHYDE DEHYDRO	I.I.4
RSp0054	RS02029			-0,97 0,03				TRANSCRIPTION REGULATOR TRANSCRIPTIONAL REGULATOR	VI.A
RSp0055	RS02030				2,16 0,00		glyA2	SERINE HYDROXYMETHYLTRANSFERASE	I.A.11
RSp0056	RS02031							DIPEPTIDASE	II.A.4
RSp0057	RS02032							CONSERVED HYPOTHETICAL PROTEIN	VI.C
RSp0058	RS02033						stcD	OXIDOREDUCTASE STACHYDRINE UTILIZATION PROTEIN	II.A.4
RSp0059	RS02034							OXIDOREDUCTASE COG0247 Fe-S OXIDOREDUCTASE	I
RSp0060	RS02035		0,64 0,05				eftA	ELECTRON TRANSFER FLAVOPROTEIN ALPHA-SUBUNIT (ALPHA-ETF)	I.G.1
RSp0061	RS02036			-0,35 0,05			eftB	ELECTRON TRANSFER FLAVOPROTEIN BETA-SUBUNIT	I.G.1
RSp0062	RS02037							OXIDOREDUCTASE DIOXYGENASE (ALPHA SUBUNIT)	I
RSp0063	RS02038							OXIDOREDUCTASE FERREDOXIN	I.G.1
RSp0064	RS02039			1,10 0,04			opuC	ABC TRANSPORTER GLYCINE BETAINE TRANSMEMBRANE AND PERIPLASMIC	IV.D.2
RSp0065	RS02040						purU2	FORMYLTETRAHYDROFOLATE DEFORMYLASE	I.D.2
RSp0066	RS02041			-0,79 0,02			opuA	ABC TRANSPORTER GLYCINE BETAINE/L-PROLINE ATP-BINDING PROTEIN	IV.D.2
RSp0067	RS02042							ABC TRANSPORTER TRANSMEMBRANE PROTEIN	IV.D
RSp0068	RS02043							TRANSCRIPTION REGULATOR TRANSCRIPTIONAL REGULATOR	VI.A
RSp0069	RS02044		0,68 0,02	-0,55 0,04			adhC1	OXIDOREDUCTASE BIFUNCTIONAL PROTEIN: GLUTATHIONE-DEPENDENT FOR	I.H
RSp0070	RS02045				0,73 0,00			OXIDOREDUCTASE FORMATE DEHYDROGENASE	I.H.2
RSp0071	RS02048					2,25 0,00		CONSERVED HYPOTHETICAL PROTEIN	VI.C
RSp0072	RS02049	-2,60 0,02				2,61 0,00		CONSERVED HYPOTHETICAL PROTEIN	VI.C
RSp0073	RS05558		0,45 0,04					REMNANT OF A TRANSPOSASE GENE	V.A
RSp0074	RS02050			-0,96 0,03				SIGNAL PEPTIDE	VI.D
RSp0075	RS05546						kgtP1	TRANSMEMBRANE ALPHA-KETOGLUTARATE PERMEASE	IV.D.3
RSp0076	RS05545			-0,43 0,01				COG3636, Predicted transcriptional regulator	VI.A
RSp0077	RS05544			-0,45 0,01				CONSERVED HYPOTHETICAL PROTEIN	VI.C
RSp0078	RS05543			-0,69 0,00				TRANSMEMBRANE COG2814, Arabinose efflux permease	IV.D.3
RSp0079	RS05542			-0,69 0,00				TRANSCRIPTION REGULATOR DNA-BINDING TRANSCRIPTIONAL REGULATOR	VI.A
RSp0080	RS05541			-1,81 0,00	-2,24 0,00			COG1733, Predicted transcriptional regulators	VI.A

RSp0081	RS05540		0,60 0,03	-2,21 0,00	-1,11 0,00	1,98 0,00		CONSERVED HYPOTHETICAL PROTEIN	VI.C
RSp0082	RS05556							HYPOTHETICAL PROTEIN	VI.D
RSp0083	RS05555			-0,83 0,00				HYPOTHETICAL PROTEIN	VI.D
RSp0084	RS05553			-0,70 0,04				HYPOTHETICAL PROTEIN	VI.D
RSp0085	RS05538		0,92 0,01	-0,85 0,01				RHS-RELATED PROTEIN	VI.B
RSp0086	RS05537					1,76 0,00		RHS-RELATED PROTEIN	VI.C
RSp0087	RS05535			1,17 0,01	-0,68 0,01			CONSERVED HYPOTHETICAL PROTEIN	VI.C
RSp0088	RS05533							HYPOTHETICAL PROTEIN	VI.D
RSp0089	RS05532			-0,63 0,02				HYPOTHETICAL PROTEIN	VI.D
RSp0090	RS05531							INTEGRASE/RECOMBINASE	V
RSp0091	RS05552				-0,93 0,00			HYPOTHETICAL PROTEIN	VI.D
RSp0092	RS05551				-1,00 0,05			HYPOTHETICAL PROTEIN	VI.D
RSp0093	RS05871		-2,62 0,01	3,45 0,00			TIS1421	TIS1421-transposase ORFB	V.A
RSp0094	RS05870			-0,75 0,00			TIS1421	TIS1421-transposase ORFA	V.A
RSp0095	RS06004			-0,54 0,00				HYPOTHETICAL PROTEIN	VI.D
RSp0096	RS05526							RHS-RELATED PROTEIN	VI.C
RSp0097	RS05527			1,40 0,00		-0,62 0,04		ANKYRIN-REPEAT HARBORING PROTEIN	VI.B
RSp0098	RS05528	-2,97 0,00	-5,01 0,00	2,89 0,00	1,39 0,00	-5,70 0,00		HYPOTHETICAL PROTEIN	VI.D
RSp0099	RS03024	-2,65 0,00	-4,53 0,00	3,06 0,00	3,05 0,00		ripA	TYPE III EFFECTOR PROTEIN AWR1	VI.E
RSp0100	RS03023	-1,01 0,01	0,80 0,03	-2,38 0,00				FERRISIDEROPHORE RECEPTOR PROTEIN	IV.D.5
RSp0101	RS03022			0,30 0,03	1,04 0,00	3,14 0,00		CONSERVED HYPOTHETICAL PROTEIN	VI.C
RSp0102	RS03021			-0,59 0,00				COG3293, Transposase and inactivated derivatives	V.A
RSp0103	RS03020			-0,84 0,00				VGR-RELATED PROTEIN	VI.C
RSp0104	RS03019			-0,90 0,00				TRANSMEMBRANE	VI.C
RSp0105	RS03018			-1,09 0,00				CONSERVED HYPOTHETICAL PROTEIN	VI.C
RSp0106	RS03016						modA	SIGNAL PEPTIDE MOLYBDATE-BINDING PERIPLASMIC PROTEIN	IV.D.1
RSp0107	RS03015			-0,80 0,00	1,32 0,01			TRANSMEMBRANE TRANSPORT PROTEIN	IV.D
RSp0108	RS03014			-0,72 0,00				TRANSCRIPTION REGULATOR	VI.A
RSp0109	RS03013			-0,69 0,03				COG0714, MoxR-like ATPases	VI.C
RSp0110	RS03012		0,70 0,01	-0,49 0,00				TRANSMEMBRANE	VI.C
RSp0111	RS03011							TRANSMEMBRANE	VI.C
RSp0112	RS03010							CARBONIC ANHYDRASE	I.F.18
RSp0113	RS03009		0,46 0,02	-0,39 0,01				TRANSMEMBRANE COG0659, Sulfate permease and related transporters	IV.D
RSp0114	RS03008		0,57 0,04					TRANSMEMBRANE	VI.C
RSp0115	RS03007		0,76 0,04					SIGNAL PEPTIDE	VI.B
RSp0116	RS03006			-1,21 0,01				CONSERVED HYPOTHETICAL PROTEIN	VI.C
RSp0117	RS03005			-1,44 0,05	-0,99 0,04			HYPOTHETICAL PROTEIN	VI.D
RSp0118	RS03004			-0,49 0,00		-0,54 0,04		HYPOTHETICAL PROTEIN	VI.D
RSp0119	RS03003							TRANSMEMBRANE	VI.C
RSp0120	RS03002						acnA2	TRANSMEMBRANE ACONITATE HYDRATASE 1	I.H.6
RSp0121	RS03001			-0,16 0,05			prpC	METHYLCITRATE SYNTHASE (CITRATE SYNTHASE 2)	I
RSp0122	RS03000						prpB2	CARBOXYVINYL-CARBOXYPHOSPHONATE PHOSPHORYLMUTASE	I.I.4
RSp0123	RS02999						prpR	TRANSCRIPTION REGULATOR PROPIONATE CATABOLISM OPERON REGULAT(	I
RSp0124	RS02998		0,66 0,00	-0,43 0,00				TRANSMEMBRANE LIPOPROTEIN	VI.C
RSp0125	RS02997			-0,52 0,00		-0,82 0,01		TRANSMEMBRANE PROLIN RICH PROTEIN	VI.D
RSp0127	RS02994			-0,63 0,01			tISRso1	ISRso1 TRANSPOSASE	V.A
RSp0128	RS02993		-0,79 0,05	1,19 0,00				COG0210, Superfamily I DNA and RNA helicases	II.B.8
RSp0129	RS02991							HYPOTHETICAL PROTEIN	VI.D
RSp0130	RS02992			-0,66 0,00		0,98 0,00		HYPOTHETICAL PROTEIN	VI.B
RSp0131	RS02990							HYPOTHETICAL PROTEIN	VI.D
RSp0132	RS02988		-0,71 0,00					TRANSMEMBRANE SIGNAL TRANSDUCTION PROTEIN GGDEF DOMAIN	VI.A
RSp0133	RS02987			-0,43 0,03				TRANSMEMBRANE	VI.C
RSp0134	RS02986							TRANSMEMBRANE	VI.C
RSp0135	RS02985		-0,66 0,04	-0,88 0,02				TRANSMEMBRANE COG4257 STREPTOGRAMIN LYASE	IV.F
RSp0136	RS02984							OXIDOREDUCTASE COG1902, NADH:flavin oxidoreductases	I
RSp0137	RS02983			-1,10 0,01	-1,59 0,00			TRANSCRIPTION REGULATOR	VI.A
RSp0138	RS02982		0,44 0,03	-0,36 0,01	1,43 0,01		pme	TRANSMEMBRANE PECTINESTERASE (PECTIN METHYLESTERASE)	II.A.3
RSp0139	RS02981			-0,46 0,00				COG1246, N-acetylglutamate synthase and related acetyltransferases	I
RSp0140	RS02980							TRANSMEMBRANE COG2165, Type II secretory pathway, pseudopilin	IV.E.2
RSp0141	RS02979							TRANSMEMBRANE GENERAL SECRETION PATHWAY GSPG RELATED PROTEIN	IV.E.2
RSp0142	RS02978							TRANSMEMBRANE GENERAL SECRETION PATHWAY GSPG RELATED PROTEIN	IV.E.2
RSp0143	RS02977							GSPD-RELATED PROTEIN	IV.E.2
RSp0144	RS02976							TRANSMEMBRANE PROLIN-RICH PROTEIN	VI.C
RSp0145	RS02975							TRANSMEMBRANE	VI.C
RSp0146	RS02974		0,46 0,05					TRANSMEMBRANE	VI.C
RSp0147	RS02973				-2,02 0,00			HYPOTHETICAL PROTEIN	VI.D
RSp0148	RS02972		1,22 0,01	-0,45 0,04				GENERAL SECRETION PATHWAY GSPE-RELATED PROTEIN	IV.E.2
RSp0149	RS02971		0,88 0,02					TRANSMEMBRANE GENERAL SECRETION PATHWAY GSPF-RELATED PROTEIN	IV.E.2
RSp0150	RS02970				-2,05 0,01			TRANSMEMBRANE GENERAL SECRETION PATHWAY GSPG-RELATED PROTEIN	IV.E.2
RSp0151	RS02969							RHS-RELATED PROTEIN	VI.B
RSp0152	RS02967							HYPOTHETICAL PROTEIN	VI.D
RSp0153	RS06008							HYPOTHETICAL PROTEIN	VI.D
RSp0154	RS06009							TRANSMEMBRANE	VI.D
RSp0157	RS05520							HYPOTHETICAL PROTEIN	VI.B
RSp0158	RS06026		-1,08 0,00	1,45 0,00				HYPOTHETICAL PROTEIN	VI.D
RSp0159	RS06025		-3,70 0,00	4,47 0,00	1,39 0,04			HYPOTHETICAL PROTEIN	VI.D
RSp0160	RS05518		-3,62 0,00	4,13 0,00	4,62 0,00			CONSERVED HYPOTHETICAL PROTEIN	VI.C
RSp0161	RS05517					4,17 0,00		TRANSMEMBRANE	VI.C
RSp0162	RS05516			0,63 0,00	2,59 0,04	4,20 0,00	egl	ENDOGLUCANASE PRECURSOR (ENDO-1,4-BETA-GLUCANASE)	II.A.3
RSp0163	RS05515			0,64 0,01				PHOSPHOPANTETHEINYL TRANSFERASE	I
RSp0164	RS05514		-1,66 0,03	1,26 0,01				TRANSMEMBRANE COG0534, Na+-driven multidrug efflux pump	IV.G
RSp0165	RS05513							CONSERVED HYPOTHETICAL PROTEIN	VI.C
RSp0166	RS05511						cysC2	ADENYLYLSULFATE KINASE	I.F.3
RSp0167	RS04679							SULFOTRANSFERASE	I
RSp0168	RS04680			-0,44 0,04				HYPOTHETICAL PROTEIN	VI.D
RSp0169	RS04681					0,90 0,00		TRANSMEMBRANE	VI.D
RSp0170	RS04682			-1,53 0,03				HYPOTHETICAL PROTEIN	VI.D
RSp0171	RS04683				0,59 0,00			HYPOTHETICAL PROTEIN	VI.B
RSp0172	RS04684			0,50 0,00	0,63 0,01	1,95 0,00		CONSERVED HYPOTHETICAL PROTEIN	VI.C
RSp0173	RS04685				0,74 0,03	1,99 0,02		CYSTEINE DESULFURASE (tRNA sulfurtransferase)	II.B.6
RSp0174	RS04686			0,38 0,01	1,09 0,01			COG0500, SAM-dependent methyltransferases	I
RSp0175	RS04687		0,44 0,04	-0,89 0,00	-1,55 0,00	-2,12 0,00		VGR-RELATED PROTEIN	VI.C
RSp0176	RS04688		0,56 0,00	-1,39 0,00	-2,34 0,00			CONSERVED HYPOTHETICAL PROTEIN	VI.C
RSp0177	RS04689		-1,17 0,00	1,94 0,00	-0,55 0,03			HYPOTHETICAL PROTEIN	VI.B
RSp0178	RS04690		0,62 0,02	-0,92 0,00	-1,16 0,02	-2,74 0,00		HYPOTHETICAL PROTEIN	VI.D
RSp0179	RS04691			-0,72 0,00	-0,93 0,04	-0,92 0,01		CONJUGAL TRANSFERT TRBI-RELATED PROTEIN	VI.B
RSp0180	RS04692							CONSERVED HYPOTHETICAL PROTEIN	VI.C
RSp0181	RS04693							CONSERVED HYPOTHETICAL PROTEIN	VI.C
RSp0182	RS04694							TRANSMEMBRANE TRANSPORTER	IV.D
RSp0183	RS04695							3-CARBOXYMUCONATE CYCLASE	I
RSp0184	RS04696			-0,25 0,00		0,83 0,00		HYPOTHETICAL PROTEIN	VI.D
RSp0185	RS04697			-0,60 0,04				TRANSCRIPTION REGULATOR	VI.A
RSp0186	RS04698							ABC TRANSPORTER COG0834: ABC-type amino acid transport/signal transduction s	IV.D
RSp0187	RS04699							HYPOTHETICAL PROTEIN	VI.D
RSp0188	RS04700				-0,42 0,02			PSEUDOGENE, OXYDOREDUCTASE (FRAGMENT)	VI.B
RSp0189	RS04701							TRANSMEMBRANE TYPE IV PILUS ASSEMBLY FimV-RELATED PROTEIN	III.A.2
RSp0190	RS04702			-0,55 0,05				TRANSMEMBRANE COG0730: Predicted permeases	IV.D
RSp0191	RS04703			-0,54 0,01				HYPOTHETICAL PROTEIN	VI.D
RSp0192	RS04705							TRANSMEMBRANE	VI.C
RSp0193	RS04706			2,25 0,00	3,30 0,00			PPR REPEATS CONTAINING PROTEIN	VI.B
RSp0194	RS04707			-0,53 0,02				COG0332, 3-oxoacyl-[acyl-carrier-protein]	I
RSp0195	RS04708							HYPOTHETICAL PROTEIN	VI.D

RSp0196	RS04709			-0,44 0,02				PROLYL AMINOPEPTIDASE	II.A.4
RSp0197	RS04710			-0,46 0,03				CHORISMATE MUTASE	I
RSp0198	RS04711	-2,41 0,00		0,95 0,00	1,48 0,00	1,89 0,00		TRANSMEMBRANE	VI.D
RSp0199	RS04712			0,91 0,00	2,18 0,00			TRANSMEMBRANE	VI.C
RSp0200	RS04713			0,75 0,03	2,61 0,00	3,23 0,00		OXIDOREDUCTASE PROTEIN WITH COG5322, Predicted dehydrogenase AND CO(	I
RSp0201	RS04714					5,17 0,00		CONSERVED HYPOTHETICAL PROTEIN	VI.C
RSp0202	RS05227				-1,27 0,03			CONSERVED HYPOTHETICAL PROTEIN	VI.C
RSp0203	RS05225			-0,39 0,00				COG1607, Acyl-CoA hydrolase	I
RSp0204	RS05224		1,16 0,02	-0,51 0,05				CONSERVED HYPOTHETICAL PROTEIN	VI.C
RSp0205	RS05223			-0,69 0,02	-0,54 0,01			PSEUDOGENE (C-TERMINAL FRAGMENT)	VI.C
RSp0207	RS05221							SIGNAL PEPTIDE PSEUDOGENE(N-TERMINAL FRAGMENT)	VI.C
RSp0208	RS05220	-0,92 0,03		-1,39 0,01	-3,46 0,00			CONSERVED HYPOTHETICAL PROTEIN	VI.C
RSp0209	RS05217							HYDROXYLASE	I.I
RSp0210	RS05216			-1,31 0,00	-1,85 0,01			HYPOTHETICAL PROTEIN	VI.D
RSp0211	RS05215							SIGNAL PEPTIDE	VI.C
RSp0212	RS05214							SIGNAL PEPTIDE COG3670, Lignostilbene-alpha,beta-dioxygenase and related enzy	I
RSp0213	RS05218	-3,99 0,00	-6,59 0,00	7,29 0,00	4,58 0,00	-3,66 0,00		HYPOTHETICAL PROTEIN	VI.B
RSp0214	RS05213						amn	AMP NUCLEOSIDASE	I.F.1
RSp0215	RS05212		-1,80 0,00	3,53 0,00	3,36 0,00			CONSERVED HYPOTHETICAL PROTEIN	VI.C
RSp0216	RS05210	-3,31 0,00	-4,03 0,00	4,96 0,00	3,15 0,00			SERINE/THREONINE-PROTEIN KINASE	VI.B
RSp0217	RS05209	0,30 0,04		-0,42 0,05		-0,31 0,04	tISRso5	ISRso5-TRANSPOSASE	V.A
RSp0218	RS05208		-2,72 0,01	4,68 0,00	4,86 0,04			CONSERVED HYPOTHETICAL PROTEIN	VI.B
RSp0219	RS05205			0,80 0,00	-1,43 0,04		ncnH	TRANSMEMBRANE Naphthocyclinone hydroxylase	IV.G.3
RSp0220	RS05203			0,40 0,02				TRANSCRIPTION REGULATOR TWO-COMPONENT TRANSMEMBRANE SENSOR	VI.A
RSp0221	RS05202			-0,35 0,00				TRANSCRIPTION REGULATOR	VI.A
RSp0222	RS05201		0,92 0,01				vanA	OXIDOREDUCTASE VANILLATE O-DEMETHYLASE OXYGENASE SUBUNIT	I.I.4
RSp0223	RS05200						vanB	OXIDOREDUCTASE VANILLATE O-DEMETHYLASE (VANILLATE DEGRADATION F	I.I.4
RSp0224	RS05199		-0,84 0,03					TRANSCRIPTION REGULATOR	VI.A
RSp0225	RS05198						fca	FERULIC ACID HYDRATASE	I.I.4
RSp0226	RS05197	0,41 0,02		-0,49 0,04			vdh	OXIDOREDUCTASE VANILLIN DEHYDROGENASE	I.I.4
RSp0227	RS05196						fcs	FERULOYL-COA SYNTHETASE	I.I.4
RSp0228	RS05195							TRANSCRIPTION REGULATOR PHENOL-DEGRADATIVE GENE REGULATOR	IV.F.1
RSp0229	RS05194			-0,72 0,00	-0,98 0,04			OXIDOREDUCTASE BENZALDEHYDE DEHYDROGENASE	IV.F.1
RSp0230	RS05193							OXIDOREDUCTASE 3-ALPHA-HYDROXYSTEROID DEHYDROGENASE/CARBONY	I.I
RSp0231	RS05192							SIGNAL PEPTIDE COG4313, Protein involved in meta-pathway of phenol degradation	I.I
RSp0232	RS05191							ESTERASE/LIPASE	I.I.1
RSp0233	RS05190							TRANSMEMBRANE HYDROLASE	I.I
RSp0234	RS05189			-0,37 0,00				TRANSMEMBRANE METABOLITE TRANSPORT PROTEIN	IV.D
RSp0235	RS05188						treY	TRANSMEMBRANE MALTOOLIGOSYL TREHALOSE SYNTHASE	II.B.5
RSp0236	RS05187			-0,67 0,01			malQ	4-ALPHA-GLUCANOTRANSFERASE (AMYLOMALTASE)	II.A.3
RSp0237	RS05186				-0,37 0,02			COG0296, 1,4-alpha-glucan branching enzyme	II.B.5
RSp0238	RS05185							COG1523, PulA pullulanase and related glycosidases	II.B.5
RSp0239	RS05184			0,47 0,05	-0,84 0,01		glgB	1,4-ALPHA-GLUCAN BRANCHING ENZYME	II.B.5
RSp0240	RS05183			-0,91 0,00	-1,36 0,01			PROTEIN WITH COG0366, Glycosidases AND COG3281, Uncharacterized protein, p	II.A.3
RSp0241	RS05182			-1,01 0,00				ALPHA-AMYLASE-RELATED PROTEIN	II.A.3
RSp0242	RS03725						glgA	GLYCOGEN SYNTHASE	II.B.5
RSp0243	RS03723					-1,83 0,01		HEMIN TRANSPORT PROTEIN	IV.D.5
RSp0244	RS03722	-0,86 0,05		-0,77 0,00	-1,10 0,05	-3,14 0,01		SIGNAL PEPTIDE OUTER MEMBRANE HEMIN RECEPTOR	IV.D.5
RSp0245	RS03721			-0,86 0,00			ahpC1	OXIDOREDUCTASE ALKYL HYDROPEROXIDE REDUCTASE (SUBUNIT C)	IV.G.4
RSp0246	RS03720					-1,58 0,00	ahpF	OXIDOREDUCTASE ALKYL HYDROPEROXIDE REDUCTASE (SUBUNIT F)	IV.G.4
RSp0247	RS03719			-1,67 0,00		-1,70 0,00	fur2	TRANSCRIPTION REGULATOR FERRIC UPTAKE TRANSCRIPTIONAL (FUR)- REL	I.E.1
RSp0248	RS03718			-1,61 0,00	-0,84 0,03			HYPOTHETICAL PROTEIN	VI.D
RSp0249	RS03717			-0,36 0,02		0,68 0,05		CONSERVED HYPOTHETICAL PROTEIN	VI.C
RSp0250	RS03716				-0,90 0,01	-1,62 0,04		TRANSMEMBRANE	VI.C
RSp0251	RS03715			-0,55 0,00		-1,68 0,00	feoB	TRANSMEMBRANE FERROUS IRON TRANSPORT PROTEIN B	IV.D.5
RSp0252	RS03714			-0,91 0,04	-0,78 0,01	-0,74 0,00	feoA	COG1918, Fe2+ transport system protein A	IV.D.5
RSp0253	RS03713							TRANSMEMBRANE SIGNAL TRANSDUCTION PROTEIN	VI.A
RSp0254	RS03712			-0,32 0,03	-0,72 0,04			TRANSMEMBRANE SIGNAL TRANSDUCTION PROTEIN EAL - GGDEF DOMAINS	VI.A
RSp0255	RS03711							TRANSMEMBRANE METHYL-ACCEPTING CHEMOTAXIS TRANSDUCER	IV.C
RSp0256	RS03710							HYPOTHETICAL PROTEIN	VI.D
RSp0257	RS03709			-0,67 0,01	-1,49 0,00		viuB	COG2375, Siderophore-interacting protein	IV.D.5
RSp0258	RS03708			-3,16 0,00	-3,14 0,00			TRANSCRIPTION REGULATOR	VI.A
RSp0259	RS03707						pcp2	PYRROLIDONE-CARBOXYLATE PEPTIDASE	I.F.14
RSp0260	RS03706		1,16 0,00	-1,18 0,00	-1,78 0,00			TRANSCRIPTION REGULATOR	VI.A
RSp0261	RS03705		-0,66 0,03	0,56 0,00	1,28 0,00	1,47 0,00		TRANSMEMBRANE	VI.D
RSp0262	RS03704		0,69 0,00	-0,28 0,01				TRANSMEMBRANE COG3131 Periplasmic glucans biosynthesis protein	IV.F.2
RSp0263	RS03702							CONSERVED HYPOTHETICAL PROTEIN	VI.C
RSp0264	RS03703							CONSERVED HYPOTHETICAL PROTEIN	VI.C
RSp0265	RS03701							COG1670, Acetyltransferases, including N-acetylases of ribosomal proteins	VI.C
RSp0266	RS03700							CONSERVED HYPOTHETICAL PROTEIN	VI.C
RSp0267	RS03699			-0,44 0,00	-0,45 0,04			CONSERVED HYPOTHETICAL PROTEIN	VI.C
RSp0268	RS03698			-0,50 0,00				HYPOTHETICAL PROTEIN	VI.D
RSp0269	RS03695		0,80 0,05	-0,18 0,01		-0,38 0,00		CONSERVED HYPOTHETICAL PROTEIN	VI.C
RSp0270	RS03694					-0,32 0,01		HYPOTHETICAL PROTEIN	VI.B
RSp0271	RS03693					-0,48 0,01		HYPOTHETICAL PROTEIN	VI.B
RSp0272	RS03692				0,69 0,02	-0,78 0,02		CONSERVED HYPOTHETICAL PROTEIN	VI.C
RSp0273	RS03691			-0,44 0,02				COG0624, Acetylornithine deacetylase/Succinyl-diaminopimelate desuccinylase and r	I
RSp0274	RS03690						ggt1	SIGNAL PEPTIDE GAMMA-GLUTAMYLTRANSPEPTIDASE	I.B.6
RSp0275	RS03689		1,03 0,02	-0,42 0,00	1,49 0,00	4,76 0,00		TRANSMEMBRANE Glycosyl hydrolase family 18 protein with carbohydrate binding d(	II.A.3
RSp0276	RS03688		-1,44 0,04	1,15 0,01			cysK1	CYSTEINE SYNTHASE A	I.A.9
RSp0277	RS03687			-1,15 0,00	-0,86 0,05		treA	SIGNAL PEPTIDE PERIPLASMIC ALPHA,ALPHA-TREHALASE	IV.F.2
RSp0278	RS03686					1,51 0,00		CONSERVED HYPOTHETICAL PROTEIN	VI.C
RSp0279	RS03685		0,92 0,02					SIGNAL PEPTIDE COG0823, Periplasmic component of the Tol transport system TolE	IV.E
RSp0280	RS03684							HYPOTHETICAL PROTEIN	VI.D
RSp0281	RS03683			-0,59 0,00				SIGNAL TRANSDUCTION PROTEIN GGDEF DOMAIN	VI.A
RSp0282	RS03682		0,74 0,04					TRANSMEMBRANE AMINO-ACID PERMEASE	IV.D.2
RSp0283	RS03681						dinF	TRANSMEMBRANE DNA-DAMAGE-INDUCIBLE PROTEIN F	IV.D
RSp0284	RS03680							TRANSMEMBRANE	VI.D
RSp0285	RS03679			-0,62 0,04	-2,72 0,00			TRANSCRIPTION REGULATOR	VI.A
RSp0286	RS03678		-2,83 0,01	3,28 0,00	1,71 0,01		hmsH	TRANSMEMBRANE HEMIN-BINDING OUTER MEMBRANE PROTEIN	IV.D.5
RSp0287	RS03677	-0,91 0,04					hmsF	SIGNAL PEPTIDE HEMIN STORAGE PROTEIN	IV.D.5
RSp0288	RS03676		-0,56 0,05				hmsR	TRANSMEMBRANE HEMIN STORAGE PROTEIN	IV.D.5
RSp0289	RS03675	-0,55 0,05		-0,49 0,01		0,55 0,04		TRANSMEMBRANE	VI.B
RSp0290	RS03674							TYPE I SECRETION SYSTEM, OUTER MEMBRANE CHANEL LIPOPROTEIN	IV.E.2
RSp0291	RS03671							TRANSMEMBRANE TYPE I SECRETION SYSTEM PROTEIN	IV.E.2
RSp0292	RS03669		-1,02 0,01	1,27 0,00				ABC TRANSPORTER TYPE I SECRETION SYSTEM, ATP-BINDING TRANSMEMBR	IV.E.2
RSp0293	RS03673			-0,45 0,00		0,92 0,01		TRANSMEMBRANE	VI.D
RSp0294	RS03672					2,62 0,01		HEMOLYSIN-TYPE CALCIUM-BINDING PROTEIN	IV.G.3
RSp0295	RS03668		1,02 0,01	-0,36 0,02				HEMOLYSIN-TYPE PROTEIN	IV.G.3
RSp0296	RS05479							SKWP PROTEIN 5	VI.C
RSp0297	RS05477							HYPOTHETICAL PROTEIN	VI.D
RSp0298	RS05476		0,62 0,00	-1,55 0,00	-1,79 0,00			HYPOTHETICAL PROTEIN	VI.D
RSp0299	RS05475					-0,31 0,04		TRANSMEMBRANE HYPOTHETICAL	VI.D
RSp0300	RS05473							ACYLTRANSFERASE	VI.C
RSp0303	RS05471						cheD2	TRANSMEMBRANE METHYL-ACCEPTING CHEMOTAXIS PROTEIN	IV.C
RSp0304	RS05468	-3,33 0,00	-7,30 0,00	7,94 0,00	4,72 0,00	-4,80 0,00		AVRPPHD-RELATED PROTEIN	VI.C
RSp0305	RS05466							OXIDOREDUCTASE DEHYDROGENASE	I.I
RSp0306	RS05465							CONSERVED HYPOTHETICAL PROTEIN	VI.C
RSp0307	RS05464							OXIDOREDUCTASE	I.I
RSp0308	RS05463							TRANSCRIPTION REGULATOR	VI.A
RSp0309	RS05461			-0,21 0,03	-0,69 0,00	-0,51 0,00		TRANSCRIPTION REGULATOR	VI.A
RSp0310	RS05460							TRANSMEMBRANE MACROLIDE-EFFLUX PROTEIN	IV.D

RSp0311	RS05459						oprM	OUTER MEMBRANE CHANEL LIPOPROTEIN	IV.D
RSp0312	RS05457						mexD	TRANSMEMBRANE MULTIDRUG EFFLUX SYSTEM PROTEIN	IV.D
RSp0313	RS05456						mexC	MULTIDRUG EFFLUX SYSTEM PROTEIN	IV.G.2
RSp0314	RS05454			-0,64 0,01				TRANSCRIPTION REGULATOR TWO COMPONENT RESPONSE REGULATOR	VI.A
RSp0315	RS05453			-0,66 0,02				TRANSCRIPTION REGULATOR TRANSMEMBRANE SENSOR KINASE	VI.A
RSp0316	RS05452							OXIDOREDUCTASE COG2070, Dioxygenase related to 2-nitropropane dioxygenase	I
RSp0317	RS05451				-1,78 0,00			CONSERVED HYPOTHETICAL PROTEIN	VI.C
RSp0318	RS05449			-0,47 0,00				COG0589, Universal stress protein UspA and related nucleotide-binding proteins	VI.C
RSp0319	RS05448							TRANSMEMBRANE METAL-TRANSPORTING P-TYPE ATPASE	IV.F.1
RSp0320	RS05447							TRANSCRIPTION REGULATOR	VI.A
RSp0321	RS05446							CYSTEINE-RICH PROTEIN	VI.C
RSp0322	RS05445			0,68 0,00		-1,62 0,00	leuA2	2-ISOPROPYLMALATE SYNTHASE	I.A.1
RSp0323	RS05444		-2,47 0,00	3,52 0,00	2,71 0,00			CONSERVED HYPOTHETICAL PROTEIN	VI.C
RSp0324	RS05442			-1,38 0,00				OXIDOREDUCTASE DEHYDROGENASE, SMALL CHAIN	I.I
RSp0325	RS05441							OXIDOREDUCTASE DEHYDROGENASE, LARGE CHAIN	I.I
RSp0326	RS05440							ORGANIC HYDROPEROXIDE RESISTANCE PROTEIN	IV.G.4
RSp0327	RS05439							CONSERVED HYPOTHETICAL PROTEIN	VI.C
RSp0328	RS05438		0,37 0,02	-0,36 0,00				TRANSCRIPTION REGULATOR	VI.A
RSp0329	RS05429						leuB2	OXIDOREDUCTASE 3-ISOPROPYLMALATE DEHYDROGENASE	I.A.1
RSp0330	RS00703			-0,50 0,00	-0,65 0,01		acnA3	ACONITATE HYDRATASE 1	I.H.6
RSp0331	RS00705							TRANSCRIPTION REGULATOR	VI.A
RSp0332	RS00707					-2,35 0,00	acnB	TRANSMEMBRANE ACONITATE HYDRATASE 2	I.H.6
RSp0333	RS00709		-0,73 0,01	-0,39 0,02		1,77 0,00	osmB2	TRANSMEMBRANE OSMOTICALLY INDUCIBLE LIPOPROTEIN B2	IV.F.2
RSp0334	RS00710			-0,87 0,00				SIGNAL TRANSDUCTION PROTEIN EAL - GGDEF DOMAINS	VI.A
RSp0335	RS00711			-0,92 0,00	-1,18 0,01			TRANSCRIPTION REGULATOR HNS-LIKE PROTEIN	VI.A
RSp0336	RS00713		0,59 0,01		-0,57 0,03	-1,03 0,00		TRANSCRIPTION REGULATOR	VI.A
RSp0337	RS00714		0,55 0,04	-0,28 0,00	-0,74 0,01	-1,23 0,00		TRANSMEMBRANE PORIN PRECURSOR	IV
RSp0338	RS00717		0,27 0,04	-1,71 0,00	0,97 0,00	2,37 0,00	epsR	TRANSCRIPTION REGULATOR NEGATIVE REGULATOR OF EXOPOLYSACCHAR	III.A.1
RSp0339	RS00719	-0,96 0,02					flgN	FLAGELLA SYNTHESIS PROTEIN FLGN	IV.C
RSp0340	RS00833		0,38 0,05	-0,58 0,03			flgM	NEGATIVE REGULATOR OF FLAGELLIN SYNTHESIS (ANTI-SIGMA-28 FACTOR)	IV.C
RSp0341	RS00720						flgA	FLAGELLA BASAL BODY P-RING FORMATION PROTEIN	IV.C
RSp0342	RS00738			-0,80 0,00			flgB	FLAGELLAR BASAL-BODY ROD PROTEIN FLGB	IV.C
RSp0343	RS00739			-1,05 0,00			flgC	FLAGELLAR BASAL-BODY ROD PROTEIN FLGC	IV.C
RSp0344	RS00744			-0,80 0,00			flgD	BASAL-BODY ROD MODIFICATION PROTEIN FLGD	IV.C
RSp0345	RS00745		1,05 0,01	-0,84 0,00			flgE	FLAGELLAR HOOK PROTEIN FLGE	IV.C
RSp0346	RS00747			-0,94 0,00			flgF	FLAGELLAR BASAL-BODY ROD PROTEIN FLGF	IV.C
RSp0347	RS00748		1,11 0,04	-0,70 0,00			flgG	FLAGELLAR BASAL-BODY ROD PROTEIN FLGG (DISTAL ROD PROTEIN)	IV.C
RSp0348	RS00749		1,11 0,00	-0,59 0,03			flgH	FLAGELLAR L-RING PROTEIN PRECURSOR	IV.C
RSp0349	RS00751						flgI	SIGNAL PEPTIDE FLAGELLAR P-RING PROTEIN PRECURSOR	IV.C
RSp0350	RS00752			-0,41 0,02			flgJ	FLAGELLAR PROTEIN FLGJ	IV.C
RSp0351	RS00754						flgK	FLAGELLAR HOOK-ASSOCIATED PROTEIN 1	IV.C
RSp0352	RS00756						flgL	FLAGELLAR HOOK-ASSOCIATED PROTEIN 3	IV.C
RSp0353	RS00757			1,17 0,00				TRANSMEMBRANE	VI.C
RSp0354	RS00759		0,63 0,00		-1,18 0,05			OXIDOREDUCTASE COG0644, Dehydrogenase (flavoproteins)	I
RSp0355	RS00761		0,98 0,01			-1,43 0,00		METHYLASE	VI.C
RSp0356	RS00762		2,12 0,02					SIGNAL PEPTIDE	VI.C
RSp0357	RS00765						fabB	3-OXOACYL-[ACYL-CARRIER-PROTEIN] SYNTHASE I	I.B.1
RSp0358	RS00767						fabF2	3-OXOACYL-[ACYL-CARRIER-PROTEIN] SYNTHASE II	I.B.1
RSp0359	RS00769						fabG2	OXIDOREDUCTASE 3-OXOACYL-[ACYL-CARRIER-PROTEIN] REDUCTASE	I.B.1
RSp0360	RS00772							OXIDOREDUCTASE COG4706, 3-hydroxylacyl-(acyl carrier protein) dehydratase	I
RSp0361	RS00773			-0,70 0,04				3-OXOACYL-[ACYL-CARRIER-PROTEIN] SYNTHASE	I.B.1
RSp0362	RS00775				-1,04 0,02			TRANSMEMBRANE COG4258, Predicted exporter	IV.D
RSp0363	RS00776							SIGNAL PEPTIDE	VI.C
RSp0364	RS00778							COG0824, Thioesterase	I
RSp0365	RS00779						hutH2	HISTIDINE AMMONIA-LYASE	I.I.3
RSp0366	RS00785							TRANSMEMBRANE COG0463, Glycosyltransferase AND COG4261, Predicted acyltra	I
RSp0367	RS00787			-0,48 0,00				COG0365, Acyl-coenzyme A synthetases/AMP-(fatty) acid ligases AND COG0764, 3-	I
RSp0368	RS00790			-0,44 0,01				TRANSMEMBRANE	VI.C
RSp0369	RS00792			-0,88 0,01				ACYL-CARRIER PROTEIN	I
RSp0370	RS00794			-0,38 0,00				ACYL-CARRIER PROTEIN	I
RSp0371	RS00796			-0,59 0,03				TRANSMEMBRANE 1-ACYL-SN-GLYCEROL-3-PHOSPHATE ACYLTRANSFERASE	II.B.1
RSp0372	RS00799			-0,65 0,00				CONSERVED HYPOTHETICAL PROTEIN	VI.C
RSp0373	RS00800						fliR	TRANSMEMBRANE FLAGELLAR BIOSYNTHETIC PROTEIN FLIR	IV.C
RSp0374	RS00802						fliQ	TRANSMEMBRANE FLAGELLAR BIOSYNTHETIC PROTEIN FLIQ	IV.C
RSp0375	RS00803						fliP	TRANSMEMBRANE FLAGELLAR BIOSYNTHETIC PROTEIN FLIP	IV.C
RSp0376	RS00804						fliO	TRANSMEMBRANE FLAGELLAR PROTEIN FLIO	IV.C
RSp0377	RS00805			-1,05 0,00			fliN	FLAGELLAR MOTOR SWITCH PROTEIN FLIN	IV.C
RSp0378	RS00806		1,00 0,05	-0,40 0,00			fliM	FLAGELLAR MOTOR SWITCH PROTEIN FLIM	IV.C
RSp0379	RS00807		0,77 0,03				fliL	TRANSMEMBRANE FLAGELLAR FLIL PROTEIN	IV.C
RSp0380	RS00808							SIGNAL TRANSDUCTION PROTEIN EAL - GGDEF DOMAINS	VI.A
RSp0381	RS00809			-0,34 0,03				TRANSCRIPTION REGULATOR	VI.A
RSp0382	RS00810			-0,49 0,00	-2,58 0,00	2,46 0,00	fliC	FLAGELLIN	IV.C
RSp0383	RS00811		1,00 0,02				fliD	FLAGELLAR HOOK-ASSOCIATED PROTEIN 2 (FILAMENT CAP PROTEIN)	IV.C
RSp0384	RS00812						fliS	FLAGELLAR PROTEIN FLIS	IV.C
RSp0385	RS00813			-0,33 0,02			fliT	FLAGELLAR PROTEIN FLIT	IV.C
RSp0386	RS00814			-0,56 0,01				TRANSCRIPTION REGULATOR	VI.A
RSp0387	RS00815				-1,86 0,05			CONSERVED HYPOTHETICAL PROTEIN	VI.C
RSp0388	RS00816							TRANSMEMBRANE COG3774, Mannosyltransferase OCH1 and related enzymes	II.B
RSp0389	RS00817			-0,75 0,00	-0,51 0,02		fliE	FLAGELLAR HOOK-BASAL BODY COMPLEX PROTEIN FLIE	IV.C
RSp0390	RS00818			-0,68 0,02			fliF	TRANSMEMBRANE FLAGELLAR M-RING PROTEIN	IV.C
RSp0391	RS00819						fliG	FLAGELLAR MOTOR SWITCH PROTEIN FLIG	IV.C
RSp0392	RS00820						fliH	FLAGELLAR ASSEMBLY PROTEIN FLIH	IV.C
RSp0393	RS00821						fliI	FLAGELLUM-SPECIFIC ATP SYNTHASE	IV.C
RSp0394	RS00822		1,60 0,04				fliJ	FLAGELLAR PROTEIN	IV.C
RSp0395	RS00823		1,05 0,05	-0,67 0,05			fliK	FLAGELLAR HOOK-LENGTH CONTROL PROTEIN	IV.C
RSp0396	RS00824							TRANSMEMBRANE	VI.C
RSp0397	RS00825			-0,79 0,04			pat	PHOSPHINOTHRICIN ACETYLTRANSFERASE	IV.G.4
RSp0398	RS00826							TRANSMEMBRANE SIGNAL TRANSDUCTION PROTEIN GGDEF DOMAIN	VI.A
RSp0399	RS00827			-0,79 0,00				HYPOTHETICAL PROTEIN	VI.D
RSp0400	RS00828			0,59 0,00				TRANSMEMBRANE SIGNAL TRANSDUCTION PROTEIN GGDEF DOMAIN	VI.A
RSp0401	RS00829			-0,46 0,01				LIPOPROTEIN	VI.D
RSp0402	RS00830							TRANSMEMBRANE	VI.C
RSp0403	RS00831				-0,35 0,05			SIGNAL PEPTIDE	VI.C
RSp0404	RS00861			-0,45 0,02				TRANSMEMBRANE	VI.B
RSp0405	RS00862			-0,88 0,00	1,66 0,00	2,68 0,00		HYPOTHETICAL PROTEIN	VI.D
RSp0406	RS00863			-0,90 0,00	1,48 0,01	3,32 0,00		HYPOTHETICAL PROTEIN	VI.D
RSp0407	RS00864		-1,09 0,01	-1,68 0,00		1,43 0,02		TRANSMEMBRANE	VI.D
RSp0408	RS00865				-0,67 0,03			HYPOTHETICAL PROTEIN	VI.D
RSp0409	RS00866			-1,11 0,00				TRANSMEMBRANE	VI.D
RSp0410	RS00867			-0,77 0,00				TRANSMEMBRANE	VI.C
RSp0411	RS00868			-0,63 0,05				TRANSMEMBRANE COG0628, Permease	IV.D
RSp0412	RS00869							TRANSMEMBRANE COG0477, Permeases of the major facilitator superfamily	IV.D
RSp0413	RS00870			-2,78 0,00	-0,93 0,03	-3,20 0,00		CONSERVED HYPOTHETICAL PROTEIN	VI.C
RSp0414	RS00871			-2,44 0,00	-0,53 0,03	-3,59 0,00		FERRIC SIDEROPHORE RECEPTOR	IV.D.5
RSp0415	RS00872			-1,79 0,00				TRANSCRIPTION REGULATOR EXTRACYTOPLASMIC FUNCTION SIGMA FACTO	I.E.1
RSp0416	RS00873		0,65 0,01	-1,99 0,00		-0,57 0,00		SIGNAL PEPTIDE FERRIC SIDEROPHORE RECEPTOR OUTER MEMBRANE PRO	IV.D.5
RSp0417	RS00874			-3,64 0,00		-0,99 0,01		CYSTEINE SYNTHASE	IV.D.5
RSp0418	RS00875			-3,87 0,00				ORNITHINE CYCLODEAMINASE	IV.D.5
RSp0419	RS00876		0,54 0,04	-2,86 0,00		-1,28 0,00		Siderophore synthetase component	IV.D.5
RSp0420	RS00877			-3,68 0,00				TRANSMEMBRANE EFFLUX PROTEIN	IV.D.5
RSp0421	RS00878			-3,39 0,00		-1,05 0,04		Siderophore synthetase component	IV.D.5
RSp0422	RS00879			-3,96 0,00				Siderophore synthetase component	IV.D.5

RSp0423	RS00880	-0,66	0,05			-3,63	0,00						COG3836, 2,4-dihydroxyhept-2-ene-1,7-dioic acid aldolase	IV.D.5
RSp0424	RS00881					-3,87	0,00			-0,89	0,03		DIAMINOPIMELATE DECARBOXYLASE	IV.D.5
RSp0425	RS00882					-0,72	0,00						COG1926, Phosphoribosyltransferase	I
RSp0426	RS00883												TRANSMEMBRANE	VI.C
RSp0427	RS00884					-1,36	0,00	-1,34	0,00	-0,72	0,00		CONSERVED HYPOTHETICAL PROTEIN	VI.C
RSp0428	RS00887												PSEUDOGENE	VI.C
RSp0429	RS00888												TRANSMEMBRANE MULTIDRUG TRANSPORT PROTEIN	IV.D
RSp0430	RS00889												OXIDOREDUCTASE ALCOHOL DEHYDROGENASE (ADH-HT)	I.H
RSp0431	RS00890					-0,45	0,00			1,46	0,00		OXIDOREDUCTASE COG1231, Monoamine oxidase	I
RSp0432	RS00891			0,65	0,02	-0,34	0,00						TRANSCRIPTION REGULATOR COG3806, Transcriptional activator	VI.A
RSp0433	RS00892					-0,46	0,00						CONSERVED HYPOTHETICAL PROTEIN	VI.C
RSp0434	RS00893					-0,84	0,00						TRANSCRIPTION REGULATOR	VI.A
RSp0435	RS00894					-0,64	0,02						HYPOTHETICAL PROTEIN	VI.B
RSp0436	RS00895												TRANSCRIPTION REGULATOR	VI.A
RSp0437	RS00896												TRANSMEMBRANE TRANSPORT PROTEIN	IV.D
RSp0438	RS00897							0,27	0,04				HYDROLASE	I.I
RSp0439	RS00898					-0,43	0,00			-0,84	0,05		HYPOTHETICAL PROTEIN	VI.D
RSp0440	RS00899												TRANSMEMBRANE MULTIDRUG RESISTANCE PROTEIN HOMOLOG	IV.D
RSp0441	RS00900												TRANSMEMBRANE INNER MEMBRANE MULTIDRUG RESISTANCE PROTEIN	IV.D
RSp0442	RS00901												CHANNEL-FORMING COMPONENT OF A MULTIDRUG RESISTANCE EFFLUX PU	IV.D
RSp0443	RS00902					-0,44	0,00						TRANSCRIPTION REGULATOR	VI.A
RSp0444	RS00903							2,35	0,00				PIRIN-LIKE PROTEIN	VI.C
RSp0445	RS00904												OXIDOREDUCTASE COG1902, NemA, NADH:flavin oxidoreductases	I
RSp0446	RS00905												DEHYDROGENASE	I
RSp0447	RS00906					-0,38	0,00						TRANSCRIPTION REGULATOR	VI.A
RSp0448	RS06029												SIGNAL PEPTIDE	VI.D
RSp0449	RS00908													VI.B
RSp0454	RS00940					-0,91	0,00			-1,80	0,00		RHS-RELATED PROTEIN	VI.B
RSp0455	RS00941					-0,56	0,01						RHS-RELATED PROTEIN	VI.B
RSp0456	RS00942							-1,99	0,05	-2,28	0,03		SIGNAL PEPTIDE	VI.D
RSp0457	RS00943			1,69	0,02	-0,66	0,03						RHS-RELATED PROTEIN	VI.B
RSp0458	RS00945			1,06	0,05	-0,55	0,01						RHS-RELATED PROTEIN	VI.C
RSp0459	RS00947			1,08	0,04								RHS-RELATED PROTEIN	VI.B
RSp0460	RS00948					-0,54	0,01	-0,93	0,00	-0,56	0,02	SRso10	ISRso10-TRANSPOSASE ORFB	V.A
RSp0461	RS00949					-0,45	0,04					tISRso10	ISRSO10-TRANSPOSASE ORFA	V.A
RSp0462	RS00950												SIGNAL PEPTIDE	VI.D
RSp0463	RS00951												HYPOTHETICAL PROTEIN	VI.D
RSp0464	RS00952												GSPE-RELATED PROTEIN	VI.C
RSp0466	RS00954	-3,90	0,01										HYPOTHETICAL PROTEIN	VI.D
RSp0467	RS00345												TRANSMEMBRANE GENERAL SECRETION PATHWAY GSPF-RELATED PROTEIN	IV.E.2
RSp0468	RS00346					-0,53	0,02						TRANSMEMBRANE GENERAL SECRETION PATHWAY GSPG-RELATED PROTEII	IV.E.2
RSp0469	RS00347					-0,61	0,01						TRANSMEMBRANE	VI.C
RSp0470	RS00348					-0,34	0,00						TRANSMEMBRANE GENERAL SECRETION PATHWAY GSPG-RELATED PROTEII	IV.E.2
RSp0471	RS00349			1,13	0,00	-0,40	0,00						TRANSMEMBRANE GENERAL SECRETION PATHWAY GSPG-RELATED PROTEII	IV.E.2
RSp0472	RS00350												SIGNAL PEPTIDE PROLIN-RICH PROTEIN	VI.C
RSp0473	RS00351									2,10	0,01		TRANSMEMBRANE	VI.D
RSp0474	RS00431												GENERAL SECRETION PATHWAY GSPD-RELATED PROTEIN	IV.E.2
RSp0475	RS00352			0,45	0,02			-0,58	0,04			tISRso1	ISRso17-transposase	V.A
RSp0476	RS00354					-0,67	0,00						TRANSMEMBRANE RHS-RELATED PROTEIN	VI.B
RSp0477	RS00355					-0,57	0,00	-1,16	0,00	0,52	0,05		TRANSMEMBRANE RHS-RELATED PROTEIN	VI.C
RSp0478	RS00356	1,39	0,01	0,51	0,01	-0,44	0,02			-0,76	0,00	tISRso7	ISRso7-TRANSPOSASE	V.A
RSp0479	RS00357			0,79	0,01	-0,72	0,00						ABC TRANSPORTER ATP-BINDING PROTEIN	IV.D
RSp0480	RS00358			0,94	0,05	-0,80	0,05						ABC TRANSPORTER AMINO ACID TRANSMEMBRANE PROTEIN	IV.D
RSp0481	RS00359					-1,03	0,00						ABC TRANSPORTER SUBSTRATE-BINDING PERIPLASMIC PROTEIN (PBP)	IV.D
RSp0482	RS00360					-0,99	0,04						TRANSCRIPTION REGULATOR	VI.A
RSp0483	RS00361					-1,27	0,00						ORNITHINE CYCLODEAMINASE	I.I.3
RSp0484	RS00362					-1,09	0,00						CONSERVED HYPOTHETICAL PROTEIN	VI.C
RSp0485	RS00363					-0,58	0,03						HYDROLASE PHOSPHATASE	I.I.4
RSp0486	RS00364												HYPOTHETICAL PROTEIN	VI.D
RSp0487	RS00365					-0,68	0,00			3,08	0,00		TRANSMEMBRANE COG0624, Acetylornithine deacetylase/Succinyl-diaminopimelate	I
RSp0488	RS00366	1,12	0,05									czcS	TRANSCRIPTION REGULATOR TRANSMEMBRANE SENSORY TRANSDUCTION I	IV.G
RSp0489	RS00367							-1,06	0,02			czcR	TRANSCRIPTION REGULATOR RESPONSE REGULATOR PROTEIN FOR COBALT	IV.G
RSp0490	RS00368							-1,66	0,01				SIGNAL PEPTIDE	VI.C
RSp0491	RS00369			0,54	0,01							czcC	COBALT-ZINC-CADMIUM OUTER MEMBRANE RESISTANCE PROTEIN	IV.G
RSp0492	RS00370											czcB	TRANSMEMBRANE COBALT-ZINC-CADMIUM RESISTANCE PROTEIN (CATION EI	IV.G
RSp0493	RS00371											czcA	TRANSMEMBRANE CATION EFFLUX SYSTEM PROTEIN	IV.D
RSp0494	RS00372					-0,19	0,03					hit	HIT-LIKE PROTEIN	VI.C
RSp0495	RS00373									1,27	0,04		HYPOTHETICAL PROTEIN	VI.D
RSp0496	RS00374									0,78	0,01		HYPOTHETICAL PROTEIN	VI.D
RSp0497	RS00375							1,39	0,00				HYPOTHETICAL PROTEIN	VI.D
RSp0498	RS00376					0,50	0,02	1,95	0,00	1,13	0,01		HYPOTHETICAL PROTEIN	VI.B
RSp0499	RS00378							0,53	0,01	0,58	0,00		TRANSMEMBRANE TRANSPORT PROTEIN	IV.D
RSp0500	RS00379									0,58	0,04		HYPOTHETICAL PROTEIN	VI.D
RSp0501	RS00380												HYPOTHETICAL PROTEIN	VI.D
RSp0502	RS06030												HYPOTHETICAL PROTEIN	VI.D
RSp0505	RS00384												OXIDOREDUCTASE COG1052, Lactate dehydrogenase and related dehydrogenases	V.A
RSp0506	RS00385					-0,88	0,00	1,81	0,00	2,14	0,00		TRANSMEMBRANE GLYCIN-RICH PROTEIN	VI.B
RSp0507	RS00386	-0,59	0,03			-0,55	0,00			-0,57	0,00	cheD1	TRANSMEMBRANE METHYL-ACCEPTING CHEMOTAXIS PROTEIN I MCP-I SERIN	IV.C
RSp0508	RS00387					-0,30	0,01					nfrB	TRANSMEMBRANE INNER MEMBRANE protein	III.B.1
RSp0509	RS00388					-1,29	0,02						SIGNAL PEPTIDE	VI.C
RSp0510	RS00389											epsC2	UDP-N-ACETYLGLUCOSAMINE 2-EPIMERASE	III.A.1
RSp0511	RS00390												SIGNAL PEPTIDE	VI.D
RSp0512	RS00391												TRANSMEMBRANE COG5298, Uncharacterized protein conserved in bacteria	VI.C
RSp0513	RS05802					-0,86	0,03	-2,02	0,04				TRANSMEMBRANE	VI.C
RSp0514	RS00392					-1,00	0,01	2,03	0,03				TRANSMEMBRANE MECHANOSENSITIVE ION CHANNEL	IV.D
RSp0515	RS00394												CONSERVED HYPOTHETICAL PROTEIN	VI.C
RSp0516	RS00395							2,00	0,00				HYPOTHETICAL PROTEIN	VI.B
RSp0517	RS00396	0,51	0,01			-0,49	0,00	-1,32	0,03				CONSERVED HYPOTHETICAL PROTEIN	VI.C
RSp0518	RS00397					-0,76	0,01						VGR-RELATED PROTEIN	VI.C
RSp0519	RS00398												TRANSMEMBRANE SIGNAL TRANSDUCTION PROTEIN GGDEF DOMAIN	VI.A
RSp0520	RS00399			0,54	0,03							mntH2	TRANSMEMBRANE MANGANESE TRANSPORT PROTEIN	IV.D.1
RSp0521	RS00400			0,85	0,05	-0,61	0,03					hscC	TRANSMEMBRANE CHAPERONE PROTEIN	IV.B
RSp0522	RS00401												TRANSMEMBRANE	VI.B
RSp0523	RS00402									-0,85	0,00		TRANSCRIPTION REGULATOR TWO COMPONENT RESPONSE REGULATOR	VI.A
RSp0524	RS00403							-0,60	0,02				TRANSCRIPTION REGULATOR TRANSMEMBRANE SENSOR HISTIDINE KINASE	VI.A
RSp0525	RS00404							-1,10	0,00				OXIDOREDUCTASE COG2910, Putative NADH-flavin reductase	I
RSp0526	RS00405					-0,50	0,03	-0,98	0,00				COG1959, Predicted transcriptional regulator	VI.A
RSp0527	RS00406			-0,99	0,01								TRANSMEMBRANE	VI.D
RSp0528	RS00407			-1,38	0,00	1,22	0,00						outer membrane cation efflux system	IV.G
RSp0529	RS00408												cation efflux protein	IV.G
RSp0530	RS00409			-0,87	0,04								TRANSMEMBRANE cation efflux system	IV.D
RSp0531	RS00410					-1,05	0,02	-1,22	0,01				TRANSCRIPTION REGULATOR PROTEIN P-II-RELATED PROTEIN	VI.A
RSp0532	RS00411			2,25	0,02	-3,36	0,00						TRANSMEMBRANE AMINOPEPTIDASE	II.A.4
RSp0533	RS00413												TRANSMEMBRANE	VI.D
RSp0534	RS00414									1,22	0,05	icc	COG1409, Predicted phosphohydrolases	I
RSp0535	RS00415			1,18	0,02					3,37	0,01		TRANSCRIPTION REGULATOR	VI.A
RSp0536	RS00416	-1,43	0,01	1,61	0,00	-0,48	0,01			1,53	0,00		TRANSMEMBRANE SUGAR-PROTON SYMPORTER	IV.D
RSp0537	RS05705			1,31	0,01					1,18	0,00		HYPOTHETICAL PROTEIN	VI.D
RSp0538	RS00417			1,44	0,01	-0,56	0,00						TRANSMEMBRANE	VI.C
RSp0539	RS00418												ABC TRANSPORTER COG0683, ABC-type branched-chain amino acid transport syst	IV.D
RSp0540	RS05701												HEMAGGLUTININ-RELATED PROTEIN	VI.C
RSp0541	RS05801												CONSERVED HYPOTHETICAL PROTEIN	VI.C

RSp0542	RS05800					0,61	0,01						CONSERVED HYPOTHETICAL PROTEIN	VI.C
RSp0543	RS05799					-0,52	0,00	-1,09	0,02				HYPOTHETICAL PROTEIN	VI.D
RSp0544	RS03936												HYPOTHETICAL PROTEIN	VI.B
RSp0546	RS03935					-0,36	0,00			-2,17	0,00		HYPOTHETICAL PROTEIN	VI.C
RSp0547	RS03933					-0,34	0,05					tISRso8	ISRso8-TRANSPOSASE ORFA	VI.D
RSp0549	RS03931					-0,75	0,04						TRANSMEMBRANE	VI.D
RSp0550	RS03929					-0,71	0,01						SIGNAL PEPTIDE ACTIVATION/SECRETION PROTEIN	IV.E.2
RSp0551	RS03928												TRANSMEMBRANE COG0402, Cytosine deaminase and related metal-dependent hy	I
RSp0552	RS03927			0,43	0,04	-0,61	0,00						TRANSMEMBRANE	VI.C
RSp0553	RS03926												CONSERVED HYPOTHETICAL PROTEIN	VI.C
RSp0554	RS03925					-1,83	0,00	-1,70	0,00			chrB	SIGNAL PEPTIDE CHROMATE RESISTANCE PROTEIN	IV.D.1
RSp0555	RS03924					-0,65	0,01					chrA	TRANSMEMBRANE CHROMATE TRANSPORT PROTEIN	IV.D.1
RSp0556	RS03923					-0,48	0,00						CONSERVED HYPOTHETICAL PROTEIN	VI.C
RSp0557	RS03922			0,43	0,02			-0,47	0,01			tISRso16	ISRso16-TRANSPOSASE ORFA	V.A
RSp0559	RS03920			-0,41	0,02								TRANSMEMBRANE	V.A
RSp0560	RS03918					-0,29	0,05	-1,03	0,03				HYPOTHETICAL PROTEIN	VI.D
RSp0561	RS03917					-1,13	0,02						PSEUDOGENE (TRANSCRIPTION REGULATOR C-terminal fragment)	VI.C
RSp0562	RS03916												PSEUDOGENE (TRANSCRIPTION REGULATOR N-terminal fragment)	VI.C
RSp0563	RS03915												COG0526, Thiol-disulfide isomerase and thioredoxins	II.B.7
RSp0564	RS03914												LIPOPROTEIN	VI.C
RSp0565	RS03913					-0,45	0,02						SIGNAL PEPTIDE	VI.D
RSp0567	RS03911											tISRsob	ISRSO10-TRANSPOSASE ORFB	V.A
RSp0568	RS03910												CONSERVED HYPOTHETICAL PROTEIN	VI.C
RSp0569	RS03909							0,78	0,01	1,54	0,00		HYPOTHETICAL PROTEIN	VI.D
RSp0570	RS06032												COG1475, Predicted transcriptional regulators	VI.A
RSp0571	RS06031					-0,68	0,04						CONSERVED HYPOTHETICAL PROTEIN	VI.C
RSp0572	RS03907	-1,73	0,00	-4,10	0,00	5,72	0,00	2,69	0,00	-1,17	0,00		CONSERVED HYPOTHETICAL PROTEIN	VI.C
RSp0573	RS03906							-0,95	0,03				HYPOTHETICAL PROTEIN	VI.B
RSp0574	RS03905												CONSERVED HYPOTHETICAL PROTEIN	VI.C
RSp0575	RS03904					1,22	0,00						CONSERVED HYPOTHETICAL PROTEIN	VI.C
RSp0576	RS03903					-0,90	0,00						CONSERVED HYPOTHETICAL PROTEIN	VI.C
RSp0577	RS03902					-0,68	0,01			1,66	0,00		CONSERVED HYPOTHETICAL PROTEIN	VI.C
RSp0578	RS03901												HYPOTHETICAL PROTEIN	VI.D
RSp0579	RS03900					0,84	0,05						BACTERIOPHAGE-RELATED PROTEIN	V.D
RSp0580	RS03899												PRODUCT OF A TRUNCATED BACTERIOPHAGE GENE	V.D
RSp0583	RS03897			0,61	0,00			0,77	0,04				EXOGLUCANASE A (1,4-BETA-CELLOBIOSIDASE)	II.A.3
RSp0585	RS03895												REMNANT OF tIS1421-TRANSPOSASE ORFA	V.A
RSp0586	RS03894					-0,89	0,02						HYPOTHETICAL PROTEIN	VI.D
RSp0587	RS03893			-2,52	0,04	3,95	0,00						SIGNAL PEPTIDE	VI.B
RSp0588	RS03892												TRANSPOSASE FRAGMENT	V.A
RSp0589	RS03890					-0,30	0,05						OXIDOREDUCTASE DEHYDROGENASE/REDUCTASE	I.I
RSp0590	RS03889												TRANSCRIPTION REGULATOR	VI.A
RSp0591	RS03891					-0,41	0,05	-1,13	0,04				HYPOTHETICAL PROTEIN	VI.D
RSp0592	RS03888												HYPOTHETICAL PROTEIN	VI.D
RSp0593	RS03887			-1,26	0,00	1,16	0,00						HYPOTHETICAL PROTEIN	VI.D
RSp0594	RS03886												HYPOTHETICAL PROTEIN	VI.D
RSp0595	RS03884					-0,67	0,00	-0,47	0,02				CONSERVED HYPOTHETICAL PROTEIN	VI.C
RSp0596	RS03883							1,91	0,00	2,77	0,00		TRANSMEMBRANE	VI.B
RSp0597	RS03882												CONSERVED HYPOTHETICAL PROTEIN	VI.C
RSp0598	RS03881							-0,93	0,02				TRANSCRIPTION REGULATOR RESPONSE REGULATOR	VI.A
RSp0599	RS03880					-1,67	0,01						TRANSCRIPTION REGULATOR	VI.A
RSp0600	RS03879												COG4319, Ketosteroid isomerase homolog	VI.C
RSp0601	RS03878											phnB	CONSERVED HYPOTHETICAL PROTEIN	VI.C
RSp0602	RS03877	-0,65	0,02			-0,73	0,01	0,50	0,02	1,45	0,00		SIGNAL PEPTIDE	VI.D
RSp0603	RS03876	-2,65	0,00	0,53	0,02	-1,60	0,00	2,34	0,00	3,13	0,00		SERINE PROTEASE	II.A.4
RSp0604	RS03875							-1,55	0,03			paaA	PHENYLACETIC ACID DEGRADATION PROTEIN	I.I.4
RSp0605	RS03874					-0,43	0,04					paaB	PHENYLACETIC ACID DEGRADATION PROTEIN	I.I.4
RSp0606	RS03873											paaC	PHENYLACETIC ACID DEGRADATION PROTEIN	I.I.4
RSp0607	RS03872					-0,44	0,03					paaD	PHENYLACETIC ACID DEGRADATION PROTEIN	I.I.4
RSp0608	RS03871					-0,58	0,03					paaE	FERREDOXIN REDUCTASE	I.G.1
RSp0609	RS03758			-1,38	0,02								HYPOTHETICAL PROTEIN	VI.D
RSp0610	RS03757												CONSERVED HYPOTHETICAL PROTEIN	VI.C
RSp0611	RS03756					-0,65	0,00						TRANSMEMBRANE	VI.C
RSp0612	RS03755												TRANSMEMBRANE	VI.C
RSp0613	RS03754												TRANSCRIPTION REGULATOR	VI.A
RSp0614	RS03753												OXIDOREDUCTASE COG0778, Nitroreductase	I
RSp0615	RS03752			0,54	0,03							cobB	COBYRINIC ACID A,C-DIAMIDE SYNTHASE	I.B.3
RSp0616	RS03751			0,71	0,02							cobA	COB(I)ALAMIN ADENOSYLTRANSFERASE	I.B.3
RSp0617	RS03750			1,60	0,03							cobW	COBALAMIN BIOSYNTHESIS PROTEIN	I.B.3
RSp0618	RS03749			0,94	0,01							cbiJH	OXIDOREDUCTASE BIFUNCTIONAL PROTEIN: PRECORRIN-3 METHYLTRANSFE	I.B.3
RSp0619	RS03748							0,81	0,04			cbiG	PRECORRIN METHYLASE	I.B.3
RSp0620	RS03747											cbiF	PRECORRIN-4 C11-METHYLTRANSFERASE	I.B.3
RSp0621	RS03746							1,02	0,05			cbiL	PRECORRIN-2 C20-METHYLTRANSFERASE	I.B.3
RSp0622	RS03745											cbiD	TRANSMEMBRANE COBALAMIN BIOSYNTHESIS PROTEIN D	I.B.3
RSp0623	RS03744			0,73	0,00							cobH	TRANSMEMBRANE PRECORRIN ISOMERASE	I.B.3
RSp0624	RS03743			0,65	0,00			2,29	0,00			cobL	PRECORRIN-6B METHYLASE METHYLTRANSFERASE	I.B.3
RSp0625	RS03742			0,70	0,00			-1,09	0,00				MAGNESIUM CHELATASE (SUBUNITS D/I)	I
RSp0626	RS03741			0,50	0,03							cobN	COBALAMIN BIOSYNTHESIS PROTEIN	I.B.3
RSp0627	RS03740			0,89	0,00	-0,45	0,00	-0,59	0,01				FERREDOXIN	I.G.1
RSp0628	RS03739			0,91	0,00	-0,42	0,01			-1,32	0,01	hoxN	TRANSMEMBRANE HIGH AFFINITY NICKEL TRANSPORTER	IV.D.1
RSp0629	RS03738	0,84	0,03	0,60	0,03	-0,83	0,00			-1,99	0,00		VGR-RELATED PROTEIN	VI.C
RSp0630	RS03737			0,63	0,00					-3,27	0,01		PHOSPHOLIPASE	VI.C
RSp0631	RS03736			0,66	0,00	-0,91	0,00			-1,00	0,00		LIPOPROTEIN WITH COG0790, FOG: TPR repeat, SEL1 subfamily	VI.C
RSp0632	RS03735	1,52	0,03	0,76	0,04	-0,78	0,00	-1,26	0,05				CONSERVED HYPOTHETICAL PROTEIN	VI.C
RSp0633	RS03734			0,85	0,00	-0,86	0,00						HYPOTHETICAL PROTEIN	VI.D
RSp0634	RS03733					-0,39	0,02			0,65	0,00	rfaC2	LIPOPOLYSACCHARIDE HEPTOSYLTRANSFERASE	II.B.2
RSp0635	RS03732												TRANSMEMBRANE ACYLTRANSFERASE	VI.C
RSp0636	RS03731			1,36	0,01	-0,36	0,05						TRANSCRIPTION REGULATOR COG4941, Predicted RNA polymerase sigma factor	VI.A
RSp0637	RS03730							-1,69	0,01				CONSERVED HYPOTHETICAL PROTEIN	VI.C
RSp0638	RS03729												ABC TRANSPORTER COG4615, ABC-type siderophore export system, fused ATPase	IV.F
RSp0639	RS03728			0,63	0,01	-0,27	0,03			-1,76	0,00		COG2220, Predicted Zn-dependent hydrolases of the beta-lactamase fold	I
RSp0640	RS03727			0,47	0,02	-0,31	0,04			-1,61	0,00		CONSERVED HYPOTHETICAL PROTEIN	VI.C
RSp0641	RS05860			0,75	0,01								PEPTIDE SYNTHETASE	IV.G
RSp0642	RS05859			0,39	0,02								PEPTIDE SYNTHETASE	IV.G.3
RSp0643	RS05579			-0,77	0,00	1,12	0,00						HYPOTHETICAL PROTEIN	VI.D
RSp0644	RS05578												SIGNAL PEPTIDE	VI.D
RSp0645	RS05577												TRANSCRIPTION REGULATOR	VI.A
RSp0646	RS05576			0,68	0,01								1-AMINOCYCLOPROPANE-1-CARBOXYLATE DEAMINASE (ACC DEAMINASE)	I
RSp0647	RS05575												ENOYL-COENZYME A HYDRATASE	I
RSp0648	RS05574												ENOYL-COA HYDRATASE	I.I.4
RSp0649	RS05573											mmsB	OXIDOREDUCTASE 3-HYDROXYISOBUTYRATE DEHYDROGENASE	I.A.2
RSp0650	RS05572											mmsA	OXIDOREDUCTASE METHYLMALONATE-SEMIALDEHYDE DEHYDROGENASE	I.A.2
RSp0651	RS05571												ACETYL-COENZYME A SYNTHETASE (ACETATE--COA LIGASE)	I.C
RSp0652	RS05570												OXIDOREDUCTASE ACYL-COA DEHYDROGENASE	I
RSp0653	RS05569												TRANSCRIPTION REGULATOR	VI.A
RSp0654	RS05568							-1,16	0,00			copS	TRANSCRIPTION REGULATOR TWO COMPONENT SENSOR HISTIDINE KINASE	IV.G
RSp0655	RS05567											copR	TRANSCRIPTION REGULATOR TWO COMPONENT RESPONSE REGULATOR	IV.G
RSp0656	RS05566					-0,58	0,03					copA	TRANSMEMBRANE COPPER RESISTANCE PROTEIN	IV.G
RSp0657	RS01792					0,98	0,00					copB	COPPER RESISTANCE PROTEIN B	IV.G.4
RSp0658	RS01791					-0,38	0,03					copC	SIGNAL PEPTIDE COPPER RESISTANCE PROTEIN C	IV.G.4
RSp0659	RS01790					0,40	0,02					copD	TRANSMEMBRANE COPPER RESISTANCE PROTEIN D	IV.G.4
RSp0660	RS01789					-0,24	0,01						PORIN	IV.D

RSp0661	RS01788			-0,46 0,01				COG1335 Amidase related to nicotinamidase	I
RSp0662	RS01787							TRANSCRIPTION REGULATOR	VI.A
RSp0663	RS01786				-0,78 0,03			TRANSMEMBRANE TRANSPORT PROTEIN	IV.D
RSp0664	RS01785			-0,44 0,01		0,63 0,00		TRANSCRIPTION REGULATOR	VI.A
RSp0665	RS01784			-0,30 0,00				TRANSMEMBRANE MAGNESIUM AND COBALT TRANSPORT PROTEIN	IV.D.7
RSp0666	RS01783						tag	DNA-3-METHYLADENINE GLYCOSYLASE I	II.B.8
RSp0667	RS01782			-0,39 0,02		-1,21 0,00		TRANSMEMBRANE CYNX-RELATED TRANSPORT PROTEIN	IV
RSp0668	RS01781							CONSERVED HYPOTHETICAL PROTEIN	VI.C
RSp0669	RS01780							OUTER MEMBRANE CHANEL LIPOPROTEIN	IV.D
RSp0670	RS01779				0,21 0,05			TRANSMEMBRANE TRANSPORTER LIPOPROTEIN	IV.D.7
RSp0671	RS01778			-0,73 0,00				ENOYL COA-HYDRATASE	I.I.4
RSp0672	RS01777		-3,50 0,00	4,43 0,00	3,56 0,00			LRR-GALA FAMILY TYPE III EFFECTOR PROTEIN (GALA 2)	VI.C
RSp0673	RS01776				-0,44 0,05			LIPOPROTEIN	VI.C
RSp0674	RS01775	1,47 0,02		-0,77 0,00		-1,69 0,01		LIPOPROTEIN	VI.C
RSp0675	RS01774			-0,62 0,01	-1,59 0,04			LIPOPROTEIN	VI.C
RSp0676	RS01773	-2,02 0,00		-0,76 0,00	1,47 0,00	3,92 0,00	metE	5-METHYLTETRAHYDROPTEROYLTRIGLUTAMATE--HOMOCYSTEINE METHYLT	I.A.12
RSp0677	RS01772				1,74 0,01		metR	TRANSCRIPTION REGULATOR TRANSCRIPTIONAL ACTIVATOR PROTEIN METR	I.A.12
RSp0678	RS01771	3,80 0,00	0,83 0,00	-0,90 0,01		2,65 0,00	pdxH2	OXIDOREDUCTASE PYRIDOXAMINE 5'-PHOSPHATE OXIDASE (PNP/PMP OXIDA	I.B.10
RSp0679	RS01770	4,24 0,00	1,04 0,00	-0,96 0,00		2,12 0,00		DIAMINOPIMELATE DECARBOXYLASE	I.A.13
RSp0680	RS01769	3,26 0,00	0,76 0,00	-0,77 0,00		2,18 0,00	trpC2	INDOLE-3-GLYCEROL PHOSPHATE SYNTHASE	I.A.6
RSp0681	RS01768	1,47 0,00	0,87 0,03	-0,40 0,00	0,75 0,01	1,20 0,00	trpD2	ANTHRANILATE PHOSPHORIBOSYLTRANSFERASE (GLYCOSYLTRANSFERASE	I.A.6
RSp0682	RS01767	4,94 0,00	0,87 0,00	-0,47 0,03	0,83 0,02	2,72 0,00		OXIDOREDUCTASE TRIMETHYLAMINE DEHYDROGENASE	I.I
RSp0683	RS01766	4,26 0,00	0,87 0,00	-0,65 0,00	1,01 0,01	2,58 0,00		COG0325, Predicted enzyme with a TIM-barrel fold	VI.C
RSp0684	RS01765	4,35 0,00	0,82 0,00	-0,59 0,00	0,63 0,01	2,69 0,00		CONSERVED HYPOTHETICAL PROTEIN	VI.C
RSp0685	RS01764	3,93 0,00	0,58 0,00		0,60 0,02	3,83 0,00		TRANSMEMBRANE	VI.C
RSp0686	RS01763	-0,99 0,01			0,58 0,04			TRANSCRIPTION REGULATOR	VI.A
RSp0687	RS01762			-0,45 0,04				OXIDOREDUCTASE ACYL-CoA DEHYDROGENASE	I.I.1
RSp0688	RS01761				0,91 0,04			TRANSMEMBRANE COG5472, Predicted small integral membrane protein	VI.C
RSp0689	RS01760			-0,12 0,04				TRANSCRIPTION REGULATOR COG2808, Transcriptional regulator	VI.A
RSp0690	RS01759							FUMARYLACETOACETASE (FUMARYLACETOACETATE HYDROLASE)	I.I.3
RSp0691	RS01758						hmgA	OXIDOREDUCTASE HOMOGENTISATE 1,2-DIOXYGENASE DIOXYGENASE	I.I.3
RSp0692	RS01757							TRANSCRIPTION REGULATOR TRANSCRIPTION REGULATOR	VI.A
RSp0693	RS01756		-7,98 0,00	7,44 0,00	2,38 0,00	-2,42 0,04		COG0346, Lactoylglutathione lyase and related lyases	I
RSp0694	RS01755		-6,81 0,00	6,90 0,00	3,04 0,00			OXIDOREDUCTASE TRYPTOPHAN-2,3-DIOXYGENASE	I.A.6
RSp0695	RS01754		-6,37 0,00	5,69 0,00	2,50 0,00			SIGNAL PEPTIDE COG0644, Dehydrogenases (flavoproteins)	I
RSp0696	RS01753		-7,32 0,00	6,34 0,00	0,97 0,00			COG0520, Selenocysteine lyase	I
RSp0697	RS01752		-6,97 0,00	7,20 0,00	3,16 0,00			OXIDOREDUCTASE DEHYDROGENASE/REDUCTASE	I
RSp0698	RS01751		-5,62 0,00	6,25 0,00	1,27 0,00			AMINOTRANSFERASE	I
RSp0699	RS01750		0,51 0,03					PEPTIDE CHAIN RELEASE FACTOR HOMOLOG	II.B.7
RSp0700	RS01749		0,47 0,03	-0,52 0,00	-0,71 0,05	-0,42 0,05		RTCB-LIKE PROTEIN	VI.C
RSp0701	RS01748		-1,97 0,01					CONSERVED HYPOTHETICAL PROTEIN	VI.C
RSp0702	RS01747							OXIDOREDUCTASE COG0654, 2-polyprenyl-6-methoxyphenol hydroxylase and relat	I
RSp0703	RS01746							COG0491, Zn-dependent hydrolases, including glyoxylases	I
RSp0704	RS01745			-0,73 0,02				TRANSCRIPTION REGULATOR	VI.A
RSp0705	RS01744		0,68 0,03					ABC TRANSPORTER AMINO-ACID TRANSMEMBRANE PROTEIN	IV.D.2
RSp0706	RS01743			-0,34 0,02				ABC TRANSPORTER AMINO-ACID COMPOSITE ATP-BINDING TRANSMEMBRANI	IV.D.2
RSp0707	RS01742		0,68 0,00					ABC TRANSPORTER AMINO-ACID COMPOSITE ATP-BINDING TRANSMEMBRANI	IV.D.2
RSp0708	RS01741					0,83 0,00		SIGNAL PEPTIDE	VI.C
RSp0709	RS01740			0,26 0,00	1,41 0,00	1,21 0,04		OXIDOREDUCTASE CYTOCHROME P-450-LIKE MONOOXYGENASE	I.G.1
RSp0710	RS01739							TRANSMEMBRANE	VI.C
RSp0711	RS01738			-0,42 0,01				TRANSMEMBRANE COG5486, Predicted metal-binding integral membrane protein	III.B.1
RSp0712	RS01737				-0,65 0,01	0,77 0,03		CONSERVED HYPOTHETICAL PROTEIN	VI.C
RSp0713	RS01736			-0,50 0,04	-0,45 0,05			CONSERVED HYPOTHETICAL PROTEIN	VI.C
RSp0714	RS01735		0,73 0,02	-0,30 0,01				D-ALANINE AMINOTRANSFERASE (D-ASPARTATE AMINOTRANSFERASE)	I.F.14
RSp0715	RS01734							SIGNAL PEPTIDE	VI.B
RSp0716	RS01733			-1,69 0,00		0,95 0,00		HYPOTHETICAL PROTEIN	VI.D
RSp0717	RS01732			-0,64 0,03				HYPOTHETICAL PROTEIN	VI.D
RSp0718	RS01731			-1,18 0,00				COG1858, Cytochrome c peroxidase	I
RSp0719	RS01730		0,70 0,00	-1,09 0,00				SIGNAL PEPTIDE	VI.C
RSp0720	RS01729			-1,02 0,03				SIGNAL PEPTIDE	VI.C
RSp0721	RS01728			-0,44 0,05				TRANSMEMBRANE	VI.C
RSp0722	RS01727			-0,31 0,01	1,11 0,00			COG1712, Predicted dinucleotide-utilizing enzyme	VI.C
RSp0723	RS01725							TRANSMEMBRANE	VI.D
RSp0724	RS01726				-1,32 0,00			HYPOTHETICAL PROTEIN	VI.D
RSp0725	RS01723							OXIDOREDUCTASE COG0535, Predicted Fe-S oxidoreductases	VI.C
RSp0726	RS01724			-0,40 0,04				TRANSMEMBRANE MFS TRANSPORTER	IV.D
RSp0727	RS01701			-0,34 0,01				CONSERVED HYPOTHETICAL PROTEIN	VI.C
RSp0728	RS01700			-1,00 0,00				HYPOTHETICAL PROTEIN	VI.D
RSp0729	RS01699			0,32 0,01				CONSERVED HYPOTHETICAL PROTEIN	VI.C
RSp0730	RS01698		0,77 0,01					TRANSMEMBRANE COG3000, Sterol desaturase	I
RSp0731	RS01697			2,35 0,00				TREHALOSE-6-PHOSPHATE SYNTHASE (ALPHA,ALPHA-TREHALOSE-PHOSPHA	IV.F.2
RSp0732	RS05796		-5,90 0,00	6,74 0,00	4,07 0,02			TRANSMEMBRANE	VI.D
RSp0733	RS05792							HYPOTHETICAL PROTEIN	VI.B
RSp0734	RS05791		0,45 0,04			-1,31 0,03		CONSERVED HYPOTHETICAL PROTEIN	VI.C
RSp0735	RS01974							CONSERVED HYPOTHETICAL PROTEIN	VI.C
RSp0736	RS01973					-2,39 0,02		TRANSMEMBRANE	VI.C
RSp0737	RS01972							HYPOTHETICAL PROTEIN	VI.D
RSp0738	RS01970			-0,97 0,00		-1,74 0,03		VGR-RELATED PROTEIN	VI.C
RSp0739	RS01969			-0,94 0,00				TRANSMEMBRANE	VI.C
RSp0740	RS01968		0,43 0,03	-0,92 0,00				CONSERVED HYPOTHETICAL PROTEIN	VI.C
RSp0741	RS01967			-0,98 0,00	-2,64 0,00	-1,40 0,00		TRANSMEMBRANE	VI.C
RSp0742	RS01966		0,41 0,00	-1,01 0,00	-1,32 0,00	-1,58 0,00		LIPOPROTEIN	VI.C
RSp0743	RS01965		0,33 0,03	-1,14 0,00		-1,53 0,00		CONSERVED HYPOTHETICAL PROTEIN	VI.C
RSp0744	RS01964			-1,19 0,00	-0,60 0,03	-0,92 0,04		CONSERVED HYPOTHETICAL PROTEIN	VI.C
RSp0745	RS01963		0,69 0,03	-1,24 0,00	-0,95 0,01	-1,39 0,00		CONSERVED HYPOTHETICAL PROTEIN	VI.C
RSp0746	RS01962			-0,24 0,01	0,59 0,02			CONSERVED HYPOTHETICAL PROTEIN	VI.C
RSp0747	RS01961		0,54 0,01	-0,93 0,00	-1,72 0,00	-2,23 0,00		CONSERVED HYPOTHETICAL PROTEIN	VI.C
RSp0748	RS01960		0,45 0,02	-0,83 0,00	-1,90 0,00	-1,74 0,00		CONSERVED HYPOTHETICAL PROTEIN	VI.C
RSp0749	RS01959		0,78 0,01	-0,97 0,00		-2,31 0,00		CLPA/B-TYPE CHAPERONE	II.A.4
RSp0750	RS01958							VGR-RELATED PROTEIN	VI.C
RSp0751	RS01957		0,70 0,00	-0,88 0,00		-1,31 0,01		HYPOTHETICAL PROTEIN	VI.D
RSp0752	RS01956		0,54 0,04	-0,94 0,00	-1,81 0,00	-1,16 0,00		TRANSMEMBRANE	VI.B
RSp0753	RS01955		0,78 0,02	-1,12 0,01	-2,07 0,00	-2,31 0,00		TRANSMEMBRANE	VI.D
RSp0754	RS01954		0,66 0,01	-0,83 0,00	-2,49 0,01			HYPOTHETICAL PROTEIN	VI.D
RSp0755	RS01953			-0,41 0,02				TRANSMEMBRANE	VI.D
RSp0756	RS01952		0,82 0,00	-0,52 0,00	-1,99 0,00	-2,99 0,00		HYPOTHETICAL PROTEIN	VI.D
RSp0757	RS01951			-0,59 0,02				CONSERVED HYPOTHETICAL PROTEIN	VI.C
RSp0758	RS01950		0,91 0,01	-0,71 0,00				CONSERVED HYPOTHETICAL PROTEIN	VI.C
RSp0759	RS01949		0,56 0,04	-0,28 0,03				CONSERVED HYPOTHETICAL PROTEIN	VI.C
RSp0760	RS01948		0,65 0,00	-0,58 0,00				TRANSMEMBRANE	VI.D
RSp0761	RS01947			0,92 0,02				COG2885, Outer membrane protein and related peptidoglycan-associated (lipo)protei	VI.B
RSp0762	RS01946		0,90 0,00	-0,55 0,00				HYPOTHETICAL PROTEIN	VI.B
RSp0763	RS01945		0,68 0,01					TRANSMEMBRANE	VI.C
RSp0764	RS01944		0,37 0,00	-0,64 0,00				TRANSMEMBRANE	VI.B
RSp0765	RS01943		0,42 0,02	-0,69 0,00	-0,79 0,03	-0,97 0,00		CONSERVED HYPOTHETICAL PROTEIN	VI.C
RSp0766	RS01942							TRANSMEMBRANE	VI.B
RSp0767	RS01941			-0,90 0,00	-0,76 0,02	-0,98 0,00		TRANSMEMBRANE	VI.D
RSp0768	RS01940		0,36 0,00	-0,47 0,00		-0,58 0,00		VGR-RELATED PROTEIN	VI.C
RSp0769	RS01939			-0,32 0,02	0,85 0,04			TRANSMEMBRANE	VI.C
RSp0770	RS01938			-0,94 0,00		-1,36 0,00		CONSERVED HYPOTHETICAL PROTEIN	VI.C
RSp0771	RS01937			-0,93 0,01		-1,27 0,01		CONSERVED HYPOTHETICAL PROTEIN	VI.C
RSp0772	RS01936		0,55 0,05		0,81 0,01	0,91 0,01		SIGNAL PEPTIDE	VI.C

RSp0773	RS01935			0,63	0,01	-0,88	0,00	-3,33	0,00	-2,57	0,00		SIGNAL PEPTIDE	VI.D
RSp0774	RS01934												HYPOTHETICAL PROTEIN	VI.D
RSp0775	RS01933												HYPOTHETICAL PROTEIN	VI.D
RSp0776	RS01932												HYPOTHETICAL PROTEIN	VI.D
RSp0777	RS01931												TRANSMEMBRANE	VI.D
RSp0778	RS01929					-0,81	0,01						TRANSCRIPTION REGULATOR TRANSCRIPTIONAL ACTIVATOR PROTEIN OF QU	I.E.1
RSp0779	RS01928	2,23	0,01			-2,10	0,00						AUTOINDUCER SYNTHASE (ACYL HOMOSERINE LACTONE SYNTHASE)	I.E.1
RSp0780	RS01927	2,21	0,00	1,80	0,02	-2,29	0,00						Abhydrolase	I.D.1
RSp0781	RS01926			2,42	0,03	-1,92	0,00						COG0626, Cystathionine beta-lyases/cystathionine gamma-synthases	I
RSp0782	RS01925	1,35	0,05	2,65	0,02	-2,19	0,00						tRNA synthetase - related protein	I
RSp0783	RS01924			2,09	0,05	-1,81	0,00	-1,06	0,04				COG0236, Acyl carrier protein	I.D
RSp0784	RS01923			2,58	0,02	-2,36	0,00						TRANSMEMBRANE FATTY ACID DESATURASE	I.I.1
RSp0785	RS01922			2,09	0,01	-1,66	0,00	-1,00	0,03				HYPOTHETICAL PROTEIN	VI.D
RSp0786	RS01921			2,26	0,02	-2,02	0,00	-1,87	0,00				COG0332, 3-oxoacyl-[acyl-carrier-protein]	I.B.16
RSp0787	RS01920			2,51	0,02	-2,24	0,00	-1,30	0,02				TRANSMEMBRANE COG0318, Acyl-CoA synthetases (AMP-forming)/AMP-acid ligas	I
RSp0788	RS01919			2,44	0,02	-2,42	0,00						COG2192, Predicted carbamoyl transferase	I
RSp0789	RS01918			2,25	0,02	-1,27	0,00	-0,98	0,01				HYPOTHETICAL PROTEIN	VI.B
RSp0790	RS01917			2,00	0,04	-2,27	0,00						COG0596, Predicted hydrolases or acyltransferases (alpha/beta hydrolase superfamil	I
RSp0791	RS01916			1,91	0,04			-0,90	0,00				COG0236, Acyl carrier protein	I
RSp0792	RS01915			2,69	0,03	-2,49	0,00						TRANSMEMBRANE FATTY ACID DESATURASE	I.I.1
RSp0793	RS01914					-1,55	0,00	-3,76	0,03				HYPOTHETICAL PROTEIN	VI.D
RSp0794	RS01913			1,13	0,01	0,26	0,03						AMINOTRANSFERASE	I.F.14
RSp0795	RS01912			0,41	0,01	-0,81	0,00						COG4757, Predicted alpha/beta hydrolase	I.I
RSp0796	RS01911												TRANSCRIPTION REGULATOR	VI.A
RSp0797	RS01910												TRANSMEMBRANE TRANSPORT PROTEIN	IV.D
RSp0798	RS01909												HYPOTHETICAL PROTEIN	VI.B
RSp0799	RS01908			0,83	0,01								COG0389, Nucleotidyltransferase/DNA polymerase involved in DNA repair	II.B.8
RSp0800	RS01907											dnaE2	DNA POLYMERASE III (ALPHA CHAIN)	II.B.8
RSp0801	RS01906					-0,48	0,03	-0,47	0,00	-0,26	0,05		TRANSCRIPTION REGULATOR	VI.A
RSp0802	RS01905												TRANSMEMBRANE COG0471, Di- and tricarboxylate transporters	IV.D
RSp0803	RS01904					0,91	0,00			-0,57	0,04		SIGNAL PEPTIDE HEMAGGLUTININ-RELATED PROTEIN	VI.B
RSp0804	RS01903			0,57	0,00					-1,32	0,00	fusA2	ELONGATION FACTOR G (EF-G PROTEIN)	II.B.7
RSp0805	RS01902												TRANSCRIPTION REGULATOR	VI.A
RSp0806	RS01901												TRANSMEMBRANE	VI.C
RSp0807	RS01900					-0,63	0,00						FERREDOXIN	I.G.1
RSp0808	RS01899					1,53	0,00						TRANSMEMBRANE	VI.C
RSp0809	RS01898												HYPOTHETICAL PROTEIN	VI.B
RSp0810	RS01897					-0,79	0,01	-0,91	0,05			atpC2	ATP SYNTHASE EPSILON CHAIN	I.E.2
RSp0811	RS01896												SIGNAL PEPTIDE TONB-DEPENDENT RECEPTOR PROTEIN	III.B.2
RSp0812	RS01895							-0,53	0,04				TRANSMEMBRANE	VI.C
RSp0813	RS01894			-1,97	0,01	0,60	0,05	5,62	0,00				TRANSMEMBRANE SIGNAL TRANSDUCTION PROTEIN EAL - GGDEF DOMAINS	VI.A
RSp0814	RS01893											mqo	OXIDOREDUCTASE MALATE:QUINONE OXIDOREDUCTASE	I.H.6
RSp0815	RS01892							-1,38	0,03				TRANSCRIPTION REGULATOR	VI.A
RSp0816	RS01891												TRANSCRIPTION REGULATOR	VI.A
RSp0817	RS01890												OUTER MEMBRANE DRUG EFFLUX LIPOPROTEIN	IV.E
RSp0818	RS01889												TRANSMEMBRANE DRUG EFFLUX PROTEIN	IV.D
RSp0819	RS01888												DRUG EFFLUX LIPOPROTEIN	IV.E
RSp0820	RS01887							-0,39	0,01				COG2706, 3-carboxymuconate cyclase	I
RSp0821	RS01886												TRANSMEMBRANE	VI.C
RSp0822	RS05373			-2,15	0,01	3,61	0,00	3,75	0,00				AvrPphF-RELATED PROTEIN	VI.B
RSp0823	RS05372					1,03	0,01					Rsp082:	TRANSCRIPTION REGULATOR TRANSCRIPTIONAL REGULATOR	VI.A
RSp0824	RS05371			-0,83	0,01	0,89	0,00	0,70	0,02				GLUCONOLACTONASE (D-GLUCONO-DELTA-LACTONE LACTONOHYDROLASE	I
RSp0825	RS05370	-2,77	0,00	-5,09	0,00	5,76	0,00	3,63	0,00	-1,92	0,00		COG0421, Spermidine synthase	I.F.15
RSp0826	RS05369												5-DEHYDRO-4-DEOXYGLUCARATE DEHYDRATASE	I.F
RSp0827	RS05368												COG1012, NAD-dependent aldehyde dehydrogenases	I
RSp0828	RS05367											gudP	TRANSMEMBRANE GLUCARATE TRANSPORTER (D-GLUCARATE PERMEASE)	IV.D
RSp0829	RS05366											gudD2	GLUCARATE DEHYDRATASE	I.I.4
RSp0830	RS05365											garD	D-GALACTARATE DEHYDRATASE (D-GALACTARATE DEHYDROGENASE)	I
RSp0831	RS05364												CONSERVED HYPOTHETICAL PROTEIN	VI.C
RSp0832	RS05363												COG3386, Gluconolactonase	I
RSp0833	RS05362			-0,64	0,00	1,78	0,00	2,20	0,00			pehC	TRANSMEMBRANE POLYGALACTURONASE	II.A.3
RSp0834	RS05361					2,04	0,00	1,27	0,01			exuT1	TRANSMEMBRANE HEXURONATE TRANSPORTER	IV.D
RSp0835	RS05360			0,80	0,04								BIFUNCTIONAL NMN ADENYLYLTRANSFERASE/NUDIX HYDROLASE : NICOTIN/	I.B.9
RSp0836	RS05359					0,44	0,00						NICOTINATE PHOSPHORIBOSYLTRANSFERASE-RELATED PROTEIN	I
RSp0837	RS05358			-2,70	0,00	1,00	0,00	3,65	0,00				HYPOTHETICAL PROTEIN	VI.D
RSp0838	RS05357												HYPOTHETICAL PROTEIN	VI.D
RSp0839	RS05562			-4,87	0,00	5,74	0,00	3,89	0,00				HYPOTHETICAL PROTEIN	VI.B
RSp0840	RS05355			-1,35	0,02	2,20	0,00	2,58	0,00				TRANSMEMBRANE METHYL-ACCEPTING CHEMOTAXIS TRANSDUCER	IV.C
RSp0841	RS05356	-4,14	0,00	-7,08	0,00	7,19	0,00	4,29	0,00				LIPOPROTEIN	VI.B
RSp0842	RS05354	-1,82	0,00	-3,98	0,00	4,11	0,00	3,77	0,00	-3,93	0,01		LEUCINE-RICH-REPEAT PROTEIN	VI.B
RSp0843	RS05353									-0,40	0,03		TRANSCRIPTION REGULATOR	VI.A
RSp0844	RS05352			-1,35	0,03	1,40	0,00	2,22	0,00				COG0702, PREDICTED NUCLEOSIDE-DIPHOSPHATE-SUGAR EPIMERASE	I
RSp0845	RS05351			-3,07	0,00	5,93	0,00	2,65	0,01				HYPOTHETICAL PROTEIN	VI.D
RSp0846	RS05350			-1,15	0,03	0,49	0,00						AWR TYPE III EFFECTOR FAMILY PROTEIN	VI.C
RSp0847	RS05349												AWR TYPE III EFFECTOR FAMILY PROTEIN	VI.C
RSp0848	RS01619			-4,65	0,00	5,63	0,00	3,99	0,00				HYPOTHETICAL PROTEIN	VI.D
RSp0849	RS01620							-1,20	0,02			prhI	TRANSCRIPTION REGULATOR 3-COMPARTIMENT SIGNAL TRANSDUCTION SYS	I.E.1
RSp0850	RS01621					-0,46	0,03					prhR	TRANSMEMBRANE 3-COMPARTIMENT SIGNAL TRANSDUCTION SYSTEM, COMI	I.E.1
RSp0851	RS01622											prhJ	TRANSCRIPTION REGULATOR TRANSCRIPTION REGULATOR PRHJ	I.E.1
RSp0852	RS01623			-1,19	0,01	2,26	0,00	1,37	0,02	2,77	0,00	hrpG	TRANSCRIPTION REGULATOR RESPONSE REGULATOR	I.E.1
RSp0853	RS01624			-5,53	0,01	6,91	0,00					hpaB	CONSERVED HYPOTHETICAL PROTEIN	VI.C
RSp0854	RS01625			-7,19	0,00	7,55	0,00	3,38	0,00			hrpZ	HRPY-RELATED PROTEIN	VI.B
RSp0855	RS01626	-3,65	0,00	-7,68	0,03	7,21	0,00	3,48	0,00	-4,27	0,00	hrpY	HRP PILUS SUBUNIT HRPY	IV.E.2
RSp0856	RS01627	-4,56	0,00	-6,67	0,00	8,34	0,00	4,27	0,00	-3,44	0,00	hrpX	HRPX	IV.E.2
RSp0857	RS01628	-1,96	0,01	-4,34	0,00	5,71	0,00	3,03	0,00			hrpW	TRANSMEMBRANE HRPW	IV.E.2
RSp0858	RS01629			-4,45	0,00	4,08	0,00	2,95	0,00			hrpV	HRPV	VI.C
RSp0859	RS01630	-3,91	0,00	-7,07	0,00	7,62	0,00	4,24	0,00	-4,64	0,00	hrcS	TRANSMEMBRANE HRP CONSERVED PROTEIN HRCS	IV.E.2
RSp0860	RS01631					7,89	0,00	5,12	0,00			hrcR	TRANSMEMBRANE HRP CONSERVED PROTEIN HRCR	IV.E.2
RSp0861	RS01632	-6,74	0,00			7,23	0,01	5,73	0,00			hrcQ	HRP CONSERVED PROTEIN HRCQ	IV.E.2
RSp0862	RS01633			-5,45	0,00	5,58	0,00	2,66	0,01			hpaP	HRP ASSOCIATED PROTEIN HpaP	VI.C
RSp0863	RS01634			-2,55	0,00	3,43	0,00	1,91	0,00			hrcV	TRANSMEMBRANE HRP CONSERVED PROTEIN HRCV	IV.E.2
RSp0864	RS01635					1,01	0,00					hrcU	TRANSMEMBRANE HRP CONSERVED PROTEIN HRCU	IV.E.2
RSp0865	RS01636	-3,36	0,00	-8,36	0,00	8,35	0,00	3,73	0,00	-7,03	0,00	hrpK	HRPK	IV.E.2
RSp0866	RS01637			-7,61	0,00	8,00	0,00	5,11	0,00			hrpJ	HRPJ PROTEIN	VI.C
RSp0867	RS01638	-1,77	0,01	-4,76	0,00	6,09	0,00	3,93	0,00	-2,52	0,00	hrcJ	TRANSMEMBRANE HRP CONSERVED LIPOPROTEIN HRCJ	IV.E.2
RSp0868	RS01639			-6,64	0,00	7,37	0,00	4,42	0,00			hrpH	HRPH	VI.C
RSp0869	RS01640			-5,43	0,00	6,28	0,00	3,75	0,00			hrpF	HRPF	IV.E.2
RSp0870	RS01641			-3,98	0,00	4,60	0,00	3,41	0,00			hrcN	HRP CONSERVED PROTEIN HRCN	IV.E.2
RSp0871	RS01642			-5,71	0,00	6,03	0,00	4,21	0,00			hrpD	HRPD	VI.C
RSp0872	RS01643			-4,02	0,00	5,88	0,00	3,04	0,00			hrcT	TRANSMEMBRANE HRP CONSERVED PROTEIN HRCT	IV.E.2
RSp0873	RS01644	-3,09	0,00	-5,10	0,00	7,09	0,00	3,92	0,00			hrpB	TRANSCRIPTION REGULATOR REGULATORY PROTEIN HRPB	I.E.1
RSp0874	RS01645	-0,85	0,00	-1,33	0,00	1,08	0,00	2,23	0,00	-0,98	0,00	hrcC	TRANSMEMBRANE HRP CONSERVED PROTEIN HRCC	IV.E.2
RSp0875	RS01646			-5,69	0,00	5,59	0,00	3,16	0,00			popC	POPC PROTEIN	VI.E
RSp0876	RS01647	-3,94	0,00	-8,93	0,00	8,89	0,00	3,84	0,00	-6,10	0,00	popB	POPB PROTEIN	VI.E
RSp0877	RS01648	-5,60	0,00	-8,95	0,00	8,14	0,00	4,73	0,00	-5,62	0,00	popA	POPA PROTEIN	VI.E
RSp0878	RS01649							0,63	0,00	1,02	0,05	prhA	SIGNAL PEPTIDE OUTER MEMBRANE RECEPTOR PROTEIN PRHA	I.E.1
RSp0879	RS01650			-1,79	0,03	3,20	0,00	2,72	0,00				HYPOTHETICAL PROTEIN	VI.D
RSp0880	RS01651					1,35	0,00					pglA	SIGNAL PEPTIDE POLYGALACTURONASE PRECURSOR (PECTINASE)	II.A.3
RSp0881	RS01652					-0,42	0,02	2,81	0,00				TRANSMEMBRANE GLYCOSYL HYDROLASE	VI.B
RSp0882	RS01653			-5,23	0,00	6,16	0,00	4,51	0,00				HYPOTHETICAL PROTEIN	VI.D
RSp0883	RS01654	-4,56	0,02			-1,36	0,00	1,30	0,00	3,11	0,00		HYPOTHETICAL PROTEIN	VI.B
RSp0884	RS01655	-0,45	0,02	0,48	0,01			0,60	0,01				TRANSMEMBRANE	VI.B

RSp0885	RS01656			2,06 0,00	2,45 0,00			HYPOTHETICAL PROTEIN	VI.D
RSp0886	RS01657						glnA2	GLUTAMINE SYNTHETASE	I.A.18
RSp0887	RS01658				-0,26 0,02			TRANSCRIPTION REGULATOR TRANSCRIPTIONAL REGULATOR	VI.A
RSp0888	RS01659		-1,41 0,00	2,23 0,00			cmtC	OXIDOREDUCTASE 2,3-DIHYDROXY-p-CUMATE-3,4-DIOXYGENASE	I.I.4
RSp0889	RS01660						cmtD	HCOMODA DECARBOXYLASE	I.I.4
RSp0890	RS01661						dmpC	OXIDOREDUCTASE 2-HYDROXYMUCONIC SEMIALDEHYDE DEHYDROGENASE	I.I.4
RSp0891	RS01662							COG3971, 2-keto-4-pentenoate hydratase	I.I.4
RSp0892	RS01663							COG3971, 2-keto-4-pentenoate hydratase	I.I.4
RSp0893	RS01664			-0,39 0,00			dmpI	4-OXALOCROTONATE ISOMERASE	I.I.4
RSp0894	RS01665			-0,50 0,04			dmpF	OXIDOREDUCTASE ACETALDEHYDE DEHYDROGENASE	I.I.4
RSp0895	RS01666						dmpG	4-HYDROXY-2-OXOVALERATE ALDOLASE	I.I.4
RSp0896	RS01667							TRANSMEMBRANE 4-HYDROXYBENZOATE TRANSPORTER	IV.D.3
RSp0897	RS01668			-0,68 0,02	-0,60 0,02			SIGNAL PEPTIDE	VI.C
RSp0898	RS01669	-3,53 0,02					dsbA2	SIGNAL PEPTIDE THIOL:DISULFIDE INTERCHANGE PROTEIN	II.B.7
RSp0899	RS01670			0,66 0,03				COG3971, 2-keto-4-pentenoate hydratase	I.I.4
RSp0900	RS01671	-2,92 0,00	-6,68 0,00	6,82 0,00	6,48 0,02	-4,13 0,01	popF2	SECRETED PROTEIN POPF2	IV.E.2
RSp0901	RS01672							HYPOTHETICAL PROTEIN	VI.D
RSp0902	RS01673							INTEGRASE/RECOMBINASE	V.A
RSp0903	RS01674			-0,58 0,03		-0,30 0,04		TRANSMEMBRANE	VI.D
RSp0904	RS01675			-0,75 0,02				OXIDOREDUCTASE COG2303, Choline dehydrogenase and related flavoproteins	I
RSp0905	RS01676			-0,74 0,01				TRANSMEMBRANE	VI.D
RSp0906	RS01677	-0,55 0,03		-0,91 0,00	0,79 0,05	2,54 0,00		SIGNAL PEPTIDE	VI.B
RSp0907	RS01678							TRANSMEMBRANE	VI.B
RSp0908	RS01680							OXIDOREDUCTASE COG2141, Coenzyme F420-dependent N5,N10-methylene tetral	I
RSp0909	RS01681							COG2761, Predicted dithiol-disulfide isomerase involved in polyketide biosynthesis	I
RSp0910	RS01682			-0,69 0,01				TRANSCRIPTION REGULATOR	VI.A
RSp0911	RS01683			-0,80 0,01				OXIDOREDUCTASE SALICYLATE HYDROXYLASE	I.I.4
RSp0912	RS01684							TRANSCRIPTION REGULATOR TRANSCRIPTIONAL REGULATOR	VI.A
RSp0913	RS01685							COG0673, Predicted dehydrogenases and related proteins	I
RSp0914	RS01686				1,77 0,00			LRR-GALA FAMILY TYPE III EFFECTOR PROTEIN (GALA 1)	VI.C
RSp0915	RS01687							HYPOTHETICAL PROTEIN	V.A
RSp0916	RS01688							VACJ-RELATED LIPOPROTEIN	III.A.2
RSp0917	RS01694			-0,55 0,04				TRANSCRIPTION REGULATOR TWO COMPONENT RESPONSE REGULATOR	VI.A
RSp0918	RS05419		0,70 0,04					TRANSPOSASE, truncated	V.A
RSp0919	RS01690			0,45 0,00	1,43 0,01			HYPOTHETICAL PROTEIN	VI.D
RSp0920	RS01691		0,41 0,05					COG4688: Uncharacterized protein conserved in bacteria	VI.B
RSp0921	RS01692		0,76 0,01					INTEGRASE/RECOMBINASE	V.A
RSp0922	RS01693				-0,77 0,03			INTEGRASE / RECOMBINASE	V.A
RSp0923	RS05412			0,49 0,03				HYPOTHETICAL PROTEIN	VI.D
RSp0924	RS05410				1,28 0,00	1,31 0,00		HYPOTHETICAL PROTEIN	VI.D
RSp0925	RS05408							SIGNAL PEPTIDE	VI.C
RSp0926	RS05407		0,55 0,00	-0,34 0,03				OUTER MEMBRANE CATION EFFLUX PROTEIN	IV.G
RSp0927	RS05406		0,31 0,01		0,80 0,02			TRANSMEMBRANE CATION EFFLUX SYSTEM PROTEIN	IV.G
RSp0928	RS05405			0,99 0,00				TRANSMEMBRANE CATION EFFLUX SYSTEM	IV.D
RSp0929	RS05404							SIGNAL PEPTIDE	VI.B
RSp0930	RS05403			1,71 0,00				SKWP PROTEIN 3	VI.C
RSp0931	RS05402							ABC TRANSPORTER AMINO-ACID-BINDING PERIPLASMIC PROTEIN	IV.D.2
RSp0932	RS05401							ABC TRANSPORTER AMINO-ACID ATP-BINDING PROTEIN	IV.D.2
RSp0933	RS05400			-0,35 0,01				ABC TRANSPORTER AMINO ACID TRANSMEMBRANE PROTEIN	IV.D.2
RSp0934	RS05399							ABC TRANSPORTER AMINO ACID TRANSMEMBRANE PROTEIN	IV.D.2
RSp0935	RS05398							OXIDOREDUCTASE	I.I
RSp0936	RS05397			-0,34 0,01				CONSERVED HYPOTHETICAL PROTEIN	VI.C
RSp0937	RS05396			-0,56 0,01				CONSERVED HYPOTHETICAL PROTEIN	VI.C
RSp0938	RS05395							COG1984, Allophanate hydrolase subunit 2	I
RSp0939	RS05394							COG2049, Allophanate hydrolase subunit 1	I
RSp0940	RS05393				-0,77 0,01		accC2	BIOTIN CARBOXYLASE	I.B.1
RSp0941	RS05392						accB2	BIOTIN CARBOXYL CARRIER PROTEIN OF ACETYL-COA CARBOXYLASE (BCCF	I.B.17
RSp0942	RS05391			-0,41 0,02			nac	TRANSCRIPTION REGULATOR NITROGEN ASSIMILATION REGULATORY PROTE	I.F.5
RSp0943	RS05390		-1,25 0,02	3,09 0,00			aatA2	ASPARTATE AMINOTRANSFERASE A	I.A.19
RSp0944	RS05389							TRANSFERASE	VI.C
RSp0945	RS05388							OXIDOREDUCTASE DEHYDROGENASE	I.I
RSp0946	RS05387							GLUCONATE KINASE	I.F.4
RSp0947	RS05386						idnO	OXIDOREDUCTASE GLUCONATE 5-DEHYDROGENASE	I.I.4
RSp0948	RS05385						idnD	OXIDOREDUCTASE L-IDONATE 5-DEHYDROGENASE	I.I.4
RSp0949	RS05384						pntAa2	OXIDOREDUCTASE NAD(P) TRANSHYDROGENASE (ALPHA SUBUNIT PART 1)	I.F.18
RSp0950	RS05383						pntAb2	TRANSMEMBRANE TRANSMEMBRANE NAD(P) TRANSHYDROGENASE (ALPHA S	I.F.18
RSp0951	RS05380				-1,69 0,03	-1,20 0,00	pntB2a	TRANSMEMBRANE NAD(P) TRANSHYDROGENASE SUBUNIT BETA (PART 1)	I.F.18
RSp0954	RS05377						pntB2b	OXIDOREDUCTASE NAD(P) TRANSHYDROGENASE SUBUNIT BETA (PART 2)	I.F.18
RSp0955	RS05376							SIGNAL PEPTIDE PORIN	IV.D
RSp0956	RS02287				-0,99 0,04			HYPOTHETICAL PROTEIN	VI.D
RSp0957	RS02288		-0,63 0,03	1,02 0,00	2,46 0,00	1,20 0,00		HYPOTHETICAL PROTEIN	VI.D
RSp0958	RS02289							COG2846, Regulator of cell morphogenesis and NO signaling	IV.A
RSp0959	RS02290							TRANSCRIPTION REGULATOR SIGMA-54 INTERACTING PROTEIN	VI.A
RSp0960	RS02291			-0,46 0,05				COG0451, Nucleoside-diphosphate-sugar epimerases	I
RSp0961	RS02292						kbl	2-AMINO-3-KETOBUTYRATE COENZYME A LIGASE	I.F
RSp0962	RS02293							TRANSCRIPTION REGULATOR	VI.A
RSp0963	RS02294		0,48 0,00	-0,44 0,05			nrdD	OXIDOREDUCTASE RIBONUCLEOTIDE REDUCTASE	I.F.2
RSp0964	RS02295							HYPOTHETICAL PROTEIN	VI.B
RSp0965	RS02296			-0,21 0,02			nrdG	TRANSMEMBRANE RIBONUCLEOTIDE REDUCTASE ACTIVATING PROTEIN	I.D
RSp0966	RS02297							TRANSCRIPTION REGULATOR	VI.A
RSp0967	RS02298			-0,57 0,01				LIPOPROTEIN COG4273, Uncharacterized conserved protein	VI.C
RSp0968	RS02299							COG3154, Putative lipid carrier protein	I
RSp0969	RS02300		1,68 0,02					COG0826, Collagenase and related proteases	II.A.4
RSp0970	RS02301							COG0826, Collagenase and related proteases	II.A.4
RSp0971	RS02303							TRANSMEMBRANE	VI.C
RSp0972	RS02304						narK1	TRANSMEMBRANE NITRITE/NITRATE TRANSPORTER PROTEIN	IV.D.1
RSp0973	RS02305						narK2	TRANSMEMBRANE NITRITE/NITRATE TRANSPORTER PROTEIN	IV.D.1
RSp0974	RS02306		1,17 0,03				narG	OXIDOREDUCTASE RESPIRATORY NITRATE REDUCTASE ALPHA CHAIN	I.H.2
RSp0975	RS02307		0,97 0,04				narH	OXIDOREDUCTASE RESPIRATORY NITRATE REDUCTASE	I.H.2
RSp0976	RS02308						narJ	OXIDOREDUCTASE RESPIRATORY NITRATE REDUCTASE	I.H.2
RSp0977	RS02309						narI	TRANSMEMBRANE RESPIRATORY NITRATE REDUCTASE	I.H.2
RSp0978	RS02310						mobB1	MOLYBDOPTERIN-GUANINE DINUCLEOTIDE BIOSYNTHESIS PROTEIN	I.F.9
RSp0979	RS02311				-0,93 0,01		narX	TRANSCRIPTION REGULATOR TRANSMEMBRANE NITRATE/NITRITE SENSOR K	I.H.2
RSp0980	RS02312						narL	TRANSCRIPTION REGULATOR NITRATE/NITRITE RESPONSE REGULATOR PRO	I.H.2
RSp0981	RS02313							TRANSMEMBRANE	VI.C
RSp0982	RS02314				-1,42 0,00			TRANSMEMBRANE	VI.D
RSp0983	RS02316			0,86 0,04		2,38 0,02		TRANSCRIPTION REGULATOR	VI.A
RSp0984	RS02315	1,05 0,02					thiE2	THIAMINE-PHOSPHATE PYROPHOSPHORYLASE	I.B.8
RSp0985	RS02317							TRANSMEMBRANE TRANSPORTER	IV.D
RSp0986	RS02318			-0,47 0,00			gbd2	OXIDOREDUCTASE NAD-DEPENDENT 4-HYDDOXYBUTYRATE DEHYDROGENAS	I.I.4
RSp0987	RS02319							TRANSMEMBRANE	VI.B
RSp0988	RS02320			-0,68 0,05				TRANSMEMBRANE	VI.C
RSp0989	RS02321							TRANSMEMBRANE	VI.B
RSp0992	RS02324		-0,41 0,01	-0,77 0,02				SIGNAL PEPTIDE	VI.C
RSp0993	RS02325							TRANSMEMBRANE CYTOCHROME C OXIDASE SUBUNIT	I.H
RSp0994	RS02326						uvrA2	ABC TRANSPORTER EXCINUCLEASE ABC SUBUNIT A (DNA REPAIR ATP-BINDI	II.B.8
RSp0995	RS02327						dctA2	TRANSMEMBRANE C4-DICARBOXYLATE TRANSPORT PROTEIN	IV.D.3
RSp0996	RS02328		-0,50 0,02		0,83 0,03		ttuC2	OXIDOREDUCTASE TARTRATE DEHYDROGENASE	I.I.4
RSp0997	RS02329							TRANSCRIPTION REGULATOR	VI.A
RSp0998	RS02331							TRANSMEMBRANE COG0705, Uncharacterized membrane protein	VI.C
RSp0999	RS02332					0,80 0,01		HYPOTHETICAL PROTEIN	VI.D
RSp1000	RS02333					0,95 0,00	tek2	TEK-RELATED PROTEIN	VI.C

RSp1001	RS02334							0,96	0,01				SIGNAL PEPTIDE	VI.D
RSp1002	RS02335							0,76	0,04			tek	SIGNAL PEPTIDE TEK PROTEIN	VI.C
RSp1003	RS02336					0,42	0,05					xpsR	TRANSCRIPTION REGULATOR TRANSCRIPTION REGULATOR XPSR	III.A.1
RSp1004	RS02337							-0,62	0,01				TRANSMEMBRANE DTDP-GLUCOSE 4,6-DEHYDRATASE	II.B.2
RSp1005	RS02338					0,43	0,03	-1,02	0,00	-1,37	0,00		TRANSMEMBRANE	VI.B
RSp1006	RS02340			0,20	0,05	0,19	0,02			-0,41	0,00		EPS AMINOTRANSFERASE	III.A.1
RSp1007	RS02341												ACETYL TRANSFERASE	III.A.1
RSp1008	RS02342					0,37	0,00						TRANSMEMBRANE GLYCOSYL TRANSFERASE	III.A.1
RSp1009	RS02343					0,42	0,01			-0,38	0,03		GLYCOSYLTRANSFERASE	III.A.1
RSp1010	RS02344												CONSERVED HYPOTHETICAL PROTEIN	VI.C
RSp1011	RS02345					0,31	0,01						OXIDOREDUCTASE COG1063, Threonine dehydrogenase and related Zn-dependent	VI.C
RSp1012	RS02346					0,40	0,03			-0,50	0,00		COG0110, Acetyltransferase	VI.C
RSp1013	RS02347	-0,46	0,00			0,26	0,01	-0,61	0,01	-0,92	0,03		COG0438, Glycosyltransferase	VI.C
RSp1014	RS02348					0,32	0,05					epsF	TRANSMEMBRANE EPS I POLYSACCHARIDE EXPORT INNER MEMBRANE PROT	III.A.1
RSp1015	RS02349											epsE	TRANSMEMBRANE EPS I POLYSACCHARIDE EXPORT INNER MEMBRANE PROT	III.A.1
RSp1016	RS02350											epsD	OXIDOREDUCTASE NDP-N-ACETYL-D-GALACTOSAMINURONIC ACID DEHYDRO	II.B
RSp1017	RS02355					0,52	0,00			-0,63	0,02	epsC	UDP-N-ACETYLGLUCOSAMINE 2-EPIMERASE	III.A.1
RSp1018	RS02356			0,27	0,00					-0,63	0,01	epsB	TRANSMEMBRANE EPS I POLYSACCHARIDE EXPORT PROTEIN	III.A.1
RSp1019	RS02353			0,34	0,02	0,43	0,01			-0,81	0,00	epsP	LOW MOLECULAR WEIGHT PROTEIN-TYROSINE-PHOSPHATASE	III.A.1
RSp1020	RS02354			0,30	0,02			-0,38	0,02			epsA	TRANSMEMBRANE EPS I POLYSACCHARIDE EXPORT OUTER MEMBRANE PRO	III.A.1
RSp1021	RS02358							-0,71	0,01				HYPOTHETICAL PROTEIN	VI.D
RSp1022	RS02359			-5,12	0,00	6,10	0,00	2,87	0,00				HYPOTHETICAL PROTEIN	VI.D
RSp1023	RS02360			0,34	0,02	-0,43	0,01	0,99	0,02				TRANSCRIPTION REGULATOR RESPONSE REGULATOR	VI.A
RSp1024	RS02361					1,86	0,00						AWR TYPE III EFFECTOR FAMILY PROTEIN	VI.C
RSp1025	RS02362			-1,07	0,00	0,99	0,00	1,16	0,00				TRANSMEMBRANE AMINO-ACID EFFLUX PROTEIN	IV.D.2
RSp1026	RS02363							1,02	0,05				SIGNAL PEPTIDE	VI.C
RSp1027	RS02364					-0,45	0,00						TRANSMEMBRANE METHYL-ACCEPTING CHEMOTAXIS TRANSDUCER	IV.C
RSp1028	RS02365					-0,54	0,03	-1,72	0,02				HYPOTHETICAL PROTEIN	VI.D
RSp1029	RS02366												OXIDOREDUCTASE TRANSMEMBRANE ACYL-CoA DESATURASE (FATTY ACID D	I.I.1
RSp1030	RS02367					-0,33	0,04						COG3687, PREDICTED METAL- DEPENDENT HYDROLASE	I.I
RSp1031	RS02368			-1,94	0,01	3,50	0,00	2,70	0,01				HYPOTHETICAL PROTEIN	VI.D
RSp1032	RS02369			-1,07	0,00	1,68	0,00						CONSERVED HYPOTHETICAL PROTEIN	VI.C
RSp1033	RS02370							-0,58	0,01	-0,94	0,00		COG2916, DNA-binding protein H-NS - related protein	VI.A
RSp1034	RS02371					-0,71	0,00						SIGNAL PEPTIDE	VI.C
RSp1035	RS02372					-0,60	0,01	-1,05	0,00				CONSERVED HYPOTHETICAL PROTEIN	VI.C
RSp1036	RS02373					-0,52	0,00	-0,51	0,04				CONSERVED HYPOTHETICAL PROTEIN	VI.C
RSp1037	RS02374			-1,69	0,04								TRANSMEMBRANE	VI.C
RSp1038	RS02375					-0,56	0,00						TRANSMEMBRANE LIPOPROTEIN	VI.D
RSp1039	RS02376			0,51	0,04								TRANSMEMBRANE OUTER MEMBRANE CHANNEL LIPOPROTEIN	IV.D
RSp1040	RS02377					-0,38	0,01						TRANSMEMBRANE CATION EFFLUX SYSTEM PROTEIN	IV.D.4
RSp1041	RS02378			0,93	0,00	-0,61	0,01						TRANSMEMBRANE CATION TRANSPORTER PROTEIN	IV.D.4
RSp1042	RS02379					-0,37	0,03	-0,56	0,05				TRANSCRIPTION REGULATOR TWO-COMPONENT RESPONSE REGULATOR	VI.A
RSp1043	RS02380					-0,46	0,02	-0,72	0,03				TRANSCRIPTION REGULATOR TWO-COMPONENT SENSOR HISTIDINE KINASE	VI.A
RSp1044	RS02381					-0,40	0,02					cnrT	TRANSMEMBRANE COBALT-NICKEL-RESISTANCE SYSTEM PROTEIN	IV.G
RSp1045	RS02382												HYPOTHETICAL PROTEIN	VI.B
RSp1046	RS02383							-0,97	0,03				TRANSMEMBRANE AMINO ACID TRANSPORTER PROTEIN	IV.D.2
RSp1047	RS02384					1,17	0,00						TRANSMEMBRANE TRANSPORTER PROTEIN	IV.D
RSp1048	RS02385					0,56	0,00						OXIDOREDUCTASE FORMATE DEHYDROGENASE	I.H.2
RSp1049	RS02386					0,49	0,00						HYPOTHETICAL PROTEIN	VI.D
RSp1050	RS02387											fdhD	FORMATE DEHYDROGENASE ASSOCIATED PROTEIN (FDHD PROTEIN)	I.H.2
RSp1051	RS02388												SIGNAL TRANSDUCTION PROTEIN EAL DOMAIN	VI.A
RSp1052	RS02389											kup1	TRANSMEMBRANE POTASSIUM UPTAKE PROTEIN	IV.D.4
RSp1053	RS02390					0,63	0,00	-0,82	0,01	-0,93	0,03	cspD2	TRANSCRIPTION REGULATOR COLD SHOCK PROTEIN ACTIVATOR	VI.A
RSp1054	RS02391	0,24	0,03					-0,50	0,03				TRANSCRIPTION REGULATOR COLD SHOCK PROTEIN ACTIVATOR	VI.A
RSp1055	RS02392					0,36	0,03	-1,06	0,02				OXIDOREDUCTASE TRANSMEMBRANE ALDEHYDE DEHYDROGENASE	I.I.4
RSp1056	RS02393					-0,44	0,00						BETA-ALANINE--PYRUVATE AMINOTRANSFERASE (OMEGA-AMINO ACID--PYRU	I.F.14
RSp1057	RS02394												TRANSCRIPTION REGULATOR	VI.A
RSp1058	RS02395					-0,48	0,00					adc	ACETOACETATE DECARBOXYLASE	I
RSp1059	RS02396					-0,44	0,00	0,97	0,04				OXIDOREDUCTASE DEHYDROGENASE/REDUCTASE	I
RSp1060	RS02397							0,56	0,03				TRANSMEMBRANE COG1752, Predicted esterase of the alpha-beta hydrolase super	I
RSp1061	RS02398												OXIDOREDUCTASE COG0673, Predicted dehydrogenases and related proteins	I
RSp1062	RS02469					-1,81	0,03	-0,94	0,00				FERRISIDEROPHORE RECEPTOR	IV.D.5
RSp1063	RS02470			0,61	0,01	-0,40	0,02						SIGNAL PEPTIDE	VI.D
RSp1064	RS02472												TRANSMEMBRANE	VI.C
RSp1065	RS02471												SIGNAL PEPTIDE	VI.B
RSp1066	RS03027	0,62	0,01										CONSERVED HYPOTHETICAL PROTEIN	VI.C
RSp1067	RS06033												CONSERVED HYPOTHETICAL PROTEIN	VI.C
RSp1068	RS02476												CONSERVED HYPOTHETICAL PROTEIN	VI.C
RSp1069	RS02556							-0,72	0,05				TRANSMEMBRANE	VI.D
RSp1070	RS02559			0,60	0,04								TRANSMEMBRANE	VI.E
RSp1071	RS02473												HEMAGGLUTININ-RELATED PROTEIN	VI.B
RSp1072	RS02541					-0,36	0,01			-1,77	0,03		CONSERVED HYPOTHETICAL PROTEIN	VI.C
RSp1073	RS02477									-0,51	0,03		HEMAGGLUTININ-RELATED PROTEIN	VI.C
RSp1074	RS02573												HEMOLYSIN ACTIVATING-LIKE PROTEIN	IV.E.2
RSp1075	RS02577			0,65	0,00					-0,99	0,02		ACTIVATION/SECRETION PROTEIN	IV.E.2
RSp1076	RS02580					0,99	0,02						OXIDOREDUCTASE GLUCOSE-FRUCTOSE OXIDOREDUCTASE	I.F
RSp1077	RS02582					-0,78	0,00	-0,58	0,01				TRANSMEMBRANE	VI.D
RSp1078	RS02585					-0,78	0,00						LIPOPROTEIN	VI.B
RSp1079	RS02586					-0,22	0,03						TRANSCRIPTION REGULATOR SIGMA-54 INTERACTING PROTEIN	VI.A
RSp1080	RS02587												TRANSMEMBRANE	VI.C
RSp1081	RS02588							-0,37	0,01				SIGNAL PEPTIDE	VI.D
RSp1082	RS02589					-0,49	0,03					tadD2	TRANSMEMBRANE PILUS ASSEMBLY PROTEIN TADD	III.A.2
RSp1083	RS02590	0,32	0,01									tadC2	TRANSMEMBRANE PILUS ASSEMBLY PROTEIN TADC	III.A.2
RSp1084	RS02591											tadB2	TRANSMEMBRANE PILUS ASSEMBLY PROTEIN TADB	III.A.2
RSp1085	RS02592					-0,46	0,00					cpaF2	SECRETION ATPase	III.A.2
RSp1086	RS02593											cpaE2	PILUS ASSEMBLY PROTEIN	III.A.2
RSp1087	RS02594	0,41	0,05			-0,27	0,02					cpaC2	SIGNAL PEPTIDE PILUS ASSEMBLY PROTEIN, SECRETIN CPAC	III.A.2
RSp1088	RS02597												CONSERVED HYPOTHETICAL PROTEIN	VI.C
RSp1089	RS02596											cpaB2	SIGNAL PEPTIDE COG3745, Flp pilus assembly protein	III.A.2
RSp1090	RS02595											tadG2	TRANSMEMBRANE COG4961, FLP PILUS ASSEMBLY PROTEIN	III.A.2
RSp1091	RS02612							0,91	0,00			cpaA2	TRANSMEMBRANE PREPILIN PEPTIDASE	III.A.2
RSp1092	RS02598									0,96	0,00		PILIN	III.A.2
RSp1093	RS02599												TRANSMEMBRANE	VI.C
RSp1094	RS02600					1,38	0,00						TRANSMEMBRANE	VI.C
RSp1095	RS02601					-0,53	0,00			0,90	0,00		SIGNAL PEPTIDE ACTIVATION/SECRETION PROTEIN	IV.E.2
RSp1096	RS02602					-0,61	0,00	-0,80	0,01	-1,20	0,00		TRANSCRIPTION REGULATOR	VI.A
RSp1097	RS02603					-0,37	0,01						TRANSMEMBRANE SIGNAL TRANSDUCTION PROTEIN EAL - GGDEF DOMAINS	VI.A
RSp1098	RS02604					-0,51	0,01	-1,64	0,00	-2,13	0,01		TRANSCRIPTION REGULATOR HNS-LIKE PROTEIN	VI.A
RSp1099	RS02605												TRANSMEMBRANE METHYL-ACCEPTING CHEMOTAXIS TRANSDUCER	IV.C
RSp1100	RS02606												TRANSMEMBRANE LIPOPROTEIN	VI.B
RSp1101	RS02607			0,35	0,01	-0,44	0,00					galE	UDP-GLUCOSE 4-EPIMERASE	I.I.4
RSp1102	RS02608					-0,68	0,00						INTEGRASE/RECOMBINASE	V.A
RSp1103	RS02609												HYPOTHETICAL PROTEIN	VI.D
RSp1104	RS02610									1,71	0,04	otsB	TREHALOSE-PHOSPHATASE	IV.F.2
RSp1105	RS02613											otsA	ALPHA,ALPHA-TREHALOSE-PHOSPHATE SYNTHASE	IV.F.2
RSp1106	RS02614					-1,64	0,00	-0,53	0,04				TRANSCRIPTION REGULATOR	VI.A
RSp1107	RS02615	0,23	0,05			-1,66	0,00					ohr	ORGANIC HYDROPEROXIDE RESISTANCE PROTEIN	IV.G.4
RSp1108	RS02616	3,10	0,02			-1,43	0,01						SIGNAL PEPTIDE LIPASE	I.I.1
RSp1109	RS02617					-0,74	0,00						CONSERVED HYPOTHETICAL PROTEIN	VI.C
RSp1110	RS02618	-0,89	0,01	-0,58	0,05	-0,35	0,02						TRANSCRIPTION REGULATOR	VI.A
RSp1111	RS02619					-1,02	0,00						LIPASE	I.I.1
RSp1112	RS02620	-2,47	0,03	0,74	0,03	-0,43	0,03						TRANSMEMBRANE TRANSMEMBRANE MULTIDRUG EFFLUX SYSTEM PROTEIN	IV.G.2

RSp1113	RS02621				1,07 0,04			TRANSMEMBRANE TRANSMEMBRANE MULTIDRUG-EFFLUX SYSTEM LIPOPRO	IV.D
RSp1114	RS05506		0,94 0,05		0,88 0,02			OUTER-MEMBRANE DRUG EFFLUX PROTEIN	IV.D
RSp1115	RS05505							TRANSCRIPTION REGULATOR	VI.A
RSp1116	RS05504							LOW SPECIFICITY L-THREONINE ALDOLASE	I.I.3
RSp1117	RS05502			-0,40 0,05		-1,27 0,01		TRANSMEMBRANE	VI.D
RSp1118	RS05501			-0,54 0,04		-0,73 0,04		CONSERVED HYPOTHETICAL PROTEIN	VI.C
RSp1119	RS05500			-0,75 0,00				TRANSCRIPTION REGULATOR	VI.A
RSp1120	RS05499							COG2030, Acyl dehydratase	I
RSp1121	RS05498							TRANSCRIPTION REGULATOR	VI.A
RSp1122	RS05497				0,89 0,01	1,03 0,00		COG2220, Predicted Zn-dependent hydrolases of the beta-lactamase fold	I.I
RSp1123	RS05496				3,04 0,00			OXIDOREDUCTASE KOG1208, Dehydrogenases with different specificities (related tc	I
RSp1124	RS05494	-2,66 0,00		-1,14 0,00	3,95 0,00	2,61 0,00		OXIDOREDUCTASE NADP-DEPENDENT ZINC-TYPE ALCOHOL DEHYDROGENAS	I
RSp1125	RS05495	-3,26 0,00		-1,25 0,00	1,86 0,00	1,96 0,02		HYPOTHETICAL PROTEIN	VI.D
RSp1126	RS05493			-0,43 0,02				TRANSCRIPTION REGULATOR	VI.A
RSp1127	RS05492		0,50 0,02	-0,41 0,01				ABC TRANSPORTER TRANSMEMBRANE PROTEIN	IV.D
RSp1128	RS05491			-0,52 0,00			arsC	ARSENATE REDUCTASE (ARSENICAL PUMP MODIFIER)	IV.G
RSp1129	RS05489	-0,92 0,03		-0,52 0,00	1,00 0,00	1,10 0,04	arsR	TRANSCRIPTION REGULATOR ARSENICAL RESISTANCE OPERON REPRESSOF	IV.G
RSp1130	RS05488		-2,38 0,00	5,24 0,00	4,06 0,00			HYPOTHETICAL PROTEIN	VI.D
RSp1131	RS05485							HYPOTHETICAL PROTEIN	VI.D
RSp1132	RS05487							CONSERVED HYPOTHETICAL PROTEIN	VI.C
RSp1133	RS05510							HYPOTHETICAL PROTEIN	VI.D
RSp1134	RS05509			-0,29 0,02	-0,87 0,03			HYPOTHETICAL PROTEIN	VI.D
RSp1137	RS05482							TRANSMEMBRANE RHS-RELATED PROTEIN	VI.C
RSp1138	RS05481		1,02 0,00	-0,57 0,03				CONSERVED HYPOTHETICAL PROTEIN	VI.C
RSp1139	RS05480		0,82 0,01					VGR-RELATED PROTEIN	VI.C
RSp1140	RS05478		0,61 0,05			-0,60 0,00		ABC TRANSPORTER SUBSTRATE-BINDING PERIPLASMIC PROTEIN (PBP)	IV.D
RSp1141	RS05474			-1,26 0,00	-0,73 0,04			TRANSCRIPTION REGULATOR	VI.A
RSp1142	RS05470			-0,49 0,01				TRANSCRIPTION REGULATOR	VI.A
RSp1143	RS05469			-0,42 0,03			glsA	GLUTAMINASE A	I.A.19
RSp1144	RS05467				-0,65 0,05			ABC TRANSPORTER COG4149, ABC-type molybdate transport system, permease cc	IV.D
RSp1145	RS05462			-0,50 0,01				ABC TRANSPORTER ATP-BINDING PROTEIN	IV.D
RSp1146	RS05458							TRANSCRIPTION REGULATOR	VI.A
RSp1147	RS05455		0,78 0,01	-0,30 0,01		-2,32 0,00		OXIDOREDUCTASE COG2085, Predicted dinucleotide-binding enzymes	I
RSp1148	RS05450							SIGNAL PEPTIDE COG0596, Predicted hydrolases or acyltransferases (alpha/beta hy	I
RSp1150	RS05437			2,41 0,00	2,62 0,04			HYPOTHETICAL PROTEIN	VI.D
RSp1151	RS05436							COG1942, Uncharacterized protein, 4-oxalocrotonate tautomerase homolog	VI.C
RSp1153	RS05434			-0,21 0,05				TRANSCRIPTION REGULATOR	VI.A
RSp1154	RS05433							TRANSMEMBRANE	VI.C
RSp1155	RS05432							SIGNAL TRANSDUCTION PROTEIN EAL - GGDEF DOMAINS	VI.A
RSp1156	RS05430							SIGNAL PEPTIDE	VI.D
RSp1157	RS05428		0,69 0,03					TRANSCRIPTION REGULATOR	VI.A
RSp1158	RS05427							COG3813, Uncharacterized protein conserved in bacteria	VI.C
RSp1159	RS05426							COG0589, Universal stress protein UspA and related nucleotide-binding proteins	IV.G
RSp1160	RS05425		0,33 0,00	-0,37 0,02	-0,57 0,05			SIGNAL PEPTIDE COG3678, P pilus assembly/Cpx signaling pathway, periplasmic inl	VI.C
RSp1161	RS05646			-0,23 0,01				TRANSCRIPTION REGULATOR TWO-COMPONENT RESPONSE REGULATOR	VI.A
RSp1162	RS05645				-0,37 0,04			TRANSCRIPTION REGULATOR TWO-COMPONENT SENSOR HISTIDINE KINASE	VI.A
RSp1163	RS05644			-0,58 0,02	-1,92 0,01			HYPOTHETICAL PROTEIN	VI.D
RSp1164	RS05643			-0,46 0,04				TRANSCRIPTION REGULATOR	VI.A
RSp1165	RS05642			-0,57 0,02				SIGNAL PEPTIDE	VI.B
RSp1166	RS05641							SIGNAL PEPTIDE COG4727 , Uncharacterized protein conserved in bacteria	VI.C
RSp1167	RS05823			1,08 0,00				COG2303, BetA, Choline dehydrogenase and related flavoproteins AND KOG2624, T	I
RSp1168	RS05639			0,51 0,05				TRANSMEMBRANE	VI.C
RSp1169	RS05059			0,54 0,00				CONSERVED HYPOTHETICAL PROTEIN	VI.C
RSp1170	RS05060				-0,59 0,04			SIGNAL PEPTIDE	VI.C
RSp1171	RS05061			-0,47 0,04				TRANSMEMBRANE	VI.C
RSp1172	RS05062		0,97 0,00		-0,44 0,04			TRANSMEMBRANE	VI.C
RSp1173	RS05063			-0,26 0,04	-0,46 0,03			TRANSMEMBRANE COG1858, Cytochrome c peroxidase	I
RSp1174	RS05064		0,68 0,01				acpA	ACID PHOSPHATASE	I.F
RSp1175	RS05065			-0,46 0,00				CONSERVED HYPOTHETICAL PROTEIN	VI.C
RSp1176	RS05066	0,04 0,04			2,45 0,00			HYPOTHETICAL PROTEIN	VI.D
RSp1177	RS05067							TRANSMEMBRANE	VI.C
RSp1178	RS05068			-0,78 0,02				TRANSCRIPTION REGULATOR COMPOSITE TWO-COMPONENT TRANSCRIPTIO	VI.A
RSp1179	RS05069			-0,58 0,01		1,51 0,02	TIS102	TIS1021-TRANSPOSASE	V.A
RSp1180	RS05070		0,91 0,03	-1,47 0,00	1,85 0,00			HEMAGGLUTININ/HEMOLYSIN-RELATED PROTEIN	VI.C
RSp1181	RS05071		1,33 0,02	-1,90 0,00				TRANSMEMBRANE TYPE I SECRETION SYSTEM OUTER MEMBRANE EFFLUX PI	IV.E.2
RSp1182	RS05072		1,76 0,04	-1,24 0,00	-3,02 0,04			ABC TRANSPORTER TYPE I SECRETION SYSTEM, ATP-BINDING TRANSMEMBR	IV.E.2
RSp1183	RS05073		1,31 0,01	-1,47 0,00	-3,58 0,00			TRANSMEMBRANE TYPE I SECRETION SYSTEM PROTEIN	IV.E.2
RSp1184	RS05074							FRUCTOKINASE-LIKE PROTEIN (SUGAR KINASE)	I
RSp1185	RS03129			-0,53 0,00		-0,22 0,01	dsd	D-SERINE DEAMINASE (D-SERINE DEHYDRATASE)	I.A.10
RSp1186	RS03130			-0,86 0,00				TRANSCRIPTION REGULATOR	VI.A
RSp1187	RS03131		-0,83 0,03	-0,48 0,01			ndeD	N-ACYL-D-GLUTAMATE DEACYLASE	I
RSp1188	RS03132	-0,59 0,05		-0,72 0,00				COG0251, Translation initiation inhibitor	II.B.7
RSp1189	RS03133	-0,21 0,04		-0,26 0,01			gntP	TRANSMEMBRANE HIGH-AFFINITY GLUCONATE TRANSPORTER (GNT-III SYSTE	IV.D.3
RSp1190	RS03134		0,41 0,02	-0,51 0,00			eda	BIFUNCTIONAL ALKH PROTEIN (KHG/KDPG ALDOLASE): 4-HYDROXY-2-OXOGL	I.F.16
RSp1191	RS04751						ribB	3,4-DIHYDROXY-2-BUTANONE 4-PHOSPHATE SYNTHASE	I.B.7
RSp1192	RS03136							TRANSCRIPTION REGULATOR	VI.A
RSp1193	RS03137		0,97 0,03					TRANSMEMBRANE COG1807, 4-amino-4-deoxy-L-arabinose transferase and related	I
RSp1194	RS03138							TRANSMEMBRANE	VI.B
RSp1195	RS03139			-0,39 0,02	-0,80 0,03			TRANSCRIPTION REGULATOR TWO COMPONENT TRANSMEMBRANE SENSOR	VI.A
RSp1196	RS03140			-0,50 0,03				TRANSCRIPTION REGULATOR TWO COMPONENT RESPONSE REGULATOR	VI.A
RSp1197	RS03141			-0,82 0,00				MEMBRANE FUSION PROTEIN	III.B.2
RSp1198	RS03142		0,73 0,03					TRANSMEMBRANE DRUG EFFLUX PUMP	IV.D
RSp1199	RS03143							TRANSMEMBRANE DRUG EFFLUX PROTEIN	IV.D
RSp1200	RS03144			-0,92 0,02	0,92 0,04			TRANSMEMBRANE OUTER MEMBRANE CHANEL LIPOPROTEIN	IV.D
RSp1201	RS03145							SIGNAL PEPTIDE	VI.C
RSp1202	RS03146					0,91 0,02		TRANSMEMBRANE O-ANTIGEN POLYMERASE	II.B.2
RSp1203	RS03147							SIGNAL TRANSDUCTION PROTEIN GGDEF DOMAIN	VI.A
RSp1204	RS03148			-0,66 0,03				HYPOTHETICAL PROTEIN	VI.D
RSp1205	RS03149			-0,33 0,02				CONSERVED HYPOTHETICAL PROTEIN	VI.C
RSp1206	RS03150							OXIDOREDUCTASE TRANSMEMBRANE CYTOCHROME BD-II OXIDASE (SUBUNI	I.G.1
RSp1207	RS03151							OXIDOREDUCTASE TRANSMEMBRANE CYTOCHROME BD-II OXIDASE (SUBUNI	I.G.1
RSp1208	RS03152		0,37 0,05	-0,41 0,02				SIGNAL TRANSDUCTION PROTEIN EAL - GGDEF DOMAINS	VI.A
RSp1209	RS03153		-0,66 0,02					TRANSMEMBRANE METHYL-ACCEPTING CHEMOTAXIS TRANSDUCER PROTEII	IV.C
RSp1210	RS03154			-0,58 0,03	-1,81 0,00			TRANSMEMBRANE LIPOPROTEIN	VI.C
RSp1211	RS03155		1,03 0,02				cls	TRANSMEMBRANE CARDIOLIPIN SYNTHETASE	II.B.1
RSp1212	RS03156		-6,40 0,00	4,86 0,00	3,92 0,00			HYPOTHETICAL PROTEIN	VI.D
RSp1213	RS03157		0,81 0,01					TRANSMEMBRANE COG0534, Na+-driven multidrug efflux pump	IV.G
RSp1214	RS03158			-0,59 0,03	-1,07 0,00			COG3644, Uncharacterized protein conserved in bacteria	VI.C
RSp1215	RS03159			0,93 0,00				PSEUDOGENE (TRUNCATED ANKYRIN REPEAT HARBORING PROTEIN)	VI.B
RSp1217	RS03161			-0,64 0,00	-0,96 0,01		tISRso14	ISRso14-TRANSPOSASE ORFB	V.A
RSp1218	RS03162		-1,67 0,02	1,91 0,00	1,19 0,05			PSEUDOGENE (N-TERMINAL FRAGMENT)	VI.D
RSp1219	RS03163						nasA	OXIDOREDUCTASE NITRATE REDUCTASE LARGE SUBUNIT	I.H.2
RSp1220	RS03164							OXIDOREDUCTASE FLAVOPROTEIN FAD	I.H.2
RSp1221	RS03165						nirD	OXIDOREDUCTASE NITRITE REDUCTASE NADPH (SMALL SUBUNIT)	I.H.2
RSp1222	RS03166						nirB	OXIDOREDUCTASE NITRITE REDUCTASE NADPH (LARGE SUBUNIT)	I.H.2
RSp1223	RS03167							TRANSMEMBRANE HIGH AFFINITY NITRATE TRANSPORTER	I.H.2
RSp1224	RS03168			-0,41 0,02	-0,76 0,05		aer	TRANSMEMBRANE AEROTAXIS SENSOR RECEPTOR (CHEMOTAXIS TRANSDU(	IV.C
RSp1225	RS03169						tzs	ISOPENTENYL TRANSFERASE	I
RSp1226	RS03170							HYPOTHETICAL PROTEIN	VI.D
RSp1227	RS03171		-0,80 0,04					OXIDOREDUCTASE COG2141, Coenzyme F420-dependent N5,N10-methylene tetral	I
RSp1228	RS03172				3,51 0,02			SIGNAL PEPTIDE	VI.C
RSp1229	RS03173			-0,37 0,05				COG0596, Predicted hydrolases or acyltransferases (alpha/beta hydrolase superfamil	I

RSp1230	RS03174												OXIDOREDUCTASE DIOXYGENASE	I
RSp1231	RS03175			0,55	0,05								COG0684, Demethylmenaquinone methyltransferase	I
RSp1232	RS03176					0,27	0,00					aatA1	ASPARTATE AMINOTRANSFERASE A	I.A.19
RSp1233	RS03177												COG0684, Demethylmenaquinone methyltransferase	I
RSp1234	RS03178			0,54	0,05	-0,42	0,01	-0,84	0,04				COG0684, Demethylmenaquinone methyltransferase	I
RSp1235	RS03179					-0,42	0,04						TRANSCRIPTION REGULATOR	VI.A
RSp1236	RS03180			-2,76	0,00	4,33	0,00	3,03	0,00				HYPOTHETICAL PROTEIN	VI.B
RSp1237	RS03181												TRANSCRIPTION REGULATOR	VI.A
RSp1238	RS03182					0,71	0,00						SIGNAL PEPTIDE	VI.C
RSp1239	RS03183			-3,70	0,00	6,12	0,00	3,50	0,00				HYPOTHETICAL PROTEIN	VI.B
RSp1240	RS03184	-0,43	0,02	-2,01	0,00	3,99	0,00	1,85	0,00	-1,55	0,00		COG2951, Membrane-bound lytic murein transglycosylase B	III.B.2
RSp1241	RS03185					0,41	0,00						COG1510, Predicted transcriptional regulators	VI.A
RSp1242	RS03186					1,81	0,00						OXIDOREDUCTASE TRANSMEMBRANE CYTOCHROME BD-II OXIDASE (SUBUNI	I.G.1
RSp1243	RS03187			0,99	0,00	1,93	0,00						OXIDOREDUCTASE TRANSMEMBRANE CYTOCHROME BD-II OXIDASE (SUBUNI	I.G.1
RSp1244	RS03188												OXIDOREDUCTASE COG1893, Ketopantoate reductase	I
RSp1245	RS03189			1,21	0,03								ARYLESTERASE	I.I.4
RSp1246	RS03190												COG0251, Translation initiation inhibitor	II.B.7
RSp1247	RS03191					-0,57	0,05						TRANSCRIPTION REGULATOR	VI.A
RSp1248	RS03192												OXIDOREDUCTASE COG4188, Predicted dienelactone hydrolase	I.I.4
RSp1249	RS03193					-1,18	0,00						GLYOXALASE	I
RSp1250	RS03194												CONSERVED HYPOTHETICAL PROTEIN	VI.C
RSp1251	RS03196							0,85	0,03				SIGNAL PEPTIDE	VI.C
RSp1252	RS03197					-1,34	0,00	-1,95	0,00			hipB	TRANSCRIPTION REGULATOR	VI.A
RSp1253	RS05298					-0,96	0,00					pseudoge	HIPA PROTEIN RELATED(FRAGMENT)	VI.C
RSp1254	RS05299					-0,48	0,02	-2,55	0,03			pseudoge	HIPA PROTEIN RELATED(FRAGMENT)	VI.C
RSp1255	RS05300					-0,54	0,01						CONSERVED HYPOTHETICAL PROTEIN	VI.C
RSp1256	RS05301			0,32	0,04	-0,39	0,04						TRANSMEMBRANE INTEGRAL MEMBRANE PROTEIN	VI.C
RSp1257	RS05302					-0,71	0,00			-1,22	0,01		TRANSCRIPTION REGULATOR	VI.A
RSp1258	RS05303											acpD	[ACYL-CARRIER-PROTEIN] PHOSPHODIESTERASE	I.B.1
RSp1259	RS05304					-0,89	0,00	-1,13	0,05				TRANSMEMBRANE	VI.D
RSp1260	RS05305					-1,05	0,00	1,44	0,02	5,10	0,00		TRANSMEMBRANE LIPOPROTEIN	VI.D
RSp1261	RS05306							0,93	0,02				SIGNAL PEPTIDE	VI.B
RSp1262	RS05307												CONSERVED HYPOTHETICAL PROTEIN	VI.C
RSp1263	RS05308			1,12	0,00	-0,81	0,01					nadB2	OXIDOREDUCTASE L-ASPARTATE OXIDASE (QUINOLINATE SYNTHETASE B)	I.B.9
RSp1264	RS05309												TRANSMEMBRANE COG0730, Permease	IV.D
RSp1265	RS05310												ACETYL-COENZYME A SYNTHETASE (ACETATE--CoA LIGASE)	I.C
RSp1266	RS05311							0,83	0,03				TRANSMEMBRANE	VI.D
RSp1267	RS05312					-0,28	0,05						TRANSCRIPTION REGULATOR	VI.A
RSp1268	RS05313												TRANSMEMBRANE INTEGRAL MEMBRANE PROTEIN	VI.C
RSp1269	RS05314			-1,95	0,00	1,88	0,00	1,66	0,00				SIGNAL PEPTIDE	VI.B
RSp1270	RS05315	-3,97	0,01	-3,05	0,00	4,87	0,00	3,18	0,00	-0,91	0,00		SIGNAL PEPTIDE	VI.C
RSp1271	RS05316												FERRISIDEROPHORE RECEPTOR	IV.D.5
RSp1272	RS05317					-0,47	0,01						TRANSMEMBRANE	VI.D
RSp1273	RS05318					-0,32	0,01						CONSERVED HYPOTHETICAL PROTEIN	VI.C
RSp1274	RS05319					-0,93	0,00						TRANSMEMBRANE	VI.C
RSp1275	RS05320					-0,70	0,00						TRANSMEMBRANE	VI.C
RSp1276	RS05321												ATP-DEPENDENT DNA HELICASE II	II.B.8
RSp1277	RS05322	-1,90	0,00	-2,57	0,00	4,30	0,00	2,28	0,00				HYPOTHETICAL PROTEIN	VI.B
RSp1278	RS05323												TRANSMEMBRANE TRANSPORTER PROTEIN	IV.D
RSp1279	RS05324					-0,50	0,04					fumC	FUMARATE HYDRATASE CLASS II (FUMARASE)	I.H.6
RSp1280	RS05325					3,82	0,00	3,59	0,00				COG0524, SUGAR KINASE	IV.D.3
RSp1281	RS05326			-1,46	0,02	4,27	0,00	3,54	0,00				CONSERVED HYPOTHETICAL PROTEIN	VI.C
RSp1282	RS05327												PTS system, fructose-specific EIIA/HPr/EI components	IV.D.3
RSp1283	RS05328												TRANSMEMBRANE COG4580, MALTOPORIN (phage lambda and maltose receptor)	IV.D.3
RSp1284	RS05329											scrB	SUCROSE-6-PHOSPHATE HYDROLASE (SUCRASE INVERTASE)	I.F.4
RSp1285	RS05330											scrA	TRANSMEMBRANE PTS SYSTEM, SUCROSE-SPECIFIC (IIBC COMPONENT)	I.F.4
RSp1286	RS05331							-1,29	0,00			scrR	TRANSCRIPTION REGULATOR DNA-BINDING SUCROSE OPERON TRANSCRIPT	I.F.4
RSp1287	RS05332											pilE1	SIGNAL PEPTIDE TYPE 4 FIMBRIAL BIOGENESIS PROTEIN	III.A.2
RSp1288	RS05333												TYPE-4 FIMBRIAL BIOGENESIS PILY1-RELATED PROTEIN	III.A.2
RSp1289	RS05334											pilX	TRANSMEMBRANE TYPE-4 FIMBRIAL BIOGENESIS PROTEIN	III.A.2
RSp1290	RS05335											pilW	TRANSMEMBRANE TYPE 4 FIMBRIAL BIOGENESIS PROTEIN	III.A.2
RSp1291	RS05336											pilV	TRANSMEMBRANE TYPE-4 FIMBRIAL BIOGENESIS PILV PROTEIN	III.A.2
RSp1292	RS05337												SIGNAL PEPTIDE TYPE-4 FIMBRIAL PILIN RELATED PROTEIN	III.A.2
RSp1293	RS05338			0,84	0,04	-0,41	0,05					speD	TRANSMEMBRANE COG1586, S-adenosylmethionine decarboxylase	I.F.15
RSp1294	RS05339					0,60	0,00						TRANSMEMBRANE SERIN-RICH PROTEIN	VI.D
RSp1295	RS05340												TRANSMEMBRANE	VI.C
RSp1296	RS05341			0,79	0,00	-0,52	0,01			-0,51	0,00		TRANSMEMBRANE	VI.C
RSp1297	RS05342			0,89	0,02								TRANSMEMBRANE	VI.C
RSp1298	RS05343					-0,71	0,00						ABC TRANSPORTER COG0683, ABC-type branched-chain amino acid transport syst	IV.D
RSp1299	RS05344							-0,67	0,02			tISRso3	ISRso12-TRANSPOSASE ORFA	V.A
RSp1303	RS05696					-0,44	0,05					tISRso1	TRANSPOSASE	V.A
RSp1304	RS05976					-0,55	0,00	-1,04	0,04				HYPOTHETICAL PROTEIN	VI.D
RSp1305	RS05695			1,06	0,02	-0,76	0,04	-0,86	0,03				RHS-RELATED PROTEIN	VI.C
RSp1307	RS05692												TRANSMEMBRANE	VI.C
RSp1308	RS05691					-0,64	0,00	0,51	0,01	-1,37	0,00		COG0412, Dienelactone hydrolase and related enzymes	I
RSp1309	RS05690					-0,27	0,02						TRANSMEMBRANE	VI.C
RSp1310	RS05689									-1,73	0,01	gstO	GLUTATHIONE-S-TRANSFERASE	II.B.7
RSp1311	RS05685												TRANSMEMBRANE	VI.D
RSp1312	RS05665					-0,97	0,04						CONSERVED HYPOTHETICAL PROTEIN	VI.C
RSp1313	RS05664					-0,59	0,00	-0,64	0,01	-1,59	0,00		CONSERVED HYPOTHETICAL PROTEIN	VI.C
RSp1314	RS05663							-0,48	0,00				COG0491, Zn-dependent hydrolases, including glyoxylases	I.I
RSp1315	RS05662												COG0251, Translation initiation inhibitor	II.B.7
RSp1316	RS05661												OXIDOREDUCTASE 4-HYDROXYPHENYLPYRUVATE DIOXYGENASE	I.I.3
RSp1317	RS05660					-0,36	0,04	-2,00	0,01				TRANSCRIPTION REGULATOR	VI.A
RSp1318	RS05659												TRANSCRIPTION REGULATOR	VI.A
RSp1319	RS05658									-1,27	0,00		COG3832, Uncharacterized conserved protein	VI.C
RSp1320	RS05657					-0,46	0,01						TRANSCRIPTION REGULATOR DNA-BINDING TRANSCRIPTIONAL REGULATORY	VI.A
RSp1321	RS05656			-0,46	0,01	-0,32	0,02						TRANSMEMBRANE COG1280, Threonine efflux protein	IV.D.2
RSp1322	RS05655					-0,78	0,00	-0,97	0,00	-2,14	0,00		COG0251, Translation initiation inhibitor	II.B.7
RSp1323	RS05679			-1,75	0,00	1,13	0,00			-2,92	0,01		HYPOTHETICAL PROTEIN	VI.D
RSp1324	RS05678			-0,57	0,03	0,52	0,00						TRANSMEMBRANE LIPOPROTEIN	VI.D
RSp1325	RS05677												TRANSMEMBRANE	VI.D
RSp1326	RS05669					-0,35	0,00			-1,56	0,01		TRANSCRIPTION REGULATOR	VI.A
RSp1328	RS05653												TRANSMEMBRANE EFFLUX PUMP ANTIBIOTIC RESISTANCE PROTEIN	IV.D
RSp1329	RS05651					-0,43	0,01	-1,64	0,02	-2,34	0,01		TRANSMEMBRANE	VI.D
RSp1330	RS05650					-0,35	0,05	-1,46	0,01				HYPOTHETICAL PROTEIN	VI.B
RSp1333	RS05623					-0,43	0,04						TRANSPOSASE	V.A
RSp1334	RS06036					-0,66	0,00						CONSERVED HYPOTHETICAL PROTEIN	VI.D
RSp1335	RS04760			1,20	0,02	-0,79	0,01	-0,87	0,01				RHS-RELATED PROTEIN	VI.B
RSp1336	RS04761	1,42	0,02			-0,54	0,02						Transposase	V.A
RSp1337	RS04762											speE1	TRANSMEMBRANE SPERMIDINE SYNTHASE	I.F.15
RSp1338	RS04763												TRANSMEMBRANE	VI.C
RSp1339	RS04764					-0,79	0,00						COG0412, Dienelactone hydrolase and related enzymes	I
RSp1340	RS04765												TRANSMEMBRANE	VI.C
RSp1341	RS04766											gstP	GLUTATHIONE S-TRANSFERASE	II.B.7
RSp1342	RS04770					-0,83	0,00						TRANSMEMBRANE	VI.D
RSp1343	RS04767					-0,32	0,01						CONSERVED HYPOTHETICAL PROTEIN	VI.C
RSp1344	RS04773					-0,51	0,01	-0,60	0,02				CONSERVED HYPOTHETICAL PROTEIN	VI.C
RSp1345	RS04774												COG0491, Zn-dependent hydrolases, including glyoxylases	I.I
RSp1346	RS04775					-0,55	0,05						TRANSMEMBRANE COG0251, Translation initiation inhibitor	II.B.7
RSp1347	RS04776							-0,92	0,05				OXIDOREDUCTASE 4-HYDROXYPHENYLPYRUVATE DIOXYGENASE	I.I.3
RSp1348	RS04777					-0,32	0,00						TRANSCRIPTION REGULATOR	VI.A

RSp1349	RS04778												TRANSCRIPTION REGULATOR	VI.A
RSp1350	RS04779					-0,39	0,02						COG3832, Uncharacterized conserved protein	VI.C
RSp1351	RS04780					-0,44	0,00						TRANSCRIPTION REGULATOR	VI.A
RSp1352	RS04781												TRANSMEMBRANE COG1280, Threonine efflux protein	IV.D.2
RSp1353	RS04783					-0,79	0,00	-1,16	0,02	-2,72	0,00		COG0251, Translation initiation inhibitor	II.B.7
RSp1354	RS04818					-0,40	0,01	-0,67	0,01	-2,09	0,01		HYPOTHETICAL PROTEIN	VI.D
RSp1355	RS04819												TRANSMEMBRANE LIPOPROTEIN	VI.D
RSp1356	RS04820												TRANSMEMBRANE	VI.D
RSp1357	RS04822					-0,31	0,01						TRANSCRIPTION REGULATOR	VI.A
RSp1358	RS04784	-0,75	0,03									ilvE2	BRANCHED-CHAIN AMINO ACID AMINOTRANSFERASE	I.A.2
RSp1359	RS04823												TRANSMEMBRANE EFFLUX PUMP ANTIBIOTIC RESISTANCE PROTEIN	IV.D
RSp1360	RS04825					-0,45	0,01	-1,07	0,00	-1,37	0,00		TRANSMEMBRANE	VI.D
RSp1361	RS04826												HYPOTHETICAL PROTEIN	VI.B
RSp1363	RS04782			-1,20	0,04								TRANSMEMBRANE METHYL-ACCEPTING CHEMOTAXIS TRANSDUCER	IV.C
RSp1364	RS02094	-4,71	0,02									sucA	OXIDOREDUCTASE 2-OXOGLUTARATE DEHYDROGENASE E1 DECARBOXYLAS	I.H.6
RSp1365	RS02093			0,68	0,00	-0,32	0,00						TRANSMEMBRANE COG0204, 1-acyl-sn-glycerol-3-phosphate acyltransferase	I
RSp1366	RS02092					0,94	0,00	1,09	0,03			abpE	TRANSMEMBRANE THIAMINE BIOSYNTHESIS PROTEIN APBE	I.B.8
RSp1367	RS02091					0,62	0,05						COG3245, Cytochrome c5	I.G.1
RSp1368	RS02090											nosZ	OXIDOREDUCTASE NITROUS-OXIDE REDUCTASE	I.F.5
RSp1369	RS02089											nosR	TRANSCRIPTION REGULATOR TRANSMEMBRANE REGULATORY PROTEIN NOS	I.F.5
RSp1370	RS02088											nosD	SIGNAL PEPTIDE PERIPLASMIC COPPER-BINDING PROTEIN NOSD	I.F.5
RSp1371	RS02087											nosF	ABC TRANSPORTER ABC-TYPE COPPER TRANSPORT SYSTEM, ATPase compo	I.F.5
RSp1372	RS02086											nosY	TRANSMEMBRANE PROTEIN NOSY	I.F.5
RSp1373	RS02085							-2,64	0,03			nosL	NOSL LIPOPROTEIN	I.F.5
RSp1374	RS02084												SKWP PROTEIN 2	VI.C
RSp1375	RS02083			1,05	0,00								TRANSMEMBRANE	VI.C
RSp1376	RS02082					-0,80	0,00						TRANSMEMBRANE COG1296, Predicted branched-chain amino acid permease (aza	IV.D
RSp1377	RS02081					-2,23	0,00	-1,94	0,03	-1,14	0,00		TRANSCRIPTION REGULATOR	VI.A
RSp1378	RS02080					-2,33	0,00	-3,58	0,00				COG2963: Transposase and inactivated derivatives	V.A
RSp1379	RS02079							0,73	0,03				SIGNAL PEPTIDE	VI.D
RSp1380	RS02078			0,42	0,05								OXIDOREDUCTASE COG1249, Pyruvate/2-oxoglutarate dehydrogenase complex, di	I
RSp1381	RS02077			0,54	0,04							phnA	PHNA PROTEIN ALKYLPHOSPHONATE UPTAKE	VI.C
RSp1382	RS02076			-0,60	0,05	-0,54	0,01	-1,68	0,02				SIGNAL PEPTIDE	VI.C
RSp1383	RS02075											ssuA2	ALKANESULFONATES BINDING PROTEIN PRECURSOR	I.F.20
RSp1384	RS02074					1,09	0,02	2,45	0,00				TRANSMEMBRANE	VI.D
RSp1385	RS02073									-1,65	0,00		TRANSMEMBRANE	VI.C
RSp1386	RS02072			-2,39	0,00	2,47	0,00	0,95	0,01				LIPOPROTEIN	VI.C
RSp1387	RS02071												TRANSCRIPTION REGULATOR	VI.A
RSp1388	RS02070			-1,65	0,04	1,90	0,00	4,77	0,01				HYPOTHETICAL PROTEIN	VI.D
RSp1389	RS02069					0,45	0,02						COG2220, PREDICTED ZINC-DEPENDENT HYDROLASE	I.I
RSp1390	RS02068											fliA	TRANSCRIPTION REGULATOR RNA POLYMERASE SIGMA FACTOR FOR FLAGE	IV.C
RSp1391	RS02067					2,61	0,00					fleN	FLAGELLAR SYNTHESIS REGULATOR FleN	IV.C
RSp1392	RS02066											flhF	FLAGELLAR BIOSYNTHETIC PROTEIN FLHF	IV.C
RSp1393	RS02065											flhA	TRANSMEMBRANE FLAGELLAR BIOSYNTHESIS PROTEIN FLHA	IV.C
RSp1394	RS02064											flhB	TRANSMEMBRANE FLAGELLAR BIOSYNTHETIC PROTEIN FLHB	IV.C
RSp1395	RS02063							0,71	0,02				TRANSCRIPTION REGULATOR TRANSCRIPTION REGULATOR	VI.A
RSp1396	RS02062												TRANSMEMBRANE TRANSPORTER	IV.D
RSp1397	RS02061											aroQ2	3-DEHYDROQUINATE DEHYDRATASE (3-DEHYDROQUINASE) (TYPE II DHQASE	I.A.5
RSp1398	RS02060											aroE2	OXIDOREDUCTASE SHIKIMATE 5-DEHYDROGENASE	I.A.5
RSp1399	RS02059							0,62	0,02				SIGNAL PEPTIDE PORIN	IV.D
RSp1400	RS02058					-0,86	0,01						COG3185, 4-hydroxyphenylpyruvate dioxygenase AND COG1082, Sugar phosphate	I
RSp1401	RS02057					-1,24	0,01					cheZ1	CHEMOTAXIS PROTEIN CHEZ	IV.C
RSp1402	RS02056					-0,74	0,02	-0,79	0,02			che	TRANSCRIPTION REGULATOR CHEMOTAXIS RESPONSE REGULATOR PROTEI	IV.C
RSp1403	RS02055											cheB	PROTEIN-GLUTAMATE METHYLESTERASE	IV.C
RSp1404	RS02054												CHEMOTAXIS PROTEIN	IV.C
RSp1405	RS02053					-0,60	0,02					cheR	CHEMOTAXIS PROTEIN METHYLTRANSFERASE	IV.C
RSp1406	RS02052							-1,08	0,02			cheD3	TRANSMEMBRANE METHYL-ACCEPTING CHEMOTAXIS PROTEIN I (SERINE CH	IV.C
RSp1407	RS02051											cheW	CHEMOTAXIS PROTEIN CHEW	IV.C
RSp1408	RS05777											cheA	TRANSCRIPTION REGULATOR CHEMOTAXIS SENSOR HISTIDINE KINASE PROT	IV.C
RSp1409	RS05776					-0,64	0,00					che	TRANSCRIPTION REGULATOR CHEMOTAXIS RESPONSE REGULATOR PROTEI	IV.C
RSp1410	RS05775											motB	TRANSMEMBRANE CHEMOTAXIS PROTEIN (MOTILITY PROTEIN B)	IV.C
RSp1411	RS05774					-0,91	0,00	-0,95	0,02			motA	TRANSMEMBRANE CHEMOTAXIS PROTEIN (MOTILITY PROTEIN A)	IV.C
RSp1412	RS05773					-0,94	0,00					flhC	TRANSCRIPTION REGULATOR FLAGELLAR TRANSCRIPTIONAL ACTIVATOR	IV.C
RSp1413	RS03127					-1,29	0,01	-2,23	0,04			flhD	TRANSCRIPTION REGULATOR FLAGELLAR TRANSCRIPTIONAL ACTIVATOR	IV.C
RSp1414	RS05841					-0,38	0,05						HYPOTHETICAL PROTEIN	VI.D
RSp1415	RS03126												HYPOTHETICAL PROTEIN	VI.D
RSp1416	RS03125												TRANSMEMBRANE	VI.B
RSp1417	RS03124	-1,10	0,00	1,10	0,02	-0,38	0,04	0,95	0,02	2,21	0,00		TRANSMEMBRANE TRANSMEMBRANE EFFLUX PROTEIN	IV.D
RSp1418	RS03123	-3,49	0,00	1,05	0,00			3,13	0,00	3,02	0,00		HYDROLASE	I.I
RSp1419	RS03122	-3,09	0,00	1,23	0,00	-0,22	0,01	2,48	0,00	2,80	0,00		PEPTIDE SYNTHASE	II.B.7
RSp1420	RS06015												HYPOTHETICAL PROTEIN	VI.D
RSp1421	RS03121					-0,67	0,00			1,86	0,00		COG1804, Predicted acyl-CoA transferases/carnitine dehydratase	I
RSp1422	RS03120												PEPTIDE SYNTHETASE	II.B.7
RSp1423	RS03119												OXIDOREDUCTASE COG0644, Dehydrogenases (flavoproteins)	I
RSp1424	RS03118			0,79	0,01							ectB	DIAMINOBUTYRATE--PYRUVATE AMINOTRANSFERASE	IV.F.2
RSp1425	RS03117			0,58	0,02					1,77	0,00	pvdA	OXIDOREDUCTASE L-ORNITHINE 5-MONOOXYGENASE	IV.D.5
RSp1426	RS03116			0,66	0,00					1,44	0,00		HYPOTHETICAL PROTEIN	VI.D
RSp1427	RS03115					0,43	0,01						SIGNAL PEPTIDE	VI.D
RSp1428	RS03114			0,89	0,00					2,99	0,02		COG0607, Rhodanese-related sulfurtransferase	I
RSp1429	RS03113			0,48	0,04								COG0657, Esterase/lipase	I
RSp1430	RS03112			0,65	0,01								CONSERVED HYPOTHETICAL PROTEIN	VI.C
RSp1431	RS03111												OXIDOREDUCTASE COG0665, Glycine/D-amino acid oxidases (deaminating)	I
RSp1432	RS03110			1,04	0,00								COG2140, Thermophilic glucose-6-phosphate isomerase and related metalloenzymes	I
RSp1433	RS03109			0,67	0,02	-0,38	0,04						HYPOTHETICAL PROTEIN	VI.D
RSp1434	RS03108					-0,24	0,03			1,59	0,00		COG0318, Acyl-CoA synthetases (AMP-forming) / AMP-acid ligases II	I
RSp1435	RS03107			0,58	0,04								TRANSMEMBRANE COG0596, Predicted hydrolases or acyltransferases (alpha/beta	I
RSp1436	RS03106												Acetyltransferase	I
RSp1437	RS03105			0,60	0,05					1,78	0,00		HYPOTHETICAL PROTEIN	VI.B
RSp1438	RS03104	-0,82	0,01							0,96	0,01		TRNA SYNTHETASE	II.D.1
RSp1439	RS03103											cysE1	SERINE ACETYLTRANSFERASE	I.A.9
RSp1440	RS06034					2,94	0,00						HYPOTHETICAL PROTEIN	VI.D
RSp1441	RS03102							-1,01	0,03				TRANSCRIPTION REGULATOR TWO-COMPONENT SENSOR KINASE	VI.A
RSp1442	RS03101							0,47	0,01				TRANSCRIPTION REGULATOR TWO-COMPONENT RESPONSE REGULATOR	VI.A
RSp1443	RS03100												SIGNAL PEPTIDE	VI.B
RSp1444	RS03099					-0,48	0,05						HEMAGGLUTININ-RELATED PROTEIN	VI.C
RSp1445	RS03098							-0,95	0,01				SIGNAL PEPTIDE ACTIVATION/SECRETION PROTEIN	IV.E.2
RSp1446	RS03097											cfa1	CYCLOPROPANE-FATTY-ACYL-PHOSPHOLIPID SYNTHASE	I.C
RSp1447	RS03096							1,10	0,01	4,92	0,00		ABC TRANSPORTER ABC-type branched-chain amino acid transport systems, peripl	IV.D.2
RSp1448	RS03095											pykA2	PYRUVATE KINASE II	I.H.8
RSp1449	RS03094											ttuD1	OXIDOREDUCTASE HYDROXYPYRUVATE REDUCTASE	I.I.4
RSp1450	RS03093							-0,80	0,01				TRANSCRIPTION REGULATOR	VI.A
RSp1451	RS03092							-1,06	0,05				HYPOTHETICAL PROTEIN	VI.D
RSp1452	RS03091							-0,66	0,01				HYPOTHETICAL PROTEIN	VI.D
RSp1453	RS03090					-0,50	0,01						SIGNAL PEPTIDE COG3258, CYTOCHROME C	I.H
RSp1454	RS03089					-0,71	0,04						TRANSCRIPTION REGULATOR TWO-COMPONENT TRANSMEMBRANE SENSOR	VI.A
RSp1455	RS03088												TRANSCRIPTION REGULATOR TWO-COMPONENT RESPONSE REGULATOR	VI.A
RSp1456	RS05806												OUTER MEMBRANE LIPOPROTEIN	III.B.2
RSp1457	RS03087												SIGNAL PEPTIDE CATION-EFFLUX SYSTEM PROTEIN	IV.D.4
RSp1458	RS03086												TRANSMEMBRANE TRANSPORTER PROTEIN	IV.D
RSp1459	RS03085					0,46	0,00						SIGNAL PEPTIDE COG1574, Predicted metal-dependent hydrolase with the TIM-barr	I.I
RSp1460	RS03084			-2,71	0,01	4,14	0,00	4,05	0,00				HYPOTHETICAL PROTEIN	VI.B
RSp1461	RS03083	-3,58	0,00	-3,74	0,00	5,07	0,00	3,21	0,00	3,63	0,00		SIGNAL PEPTIDE	VI.C

RSp1462	RS03082		-1,66 0,00	2,30 0,00	2,78 0,00	3,96 0,00		Outer membrane efflux protein	IV.D
RSp1463	RS03081		-2,06 0,00	2,88 0,00	3,82 0,00	3,88 0,00		ABC TRANSPORTER HlyD family secretion protein	IV.D
RSp1464	RS03080		-0,89 0,00	1,52 0,01	3,76 0,01	3,23 0,00		ABC TRANSPORTER ABC-type transport system, ATPase component	IV.D
RSp1465	RS03079				1,41 0,02			ABC TRANSPORTER ABC-type transport system , permease component	IV.D
RSp1466	RS03078		-4,91 0,00	5,32 0,00	4,29 0,00	4,56 0,00		SIGNAL PEPTIDE	VI.C
RSp1467	RS03077		-4,32 0,00	5,05 0,00	4,81 0,00	2,99 0,00		SIGNAL PEPTIDE	VI.C
RSp1468	RS03076							SIGNAL PEPTIDE COG4313, Protein involved in meta-pathway of phenol degradation	IV.F.1
RSp1469	RS03075							TYROSINE PHOSPHATASE	VI.B
RSp1470	RS03074			-0,41 0,05				TRANSCRIPTION REGULATOR	VI.A
RSp1471	RS03073			-0,54 0,01				TRANSCRIPTION REGULATOR	VI.A
RSp1472	RS03072		1,26 0,01	-0,46 0,01				4-CARBOXYMUCONOLACTONE DECARBOXYLASE	I
RSp1473	RS03071			-0,37 0,03				OXIDOREDUCTASE COG0667, Oxidoreductase (related to aryl-alcohol dehydrogena:	I
RSp1474	RS06014		-4,48 0,00	3,88 0,00	3,42 0,00			HYPOTHETICAL PROTEIN	VI.B
RSp1475	RS03068		-3,83 0,00	5,09 0,00	3,03 0,00			CONSERVED HYPOTHETICAL PROTEIN	VI.B
RSp1476	RS03067							CONSERVED HYPOTHETICAL PROTEIN	VI.C
RSp1477	RS03066							SIGNAL PEPTIDE	VI.D
RSp1478	RS03065		0,95 0,01					TRANSCRIPTION REGULATOR	VI.A
RSp1479	RS03064			-0,29 0,00	-0,57 0,05			CONSERVED HYPOTHETICAL PROTEIN	VI.C
RSp1480	RS03063		1,77 0,00	-0,30 0,04				CONSERVED HYPOTHETICAL PROTEIN	VI.C
RSp1481	RS03062		0,96 0,01	0,52 0,03				TRANSMEMBRANE	VI.C
RSp1482	RS03061							TRANSMEMBRANE COG2885, Outer membrane protein	VI.C
RSp1483	RS03060		1,69 0,02					CONSERVED HYPOTHETICAL PROTEIN	VI.C
RSp1484	RS03059		1,26 0,00					OXIDOREDUCTASE COG0596, Hydrolase or acyltransferase (alpha/beta hydrolase s	I
RSp1485	RS03058		1,13 0,00					TRANSMEMBRANE	VI.C
RSp1486	RS03057		1,13 0,00					COG0439, Biotin carboxylase	I
RSp1487	RS03056		1,44 0,00				cmcH	3'-HYDROXYMETHYLCEPHEM-O-CARBAMOYLTRANSFERASE	II.B
RSp1488	RS03055		0,73 0,01			1,26 0,02		HYPOTHETICAL PROTEIN	VI.D
RSp1489	RS03054		1,24 0,00		-1,98 0,02			CONSERVED HYPOTHETICAL PROTEIN	VI.C
RSp1490	RS03053		0,69 0,01			1,10 0,00		TRANSMEMBRANE	VI.D
RSp1491	RS03052		1,02 0,00					COG2192, Carbamoyl transferase	I
RSp1492	RS03051		0,92 0,00		0,33 0,00			HYPOTHETICAL PROTEIN	VI.B
RSp1493	RS05842		-1,23 0,02	0,62 0,01	-0,78 0,05			HYPOTHETICAL PROTEIN	VI.D
RSp1494	RS03050			-0,35 0,01				HYPOTHETICAL PROTEIN	VI.D
RSp1495	RS03046		0,39 0,01	-0,25 0,02				COG0431, Flavoprotein	VI.C
RSp1496	RS03045							TRANSMEMBRANE CATION-EFFLUX SYSTEM PROTEIN	IV.D.4
RSp1497	RS03044							SIGNAL PEPTIDE LIPOPROTEIN	VI.C
RSp1498	RS03043							SIGNAL PEPTIDE OUTER MEMBRANE USHER PROTEIN	III.B.2
RSp1499	RS03042							TRANSMEMBRANE PILI ASSEMBLY CHAPERONE	IV.B
RSp1500	RS03041				-1,25 0,05			SIGNAL PEPTIDE	VI.C
RSp1501	RS03040		-0,99 0,01					SIGNAL PEPTIDE	VI.C
RSp1502	RS03039							SIGNAL PEPTIDE	VI.C
RSp1503	RS03038							TRANSMEMBRANE MAJOR ANAEROBICALLY INDUCED OUTER MEMBRANE PR(	I.H.2
RSp1504	RS03037							CONSERVED HYPOTHETICAL PROTEIN	VI.C
RSp1505	RS03036						norB	TRANSMEMBRANE NITRIC OXIDE REDUCTASE (SUBUNIT B)	I.H.2
RSp1506	RS03035							TRANSCRIPTION REGULATOR	VI.A
RSp1507	RS03034							ABC TRANSPORTER ATP-BINDING PROTEIN	IV.D
RSp1508	RS03033		-0,48 0,05	0,93 0,00				ABC TRANSPORTER TRANSMEMBRANEE PROTEIN	IV.D
RSp1509	RS03032							TRANSMEMBRANE PERIPLASMIC SUBSTRATE-BINDING PROTEIN	IV.D
RSp1510	RS03031		0,48 0,01	-0,30 0,04				CONSERVED HYPOTHETICAL PROTEIN	VI.C
RSp1511	RS04788			-0,62 0,00	0,86 0,01	1,27 0,02		CONSERVED HYPOTHETICAL PROTEIN	VI.C
RSp1512	RS04789					1,19 0,05		TRANSCRIPTION REGULATOR	VI.A
RSp1513	RS04790							TRANSMEMBRANE COG4125, Predicted membrane protein	VI.C
RSp1514	RS04791				-0,68 0,03			TRANSCRIPTION REGULATOR	VI.A
RSp1515	RS04792				-1,31 0,01			COG0251, TdcF, Putative translation initiation inhibitor	II.B.7
RSp1516	RS04793							AMINO-ACID DEHYDRATASE	I.I.3
RSp1517	RS04794							COG2964: Uncharacterized protein conserved in bacteria	VI.C
RSp1518	RS04795			-1,06 0,00	1,08 0,00	1,44 0,00		SIGNAL PEPTIDE	VI.C
RSp1519	RS04796			-0,45 0,01				TRANSMEMBRANE SULFATE TRANSPORTER	IV.D
RSp1520	RS04797							CONSERVED HYPOTHETICAL PROTEIN	VI.C
RSp1521	RS04798		0,38 0,01	-0,35 0,03				COG3946, Type IV secretory pathway VirJ component - related protein	VI.C
RSp1522	RS04799			-0,44 0,00				TRANSMEMBRANE	VI.C
RSp1523	RS04800			-0,77 0,00	-1,48 0,05	-0,48 0,00		CONSERVED HYPOTHETICAL PROTEIN	VI.C
RSp1524	RS04801							TRANSMEMBRANE SIGNAL TRANSDUCTION PROTEIN GGDEF DOMAIN	VI.A
RSp1525	RS04802		0,66 0,05					TRANSMEMBRANE COG0705, Uncharacterized membrane protein	VI.C
RSp1526	RS04803		0,38 0,02	0,35 0,01	1,02 0,01	1,60 0,00		HYPOTHETICAL PROTEIN	VI.B
RSp1527	RS04804			0,39 0,00				SIGNAL PEPTIDE	VI.C
RSp1528	RS04805							HYPOTHETICAL PROTEIN	VI.B
RSp1529	RS04806	-1,23 0,01				4,00 0,00		OXIDOREDUCTASE 1-AMINOCYCLOPROPANE-1-CARBOXYLATE OXIDASE (ACC	I
RSp1530	RS04807			-0,72 0,00				OXIDOREDUCTASE L-ASCORBATE OXIDASE (ASCORBASE)	I.F
RSp1531	RS06038							SIGNAL PEPTIDE HYPOTHETICAL PROTEIN	VI.D
RSp1532	RS04809			-0,40 0,00	-0,23 0,01	-0,50 0,00		LIPOPROTEIN	VI.C
RSp1533	RS04810			-0,38 0,05				LIPOPROTEIN	VI.D
RSp1534	RS04811			-0,47 0,01		-0,88 0,02		SIGNAL PEPTIDE COG0760, Peptidyl-prolyl isomerase	II.B.7
RSp1535	RS04812			0,39 0,00		-1,47 0,00		SIGNAL PEPTIDE ACTIVATION/SECRETION PROTEIN	IV.E.2
RSp1536	RS04813	-1,21 0,02	-3,06 0,00	2,34 0,00	3,89 0,00			SIGNAL PEPTIDE HEMAGGLUTININ-RELATED PROTEIN	VI.C
RSp1537	RS04814							TRANSMEMBRANE	VI.C
RSp1538	RS06035							LIPOPROTEIN	VI.D
RSp1539	RS05770			-0,53 0,02				HEMAGGLUTININ-RELATED PROTEIN	VI.C
RSp1540	RS02096							CONSERVED HYPOTHETICAL PROTEIN	VI.C
RSp1541	RS02097				-1,15 0,02	1,23 0,02		HYPOTHETICAL PROTEIN	VI.D
RSp1542	RS02098			-0,39 0,02				HYPOTHETICAL PROTEIN	VI.D
RSp1543	RS02099							CONSERVED HYPOTHETICAL PROTEIN	VI.C
RSp1544	RS02100				-1,80 0,01			HYPOTHETICAL PROTEIN	VI.D
RSp1545	RS02101							HEMAGGLUTININ-RELATED PROTEIN	VI.B
RSp1546	RS02102			-0,64 0,00				CONSERVED HYPOTHETICAL PROTEIN	VI.C
RSp1548	RS02104				0,61 0,02			CONSERVED HYPOTHETICAL PROTEIN	VI.C
RSp1549	RS02105	-0,49 0,02		-0,47 0,04	-1,29 0,03			HYPOTHETICAL PROTEIN	VI.D
RSp1550	RS02106			-0,56 0,00	-1,11 0,00	-2,31 0,00		HYPOTHETICAL PROTEIN	VI.D
RSp1551	RS02107			-0,59 0,01	-1,32 0,00	-1,00 0,00		CONSERVED HYPOTHETICAL PROTEIN	VI.C
RSp1552	RS02108							SIGNAL PEPTIDE PROTEASE	II.A.4
RSp1553	RS02109							TRANSCRIPTION REGULATOR TRANSMEMBRANE TWO-COMPONENT SENSOR	VI.A
RSp1554	RS02110		0,73 0,03	-0,45 0,01				TRANSCRIPTION REGULATOR TWO-COMPONENT RESPONSE REGULATOR	VI.A
RSp1555	RS02111	-3,10 0,00	-8,74 0,00	7,61 0,00	5,11 0,00	-4,87 0,00	popF1	SECRETED PROTEIN PopF1	IV.E.2
RSp1556	RS02112			1,01 0,00			hexR	TRANSCRIPTION REGULATOR TRANSCRIPTION REGULATION REPRESSOR HE	I.F
RSp1557	RS02113		0,29 0,01	-0,29 0,03			glk	GLUCOKINASE	I.H.8
RSp1558	RS02114						pgl	OXIDOREDUCTASE 6-PHOSPHOGLUCONOLACTONASE	I.F.19
RSp1559	RS02115			-0,45 0,00			zwf	OXIDOREDUCTASE GLUCOSE-6-PHOSPHATE 1-DEHYDROGENASE	I.F.19
RSp1560	RS02116			0,24 0,01			edd	OXIDOREDUCTASE PHOSPHOGLUCONATE DEHYDRATASE	I.F.16
RSp1561	RS02117							COG0589, Universal stress protein UspA and related nucleotide-binding proteins	IV.G
RSp1562	RS02118			-0,41 0,00				CONSERVED HYPOTHETICAL PROTEIN	VI.C
RSp1563	RS02119		-0,50 0,04	-0,58 0,00	-0,69 0,01	0,88 0,03		TRANSMEMBRANE	VI.C
RSp1564	RS02120			-1,73 0,00	-1,14 0,00		coxM	TRANSMEMBRANE CYTOCHROME C OXIDASE POLYPEPTIDE II (CYTOCHROME	I.G.1
RSp1565	RS02121						coxN	OXIDOREDUCTASE CYTOCHROME C OXIDASE POLYPEPTIDE I (CYTOCHROME	I.G.1
RSp1566	RS02122			-0,38 0,01			coxO	OXIDOREDUCTASE CYTOCHROME-C OXIDASE	I.G.1
RSp1567	RS02123						coxP	OXIDOREDUCTASE CYTOCHROME-C OXIDASE SUBUNIT III	I.G.1
RSp1568	RS02124			-0,51 0,05				TRANSMEMBRANE COG5605, Predicted small integral membrane protein	III.B.1
RSp1569	RS02125		0,95 0,00	-0,86 0,01				TRANSMEMBRANE LIPOPROTEIN	VI.B
RSp1570	RS02126			-0,38 0,04				HYPOTHETICAL PROTEIN	VI.D
RSp1571	RS02127	-0,65 0,01	0,82 0,01	-1,31 0,00	-0,57 0,00	1,15 0,00		TRANSCRIPTION REGULATOR TRANSCRIPTIONAL REGULATOR	VI.A
RSp1572	RS02128		0,74 0,01	-1,38 0,00	-1,35 0,02			TRANSMEMBRANE TRANSPORT PROTEIN	IV.D
RSp1573	RS02129			-0,52 0,01	-0,57 0,02	-0,70 0,05		CONSERVED HYPOTHETICAL PROTEIN	VI.C
RSp1574	RS02130			-0,41 0,00		-0,58 0,01		TRANSCRIPTION REGULATOR TRANSCRIPTIONAL REGULATOR	VI.A

RSp1575	RS02131			0,75	0,01	-0,48	0,00						ABC TRANSPORTER AMINO-ACID-BINDING PERIPLASMIC PROTEIN (PBP)	IV.D.2
RSp1576	RS02132			0,90	0,01	-0,49	0,02	-0,41	0,02				ABC TRANSPORTER AMINO-ACID TRANSMEMBRANE PROTEIN	IV.D.2
RSp1577	RS02133												ABC TRANSPORTER AMINO-ACID TRANSMEMBRANE PROTEIN	IV.D.2
RSp1578	RS02134			0,86	0,01			0,66	0,05			speB	AGMATINASE	I.F.15
RSp1579	RS02135			0,90	0,02	-0,39	0,00			0,82	0,00		TRANSCRIPTION REGULATOR	VI.A
RSp1580	RS02136					-0,65	0,00			1,23	0,04		TRANSMEMBRANE	VI.C
RSp1581	RS02137	-1,97	0,00			2,27	0,00	2,03	0,00	2,67	0,00	katE	OXIDOREDUCTASE CATALASE hydroperoxidase HPII	IV.G.4
RSp1582	RS02138			-4,16	0,00	4,23	0,00						HYPOTHETICAL PROTEIN	VI.D
RSp1583	RS02139			1,11	0,02	-0,34	0,01						TRANSMEMBRANE O-acetylhomoserine sulfhydrylase	I
RSp1584	RS02143					-0,52	0,02						HYPOTHETICAL PROTEIN	VI.D
RSp1585	RS02144												TRANSMEMBRANE RHS-RELATED PROTEIN	VI.C
RSp1586	RS02145												HYPOTHETICAL PROTEIN	VI.D
RSp1587	RS02141					-0,32	0,02						REMNANT OF A TRANSPOSASE	V.A
RSp1588	RS02142			1,15	0,03	-0,45	0,01						REMNANT OF A TRANSPOSASE GENE	V.A
RSp1589	RS02146												HYPOTHETICAL PROTEIN	VI.D
RSp1590	RS02147												OXIDOREDUCTASE COG0665, Glycine/D-amino acid oxidases (deaminating)	I.I.3
RSp1591	RS02148					-0,47	0,01						OXIDOREDUCTASE ALDEHYDE DEHYDROGENASE	I
RSp1592	RS02149			0,70	0,05	-0,18	0,03						ABC TRANSPORTER ABC-type branched-chain amino acid transport systems, peripla	IV.D.2
RSp1593	RS02150					-0,33	0,04						TRANSMEMBRANE REGULATOR TRANSCRIPTION REGULATOR	VI.A
RSp1594	RS02151												SIGNAL PEPTIDE PORIN	IV.D
RSp1595	RS02152												TRANSMEMBRANE METABOLITE TRANSPORT PROTEIN	IV.D
RSp1596	RS02153					-0,44	0,02						HYPOTHETICAL PROTEIN	VI.D
RSp1597	RS02154					-0,49	0,01						TRANSMEMBRANE TRANSPORT PROTEIN	IV.D
RSp1598	RS02155												TRANSMEMBRANE TRANSPORT PROTEIN	IV.G.2
RSp1599	RS02156							-0,77	0,05				OUTER MEMBRANE DRUG EFFLUX LIPOPROTEIN	IV.G.2
RSp1600	RS02157			0,77	0,00	-0,90	0,00						TRANSMEMBRANE	VI.D
RSp1601	RS02158			-1,74	0,05	1,99	0,00	2,62	0,00				CONSERVED HYPOTHETICAL PROTEIN	VI.C
RSp1602	RS02159			-0,53	0,00	-0,42	0,05			0,99	0,02		HYPOTHETICAL PROTEIN	VI.D
RSp1603	RS02160							1,39	0,00	1,21	0,00		HYPOTHETICAL PROTEIN	VI.B
RSp1604	RS02161					0,34	0,02	2,21	0,00	1,49	0,00		TRANSMEMBRANE	VI.D
RSp1605	RS02162					0,60	0,03	1,63	0,00				HYPOTHETICAL PROTEIN	VI.D
RSp1606	RS02163					0,21	0,01	1,56	0,00	1,27	0,04		TRANSMEMBRANE	VI.D
RSp1607	RS02164							2,50	0,00				HYPOTHETICAL PROTEIN	VI.D
RSp1608	RS02165							1,45	0,00				HYPOTHETICAL PROTEIN	VI.D
RSp1609	RS02166							1,68	0,03				TRANSMEMBRANE ACID SPHINGOMYELINASE-LIKE PHOSPHODIESTERASE	VI.C
RSp1610	RS02167	-4,43	0,00			-0,44	0,00	1,99	0,00	2,68	0,00		HYPOTHETICAL PROTEIN	VI.D
RSp1611	RS02168											gabD2	OXIDOREDUCTASE SUCCINATE-SEMIALDEHYDE DEHYDROGENASE	I.F.18
RSp1612	RS02169					-0,42	0,01					ttuC	OXIDOREDUCTASE TARTRATE DEHYDROGENASE	I.I.4
RSp1613	RS02170					-0,42	0,02					hpaI2	2,4-DIHYDROXYHEPT-2-ENE-1,7-DIOIC ACID ALDOLASE	I.I.4
RSp1614	RS02171												TRANSMEMBRANE TRANSPORT PROTEIN	IV.D
RSp1615	RS02172											uxaA	ALTRONATE HYDROLASE (ALTRONIC ACID HYDRATASE)	I.I.4
RSp1616	RS02173												TRANSCRIPTION REGULATOR TRANSCRIPTION REGULATOR	VI.A
RSp1617	RS02174												TRANSCRIPTION REGULATOR COG1475 TRANSCRIPTION REGULATOR	VI.A
RSp1618	RS02175							-1,09	0,01				HYPOTHETICAL PROTEIN	VI.D
RSp1619	RS02176												PHOSPHOADENOSINE PHOSPHOSULFATE SULFOTRANSFERASE	I
RSp1620	RS02177												SURFACE-EXPOSED ADHESIN PROTEIN	III.A
RSp1621	RS02178					-0,83	0,00					rcsB	TRANSCRIPTION REGULATOR CAPSULAR SYNTHESIS RESPONSE REGULATO	III.A.1
RSp1622	RS02179											rcsC	TRANSCRIPTION REGULATOR CAPSULAR SYNTHESIS REGULATORY PROTEIN	III.A.1
RSp1623	RS02180			-0,66	0,05	-0,37	0,05						RESPONSE REGULATOR WITH EAL DOMAIN	VI.A
RSp1624	RS02182							-1,72	0,01			tISTRso1	REMNANT OF A ISRso16-TRANSPOSASE ORFA	V.A
RSp1626	RS02184							0,29	0,05	-0,84	0,00		CONSERVED HYPOTHETICAL PROTEIN	VI.C
RSp1627	RS02185									-0,40	0,01		CONSERVED HYPOTHETICAL PROTEIN	VI.C
RSp1628	RS02186					-0,51	0,00	-1,01	0,02	1,10	0,04	SRso16	ISRso16-TRANSPOSASE ORFB	V.A
RSp1629	RS02187							-1,98	0,01			tISRso16	C-TERMINUS OF tISRso16 ORFA TRANSPOSASE	V.A
RSp1630	RS02188												REMNANT OF CYANATE LYASE	VI.C
RSp1632	RS02190											oprB	TRANSMEMBRANE PORIN B PRECURSOR OUTER (GLUCOSE PORIN)	IV.D.3
RSp1633	RS02191							0,74	0,00			xylF	ABC TRANSPORTER D-XYLOSE-BINDING PERIPLASMIC PROTEIN	IV.D.3
RSp1634	RS02192											xylG	ABC TRANSPORTER D-XYLOSE ATP-BINDING PROTEIN	IV.D.3
RSp1635	RS02193					-0,37	0,05					xylH	ABC TRANSPORTER XYLOSE TRANSMEMBRANE PROTEIN	IV.D.3
RSp1636	RS02194												OXIDOREDUCTASE COG0451, Nucleoside-diphosphate-sugar epimerase	I
RSp1637	RS02195					-0,39	0,02						TRANSCRIPTION REGULATOR TRANSCRIPTION REGULATOR	VI.A
RSp1638	RS02196							1,42	0,03				TRANSMEMBRANE COG0697, Permeases of the drug/metabolite transporter (DMT) :	IV.D
RSp1639	RS02197												TRANSCRIPTION REGULATOR TRANSCRIPTIONAL REGULATOR	VI.A
RSp1640	RS02198					-1,25	0,00			-1,35	0,00		TRANSMEMBRANE LIPOPROTEIN	VI.C
RSp1641	RS02199					-1,33	0,00	-0,39	0,01	-1,30	0,00		TRANSMEMBRANE	VI.C
RSp1642	RS02200					-1,14	0,00	-1,12	0,00	-2,29	0,00		TRANSMEMBRANE	VI.C
RSp1643	RS02202					-1,79	0,00	-1,18	0,00	-1,34	0,01		HYPOTHETICAL PROTEIN	VI.D
RSp1644	RS02203					0,61	0,03						TRANSCRIPTION REGULATOR TRANSCRIPTION REGULATOR	VI.A
RSp1645	RS02204					-0,67	0,00						CONSERVED HYPOTHETICAL PROTEIN	VI.C
RSp1646	RS02205					-0,40	0,01			-0,52	0,01		SIGNAL PEPTIDE	VI.C
RSp1647	RS02206			0,50	0,00								COG1352, Methylase of chemotaxis methyl-accepting proteins	IV.C
RSp1649	RS02208												TRANSMEMBRANE	VI.D
RSp1650	RS02209					-0,51	0,00						TRANSCRIPTION REGULATOR COG5662, Predicted transmembrane transcriptional	VI.A
RSp1651	RS02210					-0,59	0,01						TRANSCRIPTION REGULATOR RNA POLYMERASE SIGMA FACTOR	I.E.1
RSp1652	RS02211					-1,16	0,00						COG1409, Phosphohydrolase	I
RSp1653	RS02212												OXIDOREDUCTASE Cytochrome C oxidase subunit II	I.G.1
RSp1654	RS02213	0,37	0,03	0,43	0,00	-1,47	0,00	1,22	0,00	1,68	0,00		SIGNAL PEPTIDE	VI.D
RSp1655	RS02214					-0,43	0,00			0,99	0,00		HYPOTHETICAL PROTEIN	VI.D
RSp1656	RS02215			0,63	0,00	-0,64	0,00			0,75	0,04		CONSERVED HYPOTHETICAL PROTEIN	VI.C
RSp1657	RS02216					-0,51	0,00						CYCLOPROPANE-FATTY-ACYL-PHOSPHOLIPID SYNTHASE	I.C
RSp1658	RS02217			0,80	0,05	-0,51	0,00	-0,92	0,05				COG0824, Thioesterase	I
RSp1659	RS02218			0,42	0,04			0,34	0,02				ACYL-CARRIER-PROTEIN	I.B.1
RSp1660	RS02219			0,82	0,02	-0,38	0,01						TRANSMEMBRANE	VI.D
RSp1661	RS02220			0,45	0,04								CONSERVED HYPOTHETICAL PROTEIN	VI.C
RSp1662	RS02221											prfC	PEPTIDE CHAIN RELEASE FACTOR RF-3	II.B.7
RSp1663	RS02222					-1,06	0,00	-0,52	0,00	-0,93	0,00		CONSERVED HYPOTHETICAL PROTEIN	VI.C
RSp1664	RS02223												TRANSMEMBRANE RIBONUCLEASE RNASE BN (RBN)	II.A.2
RSp1665	RS02224												TRANSMEMBRANE	VI.D
RSp1666	RS02225							0,85	0,04	1,66	0,00		SIGNAL PEPTIDE	VI.B
RSp1667	RS02226	-0,59	0,04										TRANSCRIPTION REGULATOR SIGMA-54 INTERACTING RESPONSE REGULATC	VI.A
RSp1668	RS02227							0,96	0,00				TRANSCRIPTION REGULATOR SIGMA-54 INTERACTING PROTEIN	VI.A
RSp1669	RS02228									1,10	0,00		HYPOTHETICAL PROTEIN	VI.D
RSp1670	RS02229			-1,11	0,04					2,43	0,00		SIGNAL PEPTIDE	VI.D
RSp1671	RS02230											rpoN2	TRANSCRIPTION REGULATOR RNA POLYMERASE SIGMA N (SIGMA 54) FACTOI	I.E.1
RSp1672	RS02231									0,92	0,00		COG3233, Deacetylase	I
RSp1673	RS02232					-0,30	0,02						TRANSMEMBRANE LIPOPROTEIN	VI.D
RSp1674	RS02233												HYPOTHETICAL PROTEIN	VI.D
RSp1676	RS02235												TRANSCRIPTION REGULATOR COMPOSITE TWO-COMPONENT REGULATORY I	VI.A
RSp1677	RS02236												INTEGRASE / RECOMBINASE	V.A
RSp1678	RS02237			0,70	0,00	-0,45	0,00						TRANSMEMBRANE COG2252, Permease	IV.D
